# BEST-CSP Benchmark Study of Polymorphs I and II of Sulfamerazine and the Perils of Polytype Polymorphs

**DOI:** 10.1021/acs.cgd.5c01406

**Published:** 2025-12-12

**Authors:** William P. Wood, Mihails Arhangelskis, Erika Bartůňková, Carlos E. S. Bernardes, A. Daniel Boese, Doris E. Braun, Dejan-Krešimir Bučar, Helena Butkiewicz, Ctirad Červinka, Bartolomeo Civalleri, Nicolas Couvrat, Erik de Ronde, Lorenzo Donà, Martin Dračínský, Dzmitry Firaha, Michal Fulem, Reynaldo Geronia II, Natalia Goncharova, Marlena Gryl, Johannes Hoja, Anna Hoser, Joanna Krzeszczakowska, Alexander List, Ivor Lončarić, Bruno Mladineo, Jonas Nyman, Edgar Olehovics, Mattia Raimondo, Ivo B. Rietveld, Rute I. S. Rodrigues, Luca Russo, Matteo Salvalaglio, Mafalda Sarraguça, Jiří Šnajdr, Vojtěch Štejfa, Guangxu Sun, Paul Tinnemans, Pamela S. Whitfield, Zhuocen Yang, Yizu Zhang, Sarah L. Price

**Affiliations:** † Department of Chemistry, University College London, 20 Gordon St, London WC1H 0AJ, U.K.; ‡ Faculty of Chemistry, University of Warsaw, Pasteura 1, 02-093 Warsaw, Poland; § Institute of Organic Chemistry and Biochemistry, Czech Academy of Sciences, Prague 6 160 00, Czech Republic; ∥ Centro de Química Estrutural, Institute of Molecular Sciences, Departamento de Química e Bioquímica, Faculdade de Ciências, Universidade de Lisboa, 1749-016 Lisboa, Portugal; ⊥ Department of Chemistry, 27267 University of Graz, Heinrichstrassse 28, Graz 8010, Austria; # University of Innsbruck, Institute of Pharmacy, Pharmaceutical Technology, Josef-Moeller-Haus, Innrain 52c, A-6020 Innsbruck, Austria; ∇ Christian Doppler Laboratory for Advanced Crystal Engineering Strategies in Drug Development, Institute of Pharmacy, University of Innsbruck, 6020 Innsbruck, Austria; ○ Department of Physical Chemistry, University of Chemistry and Technology, Prague, Technická 5, CZ-166 28 Prague 6, Czech Republic; ◆ Department of Chemistry, Via Pietro Giuria 7, 10125 Turin, Italy; ¶ University of Rouen Normandy, Normandy University, SMS laboratory (UR 3233), 76000 Rouen, France; †† Radboud University Nijmegen, Institute for Molecules & Materials, Department of Solid State Chemistry, Heyendaalseweg 135, 6525 AJ Nijmegen, The Netherlands; ‡‡ Avant-Garde Materials Simulation, Alte Str. 2, Merzhausen 79249, Germany; §§ Jagiellonian University, Faculty of Chemistry, Gronostajowa 2, 30-387 Krakow, Poland; ∥∥ Ruđer Bošković Institute, Bijenička Cesta 54, 10000 Zagreb Croatia; ⊥⊥ The Cambridge Crystallographic Data Centre, 12 Union Road, Cambridge CB2 1EZ, U.K.; ## Department of Chemical Engineering, University College London, London WC1E 7JE, U.K.; ∇∇ GSK Medicines Research Centre, Gunnels Wood Road, Stevenage, Hertfordshire SG1 2NY, U.K.; ○○ LAQV, REQUIMTE, Departamento de Ciências Químicas, Faculdade de Farmácia, Universidade do Porto, Rua de Jorge Viterbo Ferreira, 228, 4050-313 Porto, Portugal; ◆◆ XtalPi Inc (Shenzhen Jingtai Technology Co., Ltd.), International Biomedical Industrial Park (Phase II), 3F, 2 Hongliu Rd, Futian District, Shenzhen 518038, China; ¶¶ Excelsus Structural Solutions, Parkstrasse 1, 5234 Villigen, Switzerland

## Abstract

We report the outcome of an interdisciplinary investigation, by the BEST-CSP network, of the kinetically favored form I and the low-temperature stable form II polymorphs of the drug sulfamerazine (SMZ). Form II can be reproducibly obtained by slurrying in acetonitrile­(MeCN)/water at room temperature, though seeding with form II significantly speeds up the conversion. New structure determinations have been obtained for both forms over a wide temperature range, with both single crystal and powder X-ray diffraction methods. Room temperature FT-IR and solid-state ^13^C NMR spectra are provided. The enantiotropic but practically irreversible crystal-to-crystal transition from form II to form I is observed at temperatures ranging from 150 to 170 °C in various differential scanning calorimetry (DSC) experiments, depending on sample and heating rate. The enthalpy of transition at 150 °C is measured as Δ_trs_ *H*_m_(II → I) = 3.15 ± 0.12 kJ mol^–1^. The differences in the heat capacities mean that the DSC measured enthalpies vary with the onset temperature by about 0.55 kJ mol^–1^ over the range of heating rates commonly used in DSC experiments. Attempts to find the solvent-mediated transition temperature were complicated by observing that slurrying experiments in both methanol and MeCN/H_2_O above 50 °C produce a new, late-identified polymorph, sulfamerazine form V, which is closely related to form I but with an alternative packing of the double layers, i.e., is a polytype polymorph. Forms I and V are only easily distinguishable by high-quality powder X-ray diffraction. Form V appears to be marginally more stable than form I across the temperature range studied. The experimental data, including heat capacities and thermal expansion rates, are used to test a wide range of assumptions and energy models for calculating free energy differences between these polymorphs, illustrating the challenges in computationally modeling the thermodynamic transition temperature between form I and II. The implications of the discovery of form V on establishing the phase diagram of sulfamerazine are discussed.

## Graphical abstract

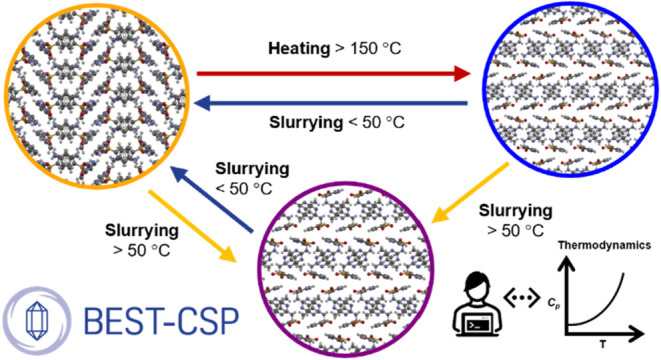

## 1 Introduction

Sulfamerazine, 4-amino-*N*-(4-methylpyrimidin-2-yl) benzenesulfonamide (SMZ), is a pharmaceutically active sulfonamide, once used as an antibiotic as part of a triple sulfa-drug combination of sulfamerazine, sulfamethazine and sulfadiazine.^1^ There has been a variety of experimental work on the ambient thermodynamically stable form II and the kinetically favored form I over the last three decades, establishing that they are enantiotropically related. However, the transformation of form II to the high temperature form I in the solid state occurs at about 150–170 °C, but slurrying experiments gave a significantly lower transition temperature around 50 °C.^2^ Furthermore, during a recent workshop, the computational working group of the EU-funded COST action (CA22107) “Bringing Experiment and Simulation Together in Crystal Structure Prediction” (BEST-CSP) (https://best-csp.eu), found (SI Figure 2.1) that the lattice energy of form II was 5–12 kJ mol^–1^ more stable than form I for some commonly used periodic dispersion-corrected density functional methods (e.g., PBE-D). This is large compared with the PBE-D2 lattice energy differences between nonconformational polymorphs,^3^ let alone enantiotropically related polymorphs. Hence, this BEST-CSP collaborative study aimed to investigate the relationship between these well-established polymorphs of sulfamerazine, with the goal of obtaining reliable experimental data between polymorph pairs that can be used to benchmark computational methods, ultimately leading to more reliable crystal structure prediction (CSP) methods. The need for experimental benchmark thermodynamic data is shown by the 2018 Faraday Discussion on CSP^4,5^ and the Cambridge Crystallographic Data Centre (CCDC) Blind Tests of CSP.^6–8^

Form I is orthorhombic with space group *Pna2*_1_ and *Z*′ = 2, form II is also orthorhombic with space group *Pbca* and *Z*′ = 1. There are other known polymorphs of SMZ. Form III^9^ (SLFNMA03) was originally crystallized from DMF and an attempt to reproduce this was unsuccessful.^10^ Crystals of form III have been observed at UCT Prague by vacuum sublimation (concomitant with form I) and at the University of Porto by crystallization from acetone at 4 °C (looks phase pure) during the project. Form IV (SLFNMA06) crystallized from ethanol with the addition of ammonia,^11^ appears to be very difficult to reproduce.^12^ In the slurrying experiments (Section 2.4), a new form was detected, which was initially solved from powder X-ray diffraction, and later confirmed by single crystal X-ray diffraction (Section 2.5.1), to give a structure with high structural resemblance to form I, but distinct enough to be considered polymorph V.

All of the experimentally observed forms of SMZ contain the same hydrogen-bonded *R*_2_^2^(8) dimer motif between the amide N–H and a N of the pyrimidine ring. This base unit is exactly centrosymmetric in form II and approximately so in form I.^13^ Each dimer is bound to another by amino NH···O=S, forming layers with no hydrogen bonding between the layers. In form II, the layers have a herringbone packing (Figure 1A) and are closer packed. The rougher interconnect in form II, in contrast to the slip planes in form I, is reflected in the bulk properties, such as form I being more millable^14^ and compressible^13^ than form II, as there is a much smaller barrier for movements between the layers. The difference in packing between form I and II does not lead to any obvious transformation pathway that maintains periodic symmetry.

**Figure 1:**
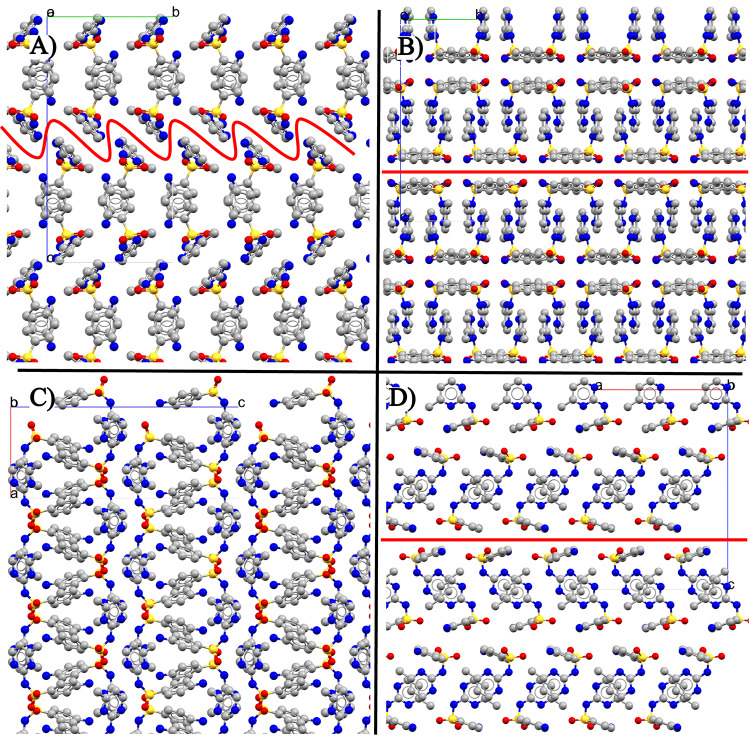
SMZ experimental crystal structures of low-temperature stable form II (SLFNMA01) viewed along *a*-axis (A) and *b*-axis (C). High-temperature form I (SLFNMA04) viewed along *a*-axis (B) and *b*-axis (D). The red lines on (B and D) form I correspond to slip planes along the (002) plane. This is compared with more rugged layering of the corresponding interface in form II (A). All hydrogen atoms are hidden for clarity.

Forms I, V and III share similar 2D layers with a lack of hydrogen bonding or other significant intermolecular interactions between the layers, resulting in the slip-planes that have been investigated for form I.^10^ Figure 2 shows that form V is a polytype of form I, with a layer reorientation at the third layer along the axis perpendicular to the slip planes, which is a more significant structural change than a simple translation of the layers.

**Figure 2:**
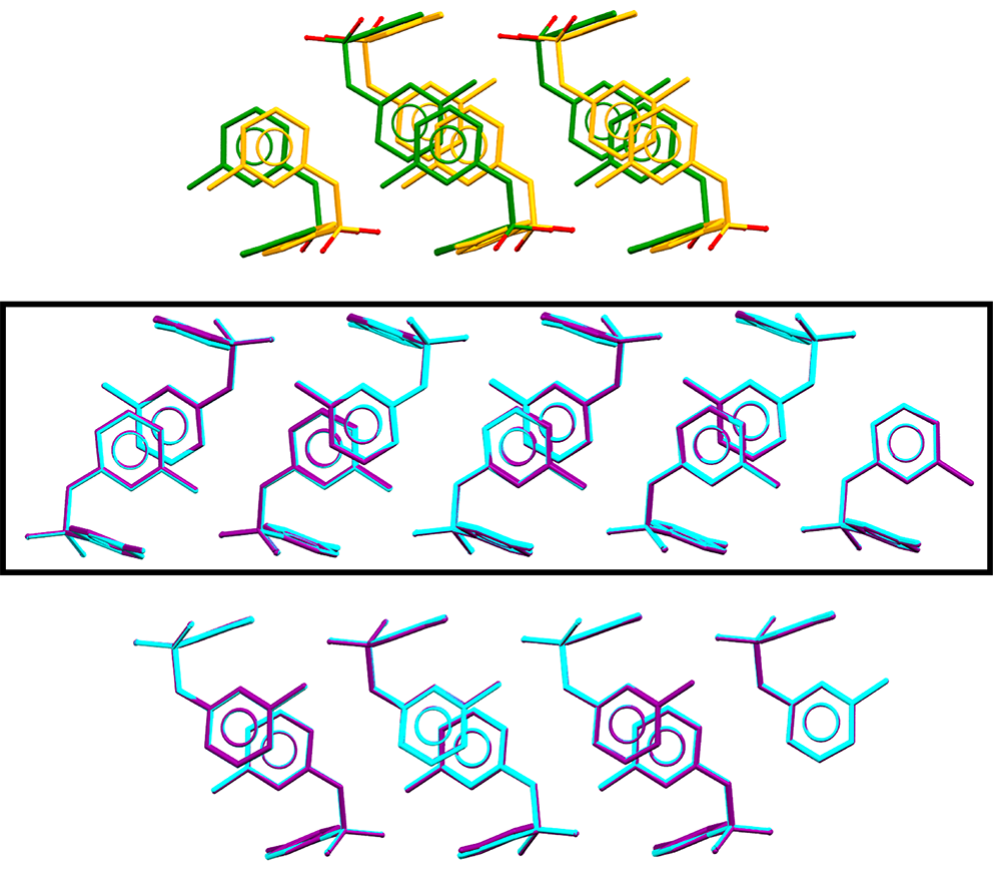
Thirty molecule crystal structure packing overlay of form I (purple) and form V (cyan) with missed matches of form I in orange and form V in green with one ‘layer’ shown in the box. In the mismatched layer, oxygen atoms are shown in red to emphasize the opposing orientations of the SO_2_ groups in molecules occupying equivalent positions in the two forms, a significant change that would be required to interconvert forms V and I. This overlay of 27/30 molecules in forms I and V has an RMSD_27_ = 0.065 Å. The central layer is also found in form III, with the overlay with form I and III being RMSD_17_ = 0.343 Å, but between forms III and V RMSD_17_ = 0.305 Å. All overlays RMSD_*n*_ are the *n*/30 molecules matched by Compare Crystal Packing Similarity function in Mercury.^15^

## 2 Outline of Experimental Methods and Results

### 2.1 Materials

Different starting materials were obtained for sulfamerazine (SMZ): from Toku-e, lot: S033–01, *≥*99.0% and analyzed by PXRD to confirm the form as form I at UCL; from Bayer, lot 703A, ≥ 99.0% and confirmed by PXRD to be form I at Innsbruck; from Aldrich, batch: WXBD7819V, *≥*99.9% and determined to be form I by PXRD at UCT Prague; from Sigma-Aldrich, lot: WXBD7819V, ≥99.0% and determined to be form I by PXRD at the Radboud University. SMZ form I was used as obtained in all cases. Solution ^1^H NMR determined that the UCL, Porto and Radboud samples contained about 1% impurities (SI Section 1.9). After our identification of form V, it was realized that it is possible that the samples used as received of form I contained some amount of form V as discussed in Section 4.2.

### 2.2 Crystallization of Polymorphs

SMZ form II was generated by slurrying the Toku-e material in MeCN/water (80:20, v/v) at room temperature for 1 week. This batch was later used to generate more material by the same method using the initial batch of form II as seeds. Using 10% weight of SMZ form II as seeds accelerated the complete transition of form I to II in 1 day, as confirmed by PXRD. This recipe was repeated by UCT Prague, the University of Porto and Radboud University, however, one attempt to produce form II by this procedure instead resulted in the formation of form V as determined by single crystal X-ray diffraction at Radboud (SI Section 1.2.2). In the case of Innsbruck their sample of form II was produced by slurrying in MeCN/water (80:20, v/v) and temperature cycled between 10 and 30 °C for 4 days.

Attempts to produce form II by crystallization from acetone, as previously reported,^16^ were successful, but yielded concomitant formation of an acetone solvate. The acetone solvate has a very similar structure to the dimethylformamide, dimethylacetamide and 3-picoline solvates, with the solvent being packed between the layers of sulfamerazine, which have similar layers comprised of hydrogen bonded dimers to forms I, III and V (SI Section 1.2.3).

SMZ form V was obtained by slurrying form I and/or form II in MeCN/water (80:20, v/v) at 60 °C for 2 days as described in Section 2.4.

### 2.3 NMR and IR Spectroscopy

The solid-state ^13^C NMR reference spectra in Figure 3 show that the form I spectrum only shows significant splitting at C3, despite being a *Z*′ = 2 structure, consistent with it having only slightly symmetry-broken inversion (Section 1). The form II spectrum shows broad signals for C2 and C3, suggesting rotation of the phenyl ring and averaging of the signals of two nonequivalent sites. The ^13^C ss-NMR spectrum of form V was also measured (SI Figure 1.5.1), but it is identical to form I, so it cannot be used to distinguish between them. The proton T1 values of form II are substantially longer than those of form I and the measurement of ^15^N spectra is not feasible. Full description of the methodology can be found in SI Section 1.5.

**Figure 3:**
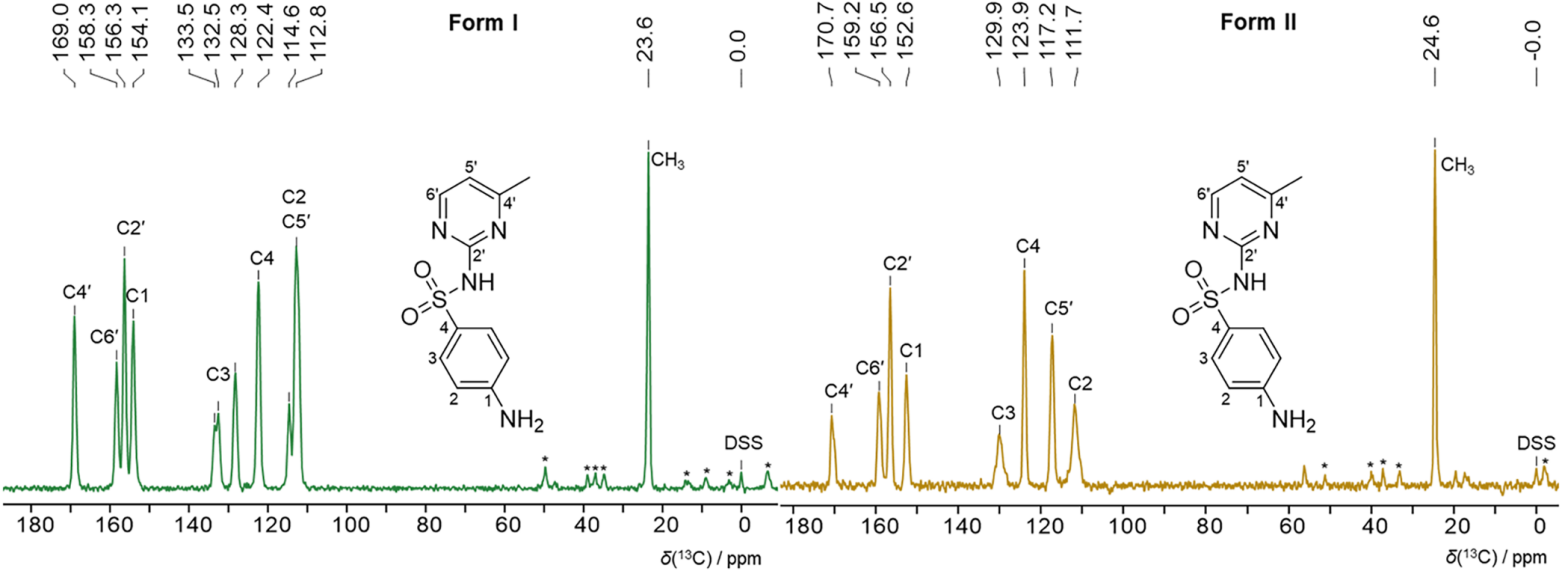
Experimental ^13^C CP-MAS solid-state NMR spectrum of sulfamerazine form I (left) and II (right). The asterisks indicate spinning side bands. Spectra measured at room temperature but friction heating means the sample is ∼40 °C when measured.

The FT-IR spectra (Figure 4) of forms I and V are almost indistinguishable with a small difference of an additional peak in the form V spectra at ∼940 cm^–1^, with peak splitting being observed demonstrating the *Z*′ = 2 nature of forms I and V. Form II, however, shows significant differences in the high frequency region, around 3500 cm^–1^ (N–H stretching) that reflect the differences in length and strength of the hydrogen bonding motif. This is a practical, easy method of clearly distinguishing forms I and II, with forms I and V having a more subtle difference that is not easy to spot.

**Figure 4:**
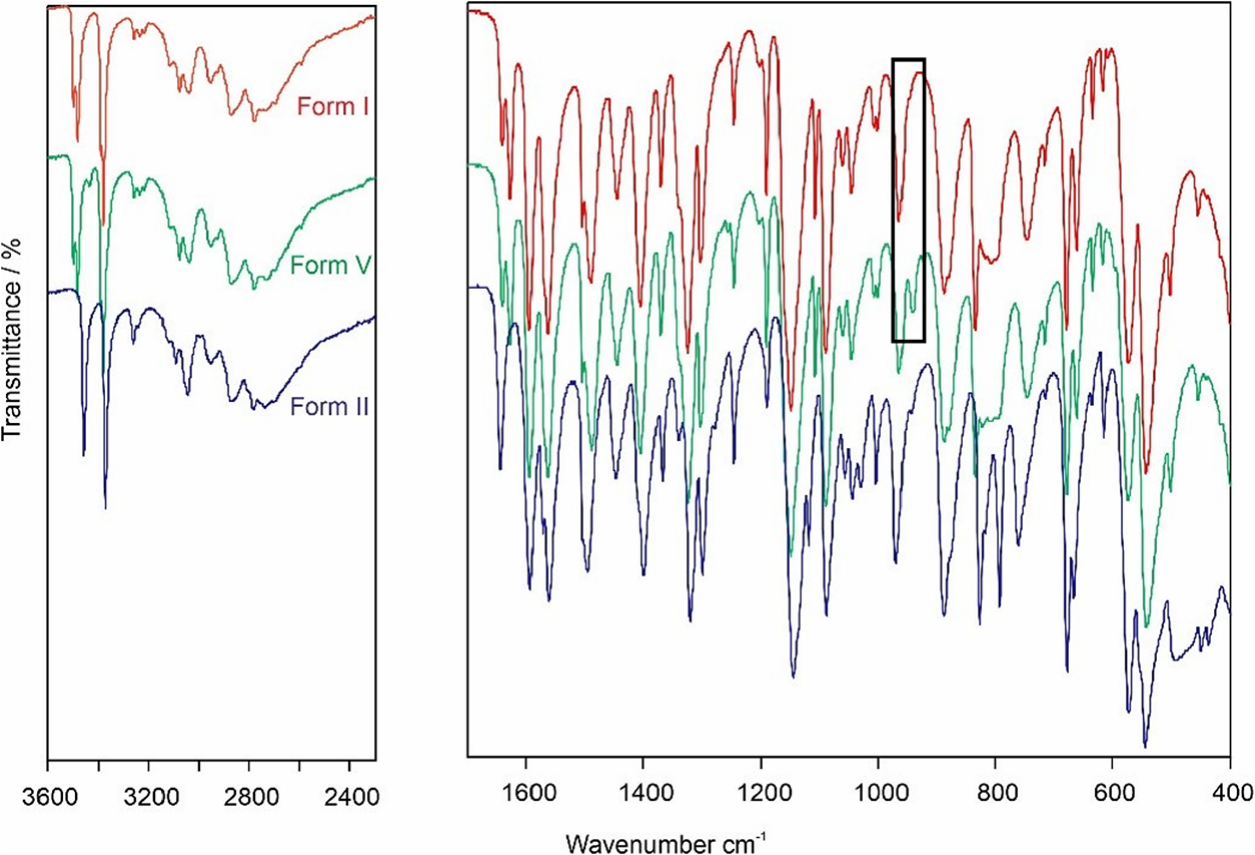
FTIR spectra of forms I, V, and II as measured in Innsbruck (Vertex 70 FTIR spectrometer, 4000 and 400 cm^–1^, resolution of 2 cm^–1^, 32 scans per spectrum). The spectrum of form V is almost indistinguishable from that of form I, an additional peak in the form V spectrum at ∼940 cm^–1^ is highlighted in the black box.

### 2.4 Slurry Experiments

Competitive slurrying experiments were conducted in two laboratories (UCL and Innsbruck), covering various solvents and a wide range of temperatures and conditions, with all the results listed in SI Section 1.4. Generally, binary or ternary mixtures of the polymorphs were produced by mixing equal weights of the forms. These mixtures were then combined with solvent and stirred either in a parallel crystallizer (Crystal 16) or in a 14 mL vial immersed in an oil bath at the specified temperature (for Innsbruck experiments, each temperature was tested in duplicate). After the designated time, a sample was filtered and analyzed by PXRD to determine its form.

Previous experiments^2^ reported a solvent-mediated transition between forms I and II between 51 and 54 °C in methanol. Our more extensive slurries have identified a new form, V, that is more stable than form I at least between 50 and 60 °C (Figure 5) in competitive slurries. The main series of competitive slurries were carried out in MeCN/H_2_O (80:20, v/v), including Figure 5, as it has been shown to favor the thermodynamically stable form II, while most solvents favor form I.^2^ At Innsbruck, starting with ternary mixtures of forms I, II and V, complete conversion to form II was observed at 45 °C and complete conversion to form V was observed at 50 °C after 2 weeks (Figure 5). Slightly finer temperature steps were investigated at UCL, which determined that form II is the most stable form up to 48 °C, with form V being the most stable at 50 °C and higher temperatures (Figure 6).

**Figure 5:**
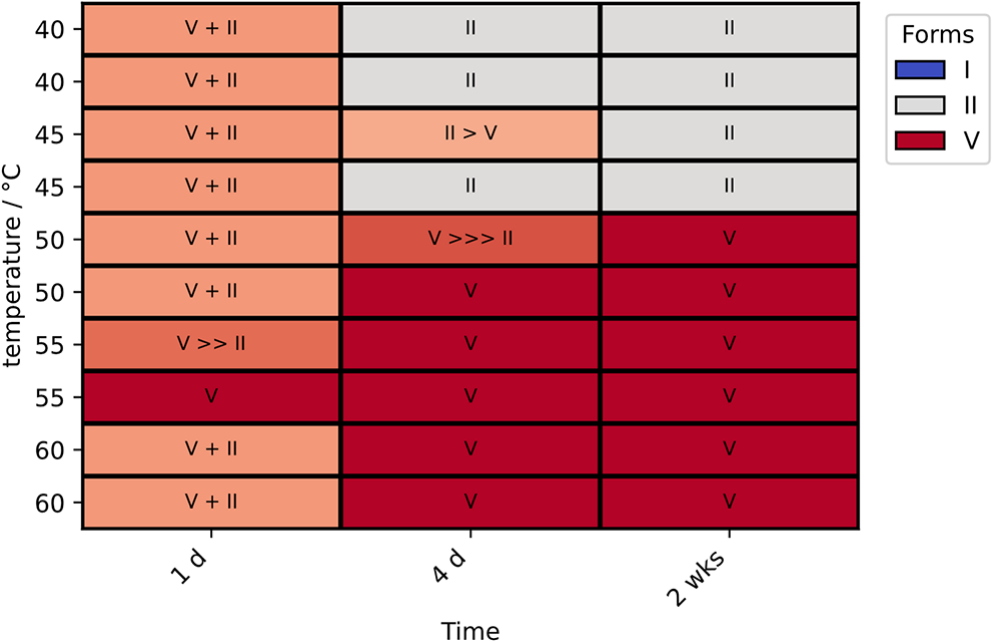
Competitive slurries of SMZ form I, II and V in MeCN/H_2_O (80:20, v/v). All inputs were equal mixtures of forms I, II and V by weight. The resulting form after the time point is shown as measured by PXRD (carried out at Innsbruck). Examples of the PXRD patterns are shown in Figure 6.

**Figure 6:**
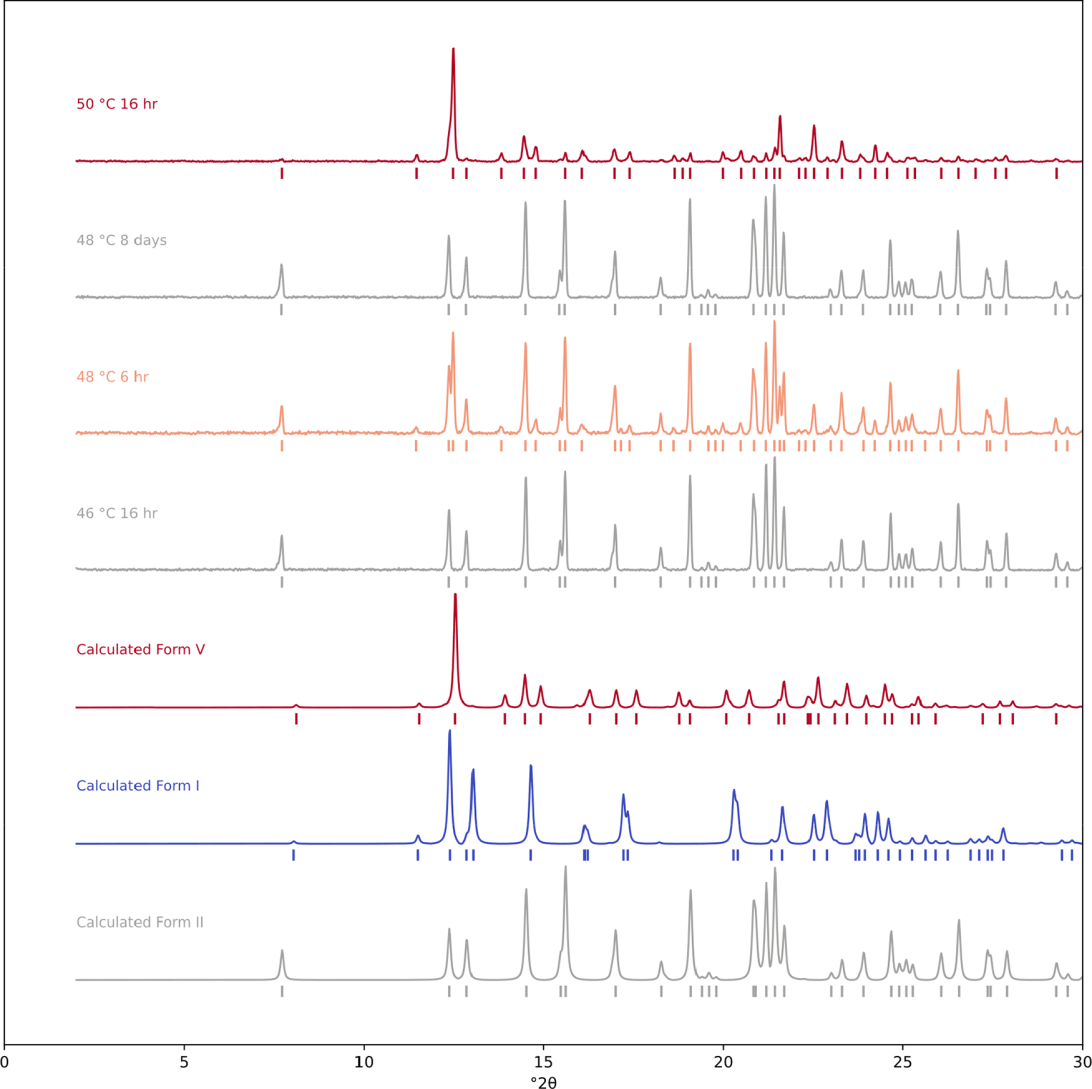
Reference Cu Kα_1_ PXRD diffractograms of form I, II and V, along with the resulting powder patterns after slurrying ternary mixtures of I, II and V in MeCN/H_2_O (80:20, v/v) at the temperature and time periods listed (carried out at UCL).

The transition in MeOH was also investigated with form I transitioning to form V between 40 and 55 °C and mixtures of forms I and II transitioning to form II at <45 °C and to mostly form V at 50 and 55 °C, all after 4 days (SI Figure 1.4.2). Slurries in MeCN/H_2_O at small temperature steps were carried out at UCL (Figure 6) and a clear and complete transformation to form II is seen at 46 and 48 °C, given long enough, and a clear and complete transformation to form V is seen at 50 °C. Thus, the thermodynamic transition temperature between form II and form V is between 48 and 50 °C. No temperature above 60 °C has been measured because the solvent systems with reasonable transition rates are approaching their boiling points (i.e., MeCN/H_2_O and MeOH).

The slurrying experiments conducted between 40 and 60 °C concluded that form V is more stable than form I between these temperatures, with form II being the most stable form at temperatures below 48 °C. Form V being the most stable above 50 °C was independently confirmed by two laboratories, UCL and Innsbruck. The emergence of form V as the most stable form above 50 °C is a new and somewhat surprising finding, given the previous study.^2^ The diffractograms of the two forms may appear very similar at first glance, owing to common features at low 2θ (Figure 6) and at high 2θ, at low amounts of form V, the distinct peaks blend in a background of more intense form I peaks, as discussed in Section 4.2. These factors may contribute to why form V has not been identified previously, especially if only mixtures have been generated. At this time, no transition into or away from form V has been observed in the solid state (DSC or VT-XRD), and so form I appears kinetically trapped.

The use of slurrying to determine the relative stability of forms V and I at higher temperatures is limited by the poor solubility of SMZ and slow kinetics in any high-boiling-point solvent.

### 2.5 Structural Data

Forms I and II readily formed good single crystals (Figure 7) unlike form V (SI Section 1.2.2).

**Figure 7:**
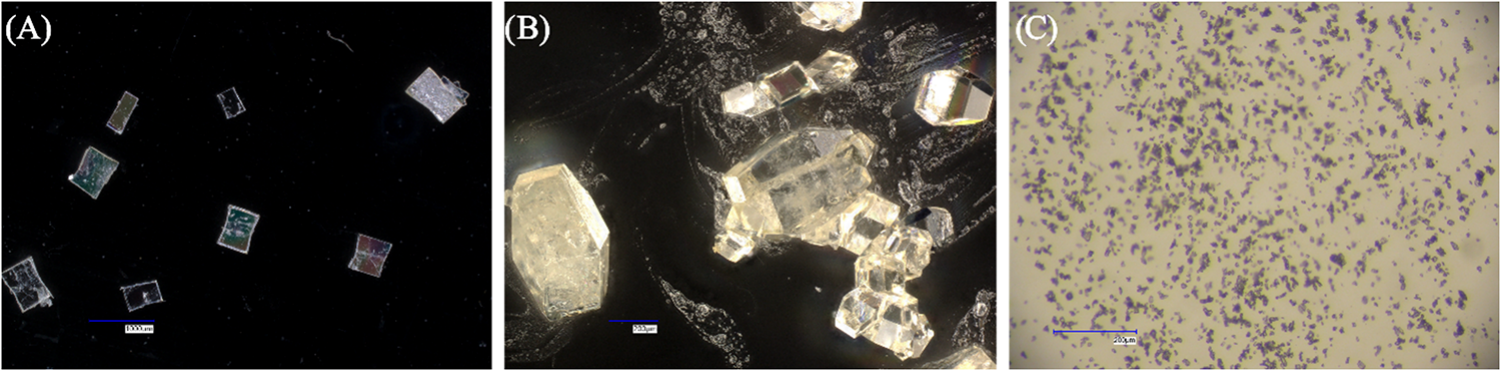
Morphologies of crystals of form I (A) and form II (B) under a digital microscope (Keyence VHX 7000N) and powder of form V (C) under an optical microscope (Leica MZ75).

#### 2.5.1 Single-Crystal X-ray Diffraction

All the single crystal structure determinations currently available for SMZ are listed in SI Table 1.1.1, along with full details of the new structure determinations from this project. This includes three determinations of the structure of form V and a high-temperature single-crystal determination for forms I and II.

Forms I and II show a similar increase in the size of ADPs with temperature from 150 to 413 K (Figure 8). Form I shows more anisotropic ADPs on the methyl-pyrimidine ring at all temperatures relative to form II, with the direction of the principal axis suggesting a wagging of the ring (not a rotation). In contrast, the aniline rings that line the slip planes show normal ADPs. Form II shows more anisotropic ADPs on the aniline ring compared to form I. The relative isotropy of the para carbons on the aniline ring versus the others suggests a rotating motion in form II.

**Figure 8:**
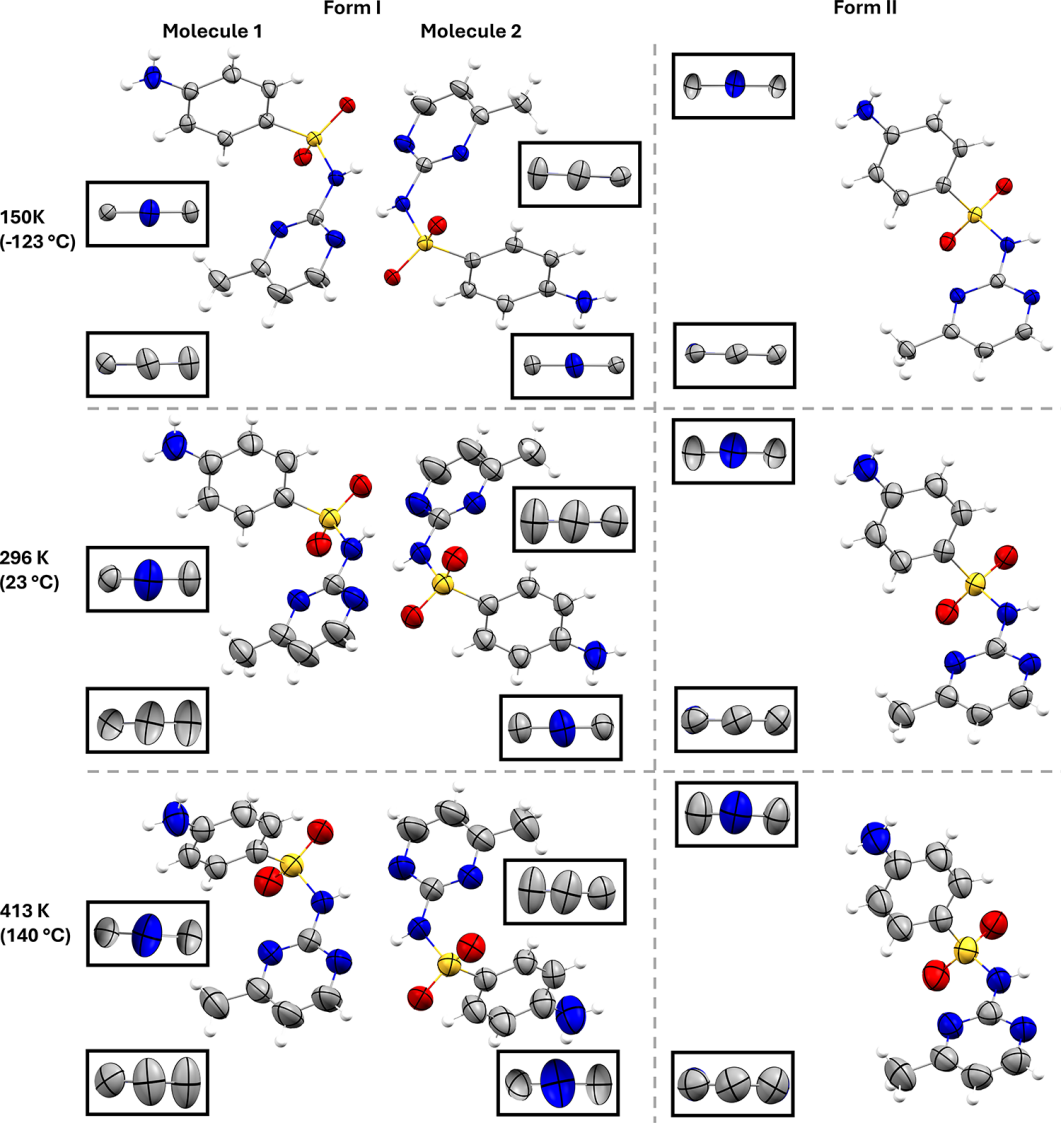
Atomic Displacement Parameters (ADPs), shown at 50% probability level, of form I and II at low (150 K), room temperature and 140 °C. Boxes show the ADPs looking down the N–C1 bond of the aniline (C2, N, C2) and the three consecutive carbons of the methyl pyrimidine rings (C4′,C5′ C6′).

#### 2.5.2 Variable Temperature Powder X-ray Diffraction

Two sets of variable temperature powder X-ray diffraction were carried out, one with long data collection times at Rouen to measure the thermal expansion, and quick data collection on a finer temperature grid at Innsbruck to determine the solid-state transition temperature (details in SI Section 1.3). In contrast to hot-stage microscopy and DSC measurements (Section 2.6.1), the powder is heated to the set point, which is held constant during the data collection.

Variable-temperature (VT) single-crystal X-ray diffraction (SC-XRD) experiments previously carried out from −123.15 to 26.85 °C (150 to 300 K) on forms I and II^10^ have been combined with variable temperature refinements of powder data measured in Rouen over the range 25 – 200 °C (298.15 – 473.15 K), in Figure 9. The largest thermal expansion in form I is along the *c* axis, perpendicular to the slip planes (Figure 1B,D) and in form II along the *b* axis between the hydrogen bonded dimers (Figure 1A) i.e., along predominantly dispersion bound directions. Form II exhibits a larger relative increase in crystal volume (4.54%) compared to form I (3.83%) between −123.15 and 143 °C. As form II exhibits a solid–solid transition into the lower density form I at ∼150 °C (Section 2.6.1), the greater expansion may indicate increasing instability of form II as the temperature approaches the transition point. The discontinuities in the form I data in Figure 9 are at least partially due to the trace presence of form V in the sample, and two alternative ways of accounting for the effect of form V contamination are discussed in the SI Section 1.3.

**Figure 9:**
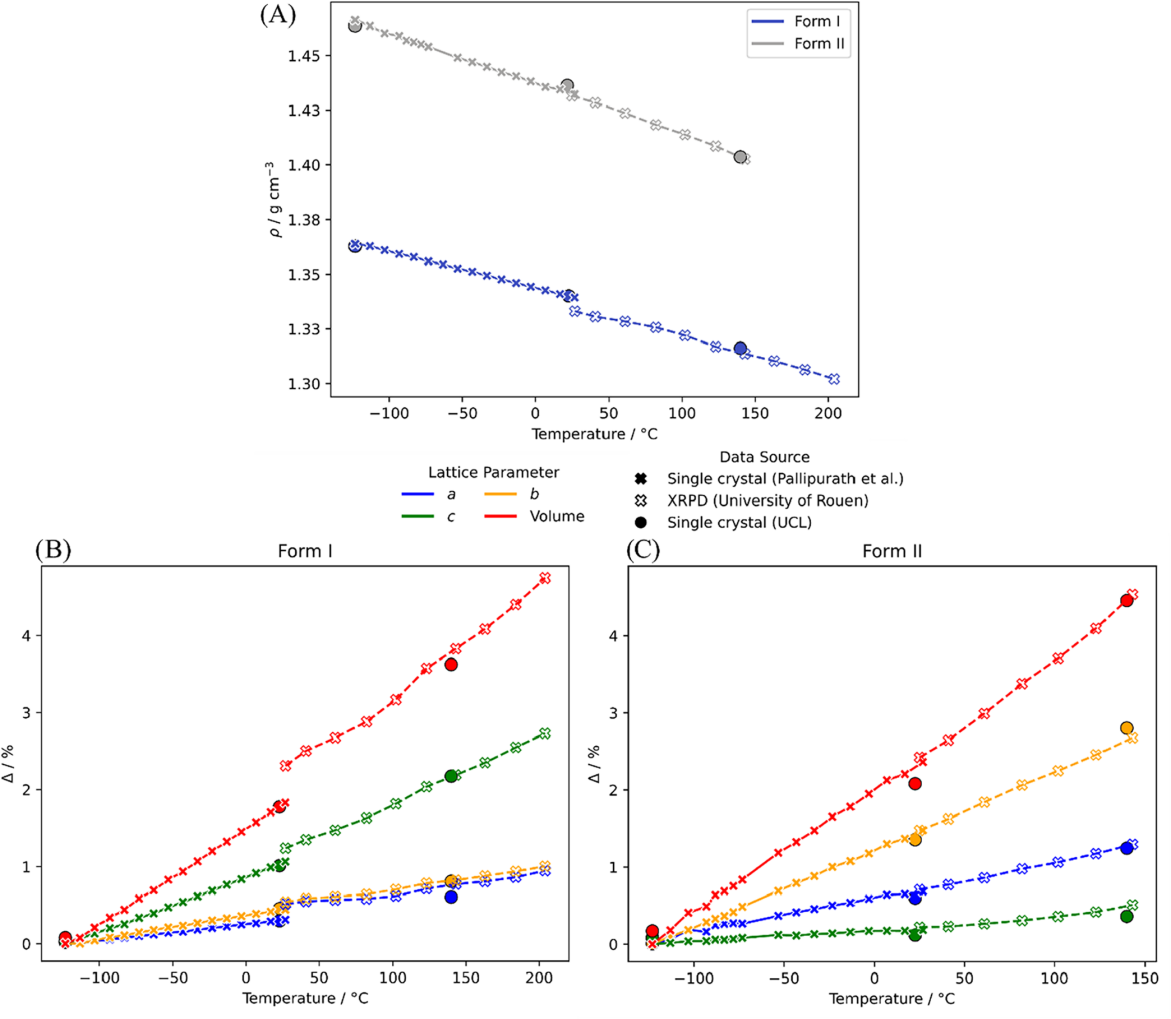
Structural data as a function of temperature, single-crystal X-ray diffraction data from Pallipurath et al.^10^ in solid crosses/lines and from UCL in solid circles, PXRD­(T) data contributed by Rouen in empty crosses/dashed lines. Densities of form I (blue) and II (gray) (A). Percentage change in the lattice parameters and volume of form I (B) and II (C).

Variable temperature PXRD of form II on heating shows a transition to form I between 150 and 160 °C (Figure 10), with no evidence of peaks of form V. In contrast, a similar experiment on form V shows no signs of any transition in the range 30 to 200 °C (SI Figure 1.3.1).

**Figure 10:**
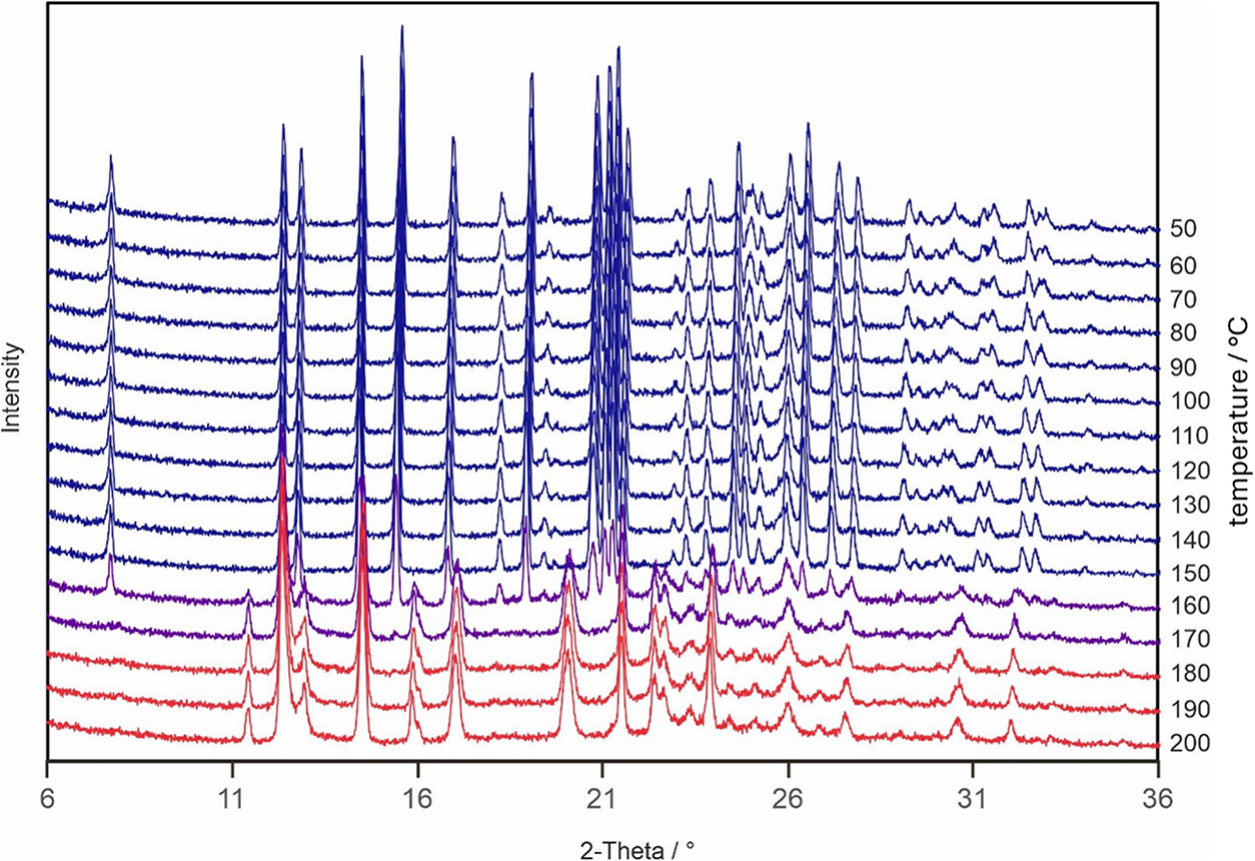
Variable temperature PXRD of form II on heating measured at Innsbruck.

### 2.6 Thermodynamic Data

#### 2.6.1 Hot-Stage Microscopy and Differential Scanning Calorimetry

The transparent crystals of form II begin to transform at approximately 160 °C, starting from defects (Figure 11). The transformation can be observed as a darkening of the crystals under cross-polarized light as the single crystal of form II transforms to microcrystalline form I and is completed at 175 °C. The transformation is clearly first order.

**Figure 11:**
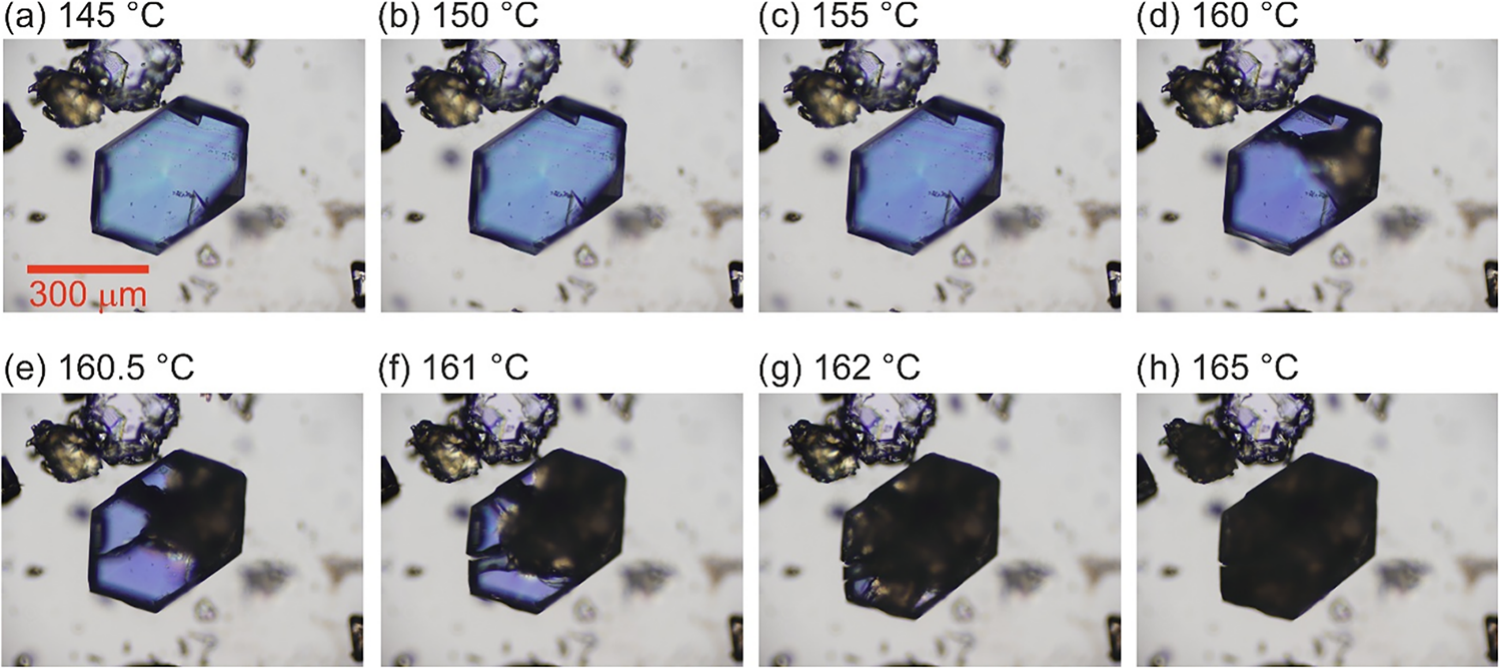
Hot stage microscopy pictures of form II transforming to domains of form I as the temperature is increased, taken under cross-polarized light, using an Olympus BH2 microscope equipped with a Kofler hot-stage and an Olympus DP71 digital camera.

Four groups independently produced samples of form II and performed DSC measurements according to the protocols in SI Section 1.6. The results (SI Table 1.6.1) are summarized in Figure 12, showing that there is a significant variation in the onset of the crystal-to-crystal transition temperature, generally rising with heating rate. The transformation was purely to form I, with no traces of form V being detected. There is no sign of the transformation being reversible down to 25 °C in the DSC experiments.

**Figure 12:**
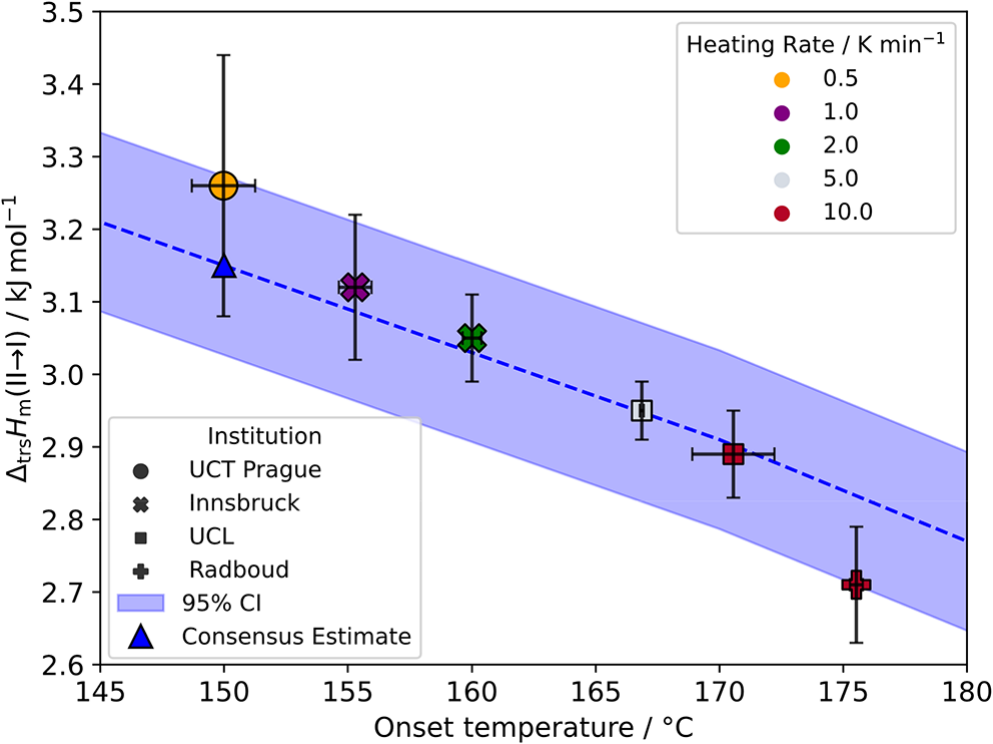
Enthalpy difference as a function of temperature as measured by DSC on SMZ form II to I transition for all institutions that measured it for BEST-CSP. Each point is made up of 5 replicates, except for UCT Prague, which is 4 replicates. The error bars are two times the standard error. Heating rates are coded by color. The consensus estimate at 150 °C is marked on the plot with the change in enthalpy as calculated from the *C*_*p*_ with 95% confidence interval shaded in blue.

The heat capacity measurements were used to estimate the enthalpy of transition from form II to I (Δ_trs_ *H*_m_(II → I)) for each measurement across all laboratories and heating rates at a reference temperature, in this case 150 °C (determined at the slowest heating rate and so the closest estimate of the transition temperature). A DerSimonian-Laird random effects model^17^ (SI Section 1.7) was then used to obtain a consensus estimate at the reference temperature with 95% confidence intervals of Δ_trs_ *H*_m_(II → I) = 3.15 ± 0.12 kJ mol^–1^ (Figure 12). The heat capacity measurements were then used to calculate the enthalpy as a function of temperature over the full temperature range using the consensus estimate as a reference point, including at ambient (Table 1).

**Table 1:** Calculated Thermodynamic Properties of Sulfamerazine Forms I and II^e^

	group^a^	lattice energy (I–II)/kJ mol^–1^	Δ_trs_ *H*_m_(II → I) at 25 °C/kJ mol^–1^	Δ_trs_ *H*_m_(II → I) at 150 °C/kJ mol^–1^	trans. temp/°C ΔG_II→I_ = 0 (ΔA_II→I_ = 0)	*C_p_* (*C*_v_) of form I at 300 K/J mol^–1^ K^–1^
type of model	BEST-CSP experimental		4.02 ± 0.21^b^	3.15 ± 0.12^b^	>48, ≪150^c^	297.1
harmonic phonons (PBE-TS)	PriceUCL	11.7	10.2	10.6	(183)	(288.3)
harmonic phonons (PBE-TS)	RussoGSK	11.4	9.6	10.0	(113)	(289.2)
harmonic phonons (PBE-TS)	XtalPi	11.3	9.7	10.1	(115)	(288.3)
harmonic phonons (PBE-D4)	Arhangelskis	5.6	3.2	3.0	(45)	(278.5)
harmonic phonons CRYSTAL^24^	TCG-UNITO	9.6	8.2	8.5	(85)	(287.6)
harmonic phonons CRYSTAL fixed cell^24^	TCG-UNITO	*7.3*	*6.6*	*6.7*	*170*	*288.2*
quasi-harmonic FF MACE-OFF^25^	Lončarić	12.3	5.5	6.9	204	279.9
quasi-harmonic FF MACE@SPICE2 ^26^	Lončarić	17.9	4.1	4.3	>227	283.1
quasi-harmonic, ME3 ^27^ light	Boese	6.8	5.1	5.1	172	284.7
quasi-harmonic, ME3 ^27^ tight	Boese	6.5	6.0	6.2	>227	284.5
quasi-harmonic, composite^28^ (PBE-D4)^d^	@CervinkaG	4.7^d^				290.19
TRHuST 23 ^29^	AMS	5.3	4.7	4.8	(13)	(289.7)
B3LYP VTZP-D3 CRYSTAL [NoMoRe^30^]	Hoser	13.6	13.5 [*13*.*4*]	13.8 [*13*.*8*]	238 [*355*]	297.8
PBE-D3 CASTEP [NoMoRe^30^]		11.9	7.3 [*7*.*3*]	7.3 [*7*.*2*]	113 [*176*]	306.4
molecular dynamics	CB@Lisbon		–0.9 ± 1.7	1.5 ± 2.7		
MD PSCP^31^	XtalPi		6.4	5.6	112	401.1
MD PGMCrys + MBAR^32,33^	MME@UCL		5.3	4.1	102 ± 26	389.3

a Names of contributing groups, as chosen to distinguish between different computational groups in same institution or computational author from other authors in same laboratory.

b Expanded uncertainties are explained in SI Section 1.7.3.

c The experimental transition temperature is probably given by the slurrying experiments and significantly less than the lowest crystal to crystal transition, as explained in Section 4.1.

d Form II calculations yielded imaginary phonons, and so only the lattice energy difference is reported.

e The only experimental data used was the crystal structures as starting point for optimization, with the exception of the methods in italics, where details of what other information was used is in the appropriate SI. The lattice energy difference is given for those methods where this is calculated and appropriate.

The consensus value aims to capture both experimental variability and systematic differences between different laboratories, providing a robust reference point for future studies and comparative analysis. The enthalpy and transition temperature values in Figure 12 compare with the literature values of Δ_trs_ *H*_m_(II → I) = 3.12 ± 0.01 kJ mol^–1^ at 175.1 ± 0.2 °C by Zhang et al.^2^ (reported as the peak temperature not onset), which is consistent with our results (within error), and the earlier determination of Δ_trs_ *H*_m_(II → I) = 1.4 ± 0.2 kJ mol^–1^ at 149–150 °C by Caira et al.,^1^ which is in significant disagreement with our results. The range of transition onset temperatures can be explained by the heating rate and the nature of the powder sample (size,^18^ impurity profile and quality of crystals etc.) as this is clearly a first-order nucleation and growth transition, requiring sufficient energy to overcome the barrier to the transition. The data shows a general trend of decreasing transition enthalpy with increasing onset temperature, consistent with the expectation that the enthalpy difference between polymorphs form II and form I should decrease at higher temperatures, as form I has a lower heat capacity compared to form II (Section 2.7). Kinetic effects are probably causing the onset to occur at higher temperatures with higher heating rates and, consequently, lower measured transition enthalpies. Ideally, one would want to estimate the transformation temperature extrapolated to zero heating rate, to give a lower bound for the transition temperature.

#### 2.6.2 Solubility by Clear Point Measurements

The solubility experiments were carried out in MeCN/H_2_O, as it is a solvent system in which SMZ is reasonably soluble and has not been shown to kinetically favor form I, as some other solvents do.^2^ The alternative solvent used during literature slurrying experiments, methanol, would be boiling at about 60 °C, and so there would be practical issues in going sufficiently above the transition temperature. The solubility was determined (SI Section 1.10) by gradually increasing temperature and measuring turbidity (clear-point, i.e, transmittance) as the end point. It does not provide true thermodynamic solubility data, as no equilibrium is reached. Therefore, it should not be used to derive thermodynamic data, but rather as a rapid method to obtain solubility estimates at different temperatures.

Figure 13 shows that the solubility curves of form II and form V cross at 43 °C. This estimates the transition temperature in this solvent (i.e., the Gibbs free energies of solution are equal for the two polymorphs). This value is slightly lower than the transition point determined by competitive slurry experiments (48–50 °C), which is probably due to the solubility method used, as the slurry experiments were conducted under equilibrium conditions. There is also a crossing between the curves of form II and form I at ∼50 °C. This is in very good agreement with the transition temperature between form II and form I in methanol as reported in Zhang et al.,^2^ though a clear form II to I transition is not observed in our slurry experiments (SI Section 1.4). Overall, the measured solubilities agree with observations from the competitive slurries, that form II is stable ≤ 40 °C and that form V is stable ≥ 50 °C, with form I being metastable at all temperatures investigated.

**Figure 13:**
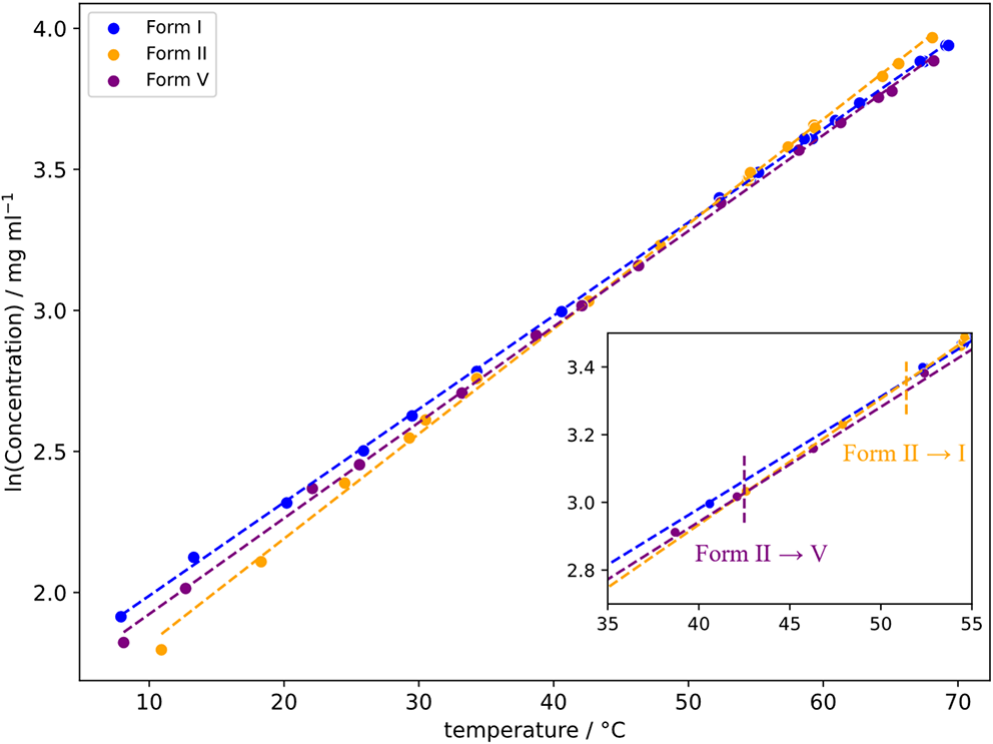
Clear-point solubility measurements of SMZ forms I, II and V in MeCN/H_2_O (80:20, v/v) as a function of temperature. All measurements were carried out in Innsbruck. The lines are fitted to the data points by a linear function. The solubility estimations suggest a transition temperature of 43 °C between II and V (marked in purple), and the transition between II and I at 51 °C (marked in orange).

### 2.7 Heat Capacity

Heat capacity values of sulfamerazine forms I and II were determined by the Tian-Calvet calorimeter and extended to higher temperature range using a power-compensation calorimeter using published methods^19,20^ (SI Section 1.8) and are shown in Figure 14. This shows that form II has a higher heat capacity and a higher rate of increase over the experimental temperature range. Based on extrapolation of the temperature trends, form II would have lower heat capacities below around −100 °C. This switch in relative heat capacities is the most common behavior for enantiotropic polymorphs. Due to the later identification of form V during this study, it cannot be comprehensively disproven that no form V was present in the sample used for the heat capacity measurement of form I.

**Figure 14:**
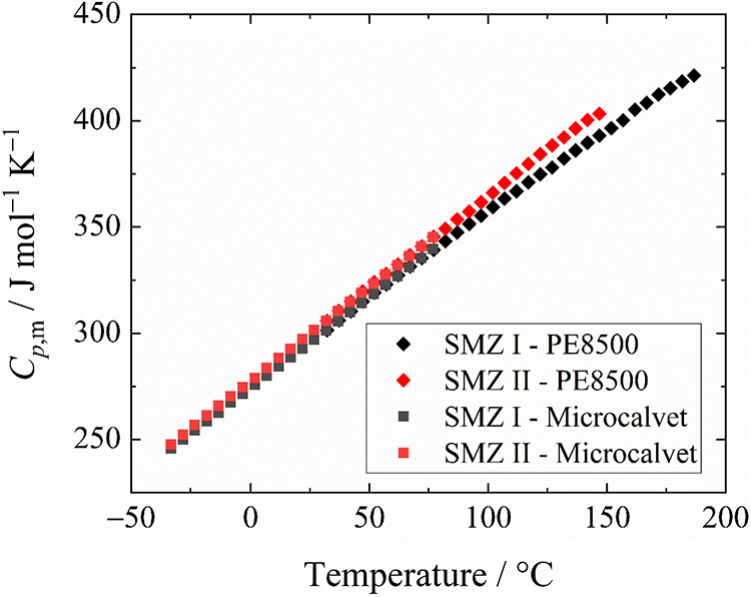
Isobaric heat capacity for forms I and II of sulfamerazine, contributed by UCT Prague.

To estimate the standard enthalpy of transition at 25 °C (Δ_trs_ *H°*_m_(II → I)) as 4.02 ± 0.21 kJ mol^–1^, the heat capacity measurements of form I and II are fitted to a quadratic function and then integrated to give the correction to the consensus transition enthalpy determined in Section 2.6.1.

## 3 Computational Modeling Results and Discussion

The modeling of enantiotropic polymorph relationships goes beyond the models used in most computational crystal structure prediction (CSP) methods, i.e., the zeroth order (CSP_0) estimates of the relative thermodynamic stability by the lattice energy differences. The lattice energy *E*_latt_ is the energy to split up an infinite static perfect lattice into its constituent static molecules, infinitely separated, and hence is not directly measurable. CSP studies minimize the lattice energy of computer-generated structures as a function of the structural variables to give the CSP_0 relative stabilities, an approach that is remarkably successful at generating the observed structures among the lowest in lattice energy. The evaluation of the lattice energies in CSP is challenging, as clearly demonstrated in the blind tests of crystal structure prediction organized by the CCDC.^8^ Attempts to estimate the absolute lattice energy of crystals of small molecules are only just converging with state-of-the-art electronic structure methods to within the errors associated with different determinations of heats of sublimation.^21^ Hence, it is not surprising that the lattice energy differences between polymorphs, which are usually the main contribution to the relative thermodynamic stability, vary significantly with the method used to model the forces between the atoms, as demonstrated for the SMZ polymorphs at the first BEST-CSP workshop (SI Section 2). The variation in the lattice energy differences is clearly a major contributor to the variation in the enthalpy difference estimates of SMZ (Table 1).

The computational methods used in CSP do not use any experimental data on the crystals of that molecule. When experimental structures are available, the structures after optimization with fixed experimental cell parameters may be used for estimating the thermodynamic properties. Some optimization is always advisable to adjust for the common systematic errors in bondlengths to hydrogen and experimental errors in other bondlengths. However, this can introduce problems: the sulfonamide group bondlengths show an unusual sensitivity to the functional used in computational modeling using periodic density functional electronic structure calculations, with the S–N and S=O bondlengths with the PBE functional being significantly longer than found in experiment, even when the room temperature bondlengths have been corrected for libration (SI Section 2.1). This can be attributed to the delocalization error in the PBE functional,^8,22,23^ which will also affect the energy associated with changing the sulfonamide angles. The PBE functional is the most commonly used in the electronic structure modeling of organic crystals in CSP, and so this indicates a source of error specific to the sulfonamide group, that has been avoided by those groups using more accurate and expensive electronic structure methods.

The neglect of the effect of temperature is obviously an approximation that is not consistent with enantiotropic phase changes. An outmoded approximation to the temperature effects assumes that the molecular conformations and vibrational modes are the same for the crystal and gas phase, the gas is ideal, and that the molar heat capacity is constant and equal for all solids (3R, Dulong-Petit law). These assumptions lead to the enthalpy of sublimation being approximated by Δ*H*_sub_ = −*E*_latt_ – 2*RT*, and so, like lattice energies, does not allow for enantiotropic relationships and implies that the enthalpy differences between the polymorphs are equal to the lattice energy differences. Hence, enantiotropic relationships require significant differences in the molecular motions within each polymorph as a function of temperature. The measurement of the heat capacities allows a test of how well the models are estimating the changes with temperature. For the computational modeling of enantiotropically related polymorphs, there are three main categories of approximations: the model for the forces between the atoms in the crystals, the model used to calculate the property, and then a variety of technical settings including level of convergence or sampling. This collaborative study includes a particularly wide range of computational methods; Table 1 and the SI Section 2 contain more details of most types of calculation and often an analysis of the effects of the technical settings and choice of model for the forces between the molecules. The discussion of the results in Table 1 will focus on the assumptions made in modeling the molecular motions.

Since the relationship between the isobaric (constant pressure), *C_p_*, and isochoric (constant volume), *C*_v_, heat capacities,

$$C_{p}\left(T\right)-C_{v}\left(T\right)=\int_{0}^{T^{\prime }}\mathrm{VK}\left(T^{\prime }\right)\alpha^{2}\left(T^{\prime }\right)dT^{\prime }$$

depends on the thermal volume expansion coefficient, α, as well as the bulk modulus, *K*, most calculations which assume the lattice energy optimized structure (i.e., ignore thermal expansion) are estimating *C*_v_, whereas *C*_*p*_ is measured experimentally.

The heat capacity terms are particularly sensitive to the low-frequency lattice modes, as a function of temperature. The flexibility of SMZ implies that the low energy lattice modes will be coupling molecular and lattice modes. These modes are below 400 cm^–1^, and so do not appear in the FT-IR spectra (Figure 4). The lattice energy calculations are on hypothetical static structures at zero pressure and temperature and so ignore the zero-point vibrations of the crystal. The pressure terms are often neglected in solid-to-solid thermodynamics. Using the cell volume per molecule of 323 Å^3^/molecule for form I and 300 Å^3^/molecule for form II, both at 150 K, the term at ambient pressure *p*Δ*V*_I→II_ = 1.4 × 10^–3^ kJ mol^–1^. The neglect of volume changes means that Gibbs Δ*G*_II→I_ and Helmholtz Δ*A*_II→I_ free energy differences between solid phases are the same. The data in Table 1 and the plot of the heat capacity differences (Figure 15) distinguish between these thermodynamic quantities.

**Figure 15:**
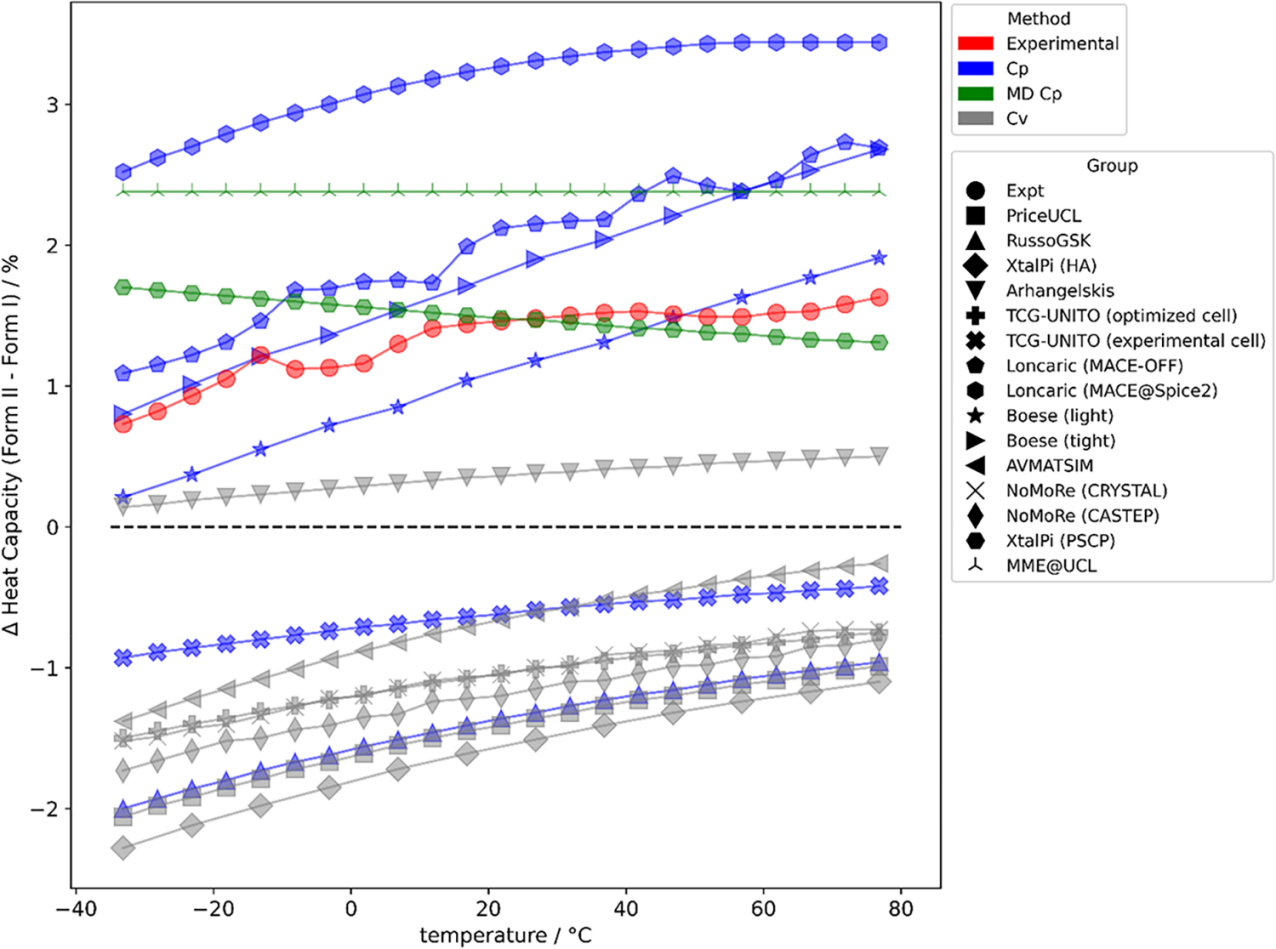
Percentage differences in calculated heat capacities between form I and II contrasted with experimental values (Figure 14) in red. Constant volume calculations in gray, constant pressure in blue and molecular dynamics constant pressure calculations in green. Curves above the dashed line at zero are correct in having the heat capacity of form II greater than that of form I. The nonmonotonic behavior of the experimental heat capacity differences reflects the experimental uncertainty (SI Section 1.8). An absolute heat capacity prediction for each method is given in Table 1.

The simplest method of estimating the atomic motions is approximating them as harmonic phonon frequencies. The harmonic approximation implies no thermal expansion, and so the structure is fixed and only *C*_v_ and Helmholtz Δ*A*_II→I_ free energies are calculated. The standard statistical thermodynamics methods can be used to calculate the thermodynamic properties at any temperature quite quickly from a set of phonon frequencies, though the summations include all the phonon modes including the acoustic modes, not just the vibrations of the unit cell (*q* = 0). The harmonic approximation uses the second derivatives of the lattice energy as it assumes that the potential energy wells around each atom are parabolic. Although conceptually simple, these calculations are challenging and vary in how well the modes which span more than the unit cell are treated (see SI Section 2.2). This treatment of the phonon dispersion (i.e., the *q* ≠ 0 modes that are not measured by IR or Raman) would be expected to be the main cause of the differences in the calculations that used the PBE-TS periodic density functional surface, except that the calculation of the SMZ phonons proved very sensitive to the convergence parameters used, requiring tighter convergence than the defaults in popular codes. Since van der Waals dispersion energy plays a major role in the structures of forms I and II, it is not surprising that there is sensitivity to the van der Waals dispersion correction. Indeed, changing the dispersion model to PBE-D4 resulted in imaginary frequencies for *q* ≠ 0 for form II (SI Section 2.6, Červinka). Imaginary frequencies can occur when the structure is not at a minimum in the potential energy surface, which can correspond to the structure being a dynamic average. Thus, the SMZ polymorphs proved to be a challenge to methods based on calculating harmonic phonons, probably because there are very small barriers between the local minima representing different structures. This is evidenced by the very low harmonic frequencies that were calculated, for example, AMS calculated that the lowest lattice mode is about 8 cm^–1^ for form I, and 12 cm^–1^ for form II. Such low frequency modes which involves motion between the layers in form I, may be quite anharmonic, but the AMS *TRHuST23* ^29^ corrections for various forms of anharmonicity, including methyl rotations, contributes only 0.44 kJ mol^–1^ at 298.15 K to the Helmholtz free energy difference between the polymorphs (SI Section 2.9, AMS) partially because of a cancellation between the different corrections. The harmonic approximation appears to incorrectly predict that the heat capacity of form I is higher than that of form II, but correctly approximates the temperature trend of Δ*C*_*p*_ (form II–I) (Figure 15). The exception, the harmonic model that gives the correct relative heat capacities, is the calculation using only the unit cell phonons on a potential surface (PBE-D4) and an interpolation procedure for the *q* ≠ 0 phonons (SI Section 2.3 Arhangelskis), and may be fortuitous.

One consequence of the molecular motions is the thermal expansion of the crystal, which is ignored in the harmonic approximation. The importance of the structure used for calculating the phonons is shown by the difference in the TCG-UNITO group calculations (SI Section 2.4) using the fully optimized structures and keeping the cell parameters fixed at the experimental values. The quasi-harmonic approximation does consider the thermal expansion, by optimizing the structure at a range of fixed cell volumes. This has been applied with the multimer embedding method ME3 for calculating the lattice energy and phonons as a function of temperature and pressure^27^ (SI Section 2.5, Boese), the Červinka group calculations on form I (SI Section 2.6), and using two recent machine-learned force fields to speed up these expensive calculations (SI Section 2.7, Lončarić). The results in Table 1 show that these calculations are sensitive to the model for the forces between the molecules, and the approximations made. The highly anisotropic thermal expansion of the SMZ polymorphs (Figure 9) provides a test of how well the model for the forces and the quasi-harmonic approximation are able to reproduce the anharmonicity of the molecular motions in SMZ crystals. Computational results of the composite quasi-harmonic approximation model by the Červinka group, listed in detail in Section 2.5 of the SI, agree with experiment that most of the thermal expansion of SMZ form I manifests in the direction of the lattice vector *c* where only weaker dispersion interactions act as the cohesive feature. On the other hand, equilibrium lengths of the lattice vector *a* exhibit only a minimum variation with respect to temperature as strong hydrogen bonds impede the crystal expansion in that direction. These findings generally align with the previously observed capability of the DFT-D based quasi-harmonic approximation to capture anisotropy of the thermal expansion of molecular crystals very well.^34^ Capturing the thermal expansion accurately can be important for predicting thermodynamic properties, as even in crystals of small molecules like imidazole, accounting for thermal expansion changes the Gibbs free energy by a few kJ mol^–1^ despite some cancellation of the effect on enthalpy and entropy.^35^ The effect of thermal expansion on the energy differences between polymorphs will depend on the differences in thermal expansion, which are quite marked for SMZ (Section 2.5.2).

Another approach that is based on the harmonic approximation, but uses the experimental structures and the experimental atomic displacement parameters (ADPs) is the NoMoRe approach^36–38^ which refines the calculated lowest energy phonon frequencies to model the ADPs. Using the 150 K crystal structures, the SMZ polymorphs proved unusually challenging for this approach, with the low-frequency modes being unusually correlated (SI Section 2.8, Hoser). However, the ADPs of forms I and II at 150 K are sufficiently similar that the effects of the NoMoRe refinement effectively canceled in the calculation of the thermodynamic differences. The transition temperature is very sensitive to the NoMoRe refinement, as this includes the entropy differences.

All phonon-based computational methods readily provide heat capacities, and it is worth noting that the absolute heat capacities are remarkably good (Table 1). Benchmarking the assembled electronic structure heat capacity values of SMZ form I (listed in Table 1) against the experimental value at 300 K reveals that the quasi-harmonic or anharmonic models provide a slightly better accuracy (RMSE of 8.5 J K^–1^ mol^–1^ or 2.9%) than the harmonic model (RMSE of 11.4 J K^–1^ mol^–1^ or 3.9%). That can be accepted as a fair computational accuracy, considering typical errors of heat capacities resulting from the quasi-harmonic DFT-D models for molecular crystals, and that the heat capacity is a response property, related to the second derivative of the Gibbs free energy.^28^ Nevertheless, this error would result in a significant error in terms of the thermal contribution to the enthalpy of a single polymorph. Such an additional computational uncertainty related to finite-temperature enthalpy hinders the current models in principle to reach the sub-kJ mol^–1^ accuracy. Since most methods resulted in underestimated heat capacities, one can deduce that the underlying computed phonon frequencies are predominantly somewhat overestimated.^39^ Capturing the positive thermal expansion within the quasi/anharmonic models increases the *Cp* which in turn improves the computational accuracy. As the temperature increases, the thermal expansion becomes even more pronounced, possibly leading to stark *Cp* increases at elevated temperatures. However, the quasi-harmonic approximation itself may fail at too high temperatures in the vicinity of the melting point as it is difficult to describe the strongly anharmonic excited (i.e., large-amplitude) vibrations at such premelting conditions.^34^

The phonon approach is making assumptions about the types of molecular motions in the crystal, and so in principle, modeling their actual motions in an MD simulation should be better for the same potential energy surface. Molecular Dynamics allows the simulation of the motions of the molecules within a supercell at different temperatures, and so these calculations do not restrict the motions to being harmonic or quasi-harmonic. However, reproducing the motions over sufficient time in a large enough supercell requires the rapid calculation of the forces between the atoms, and so is done using an atomistic model (force field). The SMZ polymorphs proved to be very sensitive to the force field used, and indeed, one commonly used force field needed to be reparametrized to improve the modeling of the sulfonamide torsion angles (SI Section 2.10, CB@Lisbon). Extracting the properties by statistical sampling of a sufficiently long Molecular Dynamics trajectory to have covered the range of configurations that can be adopted within the crystal (SI Section 2.10, CB@Lisbon) is demanding. Two methods that have been developed to calculate free energies more efficiently from the MD simulations have been tested for SMZ, the PGMCrys + MBAR approach^32,33^ by MME@UCL and the pseudosupercritical path method (PSCP)^31^ approach of XtalPi. The MD simulations are performed and analyzed at different temperatures, and so the calculation of heat capacities represented a new challenge.

Molecular Dynamics calculations allow the visualization of the molecular motions. The MME@UCL group analyzed their 10 ns simulations of 24 molecule supercells (SI Section 2.12) and saw that the methyl groups were rotating in both forms and the NH_2_ groups in form II at even the lowest temperature of –73 °C. By room temperature, the NH_2_ groups were rotating in form I. Distinct larger motions were observed nearing the experimental transition temperature, with rotation of the aniline groups in form II, consistent with the ^13^C solid-state NMR spectrum (Section 2.3) and sliding of the layers in form I. Very similar effects were seen in another MD study (SI Section 2.10, CB@Lisbon). The time scales and box size in these simulations would have made observation of the phase transition very unlikely, particularly as it is observed to initiate at defects (Section 2.6.1). However, the consistency between these simulations with different force fields, one of which was tested and gave reasonable melting points (SI Section 2.10, CB@Lisbon), supports the realism of these short time scale motions.

One limitation of Molecular Dynamics that models the classical motions of the molecules is that it neglects the zero-point energy, which is dominated by the higher frequency modes. The FT-IR spectra (Figure 4) show a significant difference in the highest frequency modes, representing the hydrogen bonding differences, with form I having higher frequencies, implying stronger hydrogen bonding, than form II. The phonon-based estimates of the zero-point energies show that form II has a higher zero-point energy than form I by 2 to 3.6 kJ mol^–1^ (SI Section 2.13), making a significant contribution to the calculated enthalpy differences in Table 1 from all phonon methods. The sign also implies that the contribution from the hydrogen bonding modes is not dominant. It is also worth noting that zero-point motion has been estimated to increase the molecular volume of the crystalline imidazole by 2%^35^ and crystalline ammonia by 3%^40^ and only the quasi-harmonic calculations include the effect of zero-point motion on cell size.

Overall, no method is in perfect agreement with experiment. All methods appear to overestimate the enthalpy difference, with the calculations using theoretically better models for the forces between the molecules generally being in better agreement with experiment. The CSP workhorse method, PBE-TS, generally overestimates the lattice energies^41^ and the errors do not appear to cancel well for sulfamerazine polymorphic energy differences (Table 1). This is partially due to the functional, but the dispersion model also makes a considerable difference. The Boese tight method gives the best difference in heat capacities, which calculates *C*_*p*_ and accounts for thermal expansion.

## 4 Discussion

This paper illustrates the distinction between the true thermodynamic differences between polymorphs, such as enthalpy or the transition temperature at which the Gibbs free energies are equal, and what we can measure or calculate. The difference depends on how the real system differs from our idealized models, both in the computational approximations, and in experiment, particularly because the SMZ form II to I solid-state transition occurs by nucleation and growth, with kinetic barriers leading to hysteresis and practical irreversibility. By comparing and contrasting different experiments and calculations, we may hope to approximate the true objective reality more closely than through any individual physical or computational experiment. The approximations in current computational models have been outlined in Section 3, so here we discuss some of the limitations of the experiments, including the questions posed by the discovery of form V, before considering the challenges that the experimental work poses for computation in the specific case of sulfamerazine.

### 4.1 Phase Diagram of Sulfamerazine

There is no doubt that there is an enantiotropic relationship between forms I and II of SMZ, with form II (*Z*′ = 1) being the low-temperature stable form. Crystalline form II undergoes a solid–solid phase transition to form I on heating above around 150 °C, with a benchmark value of Δ_trs_ *H*_m_(II → I) = 3.15 ± 0.12 kJ mol^–1^ at 150 °C from DSC measurements corrected by heat capacities for the increase in transition temperature with heating rate (Figure 12). The shift in transition temperature with heating rate corresponds to an enthalpy change of 0.55 kJ mol^–1^. This is consistent with the transition being a first-order phase transition, starting from defects as observed in the hot-stage microscopy (Figure 11), which, like the variable-temperature PXRD (Figure 10), shows a transition at approximately 160 °C. This transition is not reversible (when cooled from 275 to 25 °C via DSC, but some degradation may have occurred before the cooling cycle). However, the temperature for a solid-to-solid transformation is often higher than the thermodynamic transition temperature because of the activation energy barrier, the need for the molecules to have sufficient energy to rearrange between the crystal structures. Indeed, some enantiotropic polymorph pairs do not show a transformation in a practical heating experiment, and as the reaction is first order, any measured transition rate can be dependent on crystal size, crystallinity, history of the sample, as well as heating rate. The large difference between the estimated solution and solid-state transition temperatures for forms II to I transition of SMZ is in line with those seen in 4′-hydroxyacetophenone of ∼70 K^43^ and 4-hydroxybenzaldehyde of 46–57 K.^44^

The BEST-CSP collaboration of providing DSC measurements of the enthalpy difference between different laboratories emphasizes how the different experimental protocols (SI Section 1.6) give a variability in results that is intrinsic to measurements of a first-order solid-state phase transformation. It should be noted that the variations in the experimental enthalpy difference estimates are insignificant compared with the variation in the computational estimates (Table 1). Indeed, the degree of agreement between the laboratories working with different powder samples for form II, can be seen as reassuringly good once it is accepted that it is impossible to observe this first-order solid-state transition at the thermodynamic transition temperature. Thus, the SMZ study contributes to the BEST-CSP network aim to use the DSC data on a variety of systems from a range of groups to establish the best protocol (sample preparation, heating rate, etc.) and the intrinsic uncertainty in experimentally determining the transition enthalpy for enantiotropic systems with an observable transition. As the transition is observable, other thermal methods of assessing the thermodynamic stability of the polymorphs, such as measuring the enthalpy of sublimation, are complicated by the risk of transitioning to the high-temperature form (e.g^42^). On the other hand, solution calorimetry could provide an isothermal enthalpy difference, but these experiments are time-consuming and need care with the possibility of phase transformations.

It is significant that no signs were seen of a solid-state transformation from form II to form V or of form I transforming to any other structure on heating, independent of the crystal size of the samples used.

The project anticipated finding the thermodynamic transition temperature from the solvent-mediated transition, but this was frustrated by the slurrying experiments above 50 °C, giving form V, not form I. Form II is the stable form below 50 °C, but it appears that above this temperature form V is the most stable. The usual assumption is that the transition occurs when the Gibbs free energies of the two polymorphs are equal, though for the slurrying and solubility experiments, it is strictly the Gibbs free energies of the polymorphs in the presence of the solvent. If it is assumed that the solutions are equivalent, and there are no other phases, such as solvates involved, then the solvent only affects the kinetics, with the presence of the MeCN solvent facilitating the nucleation and growth of form II. However, the observation of form V in experiments where there has been sufficient time to reach equilibrium suggests that it is more stable than form I, at least in the temperature range 50–60 °C which we investigated for solvent-mediated transformations. An extended extrapolation of the solubilities measured by clear-point of forms I and V in Figure 13 would give a transition to form I as the most stable form at ∼85 °C.

The lack of an observed solid-state transition between forms V and I reflects the lack of a plausible mechanism. The layers are the same, but converting between form I and V requires an approximate flipping of the entire layer (or a significant rearrangement of all the molecules within the layer, Figure 2), which is unlikely to happen in the solid state. There is also no thermodynamic driving force for the transformation to go by nucleation and growth, as experimentally demonstrated by the similarities in the solubilities and melting points (Section 4.2). The structures are so similar, differing only in the dispersion-dominated stacking of the layers that it is not surprising that the computational estimates for the lattice energy difference between form I and V are within ± 0.5 kJ mol^–1^, well within the uncertainty in any method (SI Section 2.14).

It is noticeable that the layers in forms V and I are also seen in form III and four solvate structures. It appears that this flat layer is readily formed, and stacked to give the kinetically favored form I, and that the thermodynamically more stable form II and V require slow crystallization conditions with the opportunity for thermodynamic equilibration. The difference in the solubilities is so small that we cannot confidently determine whether forms I and V are monotropically or enantiotropically related. Further, equilibrium solubility studies using a different approach than the clear-point solubility measurement could provide further insight into the relationship of the forms I and V, provided that equilibrium is reached between unambiguously defined phases (including possible solvates or solid solutions) prior to solvent-mediated phase transformation. This makes these measurements tricky^45^ and can also affect the measured solubilities. Performing such solubility studies could be particularly difficult for SMZ as the *TRHuST23* predicted temperature dependence of the relative Helmholtz free energies of all the SMZ polymorphs (SI Section 2.9, AMS) suggests that both form III and IV could be the most stable at certain temperatures (though all the relative energies are within estimated computational uncertainty around the II to I transition). Similarly, the PGMcrys method (SI Section 2.12, MME@UCL), also predicts the stabilization of form III and destabilization of form IV with temperature. (The error bars in the PGMcrys are those of the sampling statistics and do not including any estimate of the accuracy of the force field.)

Hence, we can only provide part of the phase diagram of SMZ, establishing that II is enantiotropically related to both forms I and V. The thermodynamic transition temperature between II and V was established by slurrying as approximately 50 °C, but the kinetic hindrance of the phase transition between II and I intrinsically limits the measurability and accuracy of its enthalpy and temperature.

### 4.2 Is Form V a Late-Appearing or Undetected Polymorph?

The structural similarity between form I and V is so great that the difference in any physical properties must be small. This is certainly true of the spectra—only high-quality PXRD patterns are able to distinguish between the two forms, with the FTIR and ss-NMR spectra being almost identical. The melting temperatures of the two forms as measured in triplicate by DSC in high-pressure pans and a heating rate of 20 K min^–1^ at the University of Innsbruck is 236.9 ± 0.35 °C for form I and 236.8 ± 0.37 °C for form V, i.e., the same within experimental uncertainty. This similarity suggests that the form II to form I transition is probably close to the form II to form V transition, i.e., around 50 °C.

The PXRD pattern of a commercial sample (Toku-e, Figure 16) shows some shoulders on the low-angle peaks, which are indicative of the presence of form V. Rietveld analysis of this commercial sample, estimates that there is approximately 20% of form V (SI Section 1.9). A 50 year-old commercial form I sample (Bayer) does not show physical impurity, at least within the detection limits of PXRD, indicating that form I has a very high kinetic stability at room temperature. The question arises as to whether experiments done when only form I and II were known would have detected the presence of form V. The diffractograms of the commercial form I starting material in the 1998 study^46^ do show signs of form V in the 2θ range of 15–20°, but these features appear similar to those of form II in the same range, and hence the presence of a new polymorph (form V) was unsurprisingly not recognized. We note that form V was found independently by three laboratories during this BEST-CSP project. Form V can be produced from phase pure form I (Innsbruck Bayer form I), so the appearance of form V seems to not be linked to the chemical impurity profile. The identification of form V is probably a result of better instrumentation, benefiting from confirmation in a multilaboratory investigation.

**Figure 16:**
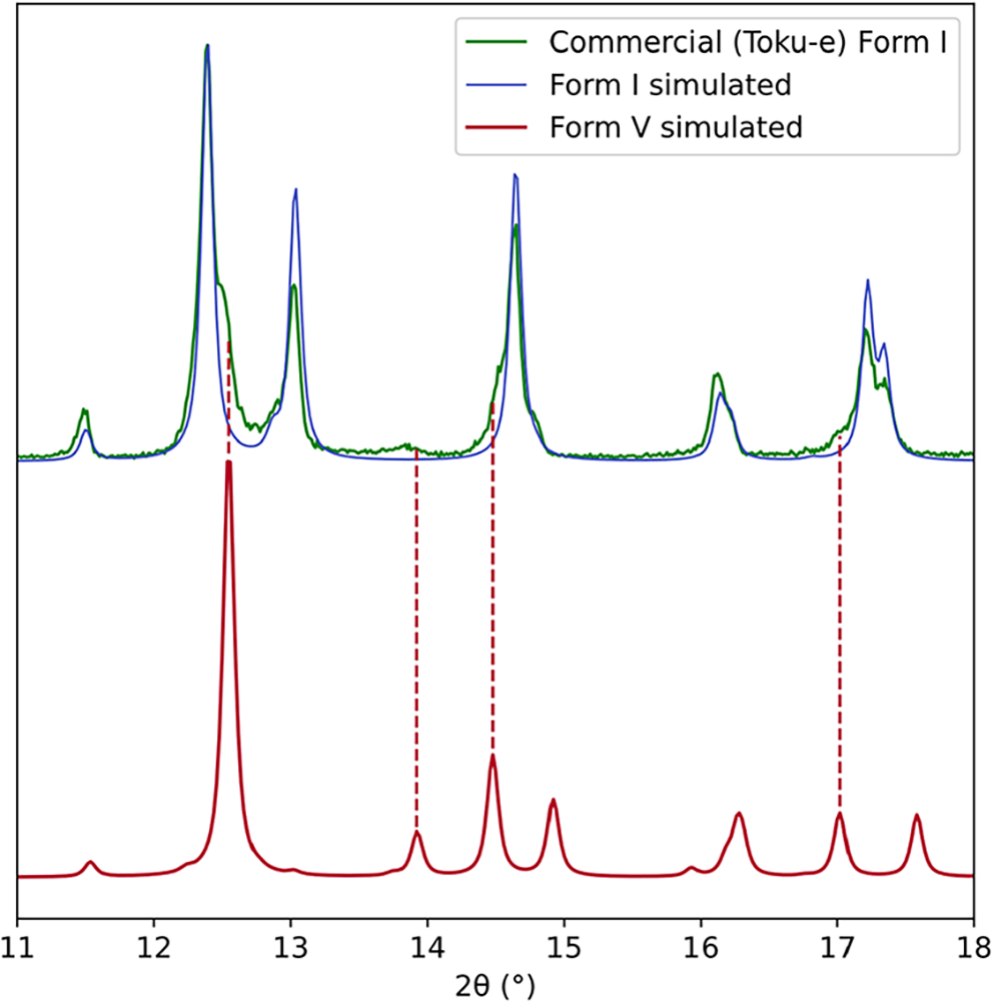
Section of the PXRD (Cu K-α_1_) diffractogram (RT) of the commercial material (Toku-e, as received) in green overlaid with the simulated diffractogram of form I (RT) in blue. The simulated diffractogram of form V (150 K) is at the bottom in red with lines to show the peaks of form V present in the commercial material.

Currently, we have established a reproducible recipe for form II by slurrying in MeOH/H_2_O (80:20, v/v) at room temperature until well-equilibrated. A well-equilibrated slurry above 50 °C produces form V. Phase pure form I can be obtained by heating form II.

We deem form V to be a polymorph as it has been reproduced in four laboratories (Innsbruck, UCL, Radboud, Jagiellonian) by different means, including sublimation (SI section 1.2.2). It has been well characterized by both PXRD and SCXRD, and is more stable than form I above 50 °C.

However, the close relationship between form I and V is typical of polytypism. Indeed, form III could be viewed as another polytype, if only the single layer is considered (Figure 2) as a compound is considered polytypic if it occurs in several structural modifications, each differing only in the stacking sequence. It is possible that many other polytypes might form, with a range of stacking sequences.^12^ Indeed, the situation could be similar to racemic tazofelone, where three polymorphs have been identified with different stackings of the layer,^47^ but the single frame X-ray diffraction images show stacking disorder and there is a variation in single crystal melting points and enthalpies. The problem of closely related structures is also shown by olanzapine forms II and III, which have not yet been produced in phase pure microcrystalline samples.^48^ The structure of form III has only recently been determined by electron diffraction on a microcrystal within the powder sample.^49^ An even more difficult to characterize type of polytypism is shown by aspirin^50^ where the two polytypic polymorphs have been found within the same crystal. The kinetically favored form of SMZ is clearly form I as it can be obtained from a variety of experiments, but it is likely that some samples will contain an amount of form V and possibly other polytypes or polymorphs such as form III.

### 4.3 Challenge to Computation

The BEST-CSP experimental study has not only provided an understanding of the enthalpy difference between SMZ form I and II and its variation with temperature, but also, unusually experimental data on heat capacity differences, thermal expansion and spectral data, which can be used to test the intermediate results generated by some computational methods. This is particularly important as the data in Table 1 shows that the most established methods, periodic dispersion corrected density functional theory (e.g., PBE-TS) and harmonic approximation, are not performing well for SMZ. This is linked to SMZ being a flexible molecule, with the sulfonamide group being a particular challenge, and that its polymorphs are predominantly bound by van der Waals dispersion forces, with some polymorphs having weak slip planes. In this paper, we have results for many of the more realistic methods that are under active development by the participating groups, and the SI Section 2 gives some of the lessons learnt that will facilitate the development of methods.

The compromise between the accuracy of the model for the forces between the atoms and the assumptions that need to be made in modeling the thermodynamics are very apparent in Table 1. In the context that until recently, electronic structure methods were aiming for chemical accuracy of 1 kcal mol^–1^ (4.2 kJ mol^–1^), the results are very encouraging. However, far greater accuracy is required for studying organic polymorphs, where the energy differences are usually smaller. The transition temperatures are very sensitive to the differences between the structures. It may be hoped that the current emphasis on developing better force fields will help bridge the gap between phonon and MD-based methods. Indeed, the Lončarić group are considering using the MACE@SPICE2 force field in MD methods. However, in addition to the model for forces, and assumptions in the thermodynamic modeling, the calculations also rely on various technical parameters, and it is worth noting that the comparison of forms I and II of SMZ has the unusual advantage that they have the same number of molecules in the unit cell.

Similarly, the computational estimates of the absolute heat capacities of crystalline SMZ appear to be good by many methods. However, it is the balance between the differences in the heat capacities and the difference in the lattice energies and zero-point energies that determines whether there is an enantiotropic relationship between the polymorphs. This appears to be much more difficult to model computationally, at least for SMZ which has very anisotropic thermal expansion, and signs of large phenyl ring librations in form II. A relatively large lattice energy difference between SMZ polymorphs requires a large difference in heat capacities, which could make the challenge of modeling molecular motions particularly difficult. Hence the extension of the quasi-harmonic model to include anharmonicity through quasi-particle theory^51^ may prove to be appropriate for sulfamerazine.

## 5 Conclusion

A COST Action network, BEST-CSP, has performed a multidisciplinary study on the kinetically favored, readily formed polymorph I and low-temperature stable polymorph II of sulfamerazine, a typical sulfonamide drug. A reliable recipe for producing form II has allowed multiple studies of the solid-state transformation of form II to form I on heating at around 150 to 175 °C. As this is an enantiotropic but practically irreversible transformation that proceeds by nucleation and growth, the solid-state transition temperature is higher than the thermodynamic transition temperature due to the need to overcome an activation barrier. This leads to a variation in the measured transition enthalpy, of order 0.5 in 3 kJ mol^–1^ in the range of the transition temperatures measured by DSC. The solution phase transformation between form II and I was reported once in the literature^2^ by slurrying at 51–54 °C but was not observed in this work, however, solubility measurements suggest a form II to I transition around 51 °C (Section 2.6.2). As is necessary for an enantiotropic phase transition, there is a difference in the heat capacities of the two forms, reflecting differences in the motions of the molecules. This is reflected in the different anisotropic thermal expansion, with the ADPs, ss-NMR and Molecular Dynamics simulations suggesting that there are differences in some large amplitude motions of the rings, that need not be directly related to the phase transition mechanism. This makes computing the thermodynamic properties of these enantiotropic polymorphs a challenge, and this has been attempted using a range of types of calculations, from molecular dynamics to (quasi-) harmonic lattice dynamics. The experimental heat capacity and thermal expansion measurements provide valuable tests of the approximations in various methods, as well as the dependence on the model for the forces between the atoms. Sulfamerazine proves to be a challenging case for modeling polymorph transition thermodynamics, with generally better results being obtained by the less approximate methods.

A new polymorph, form V, that is similar to form I, was identified as the most stable form between 50 and 70 °C by a combination of slurrying and solubility measurements. Slurries confirmed form II is the most stable form in the range 10–48 °C and is enantiotropically related to both form I and form V with form V becoming the most stable form at 48–50 °C. The solubility data indicates form V is more stable than form I over the range of temperatures investigated (10–70 °C). No form V to I transition has been observed in solution up to 60 °C or the solid state but the form V to I transition may be so kinetically hindered that it is impossible to observe in the solid state. Forms I and V are very similar in that they contain the same double layer of hydrogen-bonded molecules, with the single layer also being found in form III and some solvates. The new form V and form I are almost identical for a 27 molecule cluster, but differ in the third layer stacking (Figure 2). The differences between forms I and V are only apparent in high-quality powder diffractograms, and so form V may have gone undetected as a phase impurity in form I in past studies. At least one commercial sulfamerazine form I sample contains substantial amounts of form V. The finding of form V increased the complexity of the sulfamerazine solid-state landscape and limits what we have been able to determine about the form II to I phase transition. Further work on the system should also consider forms III and IV.

## Supplementary Material



## Data Availability

The raw experimental data is available from the BEST-CSP depository: https://github.com/ccdc-opensource/collaboration-bestcsp-experimental-data.

## Notes

Experimental details including single-crystal structure determinations, variable temperature PXRD measurements, competitive slurries, ^13^C ss-NMR, DSC, consensus estimate method and heat capacity measurements. Additional computational methodology from each contribution group and zero-point energies of the polymorphs and the differences in the lattice energies of form I and V (PDF)
