# Supplementary material for: BEST-CSP Benchmark Study of Polymorphs I and II of Sulfamerazine and the Perils of Polytype Polymorphs
Source: Cryst Growth Des. 2025 Dec 12;26(1):476–93. doi: 10.1021/acs.cgd.5c01406 (PMC12784330; doi:10.1021/acs.cgd.5c01406)
Supplement: Supplementary file 1 [file cg5c01406_si_001.pdf]

Supporting information for BEST-CSP benchmark study of polymorphs I and II of sulfamerazine and the perils of polytype polymorphs

William P. Wood[a], Mihails Arhangelskis[b], Erika Bartůňková[c], Carlos E. S. Bernardes[d], A. Daniel Boese[e], Doris E. Braun[f,g], Dejan-Krešimir Bučar[a], Helena Butkiewicz[b], Ctirad Červinka[h], Bartolomeo Civalleri[i], Nicolas Couvrat[j], Erik de Ronde[k], Lorenzo Donà[i], Martin Dračínský[c], Dzmitry Firaha[l], Michal Fulem[h], Reynaldo Geronia II[h], Natalia Goncharova[e], Marlena Gryl[m], Johannes Hoja[e], Anna Hoser[b], Joanna Krzeszczakowska[b], Alexander List[e], Ivor Lončarić[n], Bruno Mladineo[n], Jonas Nyman[o], Edgar Olehovics[p], Mattia Raimondo[i], Ivo B. Rietveld[j], Rute I. S. Rodrigues[d], Luca Russo[q], Matteo Salvalaglio[p], Mafalda Sarraguça[r], Jiří Šnajdr[h], Vojtěch Štejfa[h], Guangxu Sun[s], Paul Tinnemans[k], Pamela S. Whitfield[t], Zhuocen Yang[s], Yizu Zhang[s], Sarah L. Price[a]

[a] Department of Chemistry, University College London, 20 Gordon St, London WC1H 0AJ, UK

[b] Faculty of Chemistry, University of Warsaw, Pasteura 1, 02-093 Warsaw, Poland

[c] Institute of Organic Chemistry and Biochemistry, Czech Academy of Sciences, Prague 6 160 00, Czech Republic

[d] Centro de Química Estrutural, Institute of Molecular Sciences, Departamento de Química e Bioquímica, Faculdade de Ciências, Universidade de Lisboa, 1749-016, Lisboa, Portugal

[e] Department of Chemistry, University of Graz, Heinrichstrassse 28, Graz, Austria

[f] University of Innsbruck, Institute of Pharmacy, Pharmaceutical Technology, Josef-Moeller-Haus, Innrain 52c, A-6020 Innsbruck, Austria

[g] Christian Doppler Laboratory for Advanced Crystal Engineering Strategies in Drug Development, Institute of Pharmacy, University of Innsbruck, 6020 Innsbruck, Austria

[h] Department of Physical Chemistry, University of Chemistry and Technology, Prague, Technická 5, CZ-166 28 Prague 6, Czech Republic

[i] Department of Chemistry, Via Pietro Giuria 7, 10125, Turin, Italy

[j] University of Rouen Normandy, Normandy University, SMS laboratory (UR 3233), 76000 Rouen, France

[k] Radboud University Nijmegen, Institute for Molecules & Materials, Department of Solid State Chemistry, Heyendaalseweg 135, 6525 AJ Nijmegen, The Netherlands

[l] Avant-garde Materials Simulation, Alte Str. 2, Merzhausen, Germany

[m] Jagiellonian University, Faculty of Chemistry, Gronostajowa 2, 30-387 Krakow, Poland

[n] Ruđer Bošković Institute, Bijenička Cesta 54, 10000, Zagreb, Croatia

[o] The Cambridge Crystallographic Data Centre, 12 Union Road, Cambridge CB2 1EZ, UK

[p] Department of Chemical Engineering, University College London, London WC1E 7JE, United Kingdom

[q] GSK Medicines Research Centre, Gunnels Wood Road, Stevenage, Hertfordshire, SG1 2NY, UK

[r] LAQV, REQUIMTE, Departamento de Ciências Químicas, Faculdade de Farmácia, Universidade do Porto, Rua de Jorge Viterbo Ferreira, 228, 4050-313 Porto, Portugal

[s] XtalPi Inc (Shenzhen Jingtai Technology Co., Ltd.), International Biomedical Industrial Park (Phase II), 3F, 2 Hongliu Rd, Futian District, Shenzhen, China, 518038

[t] Excelsus Structural Solutions, Parkstrasse 1, 5234 Villigen, Switzerland

Table of Contents

1. Experimental Details 5
   1.1. Crystal Structures 5
   1.2. Single-crystal X-ray diffraction 6
      1.2.1. Form II 15
      1.2.2. Form V 15
      1.2.3. Acetone solvate structure 18
   1.3. Variable temperature Powder X-ray diffraction. 19
   1.4. Additional Competitive Slurry and Grinding Experiments 23
   1.5. Solid-state NMR spectra 26
   1.6. Differential Scanning Calorimetry (DSC) 28
      1.6.1. UCL/Innsbruck 29
      1.6.2. UCT Prague 30
      1.6.3. Radboud University 30
   1.7. Method of consensus estimate for enthalpy difference between forms I and II from DSC data 31
      1.7.1. Introduction 31
      1.7.2. The Random Effects Model 32
      1.7.3. Error estimate and propagation for enthalpy difference at STP 34
   1.8. Heat capacity measurements 35
   1.9. Evaluations of Sample Purity 39
   1.10. Solubility by Clear Point Measurements 41
2. Computational Details 43
   2.1. Bondlength variation in calculations. 44
   2.2. Harmonic mode calculations 48
   2.3. Arhangelskis Group 51
   2.4. TCG-UNITO Crystal phonon calculations 56
      2.4.1. Methodology 57
      2.4.2. Computational Details 57
      2.4.3. A few comments 58
   2.5. Boese group quasi-harmonic multimer imbedding 59
      2.5.1. Results 62
   2.6. Cervinka group quasi- harmonic combined method periodic calculations. 62

2.7. Loncaric group quasi-harmonic calculations with universal machine learning potentials 66
2.8. Hoser group: Normal Mode Refinement of frequencies 68
2.8.1. NoMoRe 68
2.8.2. Computational details 69
2.8.3. Lattice energy 70
2.8.4. Heat Capacity Estimation 70
2.8.5. Thermodynamics from NoMoRe 72
2.8.6. Calculation of ΔH for the II → I Phase Transition 75
2.8.7. Insight into the frequencies and ADPs 76
2.8.8. Comment on Refined first eight vibrational modes 77
2.8.9. Conclusion on NoMoRe refinement 79
2.9. AMS: Free energy calculations with TRHuST 82
2.9.1. Imaginary mode correction 82
2.9.2. Very soft mode correction 82
2.9.3. Methyl top correction 82
2.9.4. Hydrogen-bond correction 83
2.9.5. Large cell correction 83
2.9.6. Sulfamerazine Forms: Free Energies with the TRHuST 23 Method 84
2.10. CB@Lisbon Molecular Dynamics Simulations 85
2.11. XtalPi: pseudo-supercritical path method (PSCP) 103
2.12. MME@UCL – PGM + MBAR 106
2.12.1. Helmholtz to Gibbs conversion 106
2.12.2. Gibbs FE differences as a function of temperature with MBAR 108
2.12.3. Molecular Dynamics Simulations Setup 109
2.12.4. Supplementary Results 110
2.13. Zero-point energies 115
2.14. Lattice energy differences of forms I and V 115

1. Experimental Details

1.1. Crystal Structures

Table 1.1.1. List of SMZ crystal structures

Form	T / K	Space Group	Z'	R-factor / %	a / Å	b / Å	c / Å	β / °	Refcode/ notes
	150			3.83	14.4646	8.1812	21.7718		This work, UCL
	150			4.33	14.4588	8.1800	21.7664		
	160			4.40	14.4597	8.1802	21.7812		
	170			4.43	14.4638	8.1835	21.7953		
	180			4.44	14.4666	8.1868	21.8103		
	190			4.81	14.4701	8.1889	21.8216		
	200			4.51	14.4734	8.1921	21.8397		
	210			5.05	14.4763	8.1946	21.8522		
	220			4.53	14.4795	8.1974	21.8693		Pallipurath et al[1]
	230			4.63	14.4814	8.1993	21.8844		
	240			4.73	14.4852	8.2020	21.9002		
I	250	$Pna2_1$	2	4.77	14.4884	8.2048	21.9167	90	
	260			4.76	14.4914	8.2069	21.9332		
	270			4.87	14.4950	8.2094	21.9489		
	280			4.93	14.4973	8.2118	21.9658		
	290			5.01	14.5008	8.2141	21.9821		
	RT	$Pca2_1$		-	14.65	8.10	22.12		SLFNMA[2] No coordinates
	RT	$Pn2_1a$		4.7	14.474	21.953	8.203		SLFNMA02[3]
	RT			3.14	14.5018	8.2173	21.9870		This work, UCL
	150	$Pna2_1$		3.99	14.477	8.187	21.798		SLFNMA04[4]
	300			4.98	14.5040	8.2162	21.9984		Pallipurath et al[1]
	413			5.59	14.5464	8.2462	22.2397		This work, UCL
	150			2.88	9.0904	11.5429	22.8616		This work, UCL
	150			4.40	9.0823	11.5422	22.8439		
	160			4.41	9.0881	11.5538	22.8478		
	170			5.86	9.0992	11.5635	22.8525		
	180			4.58	9.0972	11.5751	22.8531		
	185			5.78	9.1048	11.5802	22.8576		
	190			5.67	9.1064	11.5845	22.8569		
	195			6.13	9.1070	11.5902	22.8593		
	200			4.78	9.1066	11.5983	22.8627		
	220			4.59	9.1158	11.6222	22.8715		Pallipurath et al[1]
II	230	*Pbca*	1	5.45	9.1200	11.6339	22.8697	90	
	240			4.82	9.1237	11.6442	22.8738		
	250			5.38	9.1280	11.6579	22.8759		
	260			5.06	9.1311	11.6667	22.8800		
	270			5.86	9.1357	11.6782	22.8834		
	280			6.05	9.1405	11.6923	22.8838		
	290			6.80	9.1415	11.7002	22.8837		
	RT			7.8	9.145	11.704	22.884		SLFNMA01[5]
	RT			4.77	9.101	11.549	22.874		SLFNMA05[6]
	RT			2.93	9.1370	11.7026	22.8620		This work, UCL
	300			6.35	9.1445	11.7130	22.8862		Pallipurath et al[1]
	413			3.61	9.1954	11.8656	22.9266		This work, UCL
III	150	$P2_1/c$	1	5.34	11.097	8.315	13.964	99.33	SLFNMA03[7]
IV	RT	$P2_1/c$	1	4.65	12.571	6.367	16.175	110.22	SLFNMA06[6]
	100			5.73	22.6961	8.16100	14.4100	106.548	This work, Jagiellonian
V	150	$P2_1/c$	2	3.75	22.7355	8.18664	14.45767	106.677	This work, UCL
				5.37	22.7116	8.1883	14.4536	106.631	This work, Radboud
	RT			-	23.257	8.201	14.448	109.34	This work, Innsbruck XRPD

References

[1] Pallipurath, A. R.; Skelton, J. M.; Warren, M. R.; Kamali, N.; McArdle, P.; Erxleben, A. Sulfamerazine: Understanding the Influence of Slip Planes in the Polymorphic Phase Transformation through X-Ray Crystallographic Studies and ab Initio Lattice Dynamics. *Molecular Pharmaceutics* **2015**, *12* (10), 3735-3748, Article. DOI: 10.1021/acs.molpharmaceut.5b00504.

[2] Deo, N.; Tiwari, R. K.; Singh, T. P. Crystallization and x-ray crystal data of sulfonamides. *J. Sci. Res* **1980**, *2* (2), 137.

[3] Caira, M. R.; Mohamed, R. Positive identification of two orthorhombic polymorphs of sulfamerazine (C11H12N4O2S), their thermal analyses and structural comparison. *Acta crystallographica. Section B, Structural science* **1992**, *48* (4), 492-498.

[4] Elsegood, M. R. J.; Mumtaz, A.; Weaver, G. W.; Mahmud, T. CCDC 1051902: Experimental Crystal Structure Determination. *CSD Communication* **2015**. DOI: 10.5517/cc149lbc.

[5] Ravindra Acharya, K.; Kuchela, K. N.; Kartha, G. Crystal structure of sulfamerazine. *Journal of Crystallographic and Spectroscopic Research* **1982**, *12* (4), 369-376. DOI: 10.1007/BF01159053.

[6] Direm, A.; Parlak, C.; El Bali, B.; Abdelbaky, M. S. M.; García-Granda, S. Experimental and computational insights into polymorphism in an antimicrobial sulfadrug: discovery of a novel monoclinic form of sulfamerazine. *Journal of the Iranian Chemical Society* **2024**, *21* (11), 2799-2815, Article. DOI: 10.1007/s13738-024-03110-x.

[7] Hossain, G. A new polymorph of sulfamerazine. *Acta Crystallographica Section E* **2006**, *62* (6), o2166-o2167. DOI: 10.1107/S1600536806014449.

1.2. Single-crystal X-ray diffraction

Contributed by Dejan-Krešimir Bučar

UCL: The diffraction data for all crystal structures were collected on a four-circle *Agilent SuperNova* (Dual Source) single crystal X-ray diffractometer using a micro-focus CuK_α X-ray beam (λ = 1.54184 Å) and a *HyPix-Arc 100* curved hybrid photon counting X-ray detector. The sample temperature was controlled with an *Oxford Instruments* cryojet. The temperature of the nitrogen atmosphere surrounding the single crystals was measured using a *Hanna Instruments HI 93532* thermocouple before and after the collection of each dataset.

The data collection strategies for Forms I and II were calculated according to the Laue symmetry of the crystals to reduce data collection times at high temperatures and thereby

minimise the decomposition of the single crystals. Data collections based on the Laue symmetry were then also pursued at lower temperatures to allow for a comparison of atomic displacement parameters at different temperatures in a consistent manner. Single crystals with cuboid morphologies were used to minimise the effects of anisotropic X-ray absorption on the determination of the atomic displacement parameters. And when possible, the variable temperature studies were pursued using the same single crystal.

The data were processed using the *CrysAlis^Pro^* software from *Rigaku Oxford Diffraction*.[1] All crystal structures were solved with the *SHELXT* program,[2] used within the *Olex2* program,[3] and refined by least squares on the basis of F^2 with the *SHELXL* program,[4] used within the *ShelXle* graphical user interface.[5] All non-hydrogen atoms were refined anisotropically by the full-matrix least-squares method. Hydrogen atoms associated with carbon and nitrogen were refined isotropically in geometrically constrained positions [$U_{iso}(H_C) = 1.2 \cdot U_{eq}(C)$, $U_{iso}(H_N) = 1.5 \cdot U_{eq}(N)$].

The N−H distances of the amine groups in all structures were restrained using the DFIX command in *SHELXL*. The geometry of the acetone molecule in the sulfamerazine acetone solvate was modelled using the EADP, ISOR, and SADI commands.

The asymmetric units of all crystal structures are shown in Figures **1.2.1-1.2.8**. Crystallographic and refinement parameters for all crystal structures are given in Tables **1.2.1-1.2.3**.

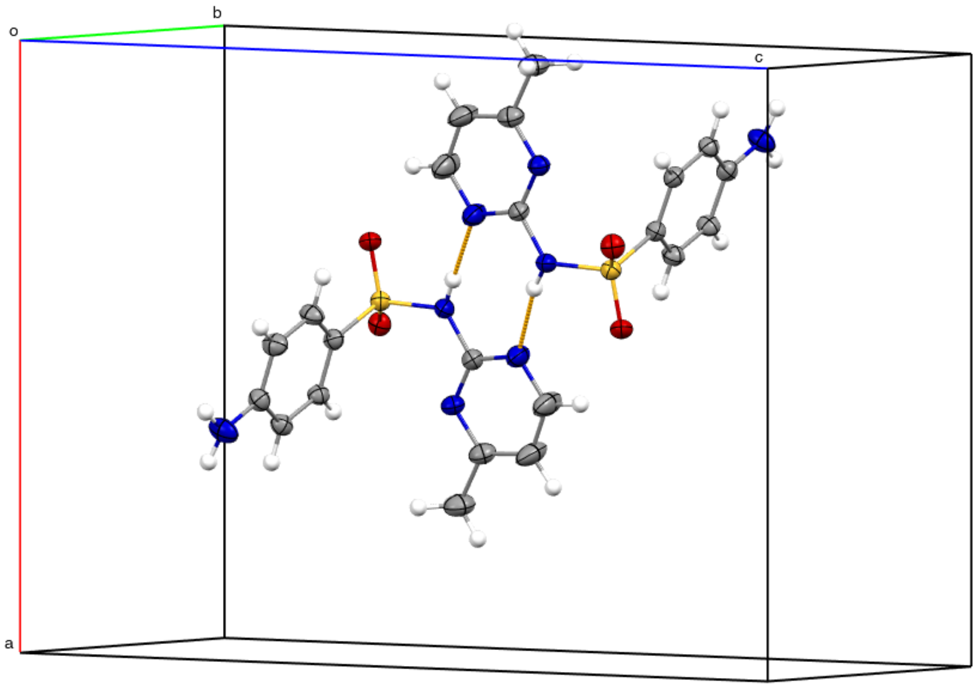

Figure 1.2.1. The asymmetric unit of sulfamerazine Form I collected at 150 K. The thermal ellipsoids are drawn at the 50% probability level. Colour scheme: carbon – dark grey, nitrogen – blue, oxygen – red, sulfur – yellow, hydrogen – white, hydrogen bonds – orange.

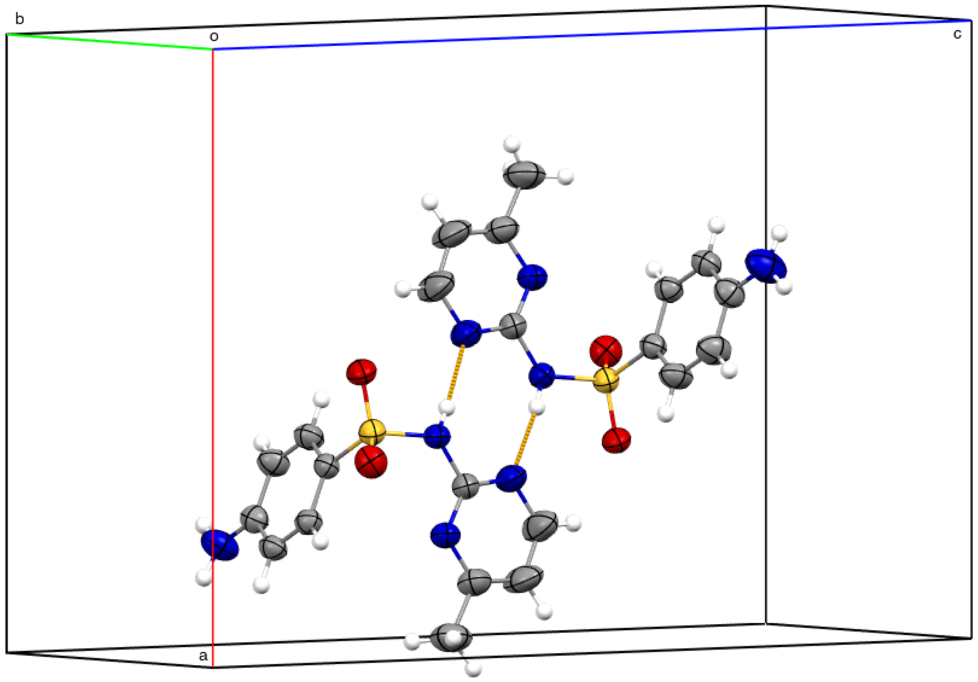

Figure 1.2.2. The asymmetric unit of sulfamerazine Form I collected at 296 K. The thermal ellipsoids are drawn at the 50% probability level. Colour scheme: carbon – dark grey, nitrogen – blue, oxygen – red, sulfur – yellow, hydrogen – white, hydrogen bonds – orange.

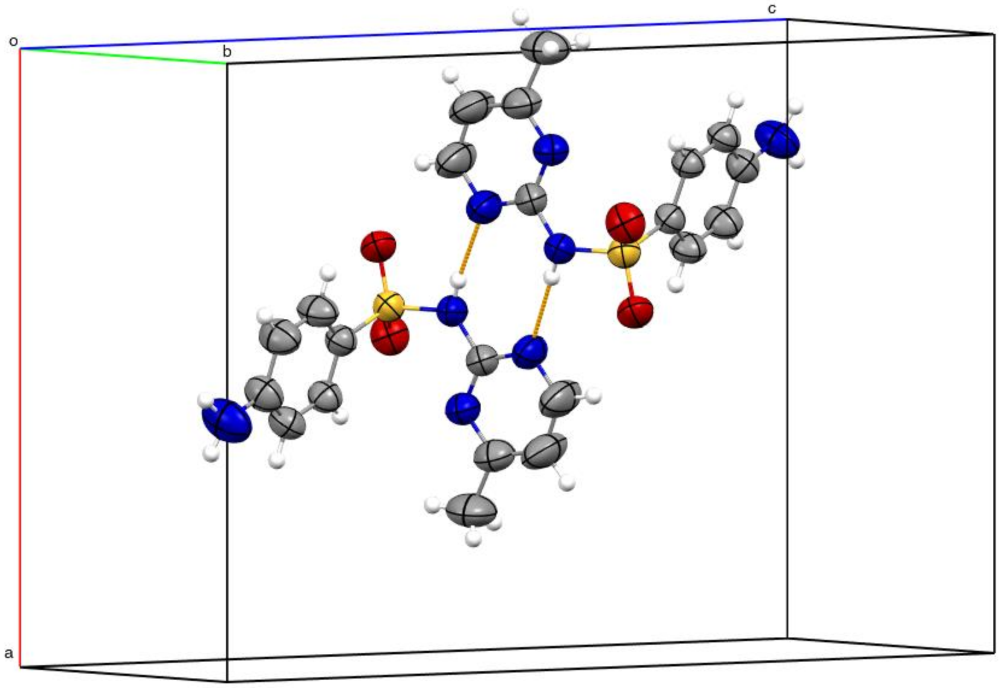

Figure 1.2.3. The asymmetric unit of sulfamerazine Form I collected at 413 K. The thermal ellipsoids are drawn at the 50% probability level. Colour scheme: carbon – dark grey, nitrogen – blue, oxygen – red, sulfur – yellow, hydrogen – white, hydrogen bonds – orange.

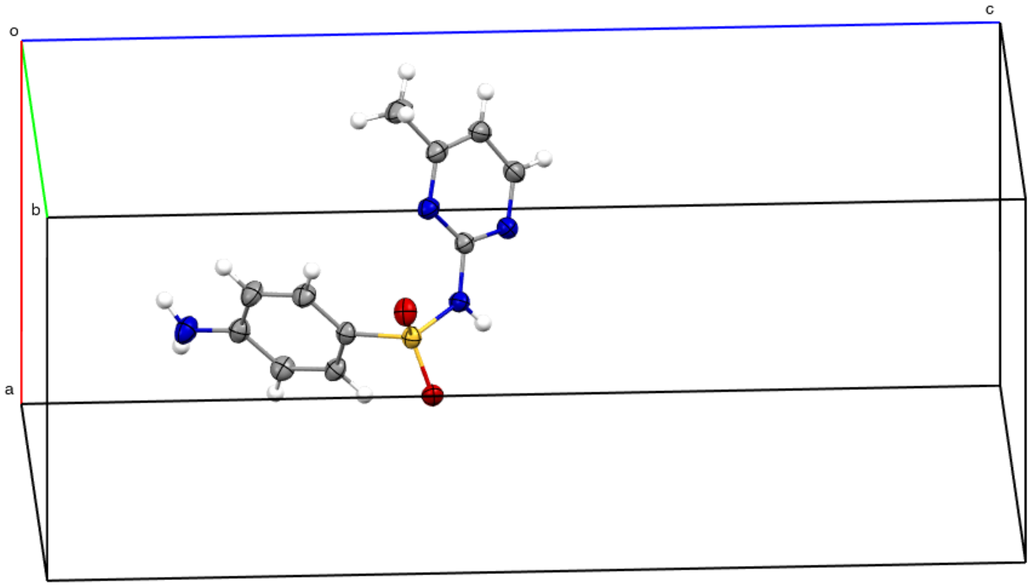

Figure 1.2.4. The asymmetric unit of sulfamerazine Form II collected at 150 K. The thermal ellipsoids are drawn at the 50% probability level. Colour scheme: carbon – dark grey, nitrogen – blue, oxygen – red, sulfur – yellow, hydrogen – white.

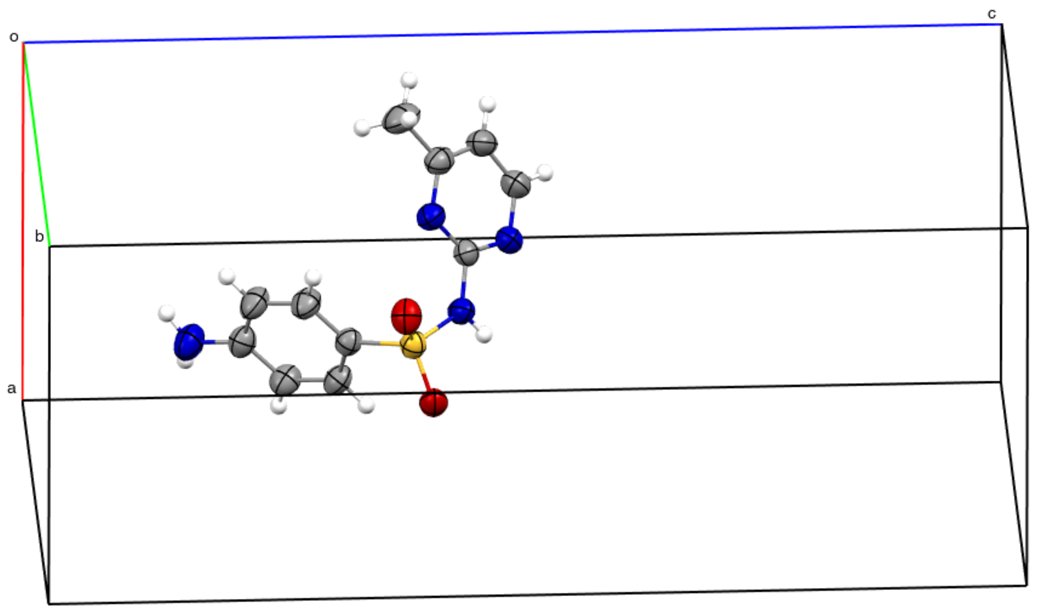

Figure 1.2.5. The asymmetric unit of sulfamerazine Form II collected at 296 K. The thermal ellipsoids are drawn at the 50% probability level. Colour scheme: carbon – dark grey, nitrogen – blue, oxygen – red, sulfur – yellow, hydrogen – white.

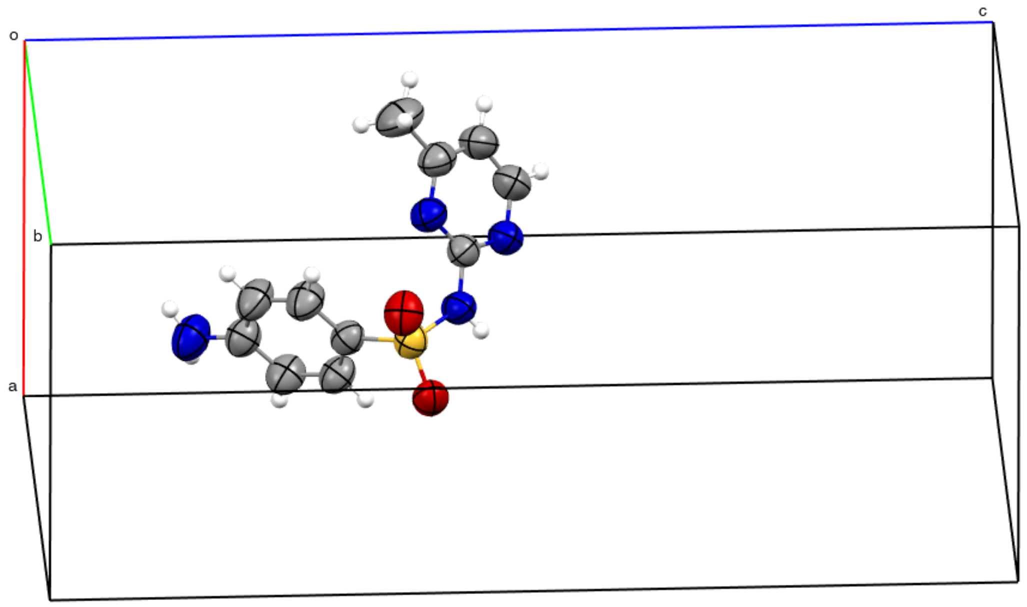

Figure 1.2.6. The asymmetric unit of sulfamerazine Form II collected at 413 K. The thermal ellipsoids are drawn at the 50% probability level. Colour scheme: carbon – dark grey, nitrogen – blue, oxygen – red, sulfur – yellow, hydrogen – white.

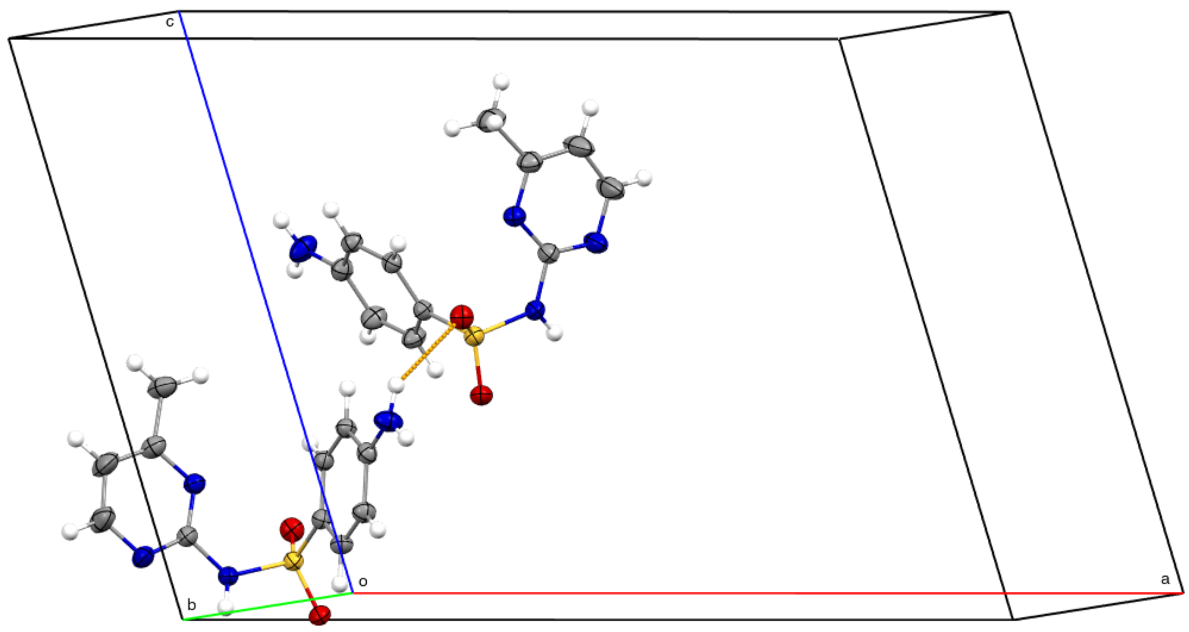

Figure 1.2.7. The asymmetric unit of sulfamerazine Form II collected at 413 K. The thermal ellipsoids are drawn at the 50% probability level. Colour scheme: carbon – dark grey, nitrogen – blue, oxygen – red, sulfur – yellow, hydrogen – white.

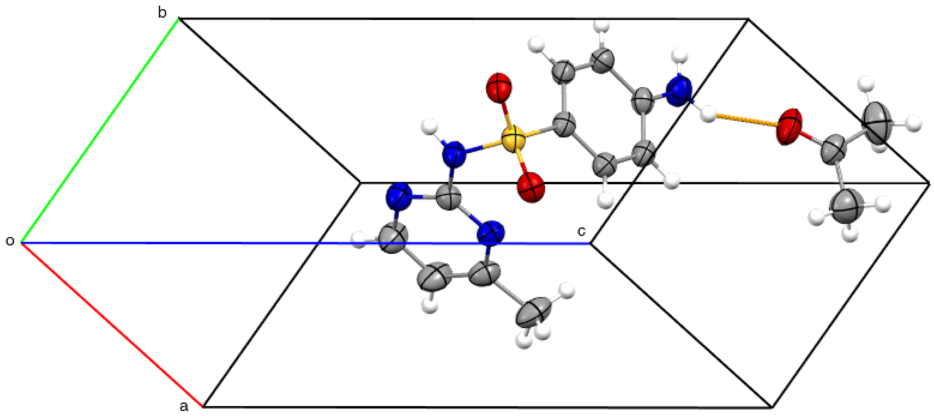

Figure 1.2.8. The asymmetric unit of the sulfamerazine acetone solvate. The thermal ellipsoids are drawn at the 50% probability level. Colour scheme: carbon – dark grey, nitrogen – blue, oxygen – red, sulfur – yellow, hydrogen – white, hydrogen bonds – orange.

Table 1.2.1. Crystallographic information and refinement parameters for sulfamerazine Form I collected at 150 K, 296 K and 413 K.

	Form I (150 K)	*Form I (296 K)*	*Form I (413 K)*
empirical formula	$C_{11}H_{12}N_4O_2S$	$C_{11}H_{12}N_4O_2S$	$C_{11}H_{12}N_4O_2S$
M / g mol^{-1}	264.31	264.31	264.31
crystal system	orthorhombic	orthorhombic	orthorhombic
space group	$Pna2_1$	$Pna2_1$	$Pna2_1$
a / Å	14.4646(2)	14.5018(2)	14.5464(2)
b / Å	8.18120(10)	8.21730(10)	8.24620(10
c / Å	21.7718(3)	21.9870(2)	22.2397(3)
α / °	90	90	90
β / °	90	90	90
γ / °	90	90	90
V / Å^3	2576.43(6)	2620.09(5)	2667.71(6)
Z	8	8	8
ρ_{calc} / g cm^{-3}	1.363	1.340	1.316
T / K	150	296	413
μ / mm^{-1}	2.257	2.219	2.180
F(000)	1104	1104	1104
crystal size / mm^3	0.20 × 0.10 × 0.08	0.20 × 0.10 × 0.08	0.36 × 0.23 × 0.17
radiation	CuK_α (λ = 1.54184 Å)	CuK_α (λ = 1.54184 Å)	CuK_α (λ = 1.54184 Å)
index ranges	$-16 \leq h \leq 18$<br>$-10 \leq k \leq 10$<br>$-27 \leq l \leq 25$	$-15 \leq h \leq 18$<br>$-10 \leq k \leq 9$<br>$-22 \leq l \leq 27$	$-17 \leq h \leq 13$<br>$-10 \leq k \leq 10$<br>$-26 \leq l \leq 26$
number of collected reflections	17319	18088	20049
unique reflections	4800	4829	4901
number of observed reflections	4474 [$I > 2\sigma(I)$]	4409 [$I > 2\sigma(I)$]	4254 [$I > 2\sigma(I)$]
R_{int}	0.0368	0.0356	0.0707
R(F), F > 2σ(F)	0.0386	0.0315	0.0559
$wR(F^2)$, $F > 2\sigma(F)$	0.1037	0.0868	0.1400
R(F), all data	0.0411	0.0348	0.0606
$wR(F^2)$, all data	0.1059	0.0888	0.1460
Δr (max., min.) / e Å^{-3}	0.180/−0.473	0.180/−0.288	0.249/−0.302
CCDC deposition number	2484990	2484991	2484992

Table 1.2.2. Crystallographic information and refinement parameters for sulfamerazine Form II collected at 150 K, 296 K and 413 K.

	Form II (150 K)	*Form II (296 K)*	*Form II (413 K)*
empirical formula	$C_{11}H_{12}N_4O_2S$	$C_{11}H_{12}N_4O_2S$	$C_{11}H_{12}N_4O_2S$
M / g mol^{-1}	264.31	264.31	264.31
crystal system	orthorhombic	orthorhombic	orthorhombic
space group	*Pbca*	*Pbca*	*Pbca*
a / Å	9.09040(1)	9.1370(2)	9.1954(5)
b / Å	11.54290(10)	11.7026(2)	11.8656(7)
c / Å	22.86160(10)	22.8620(3)	22.9266(14)
α / °	90	90	90
β / °	90	90	90
γ / °	90	90	90
V / Å^3	2398.86(2)	2444.56(10)	2501.5(3)
Z	8	8	8
ρ_{calc} / g cm^{-3}	1.464	1.436	1.404
T / K	150	296	413
μ / mm^{-1}	2.422	2.379	2.324
F(000)	1104	1104	1104
crystal size / mm^3	0.36 × 0.24 × 0.12	0.22 × 0.21 × 0.16	0.34 × 0.30 × 0.26
radiation	CuK_α (λ = 1.54184 Å)	CuK_α (λ = 1.54184 Å)	CuK_α (λ = 1.54184 Å)
index ranges	$-10 \leq h \leq 10$<br>$-13 \leq k \leq 13$<br>$-27 \leq l \leq 27$	$-9 \leq h \leq 10$<br>$-11 \leq k \leq 13$<br>$-27 \leq l \leq 26$	$-10 \leq h \leq 9$<br>$-11 \leq k \leq 14$<br>$-27 \leq l \leq 24$
number of collected reflections	50899	10040	10799
unique reflections	2122	2150	2200
number of observed reflections	2056 [$I > 2\sigma(I)$]	1894 [$I > 2\sigma(I)$]	1861 [$I > 2\sigma(I)$]
R_{int}	0.0349	0.0252	0.0282
$R(F)$, $F > 2\sigma(F)$	0.0288	0.0293	0.0361
$wR(F^2)$, $F > 2\sigma(F)$	0.0759	0.0809	0.1131
$R(F)$, *all data*	0.0294	0.0334	0.0412
$wR(F^2)$, *all data*	0.0763	0.0828	0.1173
Δr (max., min.) / e Å^{-3}	0.199/−0.440	0.191/-0.293	0.137/-0.266
CCDC deposition number	2484993	2484994	2484995

Table 1.2.3. Crystallographic information and refinement parameters for sulfamerazine Form V and the sulfamerazine acetone solvate.

	Form V	*Form V*	*Acetone solvate*
empirical formula	$C_{11}H_{12}N_4O_2S$	$C_{11}H_{12}N_4O_2S$	$C_{14}H_{18}N_4O_3S$
M / g mol⁻¹	264.31	264.31	322.38
crystal system	monoclinic	monoclinic	triclinic
space group	$P2_1/c$	$P2_1/c$	$P\bar{1}$
a / Å	22.7355(2)	22.6961(4)	7.9685(5)
b / Å	8.18664(6)	8.16100(10)	8.1220(5)
c / Å	14.45767(13)	14.4100(2)	14.5539(4)
α / °	90	90	79.039(4)
β / °	106.6773(10)	106.548(2)	76.528(5)
γ / °	90	90	69.773(6)
V / Å^3	2577.77(4)	2558.51	853.30(9)
Z	8	8	2
ρ_{calc} / g cm^{-3}	1.362	1.372	1.255
T / K	150	100	150
μ / mm^{-1}	2.259	2.272	1.850
F(000)	1104	1104	340
crystal size / mm^3	0.17× 0.11 × 0.04	0.12 x 0.05 x 0.02	0.20 × 0.12 × 0.10
radiation	CuK_α (λ = 1.54184 Å)	CuK_α (λ = 1.54184 Å)	CuK_α (λ = 1.54184 Å)
index ranges	$-28 \le h \le 27$<br>$-10 \le k \le 10$<br>$-18 \le l \le 18$	$-28 \le h \le 28$<br>$-10 \le k \le 10$<br>$-12 \le l \le 17$	$-9 \le h \le 9$<br>$-9 \le k \le 9$<br>$-17 \le l \le 17$
number of collected reflections	54611	47734	14606
unique reflections	5344	5537	3013
number of observed reflections	4592 [$I > 2\sigma(I)$]	4442	2552 [$I > 2\sigma(I)$]
R_{int}	0.0382	0.0774	0.0566
$R(F)$, $F > 2\sigma(F)$	0.0375	0.0573	0.0445
$wR(F^2)$, $F > 2\sigma(F)$	0.0929	0.1426	0.1224
$R(F)$, *all data*	0.0455	0.0716	0.0511
$wR(F^2)$, *all data*	0.0970	0.1495	0.1286
Δr (*max., min.*) / e Å^{-3}	0.230/−0.420	0.493/-0.495	0.415/−0.388
CCDC deposition number	2484996	2487129	2484997

References

[1] CrysAllisPro, Agilent Technologies Inc., 2022.

[2] G. M. Sheldrick, SHELXT - Integrated space-group and crystal-structure determination. Acta Crystallogr A., 2015, **64**, 3–8.

[3] O. V. Dolomanov, L. J. Bourhis, R. J. Gildea, J. A. K. Howard, H. Puschmann, OLEX2: a complete structure solution, refinement and analysis program., J. Appl. Crystallogr. 2009, **42**,

339–341.
[4] G. M Sheldrick, Crystal structure refinement with SHELXL. Acta Crystallogr C., 2015, **71**, 3–8.
[5] C. B. Hübschle, G. M. Sheldrick and B. Dittrich, ShelXle: a Qt graphical user interface for SHELXL, J. Appl. Cryst., 2011, **44**, 1281–1284.

1.2.1. Form II

Crystals of form II were grown by slow evaporation from acetonitrile : water by stirring excess SMZ in MeCN : H_2O (80:20, v:v) for up to one day at room temperature. The resulting slurry was filtered into separate vials with small holes to allow slow evaporation. The vials were then left at room temperature, and crystals were observed after between a few days and weeks.

1.2.2. Form V

Contributed by Dejan-Krešimir Bučar, Marlena Gryl, Paul Tinnemans and William Wood

UCL: The experiment that produced crystals of form V suitable for SXRD, used for the structure determination, was slurrying form I in MeCN:H_2O (80:20, v:v) standing on a hot plate at 60 °C for 5 days. This generated form II, so the temperature was increased to 85 °C. After 1 day, this was still form II, the hot plate was increased to 120 °C , which dissolved all the material. The temperature was reduced to 95 °C, which generated a powder of form V, and this was left for 5 days to cool. After cooling the there was a mixture of form I and form V crystals.

Radboud: Suitable crystal for structure determination of form V were produced by stirring a saturated solution of form I in MeCN:H_2O (80:20, v:v) for 7 days. The saturated solution as filtered and left to slowly evaporate in several days. After part of the solvent evaporated suitable single crystals of form V were formed and could be taken from the liquid.

Jagiellonian University: Hot-stage microscopy of sulfamerazine form I demonstrated that, near the melting point (237 °C), new small crystals formed as a result of sublimation and re-crystallization (from condensation droplets) on the cover slip. The small, well faceted crystals (Figure 1.2.9, left, marked with pink circles) appeared in the vicinity of larger form I crystals. On further heating (Figure 1.2.9, middle), additional crystallites developed, while form (I) slowly approached melting. When the sample was subjected to small temperature cycles just

below the form I melting temperature, and the heating rate was reduced to 2 °C/min, further sublimation and condensation was observed. Single-crystal X-ray diffraction confirmed that these newly formed crystals correspond to the previously unreported Form V of SMZ.

The fact that SMZ starts to decompose upon reaching the melting temperature (orange-brownish colour, Figure 1.2.9, right) did not allow us to derive the equilibrium melting temperature of the SMZ form I and V. Nevertheless, the investigation revealed that the melting temperatures of the two polymorphs are very close, within less than 1 °C.

Form (V) was also obtained independently under microwave-assisted, high pressure crystallization. Sulfamerazine (ca. 60 mg) was dissolved completely in acetonitrile (6 mL) at 120 °C within Anton Paar Monowave 450 reactor. The solution was rapidly cooled to 50 °C and was removed from the reactor and left at ambient temperature, leading to crystallization of a mixture of form (I) and form (V), as confirmed by single-crystal X-ray diffraction analysis.

It is worth noting that once crystallized, form (V) remained unchanged and did not transform into other polymorphs after isolation from solution (at least for couple of weeks).

These results highlight that multiple crystallization pathways can lead to form (V), while also demonstrating that competing polymorphs may form concommitantly under similar conditions, underlying the complexity of sulfamerazine's polymorphic landscape.

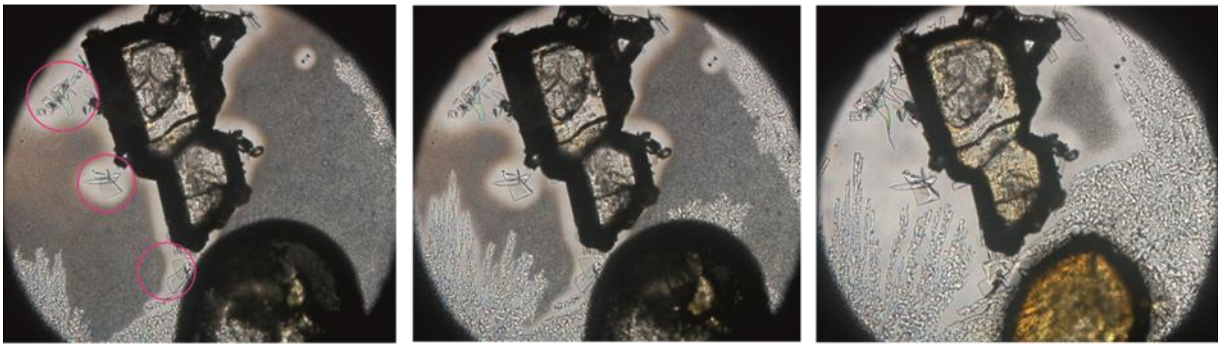

Figure 1.2.9. Hot-stage micrography experiment of sulfamerazine showing the emergence of a new polymorph (V) via sublimation and recrystallization of condensation droplets. Left – small crystals of form V emerge (marked with pink circles), middle – additional (V) crystallites develop below the melting point of form I. Right – additional (V) crystallizes in the form of larger crystals when cycling experiments are performed. Linkam hot stage was mounted on Zeiss Axio Scope.A1. Images produced with 200× magnification.

Overlay of the sulfamerazine polymorphs form (V) (green, $P2_1/c$) and form (I) (orange, $Pna2_1$) shows (Figure 1.2.10) similar molecular conformations (upper part of the picture), so the

polymorphism is packing driven, not conformational (centrosymmetric dimers that form zig-zag chains in (V) vs polar ribbons in (I) – Figure 1.2.11). The **b** axes nearly coincide (~8.16 Å), whereas a shear in the **ac** plane with β ≈ 106.5° in (V) vs 90° in (I) reflects the symmetry difference. Both polymorphs have identical packing efficiency (0.69).

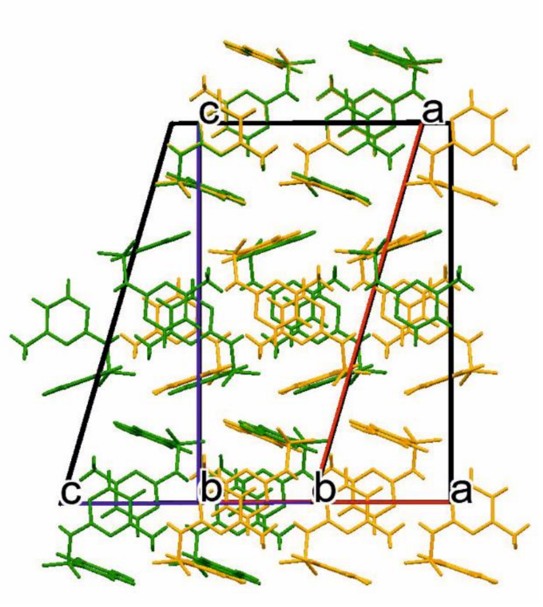

Figure 1.2.10. Overlay of sulfamerazine polymorphs (ca. along b). Superposition of Form V (green) and form I (orange) shows closely matching molecular conformations. The overlay underscores that the polymorphism is packing/topology driven and not induced by the change of conformations.

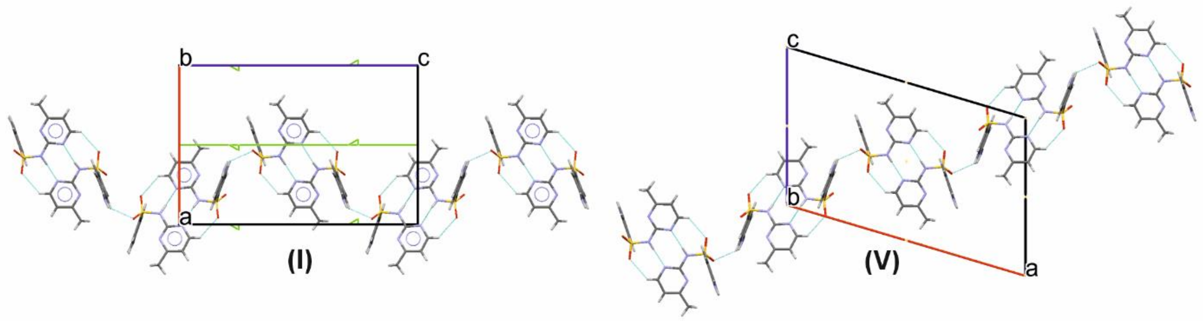

Figure 1.2.11. Structural motifs in sulfamerazine polymorphs (I) and (V) with symmetry elements highlighted. Left (form (I), Pna21) polar axis marked in green. Sulfamerazine molecules form dimers cross-connected to form polar ribbons. Right (form (V), P21/c) – inversion centres located at the midpoint of the centrosymmetric dimers are marked in orange.

1.2.3. Acetone solvate structure

Contributed by Dejan-Krešimir Bučar and William Wood

A single crystal of the acetone solvate of SMZ was grown by slow evaporation from acetone.

Table 1.2.4. Sulfamerazine solvate structures.

Solvate	T / K	Space Group	Z’	R-factor / %	a / Å	b / Å	c / Å	α / °	β / °	γ / °	Refcode/ notes
THF	150	P21/c	3	6.28	10.5765	11.8088	39.1512	90.00	92.62	90.00	AKOBUZ[1]
1,4-dioxane (1:1)	110	P21/c	1	4.62	11.5110	12.4360	12.0950	90.00	102.05	90.00	FALSES[2]
1,4-dioxane (1:0.5)	110	P-1	1	4.64	7.7675	8.1233	12.631	91.16	95.69	105.23	FALSIW[2]
DMF	110	P-1	2	7.25	9.7740	12.5560	14.6590	76.58	77.57	86.18	FALSOC[2]
DMA	110	P-1	1	6.41	7.6792	8.3101	15.1250	93.13	101.81	104.77	FALSUI[2]
Cyclopentanone	110	P-1	1	5.71	5.9650	9.6479	14.8170	76.16	83.05	89.71	FALTAP[2]
3-Picoline	110	P-1	1	4.77	7.8319	8.1117	15.3580	76.47	77.04	73.54	FALTET[2]
Acetone	150	P-1	1	4.47	7.9685	8.1220	14.5539	79.04	76.53	69.78	UCL

There are currently seven reported solvates of SMZ on the CSD (Table 1.2.4). Here, we reported an eighth acetone solvate of SMZ. Of the now eight known solvates, five (Acetone, 1,4-dioxane (1:0.5), DMF, DMA, 2-picoline) show similar packing of layers with solvate filling the cavities between (Figure 1.2.12). In a 30-molecule crystal packing overlay, the minimum match between those structures is 15, with the 3-picoline solvate (FALTET) overlaying 30/30 molecules with the acetone solvate, FALSOC and FALSUI and 25 molecules with FALSIW (Figure 1.2.13). There is also some similarity between the layers seen in this group of five solvates and those seen in the neat forms I, III and V (Figure 1.2.12C) but the solvent inserted between the sheets disrupts the packing enough that only 6/30 molecules match in a crystal packing similarity overlay with the default parameters (Figure 1.2.13). This similarity of the hydrogen bonded dimer layers explains why the solvates mostly desolvate into form I[2] rather than the low-temperature stable form II.

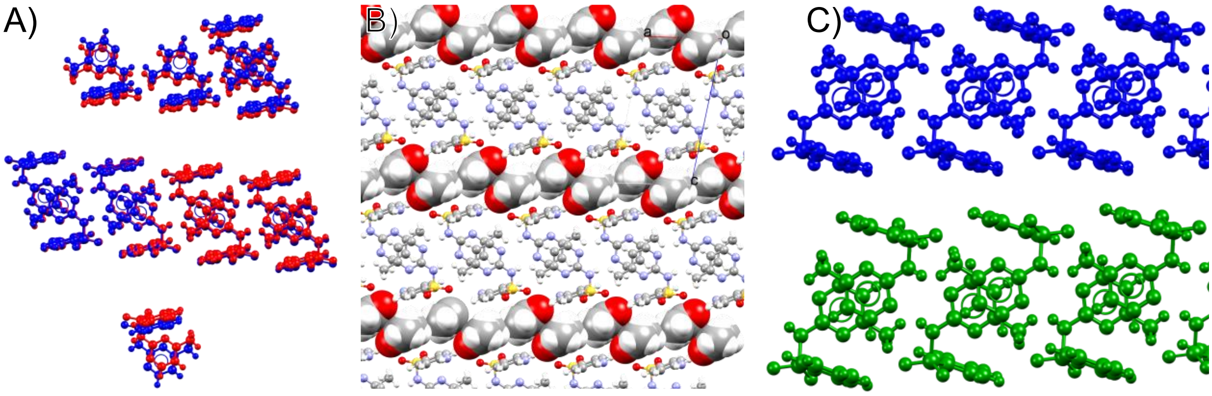

Figure 1.2.12. Crystal packing overlay of 3-picoline solvate, FALTET (blue) and acetone solvate (red), voids between the layers are filled with solvent. B) Packing of the acetone solvate with solvent in shown as space fill. C) FALTET layer (blue) Form I layer (green).

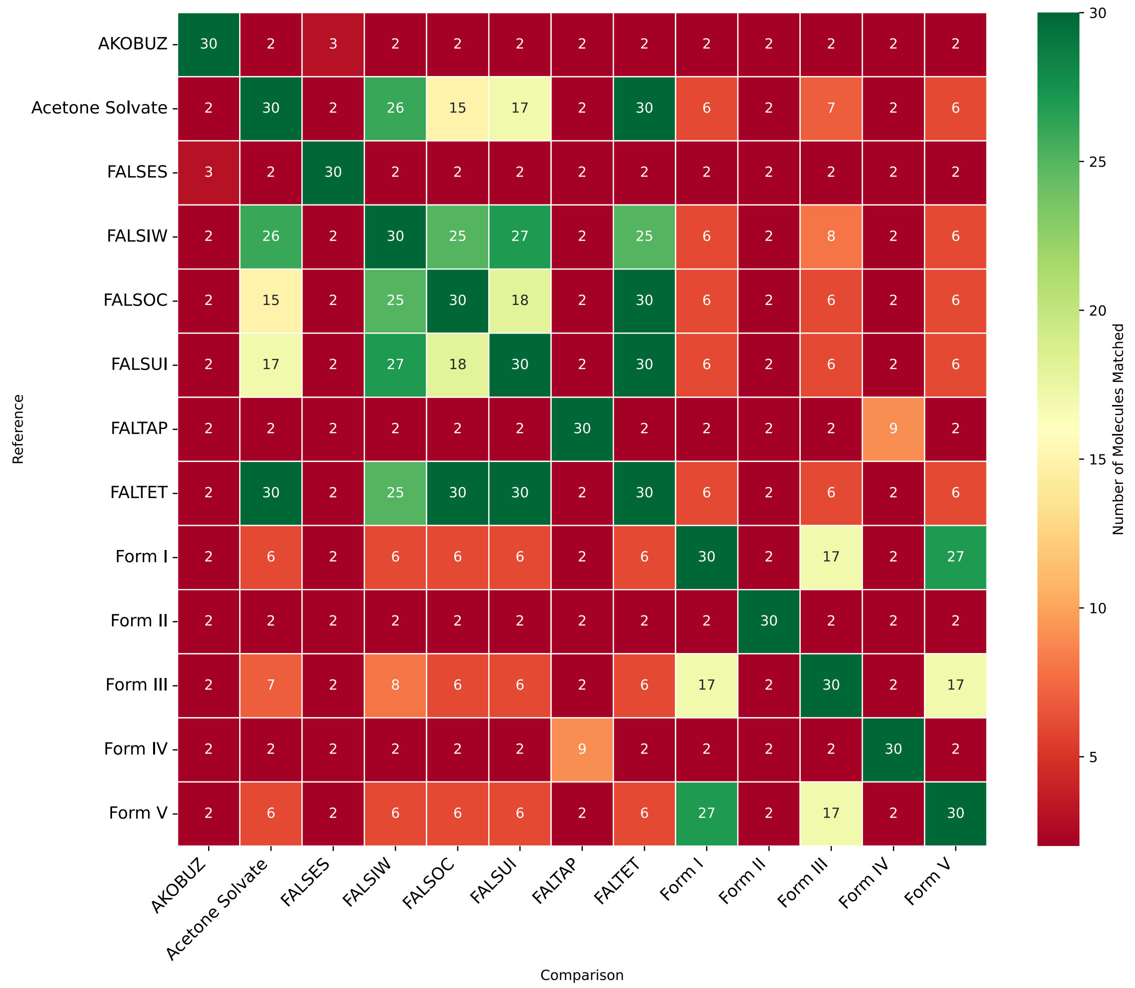

Figure 1.2.13. Crystal packing similarity map of the neat forms of SMZ and solvates. The values in squares are the number of molecules matched out of 30 by a Compare Similarity Calculation with default parameters excluding solvent molecules, using Mercury.

References

[1] Hossain, G. Sulfamerazine tetrahydrofuran monosolvate. *IUCrData* **2016**, *1* (4), x160596.

[2] Aitipamula, S.; Chow, P. S.; Tan, R. B. H. The solvates of sulfamerazine: structural, thermochemical, and desolvation studies. *CrystEngComm* **2012**, *14* (2), 691-699. DOI: 10.1039/C1CE06095C.

1.3. Variable temperature Powder X-ray diffraction.

Contributed by Doris Braun, Ivo Rietveld and Nicolas Couvrat

Innsbruck: Variable-temperature PXRD experiments were performed using an XRDynamic 500 diffractometer (Anton Paar, Austria) equipped with a Pixos 2000 detector and operated in transmission geometry. Samples were loaded into 0.5 mm glass capillaries and continuously

rotated during measurement. Temperature control was achieved using a TTK 600 heating stage (Anton Paar, Austria). Patterns were recorded from 2θ = 2°–45° (step size: 0.010°, 150 s/step, multi-channel detector) in 10 °C increments between 30–200 °C. At each step, the temperature was held constant for approx. 45 minutes.

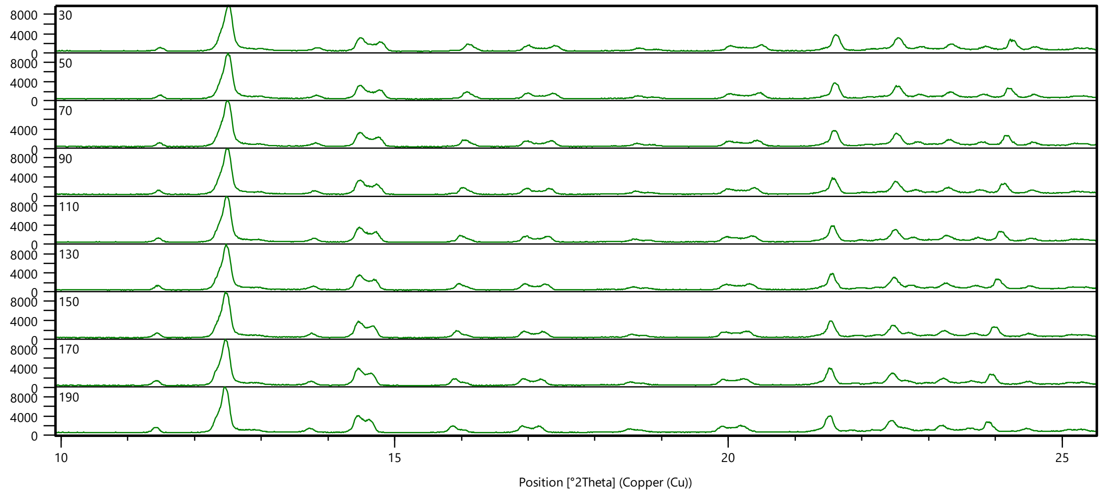

Figure 1.3.1. Variable temperature PXRD on form V on heating (Innsbruck), temperatures are in °C.

University of Rouen: Temperature-resolved X-ray diffraction (TR-XRD) patterns were recorded using a D8 series II from Bruker, equipped with a TTK-450 hot-stage sample holder, a copper anticathode source with Kα radiation (λ= 1.5418Å, voltage 40 kV and tube current 40 mA), and a Lynx eye linear detector. PXRD patterns were measured from 4° to 50° in 2-theta with a step size of 0.02° and step duration of 1s. Cooling down to -130°C was ensured by a in-house constructed liquid nitrogen setup using an Apollo 150 cryogenic container (Cryotherm company) controlled by a SC 5 safety controller (KGW isotherm). The heating rate applied between each measurement temperature was 0.03°C per second. Refinements of the data were carried out by Topas Academic V4.

The late appearance of form V resulted in the VT-XRPD measurements being carried out before the presence of form V in the sample was known. Two approaches could be used for accounting for this. Figure 1.3.2 assumes that form V causes the discontinuity in Figure 8 for form I, and shifts the lattice parameters to be in line with the low-temperature single-crystal measurements. Figure 1.3.3 reanalyzes the data by indexing only those peaks that are definitely form I, which reduces the stability of the fits.

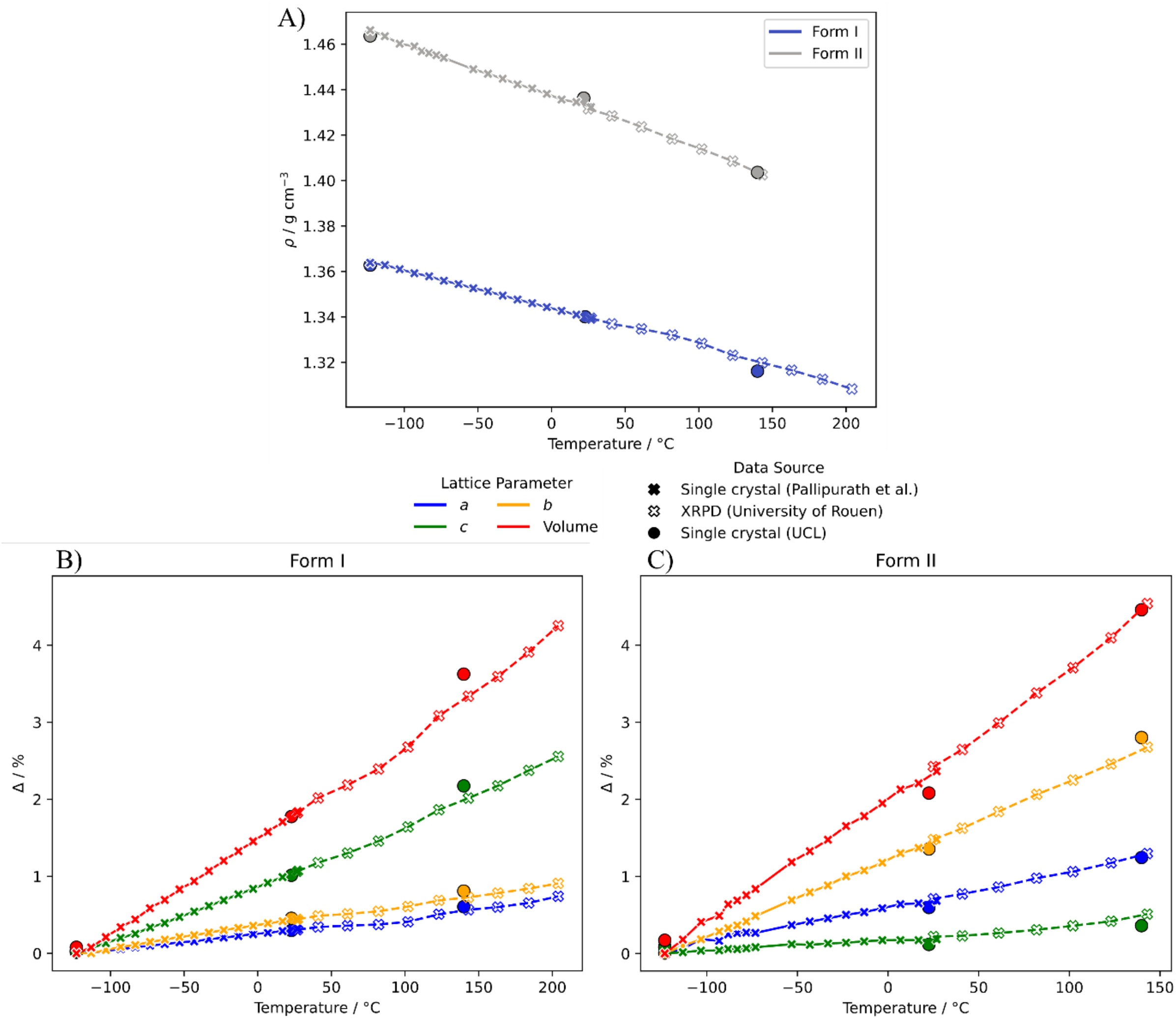

Figure 1.3.2. Structural data as a function of temperature, single-crystal X-ray diffraction data from Pallipurath et al.[1] in solid crosses/lines and from UCL in solid circles, XRPD(T) data contributed by Rouen in empty crosses/dashed lines. Densities of form I (blue) and II (grey) (A). Percentage change in the lattice parameters and volume of form I (B) and II (C). XRPD(T) data has *a*, *b* and *c* is shifted to fit with the low temperature single crystal data with the knowledge that the sample of form I used in the measurements has form V contamination.

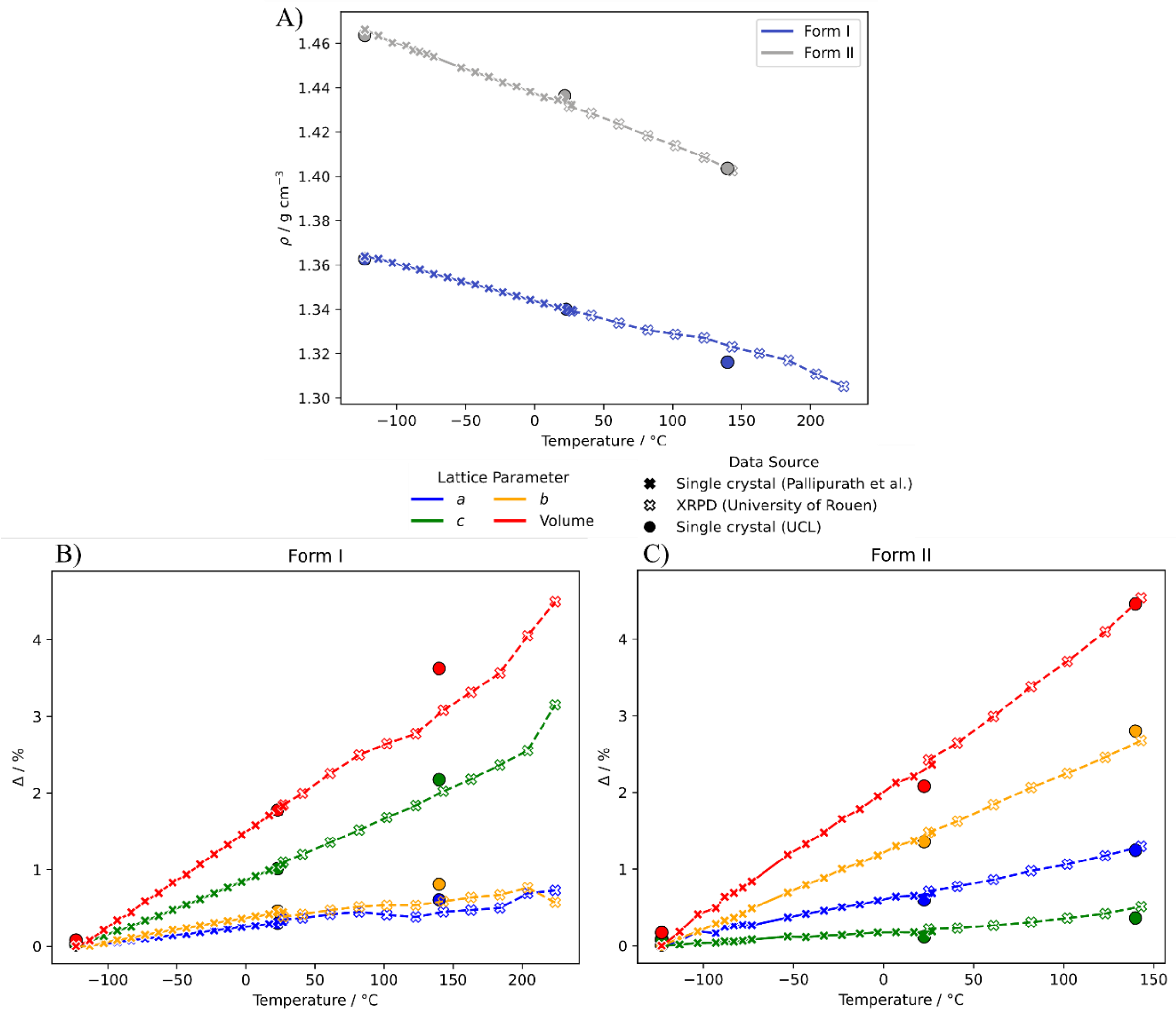

Figure 1.3.3. Structural data as a function of temperature, single-crystal X-ray diffraction data from Pallipurath et al.[1] in solid crosses/lines and from UCL in solid circles, XRPD(T) data contributed by Rouen in empty crosses/dashed lines. Densities of form I (blue) and II (grey) (A). Percentage change in the lattice parameters and volume of form I (B) and II (C). XRPD(T) data is indexed XRPD data is indexed by fitting a single zero error for the Rouen XRPD equipment to the data obtained by Pallipurath et al. The zero error is subsequently kept constant for the fits at higher temperatures. The presence of form V causes more variations in the lattice parameters.

References

[1] Pallipurath, A. R.; Skelton, J. M.; Warren, M. R.; Kamali, N.; McArdle, P.; Erxleben, A. Sulfamerazine: Understanding the Influence of Slip Planes in the Polymorphic Phase Transformation through X-Ray Crystallographic Studies and ab Initio Lattice Dynamics. *Molecular Pharmaceutics* **2015**, *12* (10), 3735-3748, Article. DOI: 10.1021/acs.molpharmaceut.5b00504.

1.4. Additional Competitive Slurry and Grinding Experiments

Contributed by Doris Braun and William Wood

Across all the slurry experiments, including different solvents (Figure 1.4.1 and Figure 1.4.2) and different input forms (Figure 1.4.3), when given enough time, a clear pattern emerged. Form II was the most stable at 45 °C and below and form V was the most stable at 50 °C and above. This observation was also confirmed independently by both Innsbruck (Fig 5 m/s) and UCL (Fig 6 m/s). Furthermore, liquid-assisted grinding experiments were carried out in MeCN:H_2O (80:20, v:v) (Figure 1.4.4) which resulted in either Form II or V, which supports the conclusion that Form I is indeed metastable at all the temperatures we have investigated.

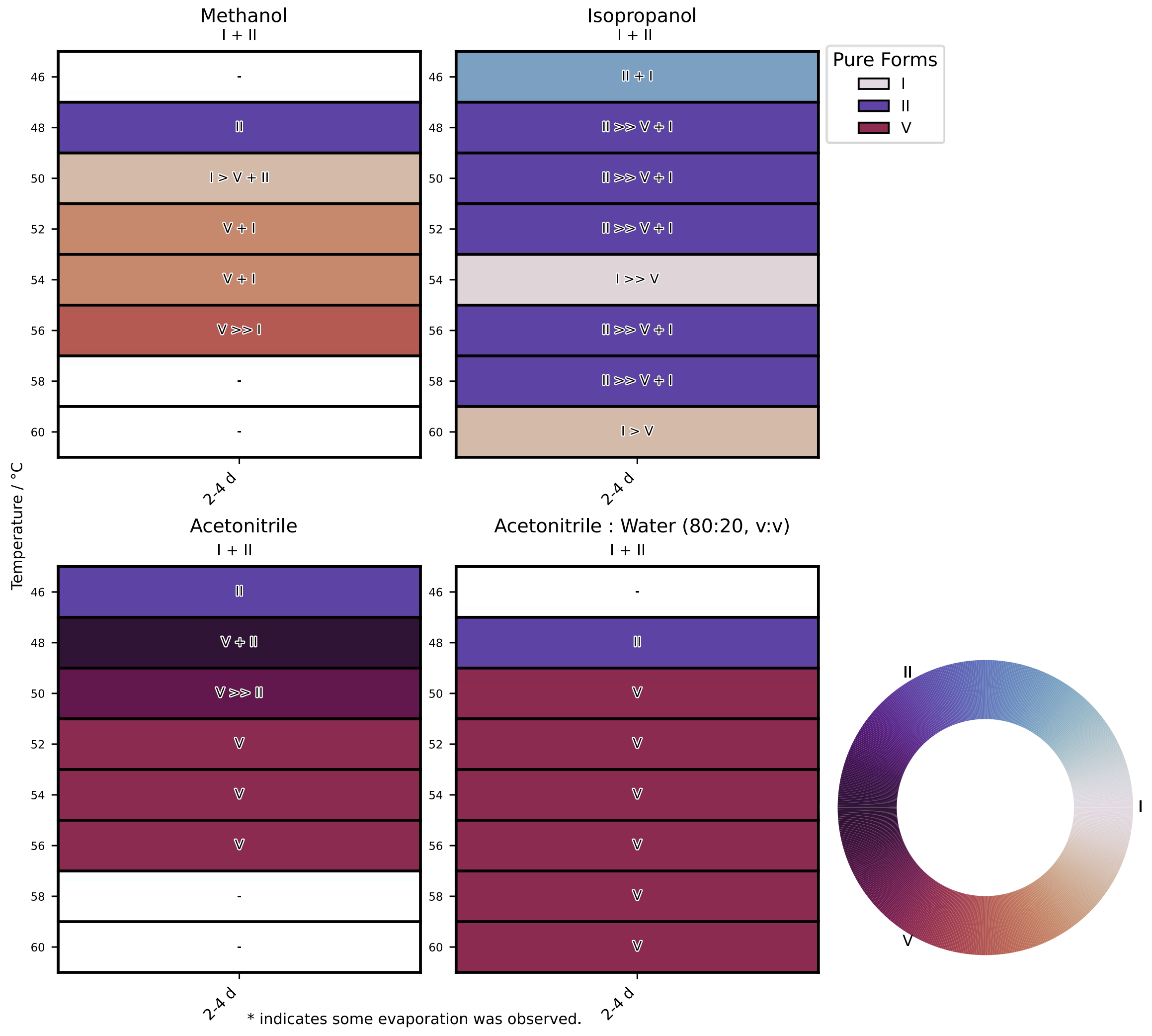

Figure 1.4.1. First series of competitive slurry experiments carried out in four solvents, when a distinct diffractogram was first noticed. Inputs were all equal mixture of form I and II by weight (form I was commercial Toku-e material was contaminated by form V). Form is measured by PXRD after between 2-4 days. The result is displayed in the cell and by colour after the time displayed. * Indicates some evaporation was observed

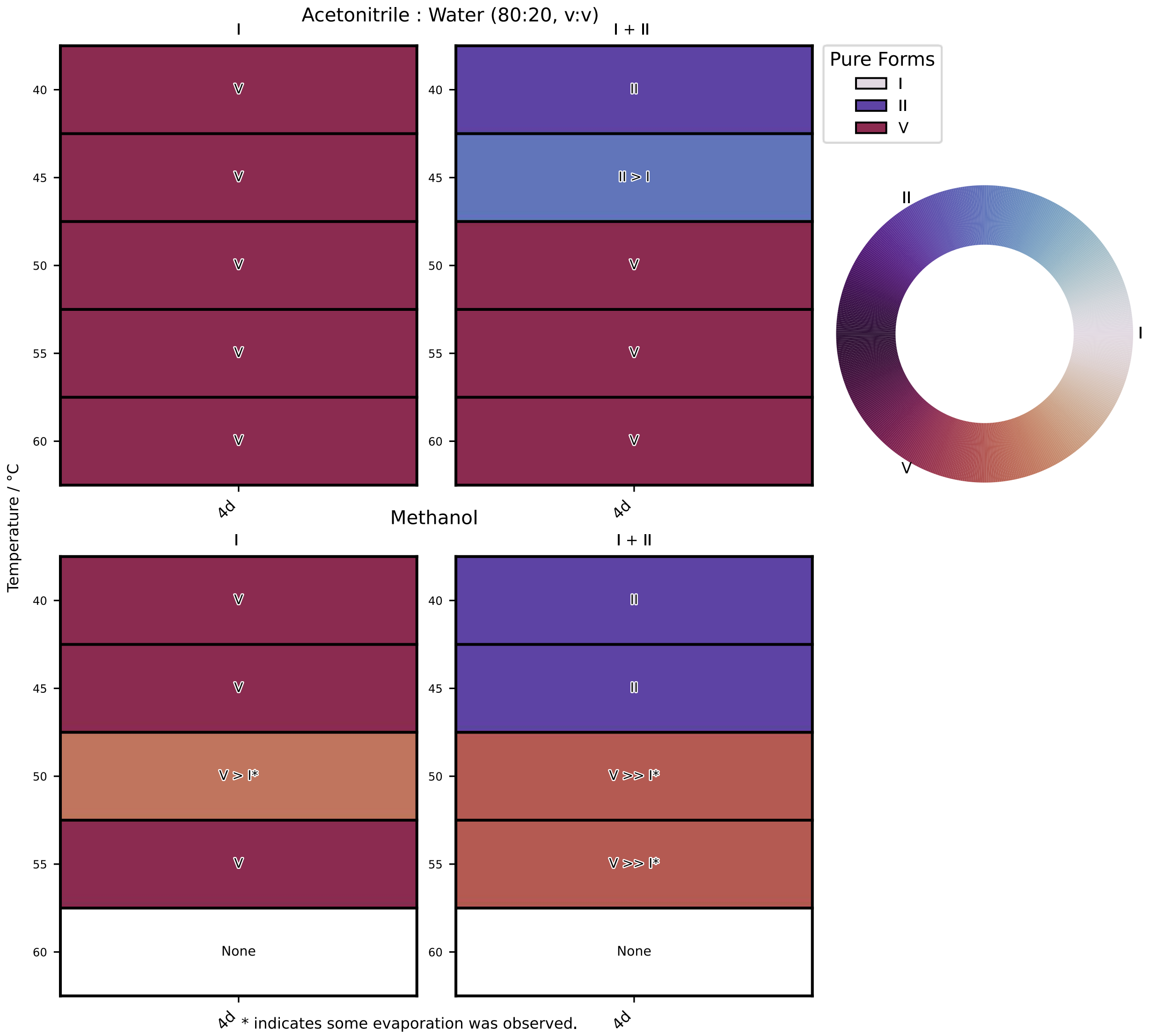

Figure 1.4.2. Second series of competitive slurry experiments carried out in methanol and acetonitrile : water (80:20, v:v), input form at the top of each result section that shows the form measured by PXRD all after 4 days. Form is measured by PXRD after between 2-4 days. The result is displayed in the cell and by colour after the time displayed. * Indicates some evaporation was observed.

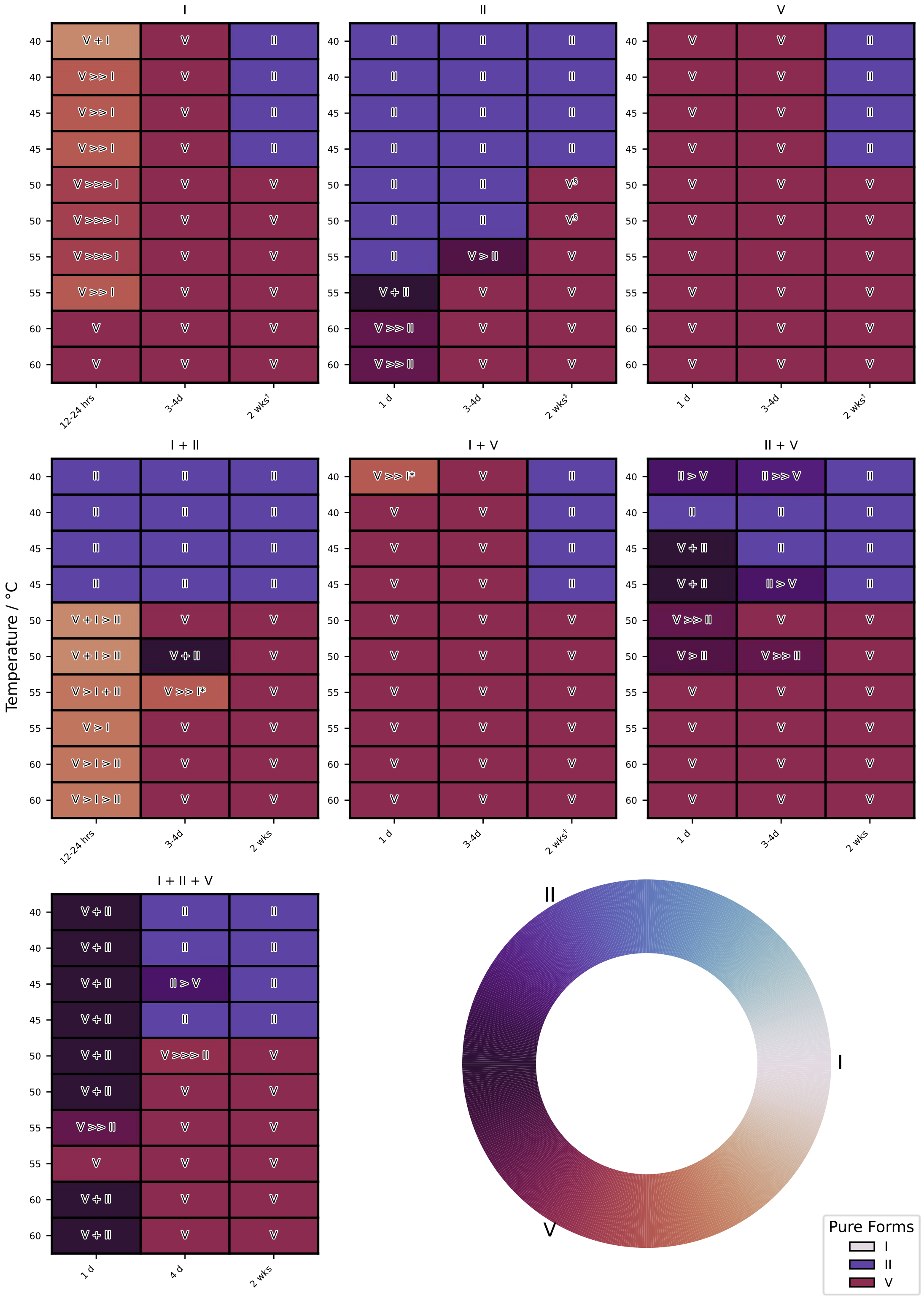

Figure 1.4.3. Additional competitive slurry experiments in MeCN:H_2O (80:20, v:v) the input form(s) are at the top of each block. Form is measured by PXRD. The result is displayed in the cell and by colour after the time displayed. * Indicates some evaporation was observed, § Measured after 4 weeks not 2, †Form II seeds added after 4 days. ‡Form V seeds added after 4 days.

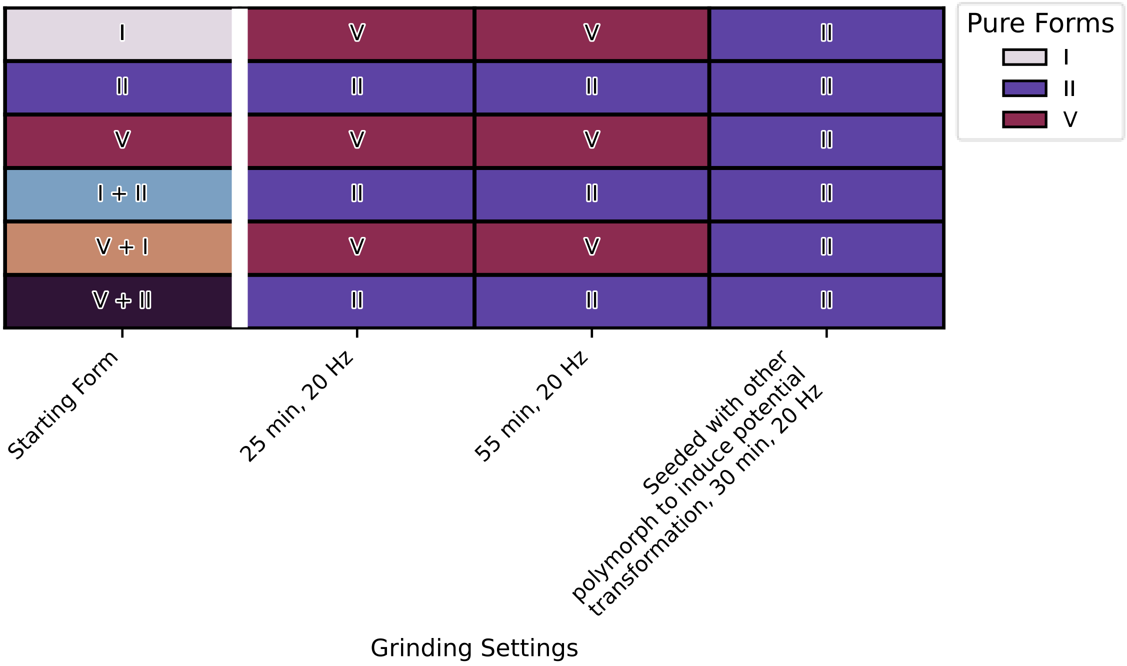

Figure 1.4.4. Grinding experiments carried out in a 5ml jar with 20 µl of MeCN:H_2O (80:20, v:v) at room temperature.

1.5. Solid-state NMR spectra

Contributed by Erika Bartůňková, Martin Dračínský

Sulfamerazine form I was purchased from Sigma-Aldrich and was used as obtained. Sulfamerazine form II was provided by Vojtech Stejfa and form V was provided from UCL.

High-resolution ^{13}C ss-NMR spectra were acquired on a JEOL ECZ600R spectrometer operating at 600.2 MHz for ^{1}H and 150.9 MHz for ^{13}C. Samples were loaded into 3.2-mm magic angle spinning (MAS) rotors. Measurements were conducted at MAS rates of 18 kHz. Cross polarization (CP) with a ramped amplitude shape pulse was employed for acquiring the ^{13}C spectra. The contact time was 5 ms for standard ^{13}C spectra and 50 µs for ^{13}C spectra with suppressed signals of quaternary carbon atoms. To determine relaxation delays, proton T_1 relaxation times were estimated from ^{1}H saturation recovery experiments. The relaxation delay was set to 1.5 times the corresponding T_1 time. Carbon chemical shifts were referenced against the signal of DSS, which was used as an internal standard ($\delta(^{13}C) = 0$ ppm). The temperature was set to 23 °C. Real sample temperature was estimated to be 40 °C, using the ^{207}Pb shift in solid $Pb(NO_3)_2$ at the same MAS frequency and spectrometer setting.[1] The assignment of carbon signals is based on comparison with solution-state NMR data and on the CP-MAS experiment with suppressed signals of quaternary carbon atoms.

Table 1.5.1. Experimental ^{13}C NMR chemical shifts (ppm) of two forms of sulfamerazine.

Carbon atom	Form I ($Z' = 2$)	Form II ($Z' = 1$)
C1	154.1	152.6
C2, C6	112.3–114.6	111.7
C3, C5	128.3, 132.5, 133.5	129.9
C4	122.4	123.9
C2′	156.3	156.5
C4′	169.0	170.7
C5′	112.3–114.6	117.2
C6′	158.3	159.2
CH_3	23.6	24.6

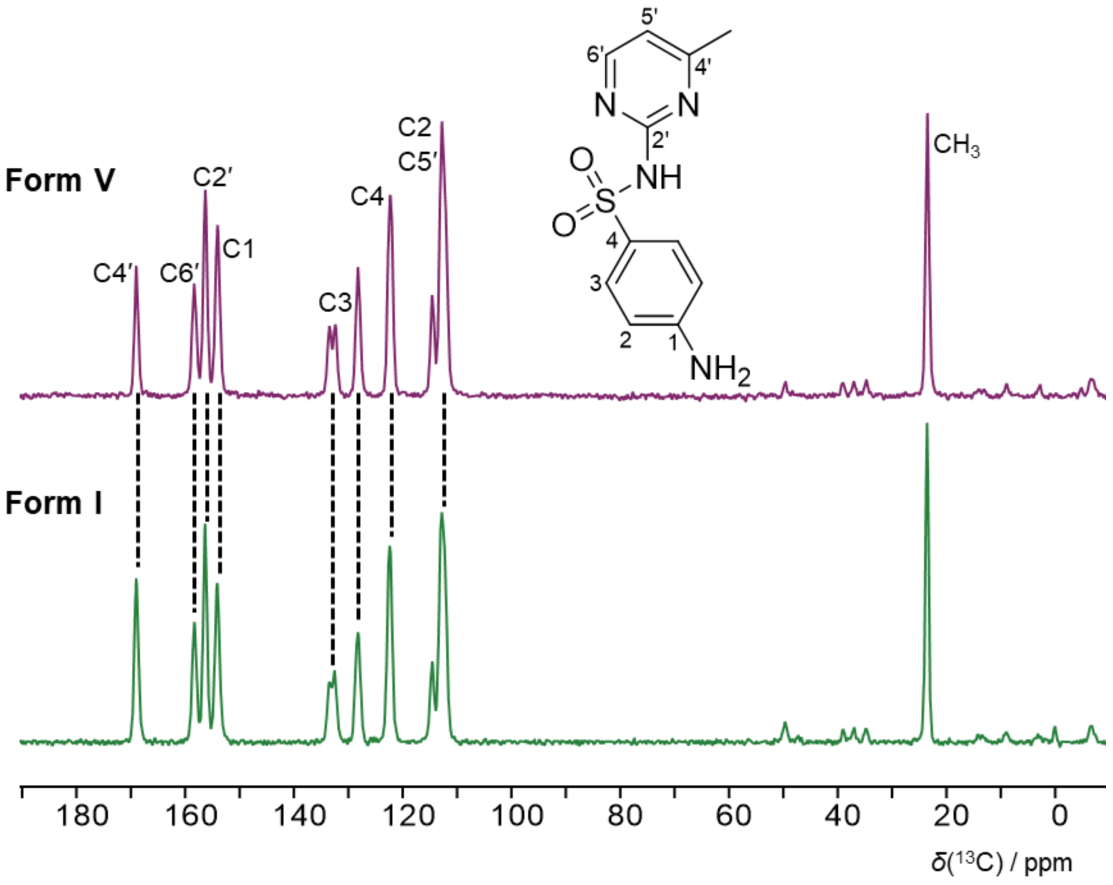

Figure 1.5.1. Solid-state ^{13}C NMR of SMZ form I and II. The matching peaks show the two forms are indistinguishable by ss-NMR.

References

[1] Bielecki, A.; Burum, D. P. Temperature-Dependence of ^{207}Pb MAS Spectra of Solid Lead Nitrate - an Accurate, Sensitive Thermometer for Variable-Temperature MAS. *J. Magn. Reson. A* **1995**, *116* (2), 215-220. DOI: DOI 10.1006/jmra.1995.0010.

1.6. Differential Scanning Calorimetry (DSC)

Table 1.6.1. DSC measurements carried out at each institution as part of the BEST-CSP work. 2σ is 2 times the standard error.

Heating Rate / K min^{-1}	Onset Temperature / °C	Onset Temperature / K	2σ / °C	$\Delta_{trs}H$(II-I) / J g^{-1}	2σ / J g^{-1}	$\Delta_{trs}H_m$(II-I) / kJ mol^{-1}	2σ/ kJ mol^{-1}	Institution
0.5	149.98	423.13	1.28	12.34	0.66	3.26	0.17	UCT Prague
1	155.3	428.45	0.66	11.8	0.36	3.12	0.10	Innsbruck
2	160.00	433.15	0.38	11.53	0.24	3.05	0.06	Innsbruck
5	166.86	440.01	0.08	11.16	0.16	2.95	0.04	UCL
10	175.53	448.68	0.26	10.26	0.34	2.71	0.08	Radboud
10	170.56	443.71	1.66	10.93	0.26	2.89	0.06	UCL

The data in Table 1.6.1 and the heat capacities (Section 1.8) were used for the calculation of the consensus estimate at 150 °C. Additional data associated with this paper can be found at https://github.com/ccdc-opensource/collaboration-bestcsp-experimental-data.

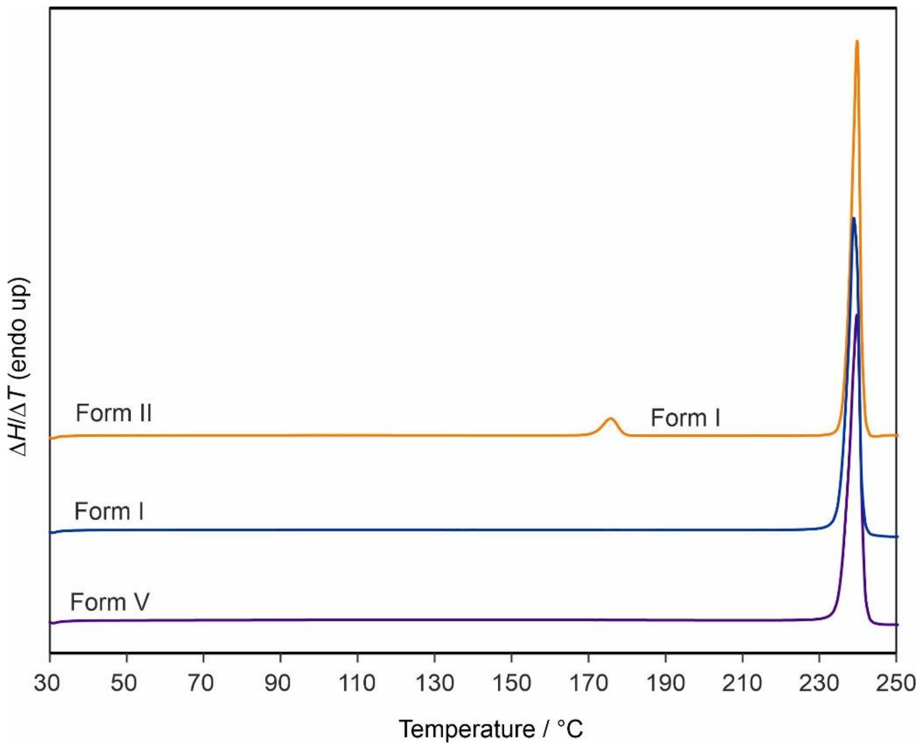

Figure 1.6.1. Characteristic DSC curves of SMZ II (orange), form I (blue) and form V (purple) at a heating rate of 10 °C min^{-1}. The transition event can be seen in the form II thermogram occurring at onset 170.9 °C.

1.6.1. UCL/Innsbruck

Contributed by Doris Braun and William Wood

Innsbruck: For Differential Scanning Calorimetry (DSC) experiments a 204 F1 Phoenix ASC instrument in conjunction with the NETZSCH-Proteus v7.1.0 software was employed (Netzsch, Germany). Precise sample weights were obtained using a XPR6UD5 ultramicrobalance (Mettler, Greifensee, Switzerland). Approximately 4 to 8 mg of the sample were used for each analysis at a heating rate of 1, 2, 5, and 10 K·min^{-1} with a N_2 purge of 20 mL min^{-1} and a protective purge of 40 mL min^{-1}. Hermetically sealed capsules were utilized for the experiments, with a minimum of five measurements performed. The calorimeter was calibrated for temperature using cyclohexane (melting point = −87.0 °C, 99.9%, Netzsch standard), mercury (−38.8 °C, 99.99%, Netzsch standard), benzophenone (48.0 °C, Mettler calibration standard), indium (156.6 °C, 99.999%, Netzsch standard), and caffeine (236.2 °C, Mettler calibration standard). Enthalpy calibration was performed using cyclohexane (79.4 J g^{-1}, 99.9%, Netzsch standard), mercury (11.4 J g^{-1}, 99.99%, Netzsch standard), tin (60.5 J g^{-1}, 99.999%, Netzsch standard), bismuth (53.1 J g^{-1}, 99.9995%, Netzsch standard), and indium

(28.45 J g^{-1}, 99.999%, Netzsch standard).

UCL: William Wood used a Mettler AT261 Delta Range balance and the same procedure as Innsbruck whilst visiting during a Short-Term Scientific Mission (STSM) funded by BEST-CSP.

1.6.2. UCT Prague

Contributed by UCT Prague: Jiří Šnajdr, Vojtěch Štejfa, Michal Fulem

DSC measurements were performed on a TA Discovery DSC 2500 (TA Instruments, New Castle, DE, United States) with the intracooler unit set to 183 K. The temperature and heat flow scales of the calorimeter were periodically calibrated with onset temperatures and fusion enthalpies of seven reference materials as described in Pouzar et al.[1] Three replicates were made for each calibrant and each tested heating rate (0.5, 2, 5, and 10 K min^{-1}). Approximately 4 to 8 mg of sample was loaded into a hermetic 40 µl aluminium pan and weighed with a precision of 0.01 mg on a Denver Instrument TB215D balance. An identical empty pan was used as reference. The purge gas was N_2 (SIAD, purity 4.0), at a flow rate of 50 cm^3 min^{-1}. The instrument control and data treatment procedures were carried out with the TRIOS 5.5.1.5 software.

References

[1] Pouzar, V.; Štejfa, V.; Fulem, M.; Růžička, K. Recommended Sublimation Pressures and Enthalpies for Biphenyl and *trans*-Stilbene. *J. Phys. Chem. Ref. Data* **2025**, *54* (1), 013101. DOI: 10.1063/5.0245848.

1.6.3. Radboud University

Contributed by Erik de Ronde

Differential Scanning Calorimetry (DSC) measurements were performed on a Mettler Toledo DSC 822e with a Julabo FT900 intercooler unit. There is no indication temperature set. Cooling happens with continuous cooling of a cooling finger that is mounted to the calorimeter cel. Without heating the cell drops to approximately 200 K. A heating rate of 10 K·min^{-1} was used. The DSC was calibrated prior to the measurement. The apparatus was calibrated at 10 K·min^{-1} with Indium (ME-119442 T_{fus} = 429.99 K, ΔH_{fus}= 28.56 J·g-1) and Zinc (ME-199441 T_{fus} = 692.34 K, ΔH_{fus} = 108.52 J·g-1). Approximately 3 to 6 mg of sample was loaded into a 40 µl Aluminum pan and weighed with a precision of 0.01 mg on a Mettler AX105 DeltaRange microbalance. The crucible was sealed with a lid and placed in the calorimeter cell. An identical

empty pan was used as a reference. The purge gas was Nitrogen, *5.0 Linde Gas 99.999%*, at a flow rate of approximately 70 cm^3 min^{-1}. The Nitrogen flow was controlled by an external gas flowmeter. The instrument control and data treatment procedures were carried out with the Mettler Toledo STARe V. 19 software, the tau-lag adjustment was not used.

1.7. Method of consensus estimate for enthalpy difference between forms I and II from DSC data

Contributed by Jonas Nyman

1.7.1. Introduction

The BEST-CSP COST Action collects new measurements and previously published data on various physical properties of molecular crystals. It is well-known that measurements on molecular crystals performed in different laboratories tend to quantitatively disagree because of systematic errors and differences in how measurements are performed, varying sample purity and other factors. Confidence intervals do not overlap as often as expected. This leads to confusion and questions regarding reproducibility and how measurements should best be performed. It also leads to mistrust, as scientists tend to not believe results from other laboratories since they appear to be irreproducible. It has also led to problems in computational chemistry, where the lack of accurate benchmarking data has made it difficult to assess the performance of density functionals and other methods used for crystal structure prediction.
The COST Action aims to address these issues in several ways.

- We collect data from several laboratories.
- Protocols are established for how measurements should be performed.
- The experimental results are compared to highly accurate computational results.
- The collected data is curated and consensus means are calculated.

When collecting data from several different laboratories, it is important to carefully consider what statistical method should be used for treating the data. Often, the raw data is not available and only a mean value and (hopefully) some kind of uncertainty or error bar is given. These error bars may have been calculated in different ways, they may be (±) a single standard deviation, two standard deviations, a so-called expanded standard deviation, a standard error of the mean, or a confidence interval. Often, the number of measurements is not given.
To pool the data by simply taking the arithmetic mean between studies does not consider the different variance of each study. It is common to instead use a weighted average where the weight is the inverse of that lab’s variance. But this also does not fully consider the situation we have, where we expect that there are also systematic errors and differences between labs.

We want a simple statistical method that allows us to calculate a consensus mean in a situation where we expect to have a spread both within and between studies.

1.7.2. The Random Effects Model

A suitable method that mimics the real-world situation with several labs, all doing their own measurements on different instruments and different samples is provided by the random effects model. The method is commonly applied to meta analyses of clinical trials where the effect of some medical intervention or treatment is evaluated. The included studies perhaps all use the same drug, but may differ in dosage or in how the treatment is given to the patients. Figure 1.7.1 schematically shows the expected outcome of some arbitrary measurement in three laboratories. Each lab has it's own sample, and observes different mean values and variances. There is a between-laboratory variance due to differences in experimental protocols, instrumentation, calibration and other confounding factors. It is this inter-laboratory spread that is treated as an additional random effect.

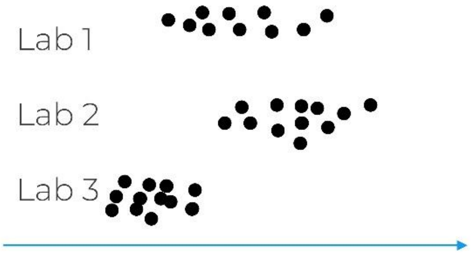

Figure 1.7.1. Schematic representation of measurement data from three laboratories. Each laboratory has measured the same physical quantity on some scale, and each laboratory has a spread or uncertainty in their results. There is also a spread between laboratories.

The unbiased sample variance for laboratory *i* with data points $1\cdots j$ can be written

$$s_i^2 = \frac{1}{n-1}\sum_j (x_j - \bar{x}_i)^2 \tag{1}$$

We use this as an estimate for the true population variance σ_i^2. It is important to note that several data points are needed for this estimate to be accurate, two or three data points are not enough. A commonly used statistical method for this situation is to apply the DerSimonianLaird random effects model[1]. The model is the simplest possible, or most parsimonious, in that it makes the fewest assumptions and contains as few parameters as possible, while being able to model the real-world situation. The model contains a single parameter for the inter-laboratory variance, τ^2. Figure 1.7.2 shows how we assume that the samples from each laboratory are normally

distributed, and have their own mean values and standard deviations. We consider all measurements to be for one and the same underlying physically real value μ, which we do not know, but wish to estimate.

In Figure 1.7.3, we show how the systematic differences between the studies are also assumed to follow a normal distribution that has variance τ^2. The original DerSimonianLaird method had an approximate method for calculating τ^2 that has since largely been replaced by better methods. The perhaps most common now is an iterative algorithm introduced by Paule and Mandel[2] and further studied and refined by Kacker[3] and DerSimonian and Kacker[4]. We use the method as described in this last study. This means that there is no single equation for τ^2, but it is calculated iteratively until the model parameters become self-consistent.

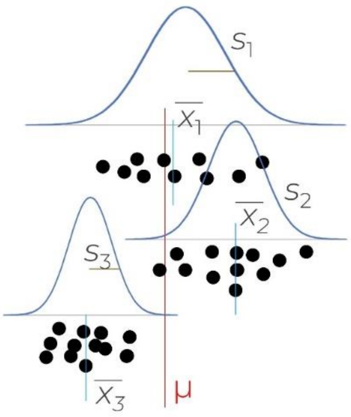

Figure 1.7.2. The data from each laboratory can be assumed to be normally distributed, but each laboratory has a different mean value $\bar{x}_i$ and standard deviation s_i. All measurements are for one and the same physical property that has one true value, μ.

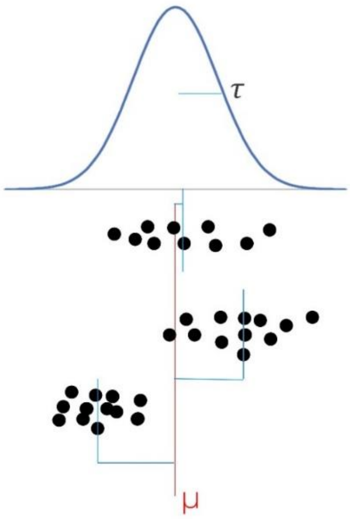

Figure 1.7.3. We assume that the inter-laboratory spread in mean values is also normally distributed. It then becomes possible to estimate μ as a weighted average that depends on both the intra- and

interlaboratory variances. Note that μ is *not* the centre of the normal distribution at the top of this figure, it just happens to look that way in this sketch.

The random effects estimate for the true value μ is a weighted average

$$\hat{\mu} = \sum_i w_i \bar{x}_i \tag{2}$$

Where the relative weight of each laboratory is:

$$w_i = \frac{(s_i^2 + \tau^2)^{-1}}{\sum_i (s_i^2 + \tau^2)^{-1}} \tag{3}$$

A 95% confidence interval for the random effects mean is calculated from the standard deviation of the random effects estimate s_{RE} and the 97.5% quantile for the standard normal distribution, *i.e.* $z_p = 1.96$.

$$s_{\mathrm{RE}} = \sqrt{\frac{1}{\sum_i (s_i^2 + \tau^2)^{-1}}} \tag{4}$$

$$\mathrm{CI}_\mu = \hat{\mu} \pm z_p s_{\mathrm{RE}} \tag{5}$$

The last expression should be understood as follows; if we were to repeat all the experiments many times and refit the statistical model on new data again and again, this confidence interval will contain the true value μ approximately 95% of the times.

Conclusion

A statistical method for treating the data within the COST Action has been presented. It is suggested that this method is used whenever possible. The method does, however, require a sufficiently large number of data points, or the variances and the size of the final confidence interval may be unreliable.

The script for doing these calculations was taken from the BEST-CSP github (https://github.com/ccdc-opensource/collaboration-bestcsp-experimental-data/blob/main/stats.py) and used on the DSC data.

1.7.3. Error estimate and propagation for enthalpy difference at STP

This was done in the spirit that experiments should be designed to minimize the errors, but the error estimate should be conservative to ensure that it encompasses the experimental value. The error on the DSC measured enthalpy of transition was taken as the largest error over all the measured and consensus values in order to overestimate the potential error from using the enthalpy from the fitted enthalpy curve. This way we take the error read of the curve within the

range of the measured enthalpies of transition to be the largest error.

The error of the heat capacity measurement was taken as an expert judgment of 20% of the thermal correction applied to the enthalpy in the measured range using the Cp values.

The propagated error of the STP value is thus:

$$\sigma(\Delta H(25°\mathrm{C})) = \sqrt{(0.17)^2 + (0.12)^2} \; = \; 0.21 \text{ kJ mol}^{-1}$$

References

[1] R. DerSimonian and N. Laird. Meta-analysis in clinical trials. *Controlled clinical trials*, 7 (3):177–188, 1986.

[2] R.C. Paule and J. Mandel. Consensus values, regressions, and weighting factors. *Journal of research of the National Institute of Standards and Technology*, 94(3):197, 1989.

R. Seabold and J. Perktold. Statsmodels: Econometric and statistical modeling with python. In *9th Python in Science Conference*, 2010.

[3] R. N. Kacker. Combining information from interlaboratory evaluations using a random effects model. *Metrologia*, 41(3):132, 2004.

[4] R. DerSimonian and R. Kacker. Random-effects model for meta-analysis of clinical trials: an update. *Contemporary clinical trials*, 28(2):105–114, 2007.

[5] S. Seabold and J. Perktold. Statsmodels: Econometric and statistical modeling with python. In 9th Python in Science Conference, 2010.

.

1.8. Heat capacity measurements

Contributed by UCT Prague: Jiří Šnajdr, Vojtěch Štejfa, Michal Fulem

The description of the samples used for the heat capacity measurements is given in Table 1.8.1. Sample purities were determined using gas-liquid chromatography; application of the van't Hoff method was impossible because of abrupt decomposition upon melting. Form I was used as received. Form II was prepared according to the BEST-CSP recipe (based on Pallipurath et al.[1]) by suspending form I in an acetonitrile–water mixture (80 g of acetonitrile, 20 g of water, and 2 g of sulfamerazine). Suspension was kept at 293 K and sporadically stirred for two weeks, filtered, left for a day to evaporate the remaining solvent and dried by vacuum (~80 Pa) at room temperature for another day.

X-ray powder diffraction (XRPD) was used to verify the crystal structures. The XRPD analysis was performed using a *θ*-*θ* powder diffractometer X'Pert[3] Powder from PANalytical in Bragg-Brentano para-focusing geometry using wavelength CuKα radiation (λ = 1.5418 Å, U = 40 kV,

I = 30 mA). The samples were scanned at temperature 298.15 ± 3 K in the range of $2\theta = 5°$ to 50° with a step size of $2\theta = 0.039$ and 0.7 s for each step. The diffractograms were analyzed with the software HighScore Plus in combination with annually updated powder diffraction databases PDF4+ and PDF4/Organics.

Table 1.8.1 Description of samples used for heat capacity measurements.

Compound	Form	Supplier	CSD ref. code [a]	Purity by supplier [b]	Purification	Final Purity [c]
Sulfamerazine	I	Aldrich	SLFNMA02	0.999	-	1.0000
Sulfamerazine	II	Aldrich	SLFNMA01	0.999	recrystallized	1.0000

[a] Cambridge Structural Database reference code of the corresponding crystal structure.

[b] Mole fraction purity according to the supplier of batch WXBD7819V determined by titration with $NaNO_2$.

[c] Mole fraction purity determined by gas-liquid chromatography (chromatograph Hewlett–Packard 6890 equipped with a column HP-1, length 25 m, film thickness 0.52 µm, diameter 0.30 mm, and FID detector) at temperatures 353 K – 513 K. Average of two determinations.

Heat capacity measurements were performed using a Tian-Calvet calorimeter (SETARAM Microcalvet, Caluire, France) with the operating temperature range (235–355) K (Table 1.8.2). The calorimeter calibration and mode of operation were described previously,[2] therefore only essential information is provided. The continuous heating method[3] was applied in connection with a three-step methodology (identical measurements performed with the sample, the reference material, and empty cell (so-called blank experiment)). Synthetic sapphire, NIST standard reference material No. 720[4], was used as the reference material and loaded in amount to keep the extensive heat capacity close to that of the compound. The combined expanded uncertainty ($k = 2$, 0.95 level of confidence) of the heat capacity measurements using the calorimeter SETARAM Microcalvet was estimated to be $U_c\left(C_{p,\mathrm{m}}\right) = 0.006C_{p,\mathrm{m}}$ based on testing with four reference materials.[2]

Table 1.8.2 Heat capacity of Sulfamerazine polymorphs determined by SETARAM Microcalvet (in J K^{-1} mol^{-1}) [a]

T / K	$C_{p,\mathrm{m}}$ (form I)	$C_{p,\mathrm{m}}$ (form II)	$100\left(C_{p,\mathrm{m}}^{\mathrm{II}}/C_{p,\mathrm{m}}^{\mathrm{I}}-1\right)$
240.0	245.8	247.6	0.73
245.0	250.1	252.2	0.82
250.0	254.4	256.8	0.93
255.0	258.6	261.3	1.05
260.0	262.6	265.9	1.22
265.0	267.4	270.4	1.12
270.0	271.5	274.6	1.13
275.0	275.7	278.9	1.16
280.0	280.0	283.7	1.30
285.0	284.3	288.4	1.41
290.0	288.5	292.8	1.44
295.0	292.8	297.2	1.46
300.0	297.1	301.5	1.48
305.0	301.3	305.9	1.50
310.0	305.6	310.3	1.52
315.0	309.9	314.7	1.53
320.0	314.2	319.0	1.51
325.0	318.5	323.3	1.49
330.0	322.7	327.6	1.49
335.0	326.9	331.9	1.52
340.0	331.1	336.3	1.53
345.0	335.4	340.8	1.58
350.0	339.6	345.2	1.63

[a] At a pressure of 100 ± 10 kPa. Standard uncertainty in temperature is $u(T)$ = 0.05 K, and the combined expanded uncertainty of the heat capacity is $U_{\mathrm{c}}\left(C_{p,\mathrm{m}}\right) = 0.006C_{p,\mathrm{m}}$ (k = 2, 0.95 level of confidence).[2]

Heat capacity measurements were further extended to 460 K using DSC 8500 (PerkinElmer, Shelton, Connecticut, US), a double furnace power-compensated DSC (Table 1.8.3). The temperature increment method was used with a step and heating rate of 5 K and 5 K min^{-1}, respectively and evaluated in the three-step methodology. Due to a higher uncertainty, the results obtained by the DSC 8500 were adjusted to agree with those determined by the more accurate SETARAM Microcalvet calorimeter in the overlapping temperature interval following a common practice.[5] After this correction, the combined expanded uncertainty (k = 2, 0.95 level of confidence) of the reported heat capacity data is estimated to be $U_{\mathrm{c}}\left(C_{p,\mathrm{m}}\right) = 0.02C_{p,\mathrm{m}}$. A detailed description of the measurement procedure, data evaluation, and calibration results were presented previously.[6]

Table 1.8.3 Heat capacity of Sulfamerazine polymorphs determined by PE8500 (in J K^{-1} mol^{-1}) [a]

T / K	$C_{p,\mathrm{m}}$ (form I)	$C_{p,\mathrm{m}}$ (form II)	$100\left(C_{p,\mathrm{m}}^{\mathrm{II}}/C_{p,\mathrm{m}}^{\mathrm{I}}-1\right)$
305.4	301.4	305.9	1.48
310.4	306.1	310.5	1.41
315.4	310.3	315.0	1.47
320.4	314.9	319.7	1.50
325.3	319.1	324.0	1.51
330.3	323.0	328.0	1.52
335.3	327.3	332.4	1.55
340.3	331.4	336.8	1.62
345.3	335.3	340.9	1.63
350.3	339.2	345.4	1.77
355.2	343.3	349.2	1.68
360.2	347.4	353.6	1.74
365.2	351.5	357.3	1.63
370.2	355.3	361.7	1.78
375.2	359.4	366.1	1.84
380.1	363.3	370.7	2.01
385.1	366.8	375.3	2.26
390.1	371.0	379.7	2.31
395.1	374.8	384.3	2.47
400.1	378.0	388.4	2.67
405.1	382.2	392.2	2.56
410.0	386.0	396.4	2.62
415.0	389.6	400.4	2.69
420.0	393.0	403.3	2.56
425.0	396.5		
430.0	400.2		
435.0	405.2		
439.9	408.4		
444.9	412.3		
449.9	415.3		
454.9	418.5		
459.8	421.3		

[a] At a pressure of 100 ± 10 kPa. Presented data were scaled by 1.003 and 0.993 for form I and form II, respectively, to obtain better agreement with the more accurate Tian-Calvet calorimeter. Standard uncertainty in temperature is $u(T) = 0.1$ K, and the combined expanded uncertainty of the heat capacity after the scaling is $U_c\left(C_{p,\mathrm{m}}\right) = 0.02C_{p,\mathrm{m}}$ ($k = 2$, 0.95 level of confidence).[6]

References

[1] A. R. Pallipurath, J. M. Skelton, M. R. Warren, N. Kamali, P. McArdle, A. Erxleben, Sulfamerazine: Understanding the Influence of Slip Planes in the Polymorphic Phase Transformation through X-Ray Crystallographic Studies and ab Initio Lattice Dynamics. Mol Pharm **12**, 3735-3748 (2015). doi:10.1021/acs.molpharmaceut.5b00504.

[2] V. Štejfa, O. Vojtíšková, V. Pokorný, J. Rohlíček, K. Růžička, M. Fulem, Heat capacities of active pharmaceutical ingredients nifedipine, griseofulvin, probucol and 5,5-

diphenylhydantoin. J. Therm. Anal. Calorim. **149**, 6179-6193 (2024). doi:10.1007/s10973-024-13220-6.

[3] G. W. H. Höhne, H.-J. Flammersheim, W. Hemminger, *Differential scanning calorimetry*. (Springer, Berlin, Germany, 2003).

[4] G. A. Uriano, National Bureau of Standards Certificate: Standard Reference Material 720 Synthetic Sapphire (α-Al_2O_3). (1982). https://tsapps.nist.gov/srmext/certificates/720.pdf

[5] Y. T. Suzuki, Y. Yamamura, M. Sumita, S. Yasuzuka, K. Saito, Neat liquid consisting of hydrogen-bonded tetramers: dicyclohexylmethanol. J Phys Chem B **113**, 10077-10080 (2009). doi:10.1021/jp9048764.

[6] V. Pokorný, C. Červinka, V. Štejfa, J. Havlín, K. Růžička, M. Fulem, Heat Capacities of L-Alanine, L-Valine, L-Isoleucine, and L-Leucine: Experimental and Computational Study. J. Chem. Eng. Data **65**, 1833-1849 (2020). doi:10.1021/acs.jced.9b01086.

1.9. Evaluations of Sample Purity

Contributed by Dejan-Krešimir Bučar

The presence of Form V in the Form I commercial sample used at UCL was *estimated* through a Rietveld refinement using data collected on a *Stoe Stadi-P* diffractometer. The instrument was equipped with a Cu anode, a Ge<111> monochromator and a *Dectris Mythen2 1K* detector. The data was collected using Cu $K\alpha_1$ X-rays in transmission mode. The sample, placed in a foil sample holder between two cellulose triacetate foils, was used as received (without being ground and sieved) to avoid the potential induction of any phase transformations. The refinement was accomplished using the *Rietica* program (http://www.rietica.org). The data was treated without considering the effects of X-ray absorption and preferred particle orientation. The amount of Form V in the Form I commercial sample was found to be *approximately* 20% by weight (Figure 1.9.1).

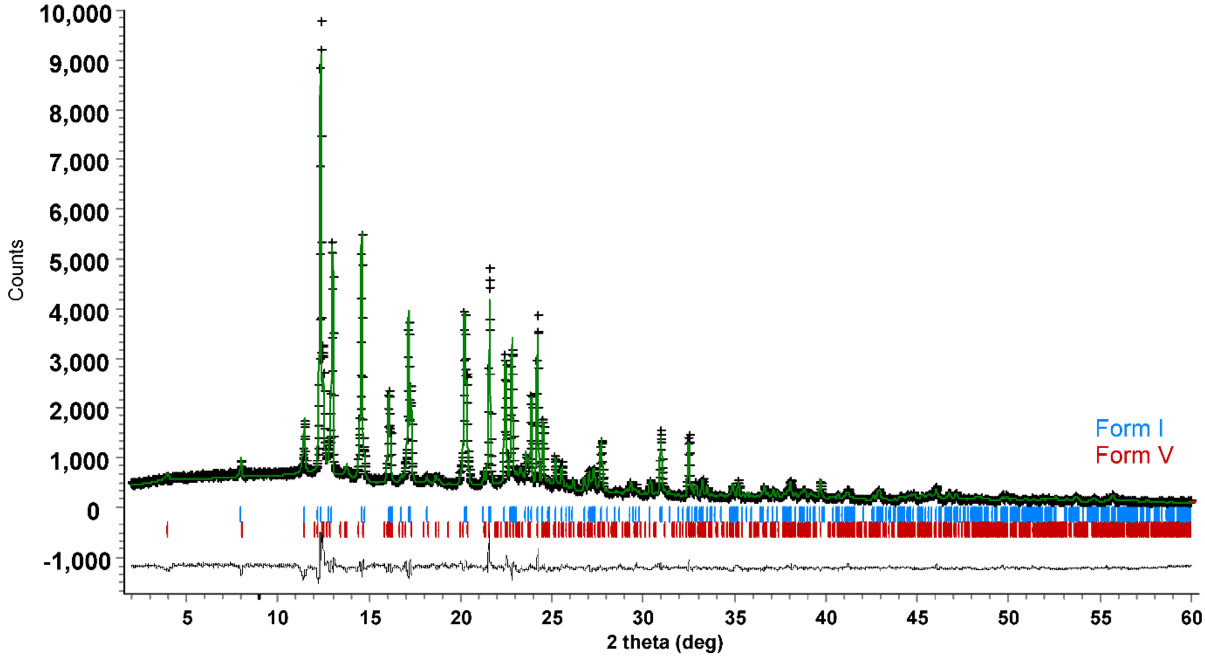

Figure 1.9.1. Rietveld refinement plot of the commercial Form I sample (Toku-E, batch S033-01). The calculated plot is shown in green and the difference plot in grey. The peak positions for Form I and V are shown in blue and red, respectively.

Solution-based ^{1}H NMR spectra were also acquired for samples used at UCL (Form I) and the University of Radboud (Forms I and II). Peak integration in all spectra, together with comparisons to the intensities of the ^{13}C satellite signals, suggested that the samples are approximately 99% pure. The impurities do not appear to be chemically related to sulfamerazine, as evidenced by the absence of impurity signals in the aromatic regions. All impurity signals appear in the aliphatic region at lower ppm values (Figure 1.9.2). The data was collected using a *Bruker Avance Neo 700* instrument. The samples were prepared using DMSO-d6 as solvent and equal amounts of sulfamerazine.

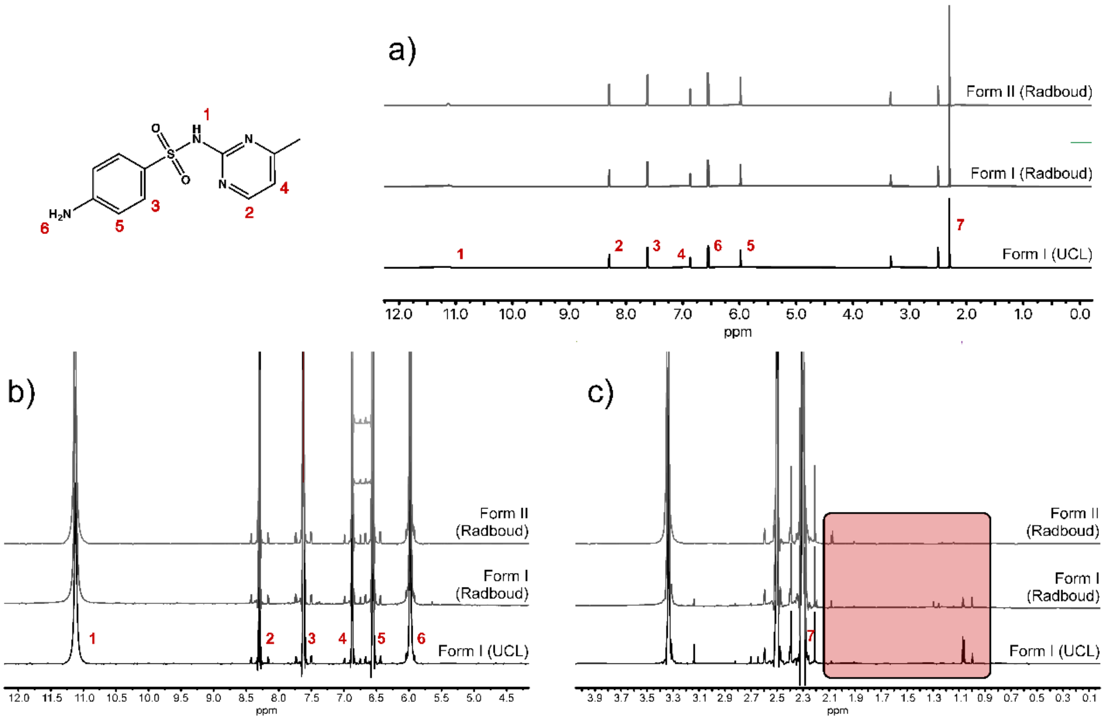

Figure 1.9.2. ^{1}H NMR spectra of Forms I and II used at UCL and University of Radboud: a) full ppm range, b) 4-12 pp range, and c) 0-4 ppm range.

1.10. Solubility by Clear Point Measurements

The solubility determination was carried out by the clear-point method, measured using the multi-reactor crystallizer Crystal16 (Technobis Crystallization Systems, Alkmaar, Netherlands). Mixtures with known concentration were prepared by weighing either Form I, II or V of SMZ into 1.5 ml HPLC vials and adding 1 mL of solvent MeCN:$H_2$0 (80:20, v:v). Samples were weighed with a UM3 ultramicrobalanc (Mettler, Greifensee, Switzerland). The mixtures were then placed in the Crystal16 at 5 °C and stirred at 1200 rpm and heated to 70 °C at 0.2 °C min^{-1}. The clear-point (dissolution temperature) is measured by a turbidity measurement and recorded by the Crystal16 software (see Table 1.10.1, Table 1.10.2 and Table 1.10.3).

The known concentrations are then plotted against their dissolution temperatures to generate the solubility curves. To make transitions between polymorphs clearer, the concentrations are plotted as the natural logarithms of the concentration values in mg ml^{-1}. The concentrations are not converted to their mole fraction as this is not usual in the pharmaceutical industry. We did not attempt to plot the van't Hoff solubility plots as the solubilities are measured by dissolution

and thus are not equilibrium values and should not be used to derive thermodynamic properties.

In parallel with the solubility experiments, slurry experiments were performed in the Crystal16 under identical conditions. Samples I, II, and V were withdrawn at approximately 10, 30, 40, 50, and 65 °C and analyzed by PXRD to confirm that no phase transformation had occurred during the experiment. This consideration guided the choice of a heating rate of 0.2 °C min^{-1}.

Table 1.10.1. SMZ form I solubility by clear-point data

Weight / mg	Volume / mL	Concentration / mg ml^{-1}	Clear-point / °C
6.03	1	6.78	7.9
8.24	1	8.37	13.3
10.15	1	10.15	20.2
12.21	1	12.21	25.9
13.82	1	13.82	29.5
16.18	1	16.18	34.3
20.00	1	20.00	40.6
25.27	1	25.27	47.9
29.94	1	29.94	52.4
29.94	1	29.94	52.3
32.08	1	32.08	54.6
32.08	1	32.08	54.4
32.76	1	32.76	55.1
32.76	1	32.76	55.2
36.92	1	36.92	59.2
36.92	1	36.92	58.6
39.38	1	39.38	60.9
39.38	1	39.38	60.9
41.89	1	41.89	62.8
41.89	1	41.89	62.7
48.56	1	48.56	67.5
48.56	1	48.56	67.2
51.39	1	51.39	69.1
51.39	1	51.39	69.3

Table 1.10.2. SMZ form II solubility by clear-point data

Weight / mg	Volume / mL	Concentration / mg mL^{-1}	Clear-point / degC
6.03	1	6.03	10.9
8.24	1	8.24	18.3
10.89	1	10.89	24.5
12.78	1	12.78	29.3
13.62	1	13.62	30.5
15.78	1	15.78	34.3
20.75	1	20.75	42.6
25.29	1	25.29	47.9
31.83	1	31.83	54.4
35.85	1	35.85	57.4
38.74	1	38.74	59.3
48.18	1	48.18	65.6
32.25	1	32.25	54.5
32.73	1	32.73	54.6
38.35	1	38.35	59.4
46.05	1	46.05	64.4
52.83	1	52.83	68.1

Table 1.10.3. SMZ form V solubility by clear-point data

Weight / mg	Volume / mL	Concentration / mg mL^{-1}	Clear-point / degC
6.19	1	6.19	8.1
7.50	1	7.50	12.7
10.68	1	10.68	22.1
11.62	1	11.62	25.6
14.99	1	14.99	33.2
18.39	1	18.39	38.7
20.43	1	20.43	42.1
23.54	1	23.54	46.3
29.41	1	29.41	52.4
35.46	1	35.46	58.2
40.10	1	39.10	61.3
42.76	1	42.76	64.1
49.66	1	48.66	68.2
44.68	1	43.71	65.1

2. Computational Details

Preliminary computational work on the SMZ polymorphs was carried out by a range of WG5 members, considering lattice energy differences at the meeting in Zagreb in April 2024 and the addition of some free energy calculations at the meeting in Warsaw in September 2024. The groups who submitted free energy calculations were encouraged to contribute to this paper. An

adaptation of the preliminary results discussed at these meetings is shown in Figure 2.1 and clearly demonstrated that there is a significant variation in the lattice energy with the method.

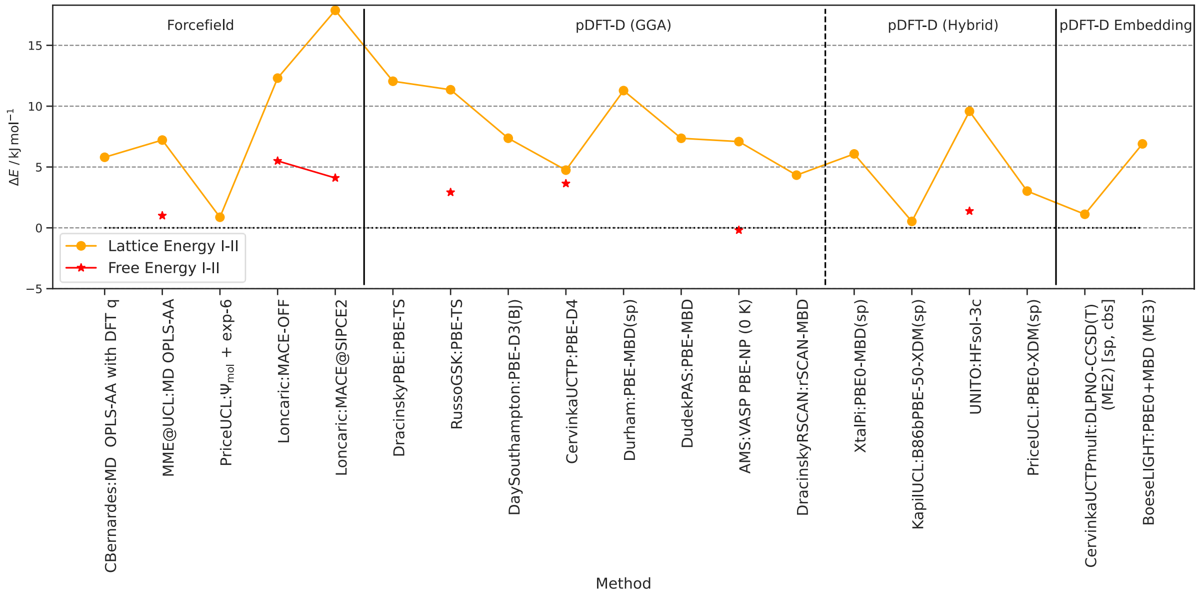

Figure 2.1. The lattice energies and free energy differences between form I and II of SMZ by method.

2.1. Bondlength variation in calculations.

Contributed by Pamela S. Whitfield

The solution of large molecular crystal structures from powder X-ray data can benefit greatly from the application of restraints on the bondlengths, but these must be as accurate as possible to avoid negatively biasing the result. Use of bond-length restraints has also been shown to have measurable benefits even with low-temperature single-crystal data in some circumstances.[1] Pamela Whitfield noted in solving room-temperature sulphonamide structures and examination of literature examples, that optimised sulphonamide structures appeared to exhibit significantly greater variance from observed values than is typical in the S-N and S=O bondlengths.[2] She developed a protocol for estimating these bondlengths by testing the sensitivity of the calculated bondlengths to computational method, including far more advanced functionals than are traditionally used in modelling organic crystal structures.

Fixed-cell structure optimizations were carried out on the 150K and 300 K crystal structures from Pallipurath et al.[3] for both Form-I and Form-II and the 150 K structure for form V using various functionals. CUDA-compiled Quantum Espresso 7.4.1[4,5] was compiled with Libxc 7.0.0.[6]. Libxc was itself compiled with the Fermi-hole curvature constraint disabled to improve the forces computed, and convergence with meta-GGA functionals such as SCAN.[7,8] Norm-conserving pseudopotentials were used throughout with an energy cutoff of 60 Ry and

wavefunction cutoff of 240 Ry. Functional/pseudopotential/dispersion-correction combinations were those commonly found to be most effective in the literature and found by the author to perform well with this particular setup. Stringent PBE pseudopotentials were obtained from the PseudoDojo website.[9] SCAN pseudopotentials were those of Yao and Kanai.[10] Convergence of the SCAN optimizations was further improved by increasing the FFT grid density with 50% additional grid points in each unit-cell direction.[10] The rVV10 dispersion correction was introduced directly from the Libxc correlation function. Custom pseudopotentials were created for the B97-3c[11], BLYP and B86bPBE functionals using the atomic package in Quantum Espresso. As per normal practice in Quantum Espresso, computations with global hybrids such as B86bPBEX-25 (B86bPBEX with 25% exact exchange) used the corresponding GGA pseudopotentials. In the interests of efficiency, the global hybrids B3LYP and B86bPBEX-25 used the computed coordinates of the corresponding GGA calculations as inputs, the SCAN-based calculations using those from the B97-3c optimizations. The B3LYP and B86bPBEX-25 global hybrid calculations used a reduced Fock exchange kinetic energy cutoff of 90 and 60 Ry respectively, reduced from the default of 240 Ry, to fit within the available 32GB VRAM of the Quadro GV100 GPU. This has the additional benefit of greatly reducing computation time. Testing on smaller crystalline systems has shown this to have minimal impact on the resulting bond lengths but introduces uncertainties to the resulting energies rendering them questionable.

The ADPs of the crystal structures were used to correct the experimental bondlengths for the libration foreshortening using Platon.[12]

The results confirm that the PBE functional yields longer S-N and S=O bondlengths than those measured experimentally (BLYP and B86bPBE GGA functionals tested showed the same trend), while the level of agreement for the S-C bondlength is more typical. This can be attributed to the delocalisation error in the GGA functionals, including the commonly used PBE. The electron delocalization behaviour of sulphonamides is known to be sensitive to the nature of the adjacent groups.[13] Additionally, the hybridization of the sulphonamide nitrogen was shown to be extremely sensitive to the torsion angle.[14] This makes sulphonamides prime candidates for exhibiting significant delocalization error during DFT computation, a known and serious unsolved issue for DFT.[15,16] One consequence of the resulting overstabilization of delocalized electrons is excessively long bond lengths compared to observed values. Some S pseudopotentials available in online libraries lack an unoccupied 3d orbital in valence. Use of these yields even longer S-N and S=O bonds than those seen shown in Figure 2.1.1, consistent with the observations of Mayer[17] for hypervalent sulphur. The impact of delocalisation error is

known to be reduced in meta-GGAs such as SCAN, and global-hybrids such as B86bPBEX-25 (Bryenton et al., 2022). Eliminating it entirely involves even higher-level computations but are usually too computationally intensive for routine applications. The effect of this error in the bondlengths and the sensitivity to the torsion angles may affect the phonon frequencies.

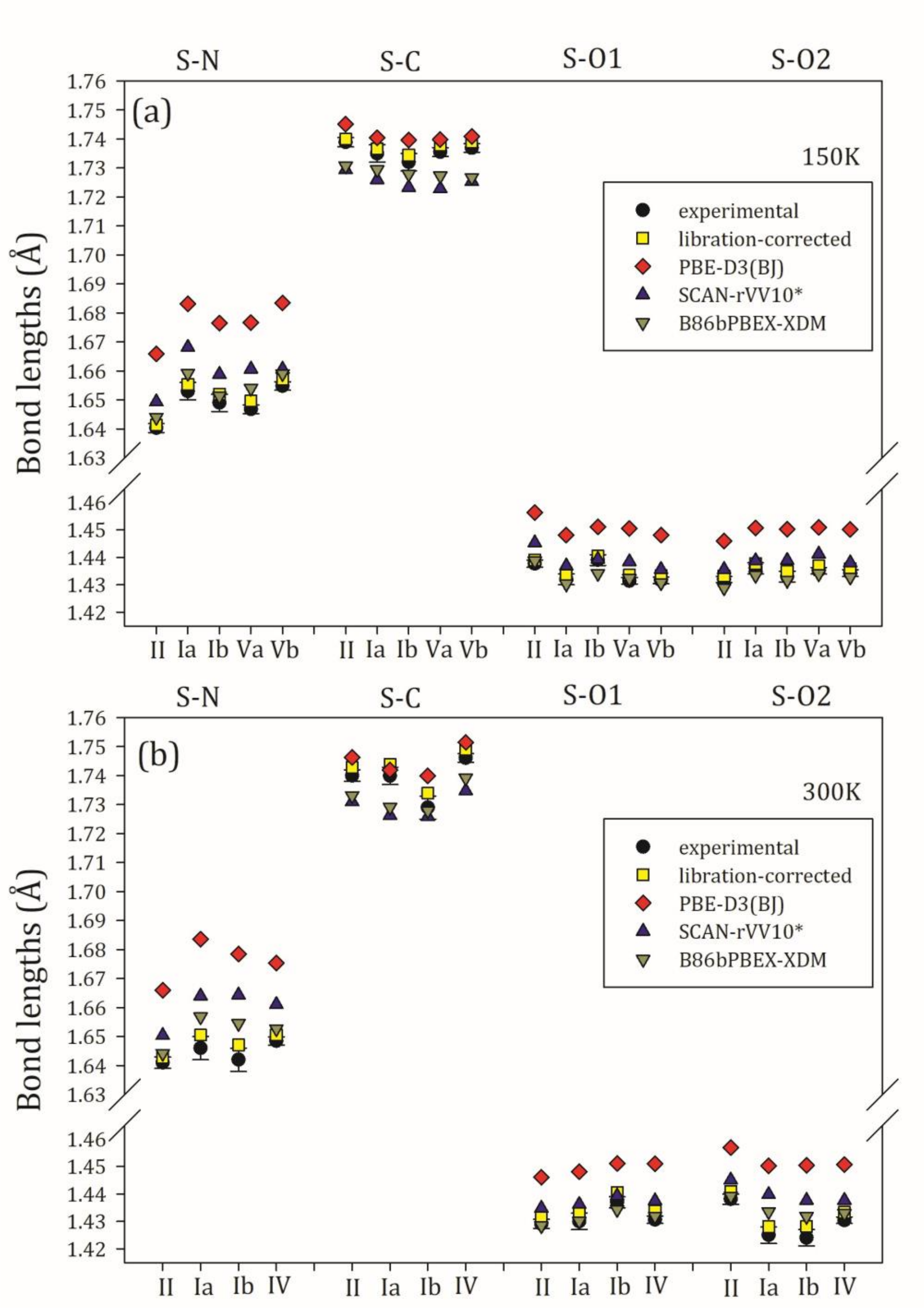

Figure 2.1.1. Sulphonamide bondlengths in sulfamerazine computed via by fixed-cell optimisations using typical GGA (PBE), meta-GGA (SCAN) and global hybrid (B86bPBEX-25) functionals. (a) 150 K structures of forms I, II, and V. (b) Room temperature structures of forms I, II and IV.

References

[1] Birger D., Breznikar R., Santarossa G., Whitfield P. & Moebitz H. (2025), "Benchmarking quantum chemical methods with X-ray structures via structure-specific restraints", IUCrJ, 12, 487

[2] Whitfield P.S. & Kaduk J. (2025). "Structure solution of sulphonamides from powder diffraction data", Presented at 18th Pharmaceutical Powder X-ray Diffraction Symposium, Cambridge UK, 6-9 May 2025

[3] Pallipurath A.R., Skelton J.M., Warren M.R., Kamali, N., McArdle P. & Erxleben A., (2015), "Sulfamerazine: Understanding the influence of slip planes in the polymorphic phase transformations through X-ray crystallographic studies and ab initio lattice dynamics", Molecular Pharmaceutics, 12, 3735-3748

[4] Giannozzi et al. (2017) "Advanced capabilities for materials modelling with QUANTUM ESPRESSO", J. Phys.: Condens. Matter, 29, 465901

[5] Giannozzi et al. (2020) "Quantum ESPRESSO towards the exascale", J. Chem. Phys., 152, 154105

[6] Lehtola et al. (2018) "Recent developments in Libxc – A comprehensive library of functionals for density functional theory", Software X, 7, 1

[7] Quantum Espresso 7.4.1 User Guide (2025), https://gitlab.com/QEF/q-e/-/blob/master/Doc/user_guide.tex

[8] VASP Wiki (2025), https://www.vasp.at/wiki/index.php/LTBOUNDLIBXC

[9] van Setten M.J., Giantomassi M., Bousquet E., Verstraete M.J., Hamann D.R., Gonze X., Rignanese G.-M. (2018), "The PseudoDojo: Training and grading a 85 element optimized norm-conserving pseudopotential table", Computer Physics Communications, 226, 39-54

[10] Yao Y. & Kanai Y. (2017), "Plane-wave pseudopotential implementation and performance of SCAN meta-GGA exchange-correlation functional for extended systems", J. Chem. Phys., 146, 224105

[11] Brandenburg J.G., Bannwarth C., Hansen A. & Grimme S. (2018), "B97-3c: A revised low-cost variant of the B97-D density functional method", J. Chem. Phys., 148, 064104

[12] Spek A. L. (2009). "Structure validation in chemical crystallography", Acta Cryst. D, 65, 148–155.

[13] Caine B.A., Bronzato M. & Popelier P.L.A. (2019), "Experiment stands corrected: accurate prediction of the aqueous pKa values of sulfonamide drugs using equilibrium bond lengths", Chem. Sci., 10, 6368-6381

[14] Breneman C.M. & Weber L.W. (1996). “Charge and energy redistribution in sulfonamides undergoing conformational changes. Hybridisation as a controlling influence over conformer stability, Can. J. Chem., 74, 1271-1282
[15] Broderick D.R. & Herbert J.M. (2024). “Delocalization error poisons the density-functional many-body expansion”, Chem. Sci., 15, 19893
[16] Bryenton K.R., Adeleke A.A., Dale S.G & Johnson E.R. (2022). “Delocalization error: The greatest outstanding challenge in density-functional theory”, WIREs Comput. Mol. Sci., 13, e1631
[17] Mayer I., (1987), “Bond orders and valences: Role of d-orbitals for hypervalent sulphur”, J. Molecular Structure (Theochem), 149, 81-89

2.2. Harmonic mode calculations

Summarized Sarah L. Price from the contributions of William P. Wood, Mattia Raimondo, Lorenzo Donà, Bartolomeo Civalleri, Ctirad Červinka, Reynaldo Geronia, Mihails Arhangelskis, Dzmitry Firaha, Luca Russo, Yizu Zhang, Zhuocen Yang, Qun Zeng, Guangxu Sun, Natalia Goncharova, Alexander List, Johannes Hoja and A. Daniel Boese.

The harmonic phonon model is very similar to the harmonic oscillator model for molecular vibrations, i.e. based on using the second derivatives of the potential energy surface as force-constants and the atomic masses. The q=0 phonons, which are those seen in the Infrared and Raman spectra of the crystals, roughly equate to treating the unit cell as a molecule. Indeed the higher energy crystal frequencies are usually a good match to the molecular frequencies, particularly for rigid molecules without hydrogen bonding. In such cases, there is often a significant gap in the spectrum between the molecular modes and the crystal modes determined by the weaker intermolecular interactions, with the traditional solid-state FT-IR spectrum, (e.g. Figure 4 m/s) being split into two ranges reflecting the molecular and the crystal modes. In the case of SMZ, the hydrogen bonding affects the highest frequency bond-stretching modes, and the molecular flexibility, particularly the low barrier torsions, mixing in with the crystal modes. Thus the frequencies are very dependent on the balancing of the intra-molecular potentials for bond stretching, bending and particularly the torsions, with the intermolecular forces, particularly in the well region where the exponentially repulsive terms are balanced with the damped dispersion around the van der Waals separation. Hence, it is expected that the frequencies will be very dependent on the type of potential energy surface being used, including details such as the basis set or plane-wave cutoff and any pseudopotentials modelling the core electron density.

A major difference between the crystal and the molecule calculations is the method of estimating the modes which do not fit in the unit cell, known as the phonon dispersion or $q \neq 0$ modes. This can be done by using bigger supercells, chosen to be a near cubic as possible, until the results converge. The method of non-diagonal supercells uses a series of small supercells to approximate a far larger near cubic supercell.[1] It must be noted that forms I and II of SMZ are a relatively rare example of the two polymorphs having similar size and shape unit cells (both orthorhombic with 8 molecules in the unit cell and a distinct small, medium and large cell length (Table 2.2.1), allowing for more cancellation of errors in the phonon calculations between the two polymorphs than is typically the case.

Most of the phonon calculations were carried out with codes originally written for inorganic solids, which is reflected in the default parameters governing the numerical accuracy of the calculations. All calculations used finite differences to calculate the second derivative, except RussoGSK who used linear response approach. Since the second derivatives are being calculated, the surface needs to be smooth, and hence the convergence of the energies (SCF convergence) as well as the optimization needs to be tight enough to avoid noise. Most of the parameters that were used are tighter than defaults, and some people reported that this was necessary to avoid getting imaginary frequencies. Phonon calculations as relying on second derivatives probe a different aspect of the potential energy surface than structure optimization (forces, which are zero at the minima) or energy calculations, and are many times more computationally expensive. Problems with being able to automate thermodynamic harmonic approximation calculations to converge the phonon dispersion and not get any imaginary frequencies are common but ignoring these in calculating the thermodynamic quantities by integration over the statistical mechanics formulae is wrong.

The computational expense of calculating the harmonic modes properly for many structures, as in a CSP study, means that methods of trying to produce cheaper calculations of the phonon density of states whilst retaining the accuracy of the thermodynamic quantities and avoiding imaginary frequencies is an area of active research.[2,3]

Table 2.2.1 The parameters used for the harmonic (HA) and quasi-harmonic (QHA) calculations.

Name	Type	Code	Functional	Disp.	Method for q≠0	Pseudopotential	electronic k-points	plane wave cutoff / eV	SCF convergence /eV	Optimisation convergence / e V Å^{-1}
PriceUCL	Harmonic	CASTEP	PBE	TS	non-diagonal supercells	ultrasoft on the fly	spacing 0.1 (1x1x1)	900	1.00E-12	0.001
RussoGSK	Harmonic	CASTEP(Materials Studio v2022)	PBE	TS	Linear Response	norm conserving on the fly	1x1x2 form I; 2x1x1 form II	925.2	1.00E-10	1.00E-05
XtalPi	Harmonic	VASP	PBE	TS	Supercells ≥15Å	PAW on the fly	spacing 0.05	620	1.00E-05	1.00E-03
Arhangelskis	Harmonic	CASTEP	PBE	D4	Unit cell only	ultrasoft on the fly	2x1x3 (form I)	800	1.00E-10	1.00E-02
TCG-UNITO	Harmonic	CRYSTAL23	HFsol-3c	D3	Supercells<br>1 x 1 x 2 (form I)<br>2 x 1 x 1 (form II)	GTOs	4x4x4 IBZ		2.72E-09	4.00E-03
@CervinkaG	Harmonic	VASP	PBE	D4	Supercells (> 10 Å)	hard PAW version PBE_6.4 in VASP	3x2x1 optimizations, 1x1x1 phonons	1000	1.00E-08	5.00E-05
@CervinkaG	QHA	DFTB+	DFTB3/3ob	D4	Supercells (> 10 Å)	3ob parametrization in DFTB+	3x2x1 optimizations, 1x1x1 phonons	n/a	2.70E-07	5.10E-05
Boese	QHA	MEmbed, FHI-aims, Phonopy	PBE0:PBE (multimer embedding ME3)	MBD	Supercells (≥ 12 Å)	None (numeric atom-centered basis)	2x1x3 form 1; 3x2x1 form II	None (light or tight settings within FHI-aims)	1.00E-06	5.00E-03
AMS	HA with QHA corrections	GRACE with FHI-aims	PBE, PBE0, MP2	NP, MBD-nl, D for MP2	supercell with a minimum lattice point distance of 8 Å	None (numerical atom-centered basis)	1x1x1 for both forms	None (light,tight,NAO-VCC-4Z)	5.20E-4	8.63E-03

References
[1] Nyman, J.; Pundyke, O. S.; Day, G. M. Accurate force fields and methods for modelling organic molecular crystals at finite temperatures. Physical Chemistry Chemical Physics 2016, 18 (23), 15828-15837. DOI: 10.1039/C6CP02261H.
[2] Cook, C.; Beran, G. Reduced-cost supercell approach for computing accurate phonon density of states in organic crystals. Journal of Chemical Physics 2020, 153 (22), Article. DOI: 10.1063/5.0032649.
[3] Ludík, J.; Kostková, V.; Kocian, Š.; Touš, P.; Štejfa, V.; Červinka, C. First-Principles Models of Polymorphism of Pharmaceuticals: Maximizing the Accuracy-to-Cost Ratio. Journal of Chemical Theory and Computation 2024, 20 (7), 2858-2870. DOI: 10.1021/acs.jctc.4c00099.

2.3. Arhangelskis Group

Contributed by Mihails Arhangelskis

All calculations were performed in the plane-wave periodic DFT code CASTEP 23.1.[1] Prior to geometry optimization, the C-H bond lengths in the experimental crystal structures of the polymorphs of SMZ (CSD SLFNMA01 and SLFNMA02) were adjusted to the average neutron diffraction values, using the "Normalise hydrogens" function in Mercury.[2] The CIFs of the experimental structures were then converted to CASTEP input format using the code cif2cell.[3] The plane-wave DFT calculations were performed with PBE functional,[4] combined with Grimme D4 dispersion correction model.[5] The plane-wave basis set was truncated at 800 eV cutoff, and the CASTEP internal ultrasoft pseudopotentials were used. The electronic Brillouin zone was sampled with a Monkhorst-Pack[6] k-point grid spacing of 2πx0.05 Å-1.

The structure optimization and subsequent phonon calculations were performed in four steps:

1) Initially, the structures were optimized with respect to atom positions and unit cell parameters, subject to the space group symmetry constraints. At that stage the following convergence criteria were used: energy tolerance 2x10-5 eV/atom; force tolerance 0.05 eV/Å; maximum atom displacement 10-3 Å; residual stress 0.05 GPa.

2) In the next optimization step, the structure was optimized to a tighter force tolerance of 0.01 eV/Å, but this time keeping the unit cell parameters fixed at the values obtained after the optimization from step 1. At this point the FFT grid and fine FFT grid scales were changed from their default values to 2 and 3, respectively. Optimisation with tighter force tolerance is necessary to avoid occurrence of the imaginary frequencies during the subsequent phonon calculations, yet optimizing with such a tight force tolerance with a variable unit cell tends to

take a large number of iterations to converge. Hence it was decided to converge unit cell parameters and atomic forces in a two-step process described above.

3) Finally, the structures were re-optimised with the conversion to odd-numbered electronic k-point grid, in order to ensure presence of the Γ (0, 0, 0) k-point within the electronic k-points. All the other settings were kept as in step 2, described above.

4) The structures optimised in step 3) were subjected to phonon finite displacement calculations with the phonon fine method set to the supercell approach. The finite displacement amplitude was set to 5.292x10-3 Å, reciprocal space acoustic sum rule was applied to the phonon frequencies and the thermodynamic functions were evaluated up to 600 K temperature. The phonon q-point grid was constructed with the same set of points as used for the electronic k-point grid. The calculated electronic and vibrational zero-point energies (ZPE) for both polymorphs of SFZ are given in Table 2.3.1, while the thermodynamic functions as a function of temperature are listed in Table 2.3.2.

Table 2.3.1. Electronic and zero-point energies.

Structure	Electronic energy per SFZ molecule / eV	ZPE per SFZ molecule / eV
Form I	-4217.3064	6.1162
Form II	-4217.3647	6.1444

Table 2.3.2. Thermodynamic functions as a function of temperature.

T / K	Form I				Form II			
	Vibrational enthalpy / eV	Vibrational free energy / eV	S / J mol^{-1} K^{-1}	C_v / J mol^{-1} K^{-1}	Vibrational enthalpy / eV	Vibrational free energy / eV	S / J mol^{-1} K^{-1}	C_v / J mol^{-1} K^{-1}
1	6.1162	6.1162	0.0000	0.0001	6.1445	6.1445	0.0000	0.0000
5	6.1162	6.1162	0.2688	0.8843	6.1445	6.1445	0.0854	0.3671
10	6.1163	6.1161	2.0256	5.1929	6.1446	6.1445	0.9959	2.9523
15	6.1168	6.1160	5.2804	11.4620	6.1448	6.1444	3.0049	7.5398
20	6.1175	6.1156	9.5246	18.4578	6.1454	6.1441	5.9664	13.5290
25	6.1187	6.1150	14.4243	25.7508	6.1462	6.1437	9.7139	20.4226
30	6.1202	6.1141	19.7660	33.0506	6.1475	6.1431	14.0796	27.7228
35	6.1221	6.1129	25.3983	40.1635	6.1491	6.1423	18.9055	35.0634

T / K	Form I				Form II			
	Vibrational enthalpy / eV	Vibrational free energy / eV	S / J mol^{-1} K^{-1}	C_v / J mol^{-1} K^{-1}	Vibrational enthalpy / eV	Vibrational free energy / eV	S / J mol^{-1} K^{-1}	C_v / J mol^{-1} K^{-1}
40	6.1244	6.1114	31.2103	46.9850	6.1511	6.1411	24.0583	42.2329
45	6.1270	6.1097	37.1223	53.4745	6.1535	6.1398	29.4338	49.1290
50	6.1299	6.1076	43.0779	59.6325	6.1562	6.1381	34.9536	55.7149
55	6.1332	6.1052	49.0381	65.4836	6.1593	6.1361	40.5605	61.9903
60	6.1367	6.1025	54.9770	71.0641	6.1626	6.1339	46.2129	67.9746
65	6.1405	6.0995	60.8779	76.4143	6.1663	6.1313	51.8814	73.6968
70	6.1446	6.0962	66.7306	81.5723	6.1703	6.1285	57.5453	79.1884
75	6.1490	6.0926	72.5299	86.5729	6.1745	6.1254	63.1901	84.4811
80	6.1536	6.0887	78.2734	91.4453	6.1790	6.1220	68.8069	89.6041
85	6.1584	6.0845	83.9608	96.2135	6.1838	6.1182	74.3891	94.5836
90	6.1636	6.0800	89.5930	100.8966	6.1888	6.1142	79.9335	99.4421
91	6.1646	6.0790	90.7130	101.8244	6.1898	6.1134	81.0376	100.4011
92	6.1657	6.0781	91.8310	102.7495	6.1909	6.1126	82.1401	101.3563
93	6.1667	6.0771	92.9468	103.6721	6.1919	6.1117	83.2410	102.3075
94	6.1678	6.0762	94.0605	104.5923	6.1930	6.1108	84.3403	103.2553
95	6.1689	6.0752	95.1721	105.5100	6.1941	6.1100	85.4379	104.1994
100	6.1745	6.0701	100.7001	110.0655	6.1996	6.1054	90.9018	108.8719
105	6.1803	6.0648	106.1795	114.5725	6.2054	6.1005	96.3253	113.4736
110	6.1864	6.0591	111.6126	119.0385	6.2114	6.0954	101.7091	118.0163
111	6.1876	6.0580	112.6939	119.9273	6.2126	6.0944	102.7811	118.9185
112	6.1888	6.0568	113.7735	120.8148	6.2138	6.0933	103.8518	119.8190
113	6.1901	6.0556	114.8514	121.7009	6.2151	6.0922	104.9209	120.7176
114	6.1914	6.0544	115.9275	122.5856	6.2163	6.0911	105.9884	121.6145
115	6.1926	6.0532	117.0020	123.4693	6.2176	6.0900	107.0544	122.5096
120	6.1992	6.0470	122.3499	127.8695	6.2241	6.0843	112.3626	126.9621
125	6.2059	6.0405	127.6585	132.2434	6.2308	6.0784	117.6351	131.3805
130	6.2129	6.0338	132.9300	136.5940	6.2377	6.0721	122.8735	135.7708
135	6.2201	6.0267	138.1664	140.9241	6.2448	6.0656	128.0795	140.1378
140	6.2275	6.0194	143.3694	145.2365	6.2522	6.0589	133.2546	144.4855
145	6.2351	6.0119	148.5409	149.5331	6.2598	6.0518	138.4005	148.8175

T / K	Form I				Form II			
	Vibrational enthalpy / eV	Vibrational free energy / eV	S / J mol^{-1} K^{-1}	C_v / J mol^{-1} K^{-1}	Vibrational enthalpy / eV	Vibrational free energy / eV	S / J mol^{-1} K^{-1}	C_v / J mol^{-1} K^{-1}
150	6.2430	6.0040	153.6825	153.8159	6.2676	6.0445	143.5184	153.1366
155	6.2510	5.9959	158.7959	158.0865	6.2757	6.0369	148.6100	157.4453
160	6.2593	5.9876	163.8821	162.3465	6.2839	6.0291	153.6766	161.7453
165	6.2679	5.9790	168.9429	166.5973	6.2924	6.0210	158.7195	166.0384
170	6.2766	5.9701	173.9794	170.8396	6.3012	6.0127	163.7400	170.3258
175	6.2856	5.9609	178.9928	175.0749	6.3101	6.0040	168.7390	174.6085
180	6.2948	5.9515	183.9840	179.3035	6.3192	5.9952	173.7179	178.8873
185	6.3042	5.9419	188.9544	183.5263	6.3286	5.9860	178.6775	183.1625
190	6.3138	5.9319	193.9046	187.7436	6.3382	5.9766	183.6189	187.4345
195	6.3236	5.9218	198.8359	191.9558	6.3481	5.9670	188.5428	191.7033
200	6.3337	5.9113	203.7488	196.1629	6.3581	5.9571	193.4501	195.9689
205	6.3439	5.9006	208.6443	200.3650	6.3684	5.9470	198.3415	200.2311
210	6.3544	5.8897	213.5230	204.5618	6.3789	5.9366	203.2176	204.4894
215	6.3652	5.8785	218.3855	208.7531	6.3896	5.9259	208.0793	208.7434
220	6.3761	5.8671	223.2326	212.9386	6.4005	5.9150	212.9269	212.9924
225	6.3872	5.8554	228.0648	217.1178	6.4116	5.9038	217.7609	217.2356
230	6.3986	5.8434	232.8824	221.2899	6.4230	5.8924	222.5819	221.4725
235	6.4102	5.8312	237.6861	225.4543	6.4346	5.8808	227.3903	225.7019
240	6.4219	5.8188	242.4764	229.6101	6.4464	5.8688	232.1864	229.9229
245	6.4340	5.8061	247.2534	233.7568	6.4584	5.8567	236.9705	234.1346
250	6.4462	5.7932	252.0175	237.8930	6.4707	5.8443	241.7430	238.3359
255	6.4586	5.7800	256.7693	242.0181	6.4831	5.8316	246.5040	242.5256
260	6.4713	5.7666	261.5085	246.1311	6.4958	5.8187	251.2539	246.7028
265	6.4841	5.7529	266.2359	250.2308	6.5087	5.8056	255.9926	250.8661
270	6.4972	5.7390	270.9513	254.3161	6.5218	5.7922	260.7205	255.0146
275	6.5105	5.7248	275.6550	258.3861	6.5351	5.7786	265.4376	259.1470
280	6.5240	5.7104	280.3471	262.4398	6.5487	5.7647	270.1441	263.2623
285	6.5377	5.6958	285.0279	266.4758	6.5624	5.7506	274.8399	267.3590
290	6.5516	5.6809	289.6973	270.4933	6.5764	5.7362	279.5251	271.4364
291	6.5544	5.6779	290.6298	271.2944	6.5792	5.7333	280.4609	272.2495

T / K	Form I				Form II			
	Vibrational enthalpy / eV	Vibrational free energy / eV	S / J mol^{-1} K^{-1}	C_v / J mol^{-1} K^{-1}	Vibrational enthalpy / eV	Vibrational free energy / eV	S / J mol^{-1} K^{-1}	C_v / J mol^{-1} K^{-1}
292	6.5572	5.6748	291.5618	272.0948	6.5820	5.7304	281.3963	273.0616
293	6.5600	5.6718	292.4934	272.8944	6.5848	5.7275	282.3311	273.8730
294	6.5629	5.6688	293.4245	273.6931	6.5877	5.7245	283.2656	274.6835
295	6.5657	5.6657	294.3553	274.4910	6.5905	5.7216	284.1998	275.4933
**298**	**6.5743**	**5.6565**	**297.1446**	**276.8799**	**6.5991**	**5.7127**	**286.9995**	**277.9170**
300	6.5800	5.6504	299.0020	278.4681	6.6049	5.7068	288.8639	279.5284
305	6.5946	5.6347	303.6375	282.4236	6.6195	5.6917	293.5174	283.5408
310	6.6093	5.6189	308.2618	286.3563	6.6343	5.6763	298.1603	287.5294
315	6.6242	5.6028	312.8749	290.2654	6.6493	5.6608	302.7925	291.4934
320	6.6394	5.5865	317.4766	294.1498	6.6645	5.6450	307.4140	295.4318
325	6.6547	5.5699	322.0670	298.0088	6.6799	5.6289	312.0248	299.3436
330	6.6703	5.5531	326.6460	301.8414	6.6955	5.6126	316.6245	303.2281
335	6.6860	5.5360	331.2136	305.6470	6.7114	5.5961	321.2134	307.0844
340	6.7020	5.5188	335.7698	309.4246	6.7274	5.5793	325.7913	310.9116
345	6.7181	5.5012	340.3144	313.1736	6.7436	5.5623	330.3579	314.7093
350	6.7344	5.4835	344.8473	316.8934	6.7600	5.5451	334.9131	318.4766
355	6.7509	5.4655	349.3685	320.5833	6.7766	5.5276	339.4571	322.2130
360	6.7676	5.4473	353.8778	324.2426	6.7934	5.5099	343.9895	325.9179
365	6.7845	5.4288	358.3751	327.8710	6.8104	5.4920	348.5104	329.5908
370	6.8016	5.4101	362.8605	331.4679	6.8275	5.4738	353.0194	333.2310
375	6.8189	5.3912	367.3338	335.0329	6.8449	5.4554	357.5165	336.8385
380	6.8363	5.3721	371.7946	338.5655	6.8624	5.4367	362.0018	340.4126
385	6.8540	5.3527	376.2433	342.0654	6.8802	5.4179	366.4748	343.9531
390	6.8718	5.3331	380.6795	345.5324	6.8981	5.3987	370.9355	347.4598
395	6.8898	5.3132	385.1030	348.9661	6.9162	5.3794	375.3839	350.9323
400	6.9080	5.2932	389.5140	352.3664	6.9345	5.3598	379.8199	354.3703
405	6.9263	5.2729	393.9121	355.7329	6.9529	5.3400	384.2431	357.7738
410	6.9448	5.2523	398.2975	359.0658	6.9715	5.3200	388.6538	361.1428
415	6.9635	5.2316	402.6699	362.3646	6.9903	5.2998	393.0515	364.4769
420	6.9824	5.2106	407.0291	365.6295	7.0093	5.2793	397.4363	367.7761

T / K	Form I				Form II			
	Vibrational enthalpy / eV	Vibrational free energy / eV	S / J mol^{-1} K^{-1}	C_v / J mol^{-1} K^{-1}	Vibrational enthalpy / eV	Vibrational free energy / eV	S / J mol^{-1} K^{-1}	C_v / J mol^{-1} K^{-1}
425	7.0014	5.1894	411.3753	368.8604	7.0285	5.2586	401.8080	371.0408
430	7.0206	5.1680	415.7081	372.0573	7.0478	5.2376	406.1666	374.2704
435	7.0400	5.1463	420.0278	375.2201	7.0673	5.2165	410.5120	377.4654
440	7.0595	5.1244	424.3339	378.3490	7.0869	5.1951	414.8440	380.6256
445	7.0792	5.1023	428.6265	381.4441	7.1067	5.1735	419.1625	383.7514
450	7.0990	5.0800	432.9056	384.5054	7.1267	5.1516	423.4675	386.8426
500	7.3059	4.8447	474.9363	413.3005	7.3348	4.9212	465.7586	415.9023
550	7.5269	4.5880	515.5556	438.9748	7.5572	4.6692	506.6363	441.7885
600	7.7604	4.3106	554.7515	461.8578	7.7922	4.3963	546.0846	464.8443

References

[1] Clark, S. J.; Segall, M. D.; Pickard, C. J.; Hasnip, P. J.; Probert, M. I. J.; Refson, K.; Payne, M. C. First Principles Methods Using CASTEP. *Zeitschrift für Krist. - Cryst. Mater.* **2005**, *220* (5–6), 567–570. https://doi.org/10.1524/zkri.220.5.567.65075.

[2] Macrae, C. F.; Sovago, I.; Cottrell, S. J.; Galek, P. T. A.; McCabe, P.; Pidcock, E.; Platings, M.; Shields, G. P.; Stevens, J. S.; Towler, M.; Wood, P. A. Mercury 4.0 : From Visualization to Analysis, Design and Prediction. *J. Appl. Crystallogr.* **2020**, *53* (1), 226–235. https://doi.org/10.1107/S1600576719014092.

[3] Björkman, T. CIF2Cell: Generating Geometries for Electronic Structure Programs. *Comput. Phys. Commun.* **2011**, *182* (5), 1183–1186. https://doi.org/10.1016/j.cpc.2011.01.013.

[4] Perdew, J. P.; Burke, K.; Ernzerhof, M. Generalized Gradient Approximation Made Simple. *Phys. Rev. Lett.* **1996**, *77* (18), 3865–3868. https://doi.org/10.1103/PhysRevLett.77.3865.

[5] Caldeweyher, E.; Bannwarth, C.; Grimme, S. Extension of the D3 Dispersion Coefficient Model. *J. Chem. Phys.* **2017**, *147* (3), 034112. https://doi.org/10.1063/1.4993215.

[6] Monkhorst, H. J.; Pack, J. D. Special Points for Brillouin-Zone Integrations. *Phys. Rev. B* **1976**, *13* (12), 5188–5192. https://doi.org/10.1103/PhysRevB.13.5188.

2.4. TCG-UNITO Crystal phonon calculations

Contributed by Mattia Raimondo, Lorenzo Donà and Bartolomeo Civalleri

2.4.1. Methodology

The calculations were performed using the HFsol-3c composite method[1], which is a slightly modified version of the HF-3c method[2] targeted to solid-state calculations. Recently, it has been demonstrated that HF-based cost-effective composite methods an accuracy similar to DFT results in treating polymorphism of molecular crystals[3].

The total energy provided by the HFsol-3c method can be written as

$$E_{tot}^{HFsol-3c} = E_{tot}^{HF/SOLMINIX} + E_{disp}^{D3} + E_{BSSE}^{gCP} + E^{SRB}$$

where $E_{tot}^{HF/SOLMINIX}$ is the total energy evaluated at the HF level of theory with a minimal basis set that has been partly revised for solids. The total energy is supplemented with an established semiclassical London dispersion correction, D3 model[4], used in the rational (Becke-Johnson) damping variant and includes dipole-dipole and dipole-quadrupole terms. The removal of the BSSE due to the use of minimal basis set with large BSIE is accomplished through a geometrical counterpoise correction (gCP)[5]. Finally, a short-range basis set (SRB) correction that corrects the systematic overestimation of bond lengths involving electronegative elements is included. In addition, HFsol-3c employs an additional scaling of 0.7 to the original s6 scaling factor in the D3 correction as proposed in ref. 6.

2.4.2. Computational Details

All calculations were performed with the CRYSTAL23 code[7,8]. Two types of geometry optimizations were carried out: (i) full relaxation of both atomic coordinates and lattice parameters, and (ii) atom-only relaxation, in which only atomic positions were optimized while keeping the unit cell fixed at the corresponding experimental values for SLFNMA02 and SLFNMA01. All optimized structures are true minima on the potential energy surface, as confirmed by the absence of imaginary frequencies in the vibrational analysis. Default convergence criteria for geometry optimization and frequency calculations were employed. The tolerances for one- and two-electron integral calculation were set to 10^{-8} and 10^{-8} for the Coulomb and to 10^{-8}, 10^{-8}, and 10^{-30} for the exchange series, respectively. The shrinking factors for the diagonalization of the Hamiltonian matrix in the reciprocal space were set to 6 for the Monkhorst–Pack net and to 6 for the Gilat net. Harmonic vibrational frequencies were calculated on top of the two optimized geometries using a supercell approach with a 1x1x2 and a 2x1x1 supercell for phase I and phase II, respectively. Computed vibrational frequencies were then scaled by 0.86 as proposed for HF-3c.[2]

2.4.3. A few comments

Although in our calculations thermal corrections have been computed at the harmonic level, the use of fixed lattice parameters (i.e. experimental values) allows us to mimic the thermal expansion and the effect of the volume on the vibrational frequencies with respect to the fully optimized structures. This is not a quasi-harmonic approximation rather a rough way to include thermal effects.

Overall, HFsol-3c computed heat capacities at different temperature agree with results obtained with other methods. Not unexpectedly, for the fixed unit cell structures the predicted values of Cv increase because of a softening of the low frequencies vibrational modes. In that case, the enthalpy difference for the two phases is predicted to be of about 6.6 kJ/mol at STP with an estimated transition temperature of 171 °C.

References

[1] L. Doná, J. G. Brandenburg and B. Civalleri, “Extending and assessing composite electronic structure methods to the solid state,” J. Chem. Phys. 151, 121101 (2019)

[2] R. Sure and S. Grimme, “Corrected small basis set Hartree-Fock method for large systems,” J. Comput. Chem. 34, 1672–1685 (2013).

[3] J. A. Weatherby, A. F. Rumson, A. J. A. Price A. O. d. l. Roza and E. R. Johnson, “A density-functional benchmark of vibrational free-energy corrections for molecular crystal polymorphism,” *J. Chem. Phys.* 156, 114108 (2022).

[4] S. Grimme, J. Antony, S. Ehrlich, and H. Krieg, “A consistent and accurate ab initio parametrization of density functional dispersion correction (DFT-D) for the 94 elements H-Pu,” J. Chem. Phys. 132, 154104 (2010).

[5] H. Kruse and S. Grimme, “A geometrical correction for the inter- and intra-molecular basis set superposition error in Hartree-Fock and density functional theory calculations for large systems,” *J. Chem. Phys.* 136, 154101 (2012).

[6] M. Cutini, B. Civalleri, M. Corno, R. Orlando, J.G. Brandenburg, L. Maschio, P. Ugliengo “Assessment of different quantum mechanical methods for the prediction of structure and cohesive energies of molecular crystals” J. Chem. Theory Comput. 12, 3340-3352 (2016).

[7] A. Erba, J.K. Desmarais, S. Casassa, B. Civalleri, L. Donà, I. J. Bush, B. Searle, L. Maschio, L.-E. Daga, A. Cossard, C. Ribaldone, E. Ascrizzi, N. L. Marana, J.-P. Flament, B. Kirtman. CRYSTAL23: A Program for Computational Solid State Physics and Chemistry. J. Chem. Theory Comput. 19, 6891−6932 (2023).

[8] R. Dovesi, V.R. Saunders, C. Roetti, R. Orlando, C. M. Zicovich-Wilson, F. Pascale, B. Civalleri, K. Doll, N.M. Harrison, I.J. Bush, Ph. D’Arco, M. Llunell, M. Causà, Y. Noel, L.

Maschio, A. Erba, M. Rerat, S. Casassa, B.G. Searle, J.K. Desmarais. CRYSTAL23 User's Manual (University of Torino, Torino, 2023).

2.5. Boese group quasi-harmonic multimer imbedding

Contributed by University of Graz: Natalia Goncharova, Alexander List, Johannes Hoja, A.Daniel Boese.

The received sulfamerazine crystal structures were optimized with PBE+MBD [1-3] (light species default settings, 2020 version) using FHI-aims (ver. 231212) [4] together with ASE [5]. As SCF convergence criteria we have utilized throughout 10^{-6} eV, 10^{-3} eV, 10^{-5} electrons/Å^3, and 10^{-4} eV/Å for the total energy, sum of eigenvalues, charge density, and forces, respectively. Optimizations were carried out until all forces were less than 0.005 eV/Å and the k-grid was determined in such a way that for each direction $k \cdot x > 20$ Å for DFT and $k \cdot x > 30$ Å for MBD, where k is the number of k-points and x is the cell length in that direction (Method 1).

Next, structures were optimized with ME3(PBE0+MBD:PBE+MBD), which is a multimer embedding method utilizing up to trimer corrections, and light settings. This multimer embedding method is implemented in the MEmbed code [6] and is abbreviated from now on as ME3 (Method 2). A cutoff radius of 4 Å was employed for all MEmbed calculations. After this step, internal minimizations (fixed cell vectors) at the ME3/tight level were performed (Method 3).

Table 2.5.1. Energy differences between sulfamerazine polymorphs (SLFNMA02-SLFNMA01) in kJ/mol for a single molecule within their respective unit cells. ME3 stands for ME3(PBE0+MBD:PBE+MBD). Temperature T is given in K, pressure p in atm and entropy S in J/(mol · K).

Method	Energy	Phonons	ΔE	T	p	ΔG	ΔH	ΔS
1	PBE+MBD/light	n/a	6.8	n/a	n/a	n/a	n/a	n/a
2	ME3/light	ME3/light	6.9	300	n/a	1.2[a]	n/a	n/a
3	ME3/tight	n/a	7.9	n/a	n/a	n/a	n/a	n/a
4	ME3/light	ME3/light	6.8[b]	0	0	4.6	4.6	0
4	ME3/light	ME3/light	6.3[b]	298.15	1	1.6	5.1	11.9
4	ME3/light	ME3/light	n/a	420	1	0.2	4.2	9.6
4	ME3/light	ME3/light	n/a	445	1	0.0	3.9	8.9
5	ME3/tight	ME3/light	7.6[c]	0	0	5.7	5.7	0
5	ME3/tight	ME3/light	6.9[c]	298.15	1	2.9	6.0	10.1
5	ME3/tight	ME3/light	n/a	420	1	1.9	4.7	6.9
5	ME3/tight	ME3/light	n/a	500	1	1.4	3.3	3.8

[a] Helmholtz free energy
[b] Constraint volume optimization with the QHA result
[c] Constraint volume optimization with the QHA result (ME3/light), followed by a single-point calculation (ME3/tight)

Quasiharmonic approximation (QHA) of Sulfamerazine: After optimization with ME3/light different scaled structures were created with unit cell volumes ranging between 0.9 and 1.15 times the optimized volumes. These structures were optimized with constraint volume and the harmonic vibrational free energieswere calculated at the ME3/light level. These calculations were performed using the phonopy code [7] with atomic displacements of 0.005 Å and a q-grid satisfying the condition $q \cdot x > 50$ Å. For the finite displacements supercells of at least 12 Å in every direction were used.

Additionally, single points with ME3/tight were calculated on top of ME3/light structures. The resulting energies were fitted to the Murnaghan equation of sate (EOS), allowing the determination of equilibrium unit cell volumes at finite temperatures (see Figure 2.5.1). The energy difference between two polymorphs are represented in Figure 2.5.2.

Two sets of minimal unit cell volumes were estimated with QHA (one set obtained with light settings, the second one with tight lattice energies and structures and phonons calculated with light settings).

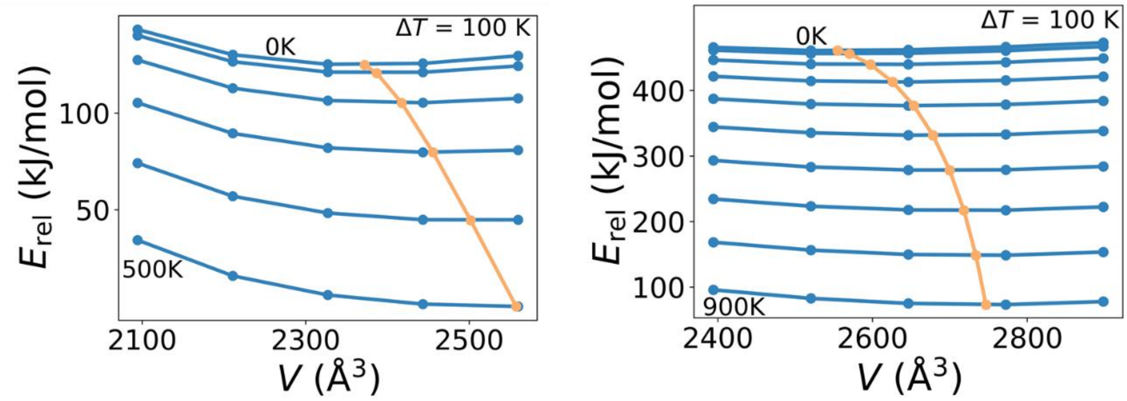

Figure 2.5.1. The EOS fits for the (a) II (SLFNMA01) and (b) I (SLFNMA02) sulfamerazine polymorphs at 101.325 kPa. The lattice energies were calculated with tight settings. The blue lines trace the EOS fit at a specific temperature, while the orange lines highlight the respective equilibrium unit cell volumes and their corresponding energies.

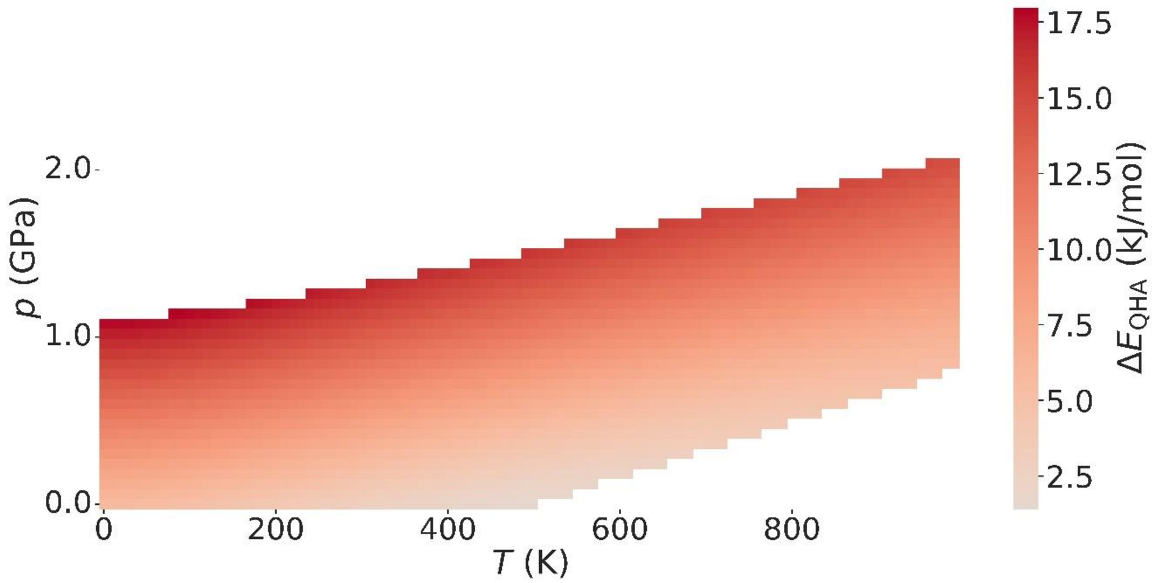

Figure 2.5.2. Energy difference between polymorphs I and II at various conditions in kJ/mol, where the red area indicates favorable conditions for the form II (SLFNMA01).

Table 2.5.2. Heat capacities in J/(mol·K) at constant pressure of 1 atm calculated at ME3(PBE0+MBD:PBE+MBD)/light (ME3/light) and ME3(PBE0+MBD:PBE+MBD)/tight (ME3/tight) level for different temperatures (in K).

	SLFNMA01		SLFNMA02	
T	ME3/light	ME3/tight	ME3/light	ME3/tight
240	236.3	237.8	235.8	235.9
250	245.0	246.6	244.1	244.1
260	253.7	255.4	252.3	252.3
270	262.4	264.1	260.5	260.5
280	271.0	272.8	268.7	268.6
290	279.6	281.4	276.7	276.6
300	288.1	290.0	284.7	284.5
310	296.6	298.5	292.7	292.4
320	305.0	307.0	300.5	300.2
330	313.3	315.3	308.2	307.8
340	321.6	323.6	315.9	315.4
350	329.7	331.8	323.4	322.9

Structures directly corresponding to 0 K, 0 Pa (including zero-point effects) and 298.15 K, 101.325 kPa were created and optimized at constant volume with ME3/light (Method 4). For the second set single point calculations with tight species default settings were performed on these structures (Method 5).

2.5.1. Results

The resulting energies and heat capacities are represented in Table 2.5.1 and Table 2.5.2. Using the ME3/light method, the transition temperature is estimated to be 445 K, while the ME3/tight method suggests it is above 500 K and cannot be determined.

References

[1] J. P. Perdew, K. Burke, M. Ernzerhof, Generalized gradient approximation made simple, *Phys. Rev. Lett.*, **1996**, *77*, 3865.

[2] A. Tkatchenko, R. A. DiStasio, R. Car, M. Scheffler, Accurate and Efficient Method for Many-Body van der Waals Interactions, *Phys. Rev. Lett.* **2012**, *108*, 236402.

[3] A. Ambrosetti, A. M. Reilly, R. A. DiStasio, A. Tkatchenko, Long-range correlation energy calculated from coupled atomic response functions, *J. Chem. Phys.* **2014**, *140*, 18A508.

[4] V. Blum, R. Gehrke, F. Hanke, P. Havu, V. Havu, X. Ren, K. Reuter, M. Scheffler, Ab initio molecular simulations with numeric atom-centered orbitals, *Comput. Phys. Commun.* **2009**, *180*, 2175–2196.

[5] A. H. Larsen, J. J. Mortensen, J. Blomqvist, I. E Castelli, R. Christensen, M. Du-lak, J. Friis,

[6] M. N Groves, B. Hammer, C. Hargus, The atomic simulation environment–a Python library for working with atoms, **2017**.

[7] J. Hoja, A. List, A. D. Boese, Multimer Embedding Approach for Molecular Crystals up to Har- monic Vibrational Properties, *J. Chem. Theory Comput.* **2024**, *20*, 357-367.

A. Togo, I. Tanaka, First principles phonon calculations in materials science. *Scr. Mater.* **2015**, *108*, 1-5.

2.6. Cervinka group quasi- harmonic combined method periodic calculations.

Contributed by UCTPrague: Ctirad Červinka and Reynaldo Geronia II

We used the quasi-harmonic approximation (QHA)[1] to model structural and thermodynamic properties of selected sulfamerazine polymorphs. Within QHA, unit-cell geometries are repeatedly optimized at various constrained volumes, resulting in sampling the dependence of the static electronic energy of the crystal on its volume. Around the minimum of this energy – volume dependence, harmonic phonon calculations are performed with the finite-displacement method for eight crystal volumes, sampling the anharmonic dependence of phonon frequencies on crystal volume. Phonon calculations were performed for supercells spanning at least 10 Å in each direction, allowing for a reasonable treatment of the impact of phonon dispersion on thermodynamic properties of the crystal.[1] To save computational resources, the composite formulation of QHA,[2] combining full (volume-dependent) QHA at a low QM level of theory

with a single-volume harmonic calculation with a more sophisticated method to correct the output of the cheaper method, was followed. Semi-empirical third-order density-functional tight binding DFTB3 theory[3] within periodic boundary conditions was used along the D4 dispersion correction[4] and the 3ob parametrization,[5] implemented in the DFTB+ code, version 24.1,[6] as the low-level QM method with the composite QHA. The high-level of theory within our composite QHA was selected from the density-functional theory framework. Periodic PBE-D4 model[4,7] was used along with the PAW formalism[8] (hard PAW potentials and 1000 eV plane-wave kinetic-energy cut-off), implemented in VASP, version 6.4.2.[9-11] See our previous publication for more details on the composite QHA computational setup.[2] All underlying electronic-structure calculations were performed using a 3×2×1 *k*-point mesh to sample the respective *k*-space for unit-cell optimizations, whereas only the electronic Γ-point was sampled for the supercells upon phonon modeling.

We focused only on sulfamerazine forms I and II. Complete QHA results for form I are presented and discussed in this work. Comparing the calculated isobaric heat capacities for the form I with reference experimental values reveals a very good agreement of our composite QHA model, yielding slightly underestimated results, but in general differing by only 2–3% over a broad temperature interval. That can be considered as a very good computational accuracy considering the typical heat capacity outcomes of the QHA model relying on PBE-D theories.[1,2,12,13] Interestingly, the composite QHA model predicts a relatively low (1% on the relative scale) difference between the quasi-harmonic isobaric and harmonic isochoric heat capacity of the form I, which can be understood as an imprint of a lesser thermal expansion of the crystal at near-ambient conditions. Within the harmonic model only, both DFTB and PBE models agree closely on the C_V of the form I at ambient conditions. However, the quasi-harmonic model relying solely on the DFTB theory overestimates C_p (by 2–5%), and this computational artifact is even more amplified at elevated temperatures. Performing the composite QHA, correcting the DFTB outputs to match the PBE outcome at a reference volume then improves the overall C_p appreciably. All sets of calculated heat capacities for the SMZ form I are listed in Table 2.6.1.

Table 2.6.1 Form I heat capacities (in J/mol/K) as calculated by the Cervinka group

T [K] form I	Expt. C_p	DFTB HA only C_V	DFTB QHA C_p	PBE HA only C_V	Composite QHA PBE:DFTB C_p	Percentage error of the PBE:DFTB data set
240	245.8	239.9	257.0	238.7	239.9	−2.4%
245	250.1	244.0	261.4	242.9	244.1	−2.4%
250	254.4	248.1	265.8	247.0	248.4	−2.4%
255	258.6	252.1	270.2	251.2	252.6	−2.3%
260	262.6	256.1	274.6	255.3	256.8	−2.2%
265	267.4	260.1	278.9	259.4	261.0	−2.4%
270	271.5	264.1	283.2	263.5	265.3	−2.3%
275	275.7	268.1	287.5	267.5	269.4	−2.3%
280	280.0	272.0	291.7	271.6	273.6	−2.3%
285	284.3	275.9	296.0	275.6	277.8	−2.3%
290	288.5	279.8	300.2	279.7	281.9	−2.3%
295	292.8	283.7	304.4	283.7	286.1	−2.3%
300	297.1	287.5	308.5	287.6	290.2	−2.3%
305	301.3	291.3	312.7	291.6	294.3	−2.3%
310	305.6	295.1	316.9	295.5	298.3	−2.4%
315	309.9	298.9	321.1	299.4	302.4	−2.4%
320	314.2	302.6	325.3	303.3	306.4	−2.5%
325	318.5	306.4	329.6	307.2	310.4	−2.5%
330	322.7	310.1	333.8	311.0	314.3	−2.6%
335	326.9	313.7	338.2	314.8	318.2	−2.7%
340	331.1	317.3	342.5	318.6	322.0	−2.7%
345	335.4	321.0	347.0	322.4	325.9	−2.8%
350	339.6	324.5	351.4	326.1	329.6	−2.9%

Current composite QHA model underestimates the equilibrium unit-cell volume of SMZ form I at finite temperatures by 5.6% which is not particularly good result when compared to a typical performance of DFT-D powered QHA models of molecular crystals.[1,2,12,13] However, the current model consistently maintains that percentage volume error over a broad temperature interval, spanning from 30 K to 200 K as listed in Table 2.6.2. That indicates that the thermal expansion of this crystal structure is captured very well. SMZ form I expands its volume by 2.2% upon heating from 30 K to 200 K as observed experimentally, whereas our model states this value at 2.3%. Due to the orientation of the hydrogen bonding (N–H…N and N–H…O bonds) predominantly in direction of the lattice vector *a*, impeding thus the expansion of the material in this direction, most of this thermal expansion manifests in directions of the lattice vectors *b* and *c*, where weaker dispersion interaction govern the crystal cohesion. On the other hand, calculated equilibrium lengths of the *b* and *c* lattice vectors are the dominant error sources

for the overall overestimation of the bulk crystal density at finite temperatures. Calculated linear expansion in the directions of the *a*, *b* and *c* lattice vectors reach 0.0%, 1.3%, and 1.0%, respectively. Both theory and experiment agree on this behavior closely, confirming the previously observed capability of our QHA models to capture the anisotropy of thermal expansion due to the directionality of site-specific cohesive interactions in the crystal structure.[14] Such results justify the functionality of the composite QHA model also for the treatment of anisotropic thermal expansion.

Table 2.6.2. Thermal expansion of SMZ form I documented with values of the unit-cell volume (*V*, in Å^3) its percentage error δ_V and lattice parameters (*a*, *b*, *c*, all in Å) as calculated by the Cervinka group, compared with values interpolated from raw experimental data assembled in this work.

	Experiment				Composite QHA PBE:DFTB				
T	*a*	*B*	*c*	*V*	*a*	*b*	*c*	*V*	δ_V
30	14.535	8.226	22.042	2635.2	14.658	7.952	21.347	2488.2	−5.6%
40	14.539	8.228	22.057	2638.3	14.658	7.955	21.354	2490.0	−5.6%
60	14.540	8.229	22.087	2642.9	14.657	7.964	21.372	2494.7	−5.6%
80	14.542	8.232	22.120	2648.3	14.656	7.975	21.395	2500.7	−5.6%
100	14.549	8.237	22.158	2655.5	14.654	7.987	21.421	2507.1	−5.6%
120	14.559	8.243	22.199	2663.9	14.653	8.000	21.448	2514.2	−5.6%
140	14.569	8.247	22.239	2672.2	14.651	8.014	21.476	2521.6	−5.6%
160	14.576	8.251	22.276	2679.3	14.652	8.027	21.505	2529.2	−5.6%
180	14.582	8.256	22.311	2685.7	14.650	8.041	21.535	2536.8	−5.5%
200	14.592	8.261	22.352	2694.3	14.652	8.054	21.565	2544.8	−5.5%
220	n/a	n/a	n/a	n/a	14.652	8.068	21.595	2552.8	n/a
240	n/a	n/a	n/a	n/a	14.653	8.081	21.626	2560.8	n/a
260	n/a	n/a	n/a	n/a	14.655	8.094	21.657	2568.9	n/a
280	n/a	n/a	n/a	n/a	14.658	8.107	21.687	2577.1	n/a
300	n/a	n/a	n/a	n/a	14.660	8.120	21.718	2585.3	n/a
320	n/a	n/a	n/a	n/a	14.664	8.132	21.749	2593.5	n/a
340	n/a	n/a	n/a	n/a	14.668	8.144	21.780	2601.8	n/a
Δ^a	0.057	0.035	0.310	59.1	−0.006	0.102	0.218	56.6	−4.2%

[a] Quantity Δ stands for the absolute differences between the form I structures at 200 K and 30 K.

Despite our efforts to optimize the unit-cell geometry of the form II to relatively tight convergence criteria with both DFTB3-D4/3ob and PBE-D4/PAW models, subsequent phonon calculations always yielded multiple imaginary phonon modes which restrained us from calculations of thermodynamic properties for that polymorph.

References

[1] C. Červinka, M. Fulem, R. P. Stoffel and R. Dronskowski, *J. Phys. Chem. A*, 2016, **120**, 2022-2034.

[2] J. Ludík, V. Kostková, Š. Kocian, P. Touš, V. Štejfa and C. Červinka, *J. Chem. Theory Comput.*, 2024, **20**, 2858-2870.
[3] M. Gaus, Q. Cui and M. Elstner, *J. Chem. Theory Comput.*, 2011, **7**, 931-948.
[4] E. Caldeweyher, S. Ehlert, A. Hansen, H. Neugebauer, S. Spicher, C. Bannwarth and S. Grimme, *J. Chem. Phys.*, 2019, **150**, 154122.
[5] M. Gaus, A. Goez and M. Elstner, *J. Chem. Theory Comput.*, 2013, **9**, 338-354.
[6] B. Hourahine, B. Aradi, V. Blum, F. Bonafé, A. Buccheri, C. Camacho, C. Cevallos, M. Y. Deshaye, T. Dumitrică, A. Dominguez, S. Ehlert, M. Elstner, T. van der Heide, J. Hermann, S. Irle, J. J. Kranz, C. Köhler, T. Kowalczyk, T. Kubař, I. S. Lee, V. Lutsker, R. J. Maurer, S. K. Min, I. Mitchell, C. Negre, T. A. Niehaus, A. M. N. Niklasson, A. J. Page, A. Pecchia, G. Penazzi, M. P. Persson, J. Řezáč, C. G. Sánchez, M. Sternberg, M. Stöhr, F. Stuckenberg, A. Tkatchenko, V. W.-z. Yu and T. Frauenheim, *J. Chem. Phys.*, 2020, **152**, 124101.
[7] J. P. Perdew, K. Burke and M. Ernzerhof, *Phys. Rev. Lett.*, 1996, **77**, 3865-3868.
[8] P. E. Blöchl, *Phys. Rev. B*, 1994, **50**, 17953-17979.
[9] G. Kresse and J. Furthmüller, *Phys. Rev. B*, 1996, **54**, 11169-11186.
[10] G. Kresse and J. Furthmüller, *Comput. Mater. Sci.*, 1996, **6**, 15-50.
[11] G. Kresse and D. Joubert, *Phys. Rev. B*, 1999, **59**, 1758-1775.
[12] C. Červinka, M. Klajmon, and V. Štejfa, *J. Chem. Theory Comput.*, 2019, **15**, 5563-5578.
[13] C. Červinka, and V. Štejfa, *ChemPhysChem*, 2020, **21**, 1184-1194.
[14] V. Pokorný, P. Touš, V. Štejfa, K. Růžička, J. Rohlíček, J. Czernek, J. Brus, and C. Červinka, *Phys. Chem. Chem. Phys.*, **2022**, 24, 25904-25917.

2.7. Loncaric group quasi-harmonic calculations with universal machine learning potentials

Contributed by Ivor Lončarić and Bruno Mladineo

Machine learning interatomic potentials (MLIPs) are revolutionising atomistic modelling of materials. Although their application in modelling molecular crystals has been relatively less exploited, MLIPs have been successfully used to model free energies and predict relative stabilities of polymorphs as a function of temperature. [1,2,3] However, training MLIP for a given material requires building the database. On the other hand, recently, universal MLIPs (UMLIPs) that are pretrained and ready to use are becoming increasingly accurate. UMLIPs are also proving useful for modelling molecular crystals [4,5,6]. Therefore, in this contribution, we use two UMLIPs based on the MACE architecture [7,8] and the SPICE dataset [9]. The first one is MACE-OFF (medium) [10], trained on the SPICE1 dataset, and the second one is

MACE@SPICE2 [6], which is trained on the improved version of the database SPICE2.

All free energy calculations are performed using Phonopy [11] within the quasi-harmonic approximation (QHA). We used supercells of at least 12 Å in any direction and 18 volume points. Once the force constants are obtained, we use 17×17×17 Monkhorst-Pack q-point sampling to derive all phonon-related properties. MACE-OFF predicts non-monotonic differences in calculated heat capacities (Fig. 15) that are due to the numerical differentiation of the interpolated QHA curves on a rather dense volume grid (Figure 2.7.1).

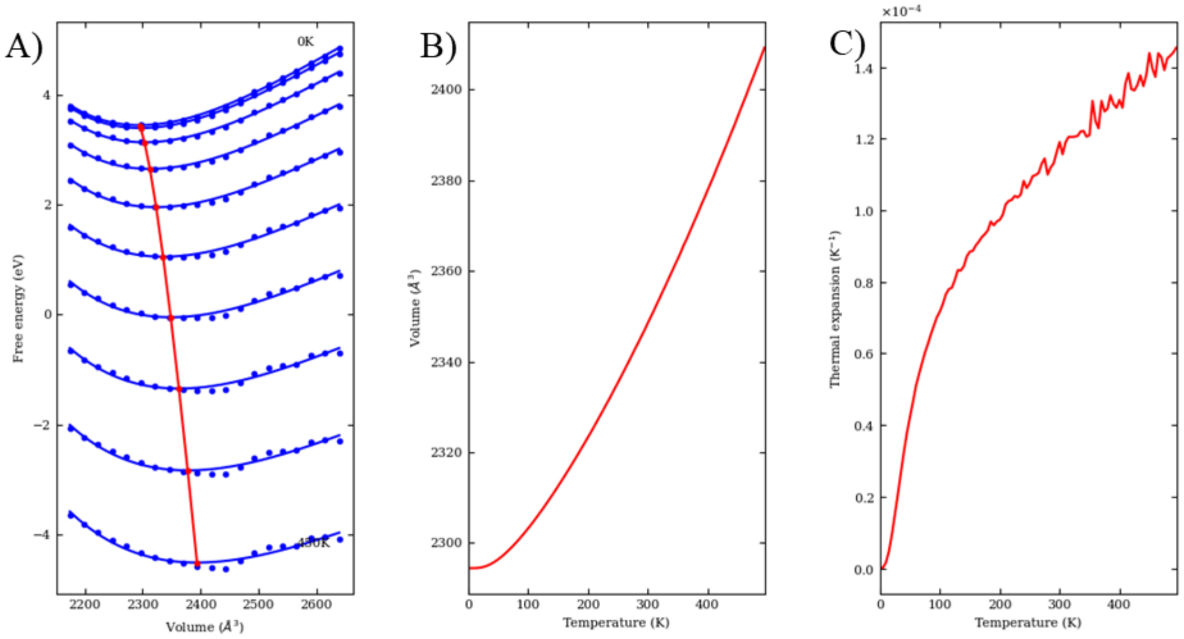

Figure 2.7.1. Quasi-harmonic approximation results showing (A) free energy as a function of volume at various temperatures between 0 and 450 K, (B) volume vs temperature, and (C) thermal expansion coefficient vs temperature.

References

[1] Kapil, Venkat, and Edgar A. Engel. "A complete description of thermodynamic stabilities of molecular crystals." Proceedings of the National Academy of Sciences 119, no. 6, e2111769119 (2022)

[2] Mladineo, Bruno, and Ivor Lončarić. "Thermosalient phase transitions from machine learning interatomic potential." Crystal growth & design 24, no. 20 , 8167-8173 (2024)

[3] Hunnisett, Lily M., Nicholas Francia, Jonas Nyman, Nathan S. Abraham, Srinivasulu Aitipamula, Tamador Alkhidir, Mubarak Almehairbi et al. "The seventh blind test of crystal structure prediction: structure ranking methods." Acta Crystallographica Section B Structural Science, Crystal Engineering and Materials 80, no. 6 (2024).

[4] Žugec, Ivan, R. Matthias Geilhufe, and Ivor Lončarić. "Global machine learning potentials

for molecular crystals." The Journal of chemical physics 160, no. 15 , 154106 (2024)
[5] Nickerson, Cameron J., and Erin R. Johnson. "Assessment of a foundational machine-learned potential for energy ranking of molecular crystal polymorphs." Physical Chemistry Chemical Physics 27, 11930-11940 (2025).
[6] Kholtobina, Anastasiia, and Ivor Lončarić. "Exploring elastic properties of molecular crystals with universal machine learning interatomic potentials." Materials & Design 254, 114047 (2025)
[7] Batatia, Ilyes, Simon Batzner, Dávid Péter Kovács, Albert Musaelian, Gregor NC Simm, Ralf Drautz, Christoph Ortner, Boris Kozinsky, and Gábor Csányi. "The design space of E (3)-equivariant atom-centred interatomic potentials." Nature Machine Intelligence 7, no. 1, 56-67 (2025)
[8] Batatia, Ilyes, David P. Kovacs, Gregor Simm, Christoph Ortner, and Gábor Csányi. "MACE: Higher order equivariant message passing neural networks for fast and accurate force fields." Advances in neural information processing systems 35 , 11423-11436 (2022)
[9] Eastman, Peter, Pavan Kumar Behara, David L. Dotson, Raimondas Galvelis, John E. Herr, Josh T. Horton, Yuezhi Mao et al. "Spice, a dataset of drug-like molecules and peptides for training machine learning potentials." Scientific Data 10, no. 1, 11 (2023)
[10] Kovács, Dávid Péter, J. Harry Moore, Nicholas J. Browning, Ilyes Batatia, Joshua T. Horton, Yixuan Pu, Venkat Kapil et al. "Mace-off: Short-range transferable machine learning force fields for organic molecules." Journal of the American Chemical Society 147, no. 21, 17598-17611 (2025)
[11] Togo, Atsushi, Laurent Chaput, Terumasa Tadano, and Isao Tanaka. "Implementation strategies in phonopy and phono3py." Journal of Physics: Condensed Matter 35, no. 35, 353001 (2023)

2.8. Hoser group: Normal Mode Refinement of frequencies

Contributed by Anna Hoser, Helena Butkiewicz and Joanna Krzeszczakowska

2.8.1. NoMoRe

Normal mode refinement method[1,2], that enables to refine the frequencies from DFT against single-crystal X-ray data. Instead of refining anisotropic displacement parameters (ADPs), we refine selected low-frequency vibrational modes. Next, all frequencies are used for estimation of thermodynamic properties such as vibrational contributions to free energy or heat capacities. It can be accomplished via the http://nomore.chem.uw.edu.pl/ free of charge web server.

2.8.2. Computational details

CRYSTAL23

Periodic DFT calculations were carried out in *CRYSTAL23*[3,4] for two forms: SMZ_I and SMZ_II. In the calculations only atomic positions were optimized, starting from experimental geometries with unit cell parameters for 150 K structures of SMZ_I and SMZ_II provided by Dejan-Krešimir Bučar.

The calculations were performed with the B3LYP[5,6] functional with D3 dispersion correction[7,8], the 6-31G(d,p) basis set, and standard TOLINTEG (7 7 7 7 25) and SHRINK (8 8) settings. The resulting electronic energies were used to estimate free energies. Vibrational frequencies and normal modes were then computed at the Γ point using the finite displacement method, with geometry optimization controlled by the PREOPTGEOM keyword. Input files were prepared with *cif2crystal*[9].

Single-point energies were calculated with CRYSTAL23, with B3LYP-D3 functional and the Ahlrichs-VTZP basis set.

CASTEP

Periodic DFT calculations were carried out in *CASTEP*[10] for two forms: SMZ_I and SMZ_II. A relaxation of atomic coordinates only, with unit cell parameters kept unrefined (experimental unit cell parameters from 150 K X-ray measurements provided by Dejan-Krešimir Bučar), were performed in the Broyden-Fletcher-Goldfarb-Shanno (BFGS) minimization scheme. Geometry optimization and vibrational frequencies calculations were performed using PBE functional, augmented with an empirical D2 Grimme dispersion correction. Norm-conserving pseudopotentials(NCP) and cut off energy of 1500 eV for plain waive basis sets were employed in the calculations. The k-point sampling of the Brillouin zone was constructed using Monkhorst-Pack scheme and 2 4 2 or 4 4 2 grid. Symmetry of each crystal was maintained during the optimization process. In the geometry optimization, carefully tested and leading to a well converged total energy, criteria of convergence were set to 1.0×10 −8 [eV] for energy,5.0×10 −4 [eV/Å] for force, and 1.0x10 −5 for displacements of atomic positions.

Vibrational frequencies and normal modes were then computed at the Γ point using the DFPT method[11].

For Energy calculations ultrasoft pseudopotentials (OTF) from a built-in library were used and D3 empirical Grimme dispersion correction[7,8] with Becke-Johnson damping were used.

2.8.3. Lattice energy

Table 2.8.1. Details of theoretical intermolecular lattice energy calculations. All energies are in kJ/mol.

		**smz_I**	**smz_II**
**bulk**	**Ebulk +D3**	-25071132.663	-25071240.175
**Molecule 1**	**Emol1 nod+D3**	-3133637.789	-3133631.515
	**Emol1 nod**	-3133457.006	-3133455.729
	**Emol1 cpc**	-3133508.533	-3133514.289
	**BSSE1**	51.527	58.560
**Molecule 2**	**Emol2 nod +D3**	-3133638.700	
	**Emol2 nod**	-3133457.963	
	**Emol2 cpc**	-3133510.341	
	**BSSE2**	52.377	
**Lattice Energy**	**E coh**	-253.338	-273.507
	**E coh +BSSE**	**-201.386**	**-214.948**

2.8.4. Heat Capacity Estimation

Heat capacity was determined using the approach proposed by Aree and Bürgi[12], which has been successfully applied in previous NoMoRe studies[2,13-15]. In this method, acoustic and optical modes are treated using Debye and Einstein approximations, respectively. The difference between heat capacities at constant pressure (Cp) and constant volume (Cv) was estimated using the Nernst–Lindemann relation.

Heat capacity values (Cp and Cv) calculated for smz_I and smz_II are summarized in the table. For smz_I, experimental Cp values were compared with theoretical predictions obtained using two sets of vibrational frequencies: those directly taken from periodic DFT calculations (CRYSTAL23), and those with eight lowest-frequency modes refined using NoMoRe (without a clear improvement in accuracy). Both models yield Cp results comparable to the experimental data. For smz_II, only Cv values were calculated, as the melting point of this form could not be determined — hence, Cp could not be estimated using the Nernst-Lindeman approximation.

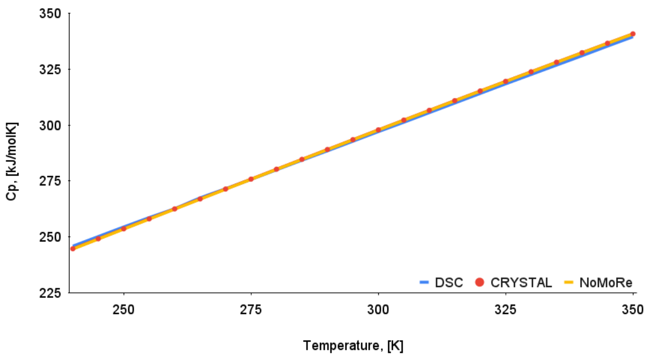

Figure 2.8.1. The heat capacity (Cp) for smz_I obtained from DSC experiment (green solid), DFT Γ-point calculations with acoustic mode frequencies of 50 cm^{-1} (red dots) and NoMoRe (yellow solid). The heat capacity was computed only for temperatures for which the calorimetric data were available.

Table 2.8.2. Heat capacities for smz_I (both, constant pressure and constant volume) and for smz_II (only constant volume) in the 240 – 350 K temperature range. The heat capacities were calculated using three different sets of vibrational frequencies: (1) frequencies obtained from periodic DFT calculations in CRYSTAL, excluding the negative (acoustic) modes; (2) the same CRYSTAL-derived frequencies, but with the acoustic modes replaced by a fixed value of 50 cm^{-1}, following the approach used in NoMoRe; and (3) frequencies refined using the NoMoRe method.

	smz_I						smz_II		
T, [K]	Cp, [J/mol·K]			Cv, [J/mol·K]			Cv, [J/mol·K]		
	CRYSTAL[1]	CRYSTAL[2]	NoMoRe	CRYSTAL[1]	CRYSTAL[2]	NoMoRe	CRYSTAL[1]	CRYSTAL[2]	NoMoRe
240	241,4	244,6	244,5	230,8	233,9	233,8	227,3	230,4	230,3
245	245,9	249,1	249,0	234,9	238,0	237,9	231,4	234,5	234,4
250	250,3	253,6	253,4	238,9	242,0	241,9	235,6	238,6	238,5
255	254,8	258,0	257,9	243,0	246,1	246,0	239,7	242,7	242,6
260	259,3	262,5	262,4	247,0	250,1	250,0	243,7	246,8	246,7
265	263,7	266,9	266,8	251,0	254,1	254,0	247,8	250,9	250,8
270	268,1	271,4	271,3	255,0	258,1	258,0	251,9	255,0	254,9
275	272,6	275,8	275,7	259,0	262,1	262,0	255,9	259,0	258,9
280	277,0	280,3	280,2	263,0	266,1	266,0	259,9	263,0	263,0
285	281,4	284,7	284,6	266,9	270,0	269,9	264,0	267,0	267,0
290	285,8	289,1	289,0	270,9	273,9	273,9	267,9	271,0	271,0
295	290,2	293,5	293,4	274,8	277,9	277,8	271,9	275,0	274,9
300	294,6	297,9	297,8	278,7	281,8	281,7	275,9	279,0	278,9
305	299,0	302,3	302,2	282,6	285,7	285,6	279,8	282,9	282,8
310	303,4	306,6	306,6	286,4	289,5	289,4	283,7	286,8	286,8
315	307,7	311,0	310,9	290,3	293,4	293,3	287,6	290,7	290,7
320	312,0	315,3	315,2	294,1	297,2	297,1	291,5	294,6	294,5
325	316,3	319,6	319,5	297,9	301,0	300,9	295,3	298,4	298,4
330	320,6	323,9	323,8	301,6	304,7	304,7	299,2	302,3	302,2
335	324,9	328,2	328,1	305,4	308,5	308,4	303,0	306,1	306,0
340	329,1	332,5	332,4	309,1	312,2	312,1	306,7	309,8	309,8
345	333,4	336,7	336,6	312,8	315,9	315,8	310,5	313,6	313,5
350	337,6	340,9	340,8	316,5	319,6	319,5	314,2	317,3	317,2

Table 2.8.3. Heat capacities for smz_I (both, constant pressure and constant volume) and for smz_II (only constant volume) in the 240 – 350 K temperature range, CASTEP. The heat capacities were calculated using three different sets of vibrational frequencies: (1) frequencies obtained from periodic DFT calculations in CASTEP, excluding the negative (acoustic) modes; (2) the same CASTEP-derived frequencies, but with the acoustic modes replaced by a fixed value of 50 cm⁻¹; and (3) frequencies refined using the NoMoRe method.

	smz_I						smz_II		
T, [K]	Cp, [J/mol·K]			Cv, [J/mol·K]			Cv, [J/mol·K]		
	CASTEP[1]	CASTEP[2]	NoMoRe	CASTEP[1]	CASTEP[2]	NoMoRe	CASTEP[1]	CASTEP[2]	NoMoRe
240	247.5	251.8	251.8	236.7	240.8	240.8	232.5	236.7	236.7
245	252.1	256.4	256.3	240.9	245.0	244.9	236.8	240.9	240.9
250	256.7	261.0	260.9	245.0	249.1	249.1	241.0	245.1	245.2
255	261.3	265.6	265.5	249.2	253.3	253.2	245.2	249.3	249.4
260	265.8	270.2	270.1	253.3	257.4	257.4	249.4	253.5	253.6
265	270.4	274.7	274.7	257.4	261.5	261.5	253.6	257.7	257.8
270	275.0	279.3	279.2	261.5	265.6	265.6	257.8	261.9	261.9
275	279.5	283.9	283.8	265.6	269.7	269.7	261.9	266.1	266.1
280	284.1	288.4	288.3	269.7	273.8	273.8	266.1	270.2	270.2
285	288.6	293.0	292.9	273.7	277.8	277.8	270.2	274.3	274.4
290	293.1	297.5	297.4	277.7	281.9	281.9	274.3	278.4	278.5
295	297.6	302.0	301.9	281.8	285.9	285.9	278.4	282.5	282.5
300	302.1	306.5	306.4	285.8	289.9	289.9	282.4	286.6	286.6
305	306.6	311.0	310.9	289.7	293.9	293.8	286.5	290.6	290.6
310	311.0	315.4	315.3	293.7	297.8	297.8	290.5	294.6	294.6
315	315.5	319.9	319.8	297.6	301.7	301.7	294.5	298.6	298.6
320	319.9	324.3	324.2	301.5	305.6	305.6	298.4	302.6	302.6
325	324.3	328.7	328.6	305.4	309.5	309.5	302.4	306.5	306.5
330	328.7	333.1	333	309.2	313.3	313.3	306.3	310.4	310.4
335	333.0	337.4	337.4	313.0	317.2	317.2	310.2	314.3	314.3
340	337.4	341.8	341.7	316.8	321.0	320.9	314.0	318.1	318.2
345	341.7	346.1	346	320.6	324.7	324.7	317.8	322.0	322.0
350	346.0	350.4	350.3	324.3	328.4	328.4	321.6	325.7	325.8

2.8.5. Thermodynamics from NoMoRe

The electronic energies obtained from the periodic DFT calculations in combination with the vibrational frequencies derived from the normal mode refinements allow us to estimate the vibrational contributions to the free energies of the polymorphs as a function of temperature, as we have performed in previous work[16–18].

CRYSTAL

The transition temperature was estimated using two approaches. First, we used vibrational frequencies from periodic DFT calculations (B3LYP-D3/6-31G(d,p)), Gamma point calculations, substituting 50 cm⁻¹ for the three acoustic modes (it is usually our starting point for NoMoRe), Figure 2.8.2a. Around 0 K, free energy differences reflect enthalpy differences, dominated by electronic energy. The smz_II form is more stable by 13.4 kJ/mol, consistent with its low-temperature stability. As temperature rises, the higher entropy of the II form causes its free energy to decrease more rapidly, with the curves crossing around 560 K. The calculated

transition temperature is about 200 K higher than observed, but this deviation is expected because of the high sensitivity of the method to small entropy differences, as the free energy curves are nearly parallel. Next, we used frequencies refined with the NoMoRe method, where the eight lowest modes were refined (Figure 2.8.2b). This approach should give us estimates of frequencies in different BZ points and estimates of acoustic modes. This resulted in a slightly higher transition temperature of around 690 K.

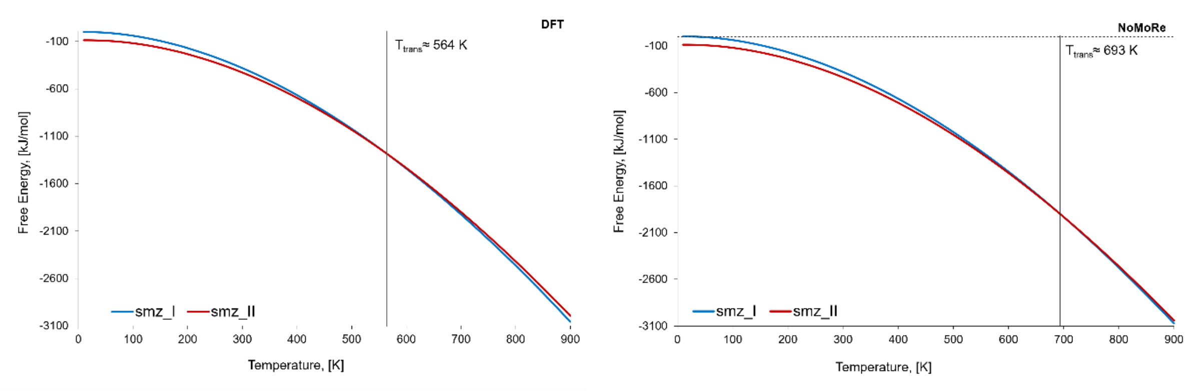

Figure 2.8.2. Free energy of the two polymorphs as a function of temperature, as a) derived from the periodic DFT calculations and b) combined with NoMoRe refinements; B3LYP-D3/6-31G(d,p) level of theory.

In the second case, while vibrational properties were obtained at the B3LYP-D3/6-31G(d,p) level, electronic energies were taken from calculations performed by Bartolomeo Civalleri at the HFsol3c level of theory. It resulted in 390K transition temperature for CRYTSTAL23 frequencies only (Figure 2.8.3a) and 490K when frequencies from NoMoRe were used (Figure 2.8.3b). Third attempt was related with combination of frequencies used in the previous step with electronic energies calculated at B3LYP-D3/Ahlrichs-VTZP level of theory. This merged method resulted in T_{trans} around 510K for CRYSTAL23 (Figure 2.8.4a) and 630K for NoMoRe (Figure 2.8.4b).

Such combined method was used to reduce computational cost while maintaining accuracy. Lower-level frequency calculations capture the important features of molecular vibrations and enable reliable estimation of thermodynamic contributions (enthalpy, entropy, heat capacity), especially when scaled or refined against experimental data. At the same time, computing electronic energies with a larger basis set improves the precision of lattice energy and free energy values. This strategy offers a good balance between accuracy and efficiency and shows consistent results.

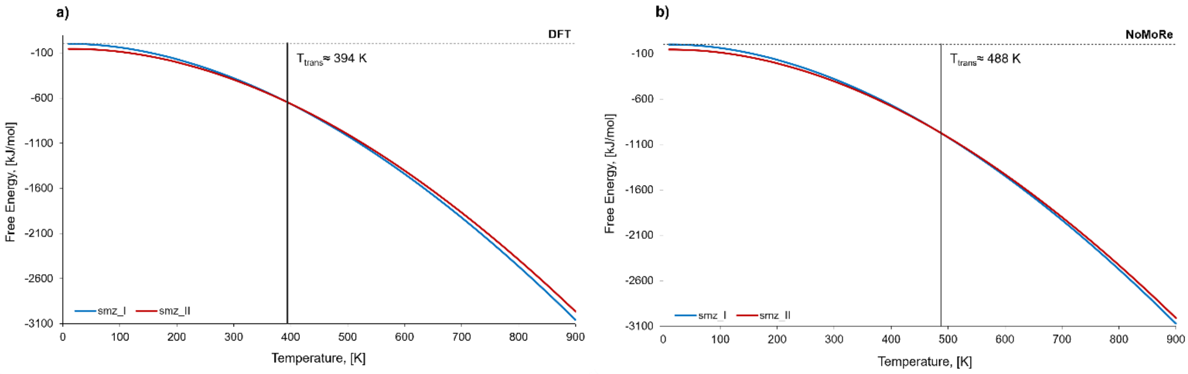

Figure 2.8.3. Free energy of the two polymorphs as a function of temperature, as a) derived from the periodic DFT calculations and b) combined with NoMoRe refinements; combinations of two levels of theory: B3LYP-D3/6-31G(d,p) for frequency calculations and HFsol3c for electronic energy.

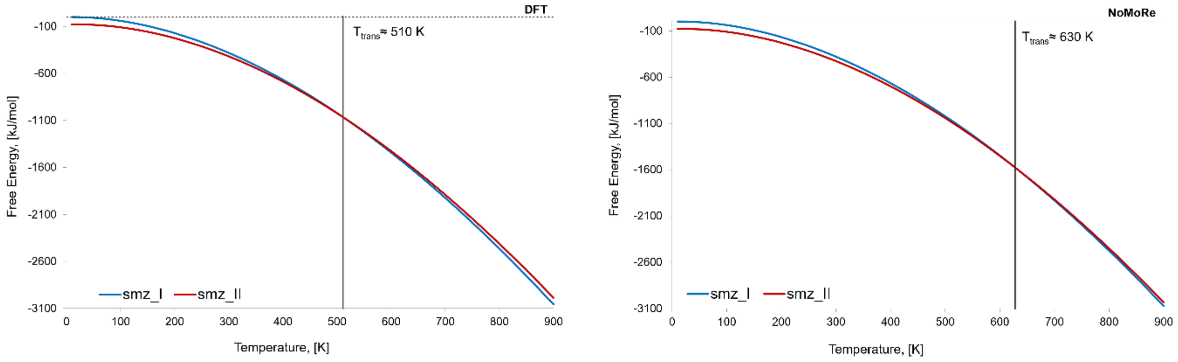

Figure 2.8.4. Free energy of the two polymorphs as a function of temperature, as a) derived from the periodic DFT calculations and b) combined with NoMoRe refinements; combinations of two levels of theory: B3LYP-D3/6-31G(d,p) for frequency calculations and Ahl-VTZP basis set for electronic energy.

CASTEP

The transition temperature was estimated using vibrational frequencies from CASTEP periodic DFT Gamma point calculations. We used frequencies refined with the NoMoRe method, where the six lowest modes were refined (Figure 2.8.5a). This approach should give us estimates of frequencies in different BZ points and estimates of acoustic modes. At 0 K, free energy differences reflect enthalpy differences, dominated by electronic energy. The smz_II form is more stable by 7.9 kJ/mol, consistent with its low-temperature stability. As temperature rises, the higher entropy of the II form causes its free energy to decrease more rapidly, with the curves crossing around 449 K (176 °C).

NoMoRe refinement was compared to simle approach where negative values of acoustic frequencies were substituted by 50 cm^{-1} (Figure 2.8.5b). This approach resulted in lower

transition temperature of 386 K (113 °C). NoMoRe refinement corrected this value.

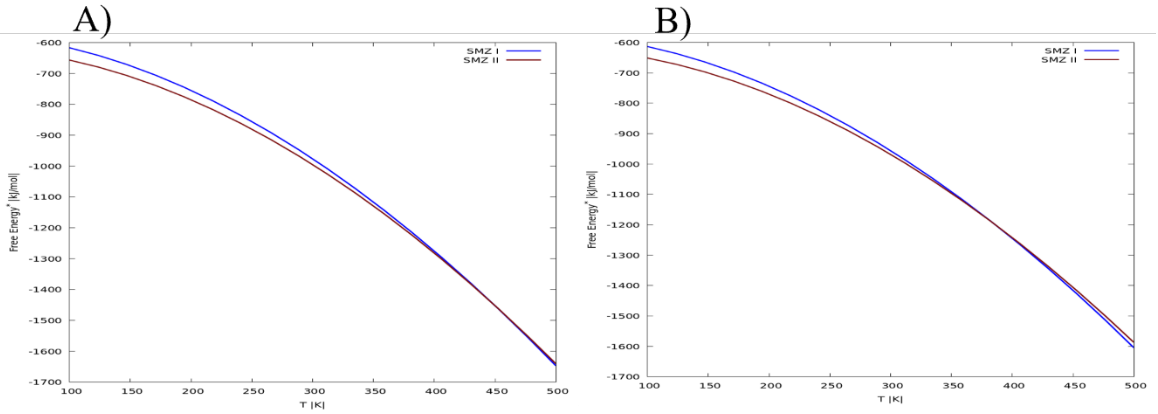

Figure 2.8.5. Free energy of the two polymorphs as a function of temperature: a) derived from NoMoRe refinements of freguencies calculated in CASTEP in *Γ* point ; b) derived directly from freguencies calculated in CASTEP in *Γ* point.

2.8.6. Calculation of ΔH for the II → I Phase Transition

The enthalpy difference (ΔH) for the polymorph II → polymorph I transition was calculated by combining two components:

1- Electronic energy, obtained from periodic DFT calculations using the CRYSTAL program,

2- Vibrational enthalpy, calculated from vibrational frequencies.

Vibrational contributions were evaluated in two ways:

– directly from the DFT-calculated frequencies, and

– from frequencies refined using the NoMoRe method (refining the 8 lowest-energy modes).

This allowed us to estimate the total enthalpy for each polymorph and determine ΔH as the difference between the two forms (see Table 2.8.4).

Table 2.8.4. Transition Enthalpy (for STP or 150°C) and temperature calculated for two sets of frequencies (from DFT calculations and after NoMoRe refinement of 8 first modes) and for different levels of theory.

Level of theory	$\Delta H_{II\to I}$ (STP) / kJ mol^{-1}	$\Delta H_{II\to I}$ (150 °C) / kJ mol^{-1}	Trans. Temp / °C
DFT-calculated frequencies			
6-31G(dp)	14.70	15.05	291
Ahlrichs-VTZP	13.47	13.83	238
HF	10.84	11.20	121
CASTEP	7.31	7.25	113
Frequencies from NoMoRe refinement			
6-31G(dp)	14.65	15.01	420
Ahlrichs-VTZP	13.43	13.78	355
HF	10.80	11.15	215
CASTEP	7.25	7.20	176

2.8.7. Insight into the frequencies and ADPs

In the NoMoRe approach, anisotropic displacement parameters (ADPs) are not refined directly but reconstructed from normal mode vibrations obtained via periodic DFT calculations. Among these modes, low-frequency external vibrations—such as translational and rotational motions of the entire molecule within the crystal lattice—play a key role in shaping the overall displacement pattern of atoms.

These external modes capture how molecules move collectively within their local environment, reflecting lattice dynamics and intermolecular interactions. Including them in the reconstruction ensures that ADPs represent not only the internal vibrations of the molecule but also the influence of its surroundings. This leads to a more physically realistic description of atomic motion, particularly for hydrogen atoms, and should result in improved ADP orientations and anisotropies when compared to traditional refinement approaches (see Figure 2.8.6).

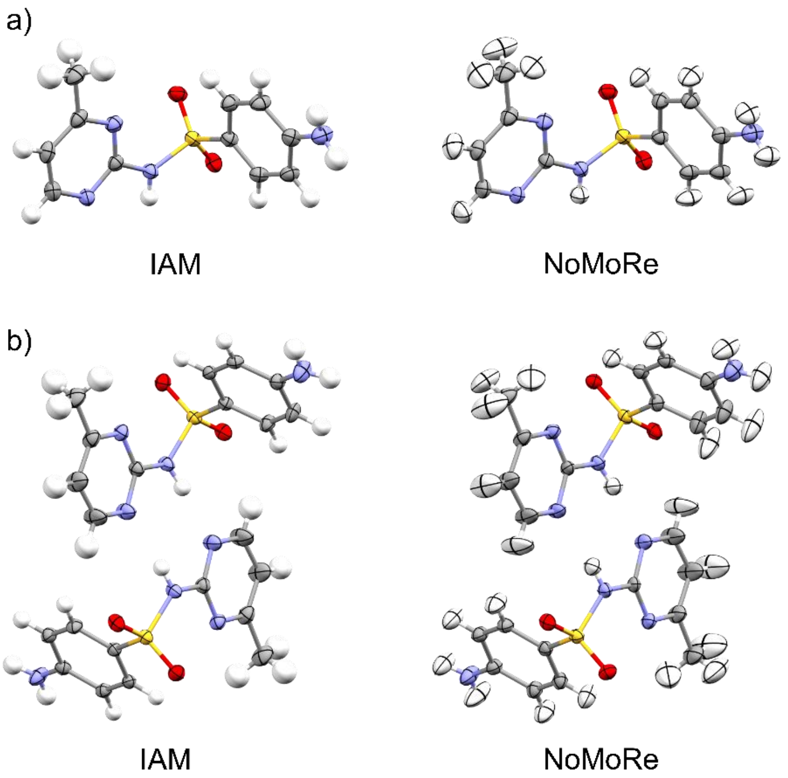

Figure 2.8.6. Thermal ellipsoids (50% probability level) of the molecules in the a) smz_II and b) smz_I polymorphs, refined using IAM and NoMoRe refinement (8 modes).

To compare volumes of non-hydrogen atom ellipsoids we calculated mean value of U_{eq} using

PLATON software[19]. U_{eq} for non-hydrogen atom for Independent Atom model are equal to 0.033 $Å^2$ and 0.034 $Å^2$ for smz_I molecules and 0.028 $Å^2$ for smz_II molecule. Although the values are almost the same, the fact that for smz_I it is slightly higher may indicate entropic stabilization of the form II at a temperature of 150K.

compare volumes of non-hydrogen atom ellipsoids we calculated mean value of U_{eq} using PLATON software[19]. U_{eq} for non-hydrogen atom for Independent Atom model are equal to 0.029 $Å^2$ and 0.030 $Å^2$ for smz_I molecules and 0.028 $Å^2$ for smz_II molecule. Although the values are almost the same, the fact that for smz_I it is slightly higher may indicate entropic stabilization of the form I at a temperature of 150K. Unfortunately, we do not have multitemperature measurements, therefore we cannot see the temperature evolution of ADPs and we can't conduct NoMoRe refinements against multi temperature data sets.

2.8.8. Comment on Refined first eight vibrational modes

CRYSTAL

We tested frequency refinement using different numbers of low-energy modes (3, 6, 8, 11, and 21; see Table 2.8.5 and Table 2.8.6) and selected the 8-mode model for all further analyses. This choice was guided by the fact that it yielded the most reasonable thermodynamic results — particularly for the phase transition temperature — despite some of the refined frequencies exceeding 100 cm^{-1}. Other models, especially those involving a larger number of refined modes, led to inconsistencies or unphysical values, likely due to increased correlations between overlapping low-energy vibrations, which can hinder convergence and introduce artifacts. In contrast, the 8-mode model maintained a balance between incorporating experimental information and preserving physically meaningful vibrational features.

CASTEP

We tested frequency refinement using different numbers of low-energy modes(up to 26).

For every refinement, refinement statistics (R1 and wR) were compared together with standard uncertainties of refined frequencies and covariances. The NoMoRe refinemnt of 5 for SMZ_I and 6 modes for SMZ_II (see Table 2.8.7) ennsures the biggest drop in the refinement statistics with standard uncertainties below 0.1-2%. Thermodynamic properties estimated for SMZ_II from NoMoRe refinements of 6 and 11 modes were almost identical. NoMoRe refinement of larger number of modes (11 or 16) doesn't correct refinement statistics significantly and standard uncertainties of refined frequencies were larger.

Table 2.8.5. Refined frequencies (bolded) from CRYSTAL23 for different models of smz_I.

no.	DFT	3 modes	6 modes	8 modes	11 modes	21 modes
**1**	-31,590	**43,317**	**14,391**	**13,207**	**12,764**	**12,688**
**2**	-7,809	**66,429**	**14,760**	**15,019**	**14,344**	**14,100**
**3**	-5,175	**75,653**	**119,629**	**122,827**	**89,349**	**23,833**
**4**	3,244	3,244	**16,747**	**16,061**	**15,776**	**15,482**
**5**	6,999	6,999	**20,033**	**13,234**	**12,429**	**14,227**
**6**	16,371	16,371	**73,640**	**97,293**	**82,010**	**52,739**
**7**	17,559	17,559	17,559	**105,360**	**104,620**	**60,380**
**8**	17,793	17,793	17,793	**101,785**	**101,360**	**65,365**
**9**	18,889	18,889	18,889	18,889	**113,323**	**62,735**
**10**	25,933	25,933	25,933	25,933	**101,903**	**53,227**
**11**	28,563	28,563	28,563	28,563	**141,146**	**89,265**
**12**	28,592	28,592	28,592	28,592	28,592	**88,719**
**13**	29,823	29,823	29,823	29,823	29,823	**68,528**
**14**	30,629	30,629	30,629	30,629	30,629	**97,109**
**15**	33,553	33,553	33,553	33,553	33,553	**66,745**
**16**	34,055	34,055	34,055	34,055	34,055	**79,670**
**17**	34,281	34,281	34,281	34,281	34,281	**72,154**
**18**	36,270	36,270	36,270	36,270	36,270	**73,458**
**19**	36,552	36,552	36,552	36,552	36,552	**21,168**
**20**	40,867	40,867	40,867	40,867	40,867	**28,803**
**21**	41,846	41,846	41,846	41,846	41,846	**16,109**

Table 2.8.6. Refined frequencies (bolded) from CRYSTAL23 for different models of smz_II.

no.	DFT	3 modes	6 modes	8 modes	11 modes	21 modes
**1**	-9,255	**12,918**	**10,368**	**10,501**	**10,732**	**12,381**
**2**	-7,592	**15,911**	**12,363**	**15,653**	**14,568**	**18,561**
**3**	-7,078	**10,799**	**10,443**	**10,478**	**11,688**	**18,92**
**4**	9,927	9,9272	**12,114**	**11,489**	**12,047**	**12,857**
**5**	14,867	14,867	**75,642**	**49,608**	**64,045**	**39,779**
**6**	27,216	27,216	**36,938**	**61,808**	**80,627**	**68,287**
**7**	28,342	28,342	28,342	**17,253**	**17,009**	**18,006**
**8**	30,665	30,665	30,665	**28,131**	**36,053**	**26,124**
**9**	32,911	32,911	32,911	32,911	**42,178**	**19,846**
**10**	33,635	33,635	33,635	33,635	**19,089**	**74,512**
**11**	38,434	38,434	38,434	38,434	**20,616**	**44,723**
**12**	39,990	39,990	39,990	39,990	39,990	**22,629**
**13**	42,458	42,458	42,458	42,458	42,458	**69,982**
**14**	42,719	42,719	42,719	42,719	42,719	**78,943**
**15**	46,043	46,043	46,043	46,043	46,043	**72,365**
**16**	46,097	46,097	46,097	46,097	46,097	**34,825**
**17**	47,939	47,939	47,939	47,939	47,939	**21,047**
**18**	47,948	47,948	47,948	47,948	47,948	**20,011**
**19**	50,648	50,648	50,648	50,648	50,648	**33,138**
**20**	54,218	54,218	54,218	54,218	54,218	**14,395**
**21**	57,154	57,154	57,154	57,154	57,154	**27,381**

Table 2.8.7. Refined frequencies (bolded) for SMZ_I and SMZ_II from CASTEP with NoMoRe method.

no.	**SMZ_I**	**SMZ_II**
**1**	**115,55**	**11,8**
**2**	**13,1**	**10,28**
**3**	**11,09**	**11,16**
**4**	**19,12**	**17,61**
**5**	**28,3**	**65,18**
**6**	19,22	**16,92**
**7**	21,11	29,42
**8**	25,49	32,59
**9**	26,66	32,69
**10**	28,63	32,91
**11**	28,9	33,63
**12**	30,37	37,51
**13**	31,46	38,15
**14**	32,36	39,22
**15**	32,39	40,02
**16**	34,57	41,79
**17**	36,99	42,36
**18**	37,35	45,59
**19**	37,52	47,1
**20**	39,32	49,17
**21**	41,574	54,77

2.8.9. Conclusion on NoMoRe refinement

Our initial assumption pointed to the limited impact of normal-mode refinement (NoMoRe) on the results for both smz_I and smz_II.

The electronic energy difference, which is obtained directly from CRYSTAL, between the two polymorphs was found to be unexpectedly large — on the order of several kJ/mol — which significantly affects the calculated thermodynamic properties such as ΔH and the phase transition temperature. These deviations from experimental values can thus be attributed to the electronic energy and lattice energy calculations accuracy rather than to the vibrational component. In the case of sulfamethazine, reliable thermodynamic modelling may require not only multi-temperature vibrational refinement, but also a more accurate treatment of the electronic energies. Moreover, we note that a TCG-UNITO research group using the CRYSTAL program reported significantly better agreement with experimental thermodynamic properties. Their approach involved performing harmonic frequency calculations in supercells and applying a global scaling factor to the vibrational frequencies. Although our calculations were limited to the primitive cell and unscaled frequencies, this comparison suggests that both supercell-based modelling and empirical frequency scaling (e.g., as proposed for HF-3c) may

play an important role in improving the reliability of computed thermodynamic parameters. In case of CASTEP calculations electronic energies difference is much smaller than in the case of CRYSTAL. Normal-mode refinement (NoMoRe) of frequencies calculated in Γ point, in CASTEP, gives correct estimates of frequencies in different BZ points. Transition temperature calculated with CASTEP + NoMoRe approch equal to 449 K (176 °C) and is very close to the experimental one.

References

(1) Hoser, A. A.; Madsen, A. Dynamic Quantum Crystallography: Lattice-Dynamical Models Refined against Diffraction Data. I. Theory. Acta Crystallogr. Sect. Found. Adv. 2016, 72, 206–214. https://doi.org/10.1107/S2053273315024699.

(2) Hoser, A. A.; Madsen, A. O. Dynamic Quantum Crystallography: Lattice-Dynamical Models Refined against Diffraction Data. II. Applications to l -Alanine, Naphthalene and Xylitol. Acta Crystallogr. Sect. Found. Adv. 2017, 73 (2), 102–114. https://doi.org/10.1107/S2053273316018994.

(3) Erba, A.; Desmarais, J. K.; Casassa, S.; Civalleri, B.; Donà, L.; Bush, I. J.; Searle, B.; Maschio, L.; Edith-Daga, L.; Cossard, A.; Ribaldone, C.; Ascrizzi, E.; Marana, N. L.; Flament, J.-P.; Kirtman, B. CRYSTAL23: A Program for Computational Solid State Physics and Chemistry. J. Chem. Theory Comput. 2023, 19 (20), 6891–6932. https://doi.org/10.1021/acs.jctc.2c00958.

(4) Dovesi, R.; Saunders, V. R.; Roetti, C.; Orlando, R.; Zicovich-Wilson, C. M.; Pascale, F.; Civalleri, B.; Doll, K.; Harrison, N. M.; Bush, I. J.; D'Arco, P.; Llunell, M.; Causà, M.; No&eul, Y.; Maschio, L.; Erba, A.; Rerat, M.; Casassa, S.; Searle, B. G.; Desmarais, J. K. CRYSTAL23 User's Manual. 2023.

(5) Becke, A. D. Density-Functional Thermochemistry. III. The Role of Exact Exchange. J. Chem. Phys. 1993, 98 (7), 5648–5652. https://doi.org/10.1063/1.464913.

(6) Lee, C.; Yang, W.; Parr, R. G. Development of the Colle-Salvetti Correlation-Energy Formula into a Functional of the Electron Density. Phys. Rev. B 1988, 37 (2), 785–789. https://doi.org/10.1103/PhysRevB.37.785.

(7) Civalleri, B.; Zicovich-Wilson, C. M.; Valenzano, L.; Ugliengo, P. B3LYP Augmented with an Empirical Dispersion Term (B3LYP-D*) as Applied to Molecular Crystals. CrystEngComm 2008, 10 (4), 405–410. https://doi.org/10.1039/B715018K.

(8) Grimme, S.; Antony, J.; Ehrlich, S.; Krieg, H. A Consistent and Accurate Ab Initio Parametrization of Density Functional Dispersion Correction (DFT-D) for the 94 Elements H-

Pu. J. Chem. Phys. 2010, 132 (15). https://doi.org/10.1063/1.3382344.
(9) Madsen, A. Ø.; Hoser, A. A. SHADE3 Server: A Streamlined Approach to Estimate H-Atom Anisotropic Displacement Parameters Using Periodic Ab Initio Calculations or Experimental Information. J. Appl. Crystallogr. 2014, 47 (6), 2100–2104. https://doi.org/10.1107/S1600576714022973.
(10) Clark, S. J.; Segall, M. D.; Pickard, C. J.; Hasnip, P. J.; Probert, M. I. J.; Refson, K.; Payne, M. C. First Principles Methods Using CASTEP. Z. Für Krist. - Cryst. Mater. 2005, 220 (5–6), 567–570. https://doi.org/10.1524/zkri.220.5.567.65075.
(11) Baroni, S.; De Gironcoli, S.; Dal Corso, A.; Giannozzi, P. Phonons and Related Crystal Properties from Density-Functional Perturbation Theory. Rev. Mod. Phys. 2001, 73 (2), 515–562. https://doi.org/10.1103/RevModPhys.73.515.
(12) Aree, T.; Bürgi, H.-B. Specific Heat of Molecular Crystals from Atomic Mean Square Displacements with the Einstein, Debye, and Nernst−Lindemann Models. J. Phys. Chem. B 2006, 110 (51), 26129–26134. https://doi.org/10.1021/jp0636322.
(13) Sovago, I.; Hoser, A. A.; Madsena, A. A Combined Model of Electron Density and Lattice Dynamics Refined against Elastic Diffraction Data. Thermodynamic Properties of Crystalline l-Alanine Sovago Ioana. Acta Crystallogr. Sect. Found. Adv. 2020, 76, 32–44. https://doi.org/10.1107/S205327331901355X.
(14) Hoser, A. A.; Sztylko, M.; Trzybiński, D.; Madsen, A. Theoretically Derived Thermodynamic Properties Can Be Improved by the Refinement of Low-Frequency Modes against X-Ray Diffraction Data. Chem. Commun. 2021, 57 (74), 9370–9373. https://doi.org/10.1039/d1cc02608a.
(15) Butkiewicz, H.; Chodkiewicz, M.; Madsen, A. Ø.; Hoser, A. A. Advancing Dynamic Quantum Crystallography: Enhanced Models for Accurate Structures and Thermodynamic Properties. IUCrJ 2025, 12 (1), 123–136. https://doi.org/10.1107/S2052252524011862.
(16) Hoser, A. A.; Rekis, T.; Madsen, A. Ø. Dynamics and Disorder: On the Stability of Pyrazinamide Polymorphs. Acta Crystallogr. Sect. B Struct. Sci. Cryst. Eng. Mater. 2022, 78 (3), 416–424. https://doi.org/10.1107/S2052520622004577.
(17) Kofoed, P. M.; Hoser, A. A.; Diness, F.; Capelli, S. C.; Madsen, A. Ø. X-Ray Diffraction Data as a Source of the Vibrational Free-Energy Contribution in Polymorphic Systems. IUCrJ 2019, 6 (4), 558–571. https://doi.org/10.1107/S2052252519003014.
(18) Hoser, A. A.; Rekis, T.; Butkiewicz, H.; Bērziņš, K.; Larsen, A. S.; Bosak, A.; Boyd, B. J.; Madsen, A. Ø. Phase Transition in the Jumping Crystal l -Pyroglutamic Acid: Insights from Dynamic Quantum Crystallography and Spectroscopy. Cryst. Growth Des. 2025, 25 (3),

593–602. https://doi.org/10.1021/acs.cgd.4c01335.
(19) Spek, A. L. Single-Crystal Structure Validation with the Program PLATON. J. Appl. Crystallogr. 2003, 36 (1), 7–13. https://doi.org/10.1107/S0021889802022112.

2.9. AMS: Free energy calculations with TRHuST

Contributed by Dzmitry Firaha

For the full method description, please, refer to the original publication of Firaha et al.[1] Below is a concise description of the phonons free energy calculations part.

The harmonic phonon free energies were obtained using the second-order dynamical matrix at the PBE-NP/light level of theory in FHI-aims. These calculations provide the vibrational free energy within the harmonic approximation, which assumes that each vibrational mode behaves as a non-interacting quantum harmonic oscillator. This framework captures the main temperature-dependent vibrational contributions to crystal free energies. However, molecular crystals often deviate from harmonic behavior in both the low- and high-frequency regions. To address these deviations, several corrections were introduced:

2.9.1. Imaginary mode correction

For imaginary phonon modes, one-dimensional potential energy sampling was carried out along the normalized eigenvector. A fourth-order polynomial was then fitted to the sampled potential energy values, and the energy levels were obtained from the numerical solution of the Schrödinger equation for this potential. These energy levels were subsequently used to evaluate the free energy contribution.

2.9.2. Very soft mode correction

When the frequency of an eigenmode approaches zero cm^{-1}, its contribution to the free energy tends toward negative infinity. To correct this, the same protocol as for the imaginary mode correction was applied to all non-acoustic modes from 0 up to 25 cm^{-1}. In the range from 15 to 25 cm^{-1}, a fifth-order spline function was employed to ensure a smooth transition to the harmonic approximation.

2.9.3. Methyl top correction

Methyl group rotations were modeled as hindered rotors rather than simple harmonic oscillators, thereby capturing their true entropic contribution.

2.9.4. Hydrogen-bond correction

Hydrogen-bond stretches deviate significantly from harmonic behavior. All hydrogen-bonded atoms were treated separately to correct their vibrational free-energy contributions. A 3×3 dynamical matrix of a hydrogen-bonded hydrogen atom was used to compute the corresponding eigenvalues and eigenvectors. The largest eigenvalue and its associated eigenvector were used in the correction scheme as follows: the proton was displaced twice along this eigenvector, in both the positive and negative directions, with a step of 0.01 Å. Subsequently, the same protocol as for the imaginary and very soft modes correction was applied to compute the free energy contributions from the resulting energy levels. Unlike the imaginary and very soft mode corrections, this adjustment primarily affects the zero-point vibrational energy component of the free energy.

2.9.5. Large cell correction

At the Γ-point, the three acoustic modes are exactly zero. These modes together with other low laying modes show the strongest dependence on the lattice k-vector. Running the *ab initio* calculations with small supercells, this introduces systematic errors. To correct this systematic error, a large-cell correction was applied by computing the free energy difference between large and small supercells. The large supercell was constructed with a minimum lattice point distance of 24 Å between symmetry copies, while the small supercell had a minimum distance of 8 Å.

Table 2.9.1. Relative Helmholz free energy, relative lattice energy, harmonic vibrations, all anharmonic corrections and individual anharmonic components at 298.15 K in kJ mol^{-1} calculated by TRHuST 23. The relative lattice energies are computed on the energy minimized PBE-NP/light structures with PBE0/light, PBE/tight and PBE-MBD-nl/light single point corrections and with single molecule MP2D/NAO-VCC-4Z correction.

	Rel. Helmholz Free Energy, kJ/mol	Rel. Lattice Energy, kJ/mol	Harmonic Vibrations, kJ/mol	All anharmonic corrections, kJ/mol	Imaginary mode corr., kJ/mol	Very soft mode corr., kJ/mol	Hydrogen bond anharmonicity corr. , kJ/mol	Methyl top corr, kJ/mol	Large cell corr. kJ/mol
**Form I**	**0.34**	**5.32**	**544.41**	**-3.28**	**0.00**	**0.24**	**-0.77**	**-0.40**	**-2.34**
**Form II**	**0.61**	**0.00**	**550.44**	**-3.71**	**0.00**	**0.11**	**-0.84**	**-0.15**	**-2.84**
Form III	0.50	7.97	546.21	-7.57	0.00	0.19	-0.81	-0.82	-6.13
Form IV	0.28	2.10	547.90	-3.61	-0.12	0.51	-0.87	-0.08	-3.05
**Form V**	**0.00**	**5.39**	**544.35**	**-3.63**	**0.00**	**0.25**	**-0.77**	**-0.43**	**-2.68**

All five corrections address specific shortcomings of the harmonic phonon free energy approximation, yielding reliable free energy estimates across temperatures. The difference in the very soft mode anharmonicity correction between Form I and Form II is only 0.13 kJ mol^{-1} at 298.15 K according to the TRHuST 23 method. The hydrogen-bond anharmonicity correction is almost two times smaller, amounting to 0.07 kJ mol^{-1}, while the methyl-top and large-cell correction differences are –0.25 and 0.49 kJ mol^{-1}, respectively. This hybrid approach balances accuracy and computational efficiency, enabling quantitative stability predictions of molecular crystal forms under real-world conditions.

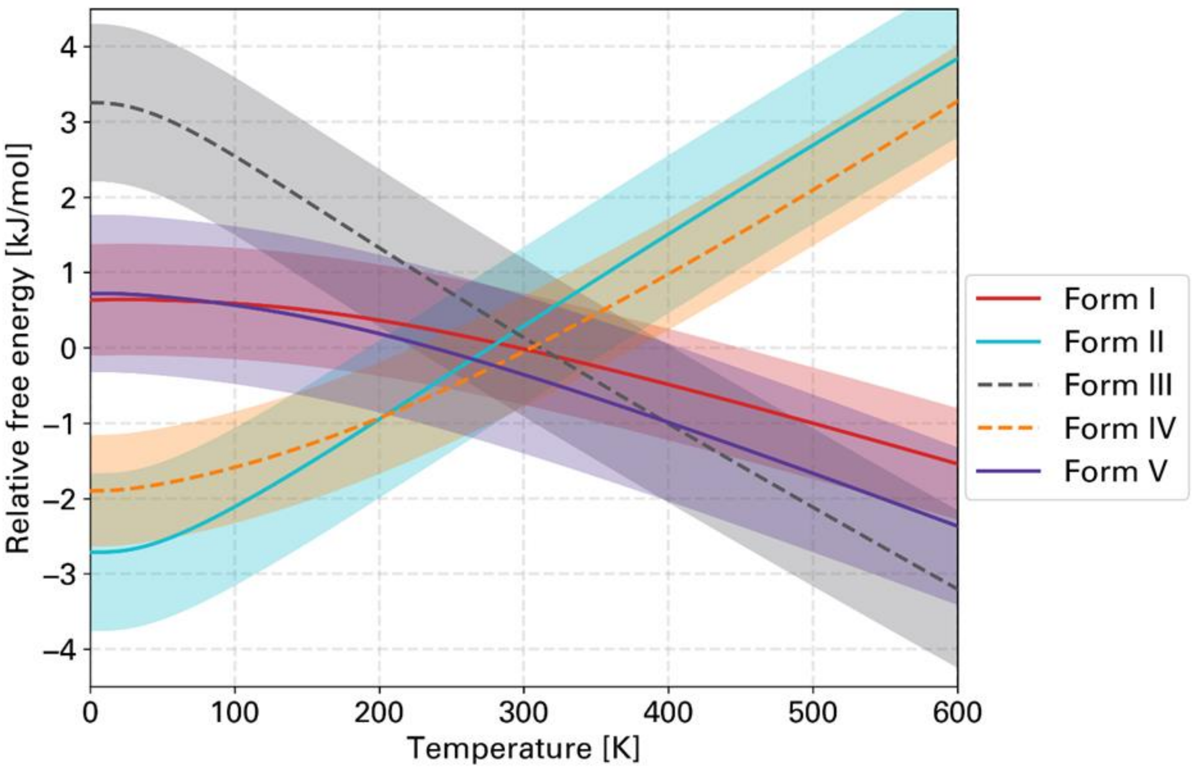

Figure 2.9.1. The relative Helmholtz free energies calculated by TRHuST 23 are plotted with the average over the polymorphs shown at each temperature being used to define the zero. The estimated errors in the computational results are shown as stripes.

2.9.6. Sulfamerazine Forms: Free Energies with the TRHuST 23 Method

Free-energy calculations using the TRHuST 23 method suggest the following stability sequence of sulfamerazine polymorphs. Form II is predicted to be the most stable thermodynamic form below 240 K. Between 240 K and 275 K, Form IV is expected to

dominate, while in the range of 275–397 K, Form V becomes the most stable. Above 397 K and up to the melting point, Form III is predicted to be the most stable phase.

The results further indicate that Form I remains metastable with respect to Form V above 85 K. A Form II → Form V transition is expected around 265 K, while a Form II → Form I transition should occur at 282 K. The sequence of phase transitions and the corresponding temperature ranges are in good agreement with experimental solubility measurements.

At 13 K below the theoretical transition temperature of 265 K (Form II → Form V), the calculated free-energy differences are: I–II = 0.49, I–V = 0.26, V–II = 0.24 kJ·mol^{-1}. For comparison, the experimental solubility data give: I–II = 0.198, I–V = 0.095, V–II = 0.103 kJ·mol^{-1}.

References

[1] Firaha, D.; Liu, Y. M.; van de Streek, J.; Sasikumar, K.; Dietrich, H.; Helfferich, J.; Aerts, L.; Braun, D. E.; Broo, A.; DiPasquale, A. G.; et al. Predicting crystal form stability under real-world conditions. *Nature (London)* **2023**, *623* (7986), 324-328. DOI: 10.1038/s41586-023-06587-3.

2.10. CB@Lisbon Molecular Dynamics Simulations

Contributed by Rute I. S. Rodrigues and Carlos E. S. Bernardes

The enthalpy of sublimation, temperature of fusion, and enthalpic difference between the sulfamerazine polymorphs were obtained from molecular dynamics (MD) simulations of the solid, liquid, and gaseous phases at different temperatures. Unless otherwise stated: (*i*) All calculations were performed using periodic boundary conditions, using the particle-particle particle-mesh Ewald method[1] to compute electrostatic interactions beyond a cutoff of 15 Å. (*ii*) The simulations were run at temperatures (T) in the range of 230 – 510 K and at a pressure (p) of 1 bar. To achieve this, a Nosé-Hoover thermostat (2 ps time constant) and a barostat (20 ps time constant) were employed to perform simulations under the $N\sigma T$, NPT, and NVT ensembles for the solid, liquid, and gaseous phases, respectively. (*iii*) A 2 fs timestep was used for all simulations. (*iv*) The MD computations were performed with LAMMPS (version 2, Aug 2023),[2] using input files generated with the program DLPGEN 3.0.[3] (*v*) All reported errors for the MD simulation data correspond to the standard deviation of the computed values.

The configurational internal energies were computed from:

$$U_{\text{cfg}} = \sum^{\text{bonds}} \frac{k_b}{2}\left(r - r_o\right)^2 + \sum^{\text{angles}} \frac{k_\theta}{2}\left(\theta - \theta_o\right)^2 + \sum^{\text{dihedrals}} \sum_{n=1}^{4} \frac{V_n}{2}\left[1 + (-1)^{(n-1)} \cos\left(n\varphi\right)\right] +$$
$$\sum_i \sum_{j>i} \frac{q_i q_j}{4\pi\varepsilon_o r_{ij}} + 4\varepsilon_{ij}\left[\left(\frac{\sigma_{ij}}{r_{ij}}\right)^{12} - \left(\frac{\sigma_{ij}}{r_{ij}}\right)^{6}\right] \quad (1)$$

where r_o and q_o are the equilibrium bond distances and angles in the molecule, respectively; k_b and k_q, are the force constants of the harmonic oscillators associated with bond and angle vibrations; V_n are the coefficients of a Fourier series that models the internal rotation as a function of the dihedral angles, j, in the molecule; e_o is the vacuum permittivity; q_i and q_j correspond to atomic point charges (APCs); and e_{ij} and s_{ij} are the parameters of the 12-6 Lennard-Jones (LJ) potential.

The parametrization used in the simulations was prepared as follows:

(*i*) The Lennard-Jones (LJ) parametrization was taken from the OPLS-AA force field, as this was found to be suitable for the simulation of sulfonamide compounds.[4]

(*ii*) The atomic point charges were computed from DFT calculations, using the program ORCA 6.0,[5] at the PBE-D3BJ/aug-cc-pVDZ level of theory.[6-9] For this, 300 dimers of sulfamerazine were initially prepared by placing two randomly oriented molecules at distances between 2.9 and 4.0 Å. After performing a single-point energy calculation for each dimer, the obtained wavefunction was used to compute the atomic point charges of the two molecules using the ChelpG[10] method. The final atomic point charges corresponded to the average of the computed values obtained for all molecules in all dimers.

(*iii*) The parameters used to describe the bonds, angles, and dihedrals were computed from ab initio molecular dynamics (AIMD) simulations. Initially, an MD run was performed with ORCA 6.0[5] at the B97M-D4/aug-cc-pVTZ[9, 11-13] level of theory for a single molecule in the gas phase. The simulation was run at 1000 K using a Berendsen thermostat for 1000 steps of 0.5 fs each. During the simulation, both the electronic energy and the forces on each atom were recorded at every step. The resulting data were then fitted to Eq. 1 using an evolutionary algorithm by minimizing the RMSD between the forces and energies obtained with the model and those from the AIMD results.

The force field parametrization, along with the data files used in the simulations necessary to reproduce the results presented here, can be downloaded from

https://doi.org/10.5281/zenodo.16948976. The additional simulation conditions and data analysis procedures are described below.

a) Solid Phase Studies. The simulation boxes for the solid forms were prepared from single-crystal X-ray diffraction data retrieved from the Cambridge Structural Database (CSD refcodes: SLFNMA02-form I, and SLFNMA01-form II)[14] or determined in this work. To attain boxes compatible with the cutoff (15 Å), several unit cells were stacked along the three coordinate axes to produce approximately cubic boxes with sides of 40–50 Å: 3×2×5 for form I, 6×5×3 for form II, and 2×5×3 for form V. The simulation boxes were equilibrated by heating the initial configuration from 1 K to the target temperature during a simulation run of 1 ns. After this process, a production run of 2 ns was conducted at the final temperature, recording the energetic, structural, and box conformation data every 2 ps. If necessary, the system temperature was then increased to a new value during an additional 1 ns stage, followed by a new production run.

b) Liquid Phase Studies. A box for the liquid phase simulation was prepared by randomly distributing 500 molecules of SMZ to create a system with an initial density of 0.5 g cm^{-3}. The box was then equilibrated using the following steps, during which the temperature and pressure were sequentially adjusted: (*i*) 1 ns at T = 550 K and p = 100 bar; (*ii*) 5 ns at T = 550 K and p = 1 bar; (*iii*) 5 ns at T = 509.35 K and p = 1 bar. After this procedure, the temperature and density of the system were approximately constant, indicating that it was in equilibrium. Subsequently, a production run of 5 ns was conducted at 509.35 K (the experimentally determined fusion temperature for SMZ form I).

c) Gas Phase Studies. To obtain the configurational energy of sulfamerazine in the gaseous phase, $U^{\mathrm{o}}_{\mathrm{conf,m}}(\mathrm{g})$, a simulation was performed using a cubic box of 300 Å containing a single molecule. Due to the limited statistical sampling in such a setup, 20 independent MD runs of 20 ns each were conducted under the *NVT* ensemble at 298.15 K. A plain cutoff of 50 Å for the Coulomb and van der Waals (VDW) interactions was applied to ensure that all intramolecular interactions were captured during the simulations. The final $U^{\mathrm{o}}_{\mathrm{conf,m}}(\mathrm{g})$ result corresponded to the average value obtained from the 20 simulations.

d) Structure and Energetics. The standard molar enthalpies of sublimation, $\Delta_{\mathrm{sub}}H^{\mathrm{o}}_{\mathrm{m}}$, were

computed from:

$$\Delta_{\text{sub}} H_{\text{m}}^{\text{o}} = U_{\text{conf,m}}^{\text{o}}(\text{g}) - U_{\text{conf,m}}^{\text{o}}(\text{cr}) + RT \qquad (2)$$

where $U_{\text{conf,m}}^{\text{o}}(\text{g})$ and $U_{\text{conf,m}}^{\text{o}}(\text{cr})$ represent the standard molar configurational internal energies in the gas and solid phases, respectively (obtained from the calculations described in Sections **a** and **c**), R = 8.3144626 J K^{-1} mol^{-1} [15] is the gas constant, and T = 298.15 K. A comparison between the computed and experimental unit cell dimensions, along with the corresponding computed $\Delta_{\text{sub}} H_{\text{m}}^{\text{o}}$ and $U_{\text{conf,m}}^{\text{o}}(\text{cr})$ values, is presented in typical level of accuracy for this type of theoretical prediction.[16] The exception to this trend was noticed for form V, for which a distortion of the unit cell relative to experiment was noticed. This occurs because an anisotropic barostat was used in these simulations, and, as a result, any imbalance in the force field (such as the LJ parametrization, which was not refined) can easily distort the simulation box. Still, the obtained data are within what is expected from MD simulations.[16]

Table 2.10.1 From the data in the table, it is possible to conclude that most of the unit cell dimensions are reproduced with deviations from experiment of less than 4%, which is a typical level of accuracy for this type of theoretical prediction.[16] The exception to this trend was noticed for form V, for which a distortion of the unit cell relative to experiment was noticed. This occurs because an anisotropic barostat was used in these simulations, and, as a result, any imbalance in the force field (such as the LJ parametrization, which was not refined) can easily distort the simulation box. Still, the obtained data are within what is expected from MD simulations.[16]

Table 2.10.1. Comparison between computed and experimental crystal unit cell dimensions (*a, b, c, α, β*, and *γ*) at different temperatures, *T*. $U^{o}_{conf,m}(cr)$ and $\Delta_{sub}H^{o}_{m}$ refer to the standard molar configurational internal energies and the enthalpy of sublimation of the solid phases, respectively. *r* is the density. Values in parentheses indicate the deviation, in percent, between experiment and theory.

Method	T /K	$U^{o}_{conf,m}(cr)$ / kJ mol^{-1}	$\Delta_{sub}H^{o}_{m}$ / kJ mol^{-1}	*a* /Å	*b*/Å	*c*/Å	α /º	β /º	γ /º	ρ /g cm^{-3}
**Form I**										
Experiment: SLFNMA02	298.15			14.474	21.953	8.203	90	90	90	1.347
MD	298.15	-595.15±0.71	159.9±4.5	14.153 (-2.2)	21.325(-2.9	8.556(4.3)	90.02(0.0)	88.4(-1.8)	90.00(0.0)	1.360(1.0)
	423.15	-548.51±1.12		13.860	22.500	8.600	90.45	89.05	90.22	1.309
**Form II**										
Experiment: SLFNMA01				9.145	11.704	22.884	90	90	90	1.434
MD	298.15	-594.85±0.71	159.0±4.5	9.266(1.3)	12.147(3.8)	22.404(2.1)	90.00(0.0)	90.00(0.0)	90.00(0.0)	1.392(-2.9)
	423.15	-550.04±0.72		9.386	12.459	22.236	90.00	90.00	90.00	1.350
**Form V**										
Experiment (this work)	150.00			22.735	8.187	14.458	90.0	106.7	90.0	1.362
	298.15			23.257	8.201	14.454	90.0	109.3	90.0	1.350
MD	150			22.303(-1.9)	8.515(4.0)	14.074(-2.7)	89.95(-0.1)	110.10(3.2)	96.28(7.0)	1.408(3.4)
	298.15	-595.51	160.3±4.5	22.634(-2.7)	8.543(4.2)	14.201(-1.8)	89.72(-0.3)	109.69(0.3)	94.53(5.0)	1.363(1.0)

From the results in typical level of accuracy for this type of theoretical prediction.[16] The exception to this trend was noticed for form V, for which a distortion of the unit cell relative to experiment was noticed. This occurs because an anisotropic barostat was used in these simulations, and, as a result, any imbalance in the force field (such as the LJ parametrization, which was not refined) can easily distort the simulation box. Still, the obtained data are within what is expected from MD simulations.[16]

Table 2.10.1, the enthalpic difference between the polymorphs of sulfamerazine was computed as:

$$\Delta_{trs}H_{m}^{o}(II \rightarrow I) = U_{conf,m}^{o}(cr\ I) - U_{conf,m}^{o}(cr\ II) \quad (3)$$

$$\Delta_{trs}H_{m}^{o}(V \rightarrow I) = U_{conf,\ m}^{o}(cr\ I) - U_{conf,\ m}^{o}(cr\ V) \quad (4)$$

$$\Delta_{trs}H_{m}^{o}(V \rightarrow II) = U_{conf,m}^{o}(cr\ II) - U_{conf,m}^{o}(cr\ V) \quad (5)$$

Table 2.10.2. Enthalpy of transition values between SMZ polymorphs, obtained from the MD simulation data in typical level of accuracy for this type of theoretical prediction.[16] The exception to this trend was noticed for form V, for which a distortion of the unit cell relative to experiment was noticed. This occurs because an anisotropic barostat was used in these simulations, and, as a result, any imbalance in the force field (such as the LJ parametrization, which was not refined) can easily distort the simulation box. Still, the obtained data are within what is expected from MD simulations.[16]

Table 2.10.1, and experimentally obtained in this work.

Transition	Temperature / K	$\Delta_{trs}H^{o}_{m}$ / kJ mol^{-1}
Experimental		
II→I	298.15	3.94±0.74
	423.15	3.07±0.72
MD		
II→I	298.15	−0.9±1.7
	423.15	1.5±2.7
V→I	298.15	0.4±2.0
V→II	298.15	1.3±2.0

The results in Table 2.10.2 show that for the II→I phase transition, the experimental enthalpic difference order between the two phases is not reproduced at room temperature. Still, considering the calculation error, this process can be predicted as an endothermic process, as experimentally observed. In turn, the obtained value at the transition temperature (T = 423.15 K) reproduces the DSC experimental results within the calculation errors. The latter result is, however, affected by a unit cell change in form I, as discussed below.

The enthalpy of fusion of sulfamerazine at T = 509.35 K (the experimentally determined melting temperature for form I) was computed from the results obtained in Sections **a** and **b** using:

$$\Delta_{fus}H^{o}_{m} = U^{o}_{conf,m}(l) - U^{o}_{conf,m}(cr) + p\Delta V \approx U^{o}_{conf,m}(l) - U^{o}_{conf,m}(cr) \quad (6)$$

where $U^{o}_{conf,m}(l)$ is the standard molar configurational internal energy of the liquid phase. The results at T = 509.35 K were:

$\Delta_{fus}H^{o}_{m}(\text{cr I})$ = (-483.87±1.07) – (-515.95±1.29) = 32.1±1.5 kJ mol^{-1}

$\Delta_{fus}H^{o}_{m}(\text{cr II})$ = (-483.87±1.07) – (-516.54±0.83) = 32.7±1.4 kJ mol^{-1}

The result for form I is in fair agreement with the experimental value of

$\Delta_{fus}H^{o}_{m}(\text{cr I})$ = 39.8 ± 2.0 kJ mol^{-1}.

The computed unit cell dimensions as a function of temperature for SMZ forms I and II are shown in

Table 2.10.3. Figure 2.10.1 displays a comparison of the variation of the experimental and computed unit cell dimensions as a function of temperature relative to the crystal structure at room temperature (~298 K). Finally, Figure 2.10.2 shows a comparison between the experimental and theoretical variation of the density as a function of temperature.

The results in

Table 2.10.3 show that, while the dimensions of the unit cell parameters of form II vary smoothly up to the melting temperature range, the same is not true for form I. In the latter case, a discontinuity is noticed at $T \sim 420$ K, suggesting a phase transition. Because an II→I phase transition was only experimentally observed, this suggests a limitation in the model used in this work. Further evidence of this is noticed in Figure 2.10.1, which reveals that, for both forms, the thermal expansion of the crystal is not accurately captured. Even so, the crystal density variation as a function of temperature and density order between the phases is reasonably captured by the simulations (Figure 2.10.2). These results suggest, therefore, that the model used in this work, although adjusted for the simulation of SMZ, may still need to be improved, or that the intricacies of the interactions in solid state can only be modeled to a certain point using such a simple model as that given by Eq. 1.

Table 2.10.3. Unit cell dimensions (*a*, *b*, *c*, *a*, *b*, and *g*) and solid density (*r*) at different temperatures, *T*, obtained from the molecular dynamics simulations of form I and II.

T /K	*a* /Å	*b* /Å	*c* /Å	*α* /°	*β* /°	*γ* /°	*ρ* /g cm^{-3}
				**Form I**			
230.00	14.099	21.102	8.543	90.00	88.46	90.00	1.382
250.00	14.116	21.164	8.547	89.99	88.43	90.01	1.376
298.15	14.153	21.325	8.556	90.02	88.35	90.00	1.360
330.00	14.176	21.447	8.563	90.00	88.33	90.00	1.349
360.00	14.196	21.563	8.570	89.98	88.30	90.00	1.339
**390.00**	**14.215**	**21.680**	**8.578**	**89.98**	**88.26**	**90.00**	**1.329**
**423.15**	**13.860**	**22.500**	**8.600**	**90.45**	**89.05**	**90.22**	**1.309**
450.00	13.635	23.032	8.614	89.94	89.85	89.98	1.298
480.00	13.655	23.127	8.628	90.04	89.96	90.03	1.289
509.35	13.709	23.161	8.641	90.07	89.99	90.01	1.280
				**Form II**			
230.00	9.196	12.013	22.502	90.00	90.00	90.00	1.412
250.00	9.217	12.050	22.473	90.00	90.00	90.00	1.407
298.15	9.266	12.147	22.404	90.00	90.00	90.00	1.392
330.00	9.297	12.218	22.358	90.00	90.00	90.00	1.383
360.00	9.326	12.289	22.318	90.00	90.00	90.00	1.373
390.00	9.355	12.368	22.276	90.00	90.00	90.01	1.362
423.15	9.386	12.459	22.236	90.00	90.00	90.00	1.350
450.00	9.412	12.540	22.202	90.00	90.00	90.01	1.340
480.00	9.440	12.636	22.174	90.00	90.00	90.00	1.327
509.35	9.471	12.733	22.156	90.01	89.99	90.03	1.314

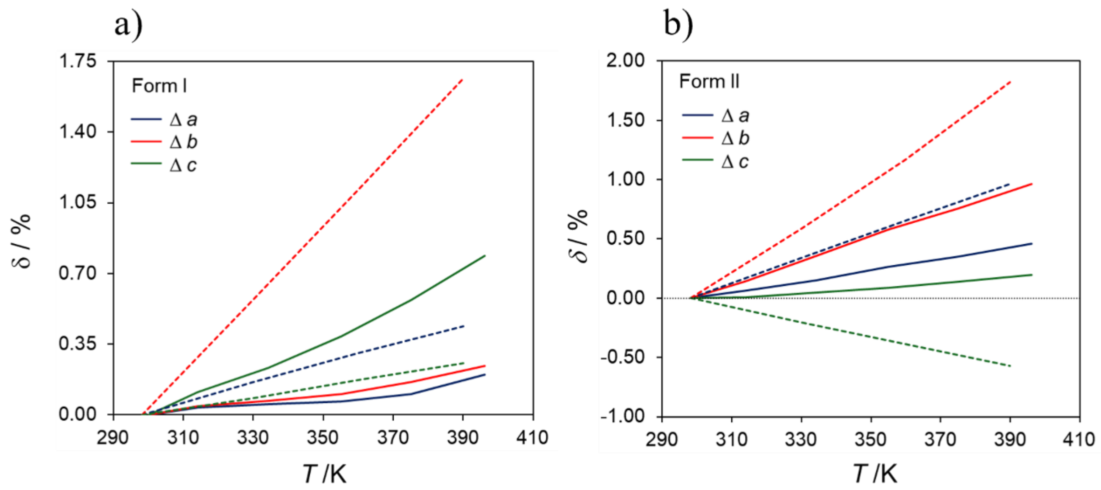

Figure 2.10.1. Comparison of the variation (d) of the unit cell dimensions as a function of temperature relative to the structure at room temperature (~298 K) for (a) form I and (b) form II. The solid lines correspond to the experimental results, while the dashed lines are computational data.

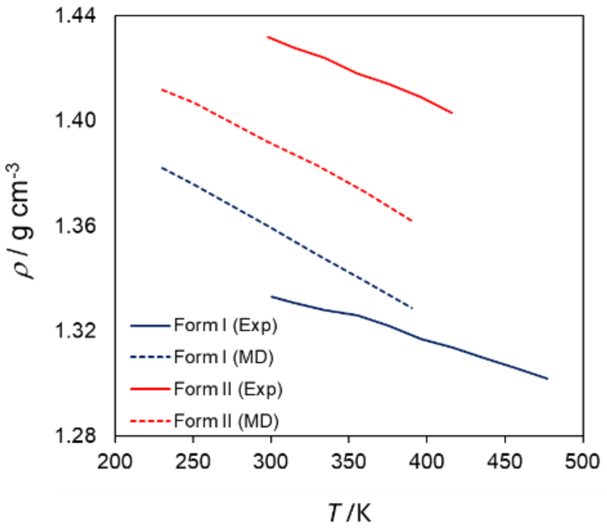

Figure 2.10.2. Comparison between the experimental and theoretical variation of the crystal density as a function of temperature for forms I and II. The solid lines correspond to the experimental results, while the dashed lines are computational data.

e) Melting Temperature. The fusion temperature of a compound is a challenging property to evaluate from MD simulations due to the difficulty in ensuring equilibrium conditions between the two phases (solid and liquid). In this work, a new approach was tested to compute this property, based on simulating crystallites placed in a vacuum. The rationale behind this method is that if the crystal used in the simulation has a sufficiently large number of molecules, at a given temperature, those found at the solid–vacuum interface can begin to melt, while those at

the core stay crystalline (similarly to what is seen experimentally). Consequently, it is possible to achieve a quasi-equilibrium state between the solid and liquid phases that mimics experiment and, therefore, obtain an estimate of the melting temperature of the material from a conventional MD run (within the limitations of the model).

To obtain each crystallite, the crystallographic files for the two polymorphs were opened in Mercury,[17] and the unit cell was packed to mimic, as closely as possible, the BFDH morphology predicted for these solids. The crystallite particles had the following characteristics: Form I (SLFNMA02), 10,500 molecules and 315000 atoms; Form II (SLFNMA01), 6108 molecules and 183240 atoms. Note that Mercury's BFDH morphology routine was not used because it is not capable of generating structures of the size considered in this work. The simulations were performed by placing the crystallites in a much larger box than the size of the crystals to approximate vacuum conditions (a cubic box with a side length of 300 Å). Then, using an *NVT* ensemble, the system was heated from 1 K to 600 K in steps of 5 K. At each temperature, a 1 ns equilibration was performed before a production run of the same duration. Snapshots of this procedure showing that the crystallite retains its structure up to the melting temperature are given as an example for form II in Figure 2.10.3. Due to the inability to use Ewald corrections under these conditions, an interaction cutoff of 20 Å was selected, and the Coulomb interactions were computed using a shift potential. These simulations were run with GROMACS 2024.1,[18,19] using input files generated with DLPGEN 3.0.[3]

To evaluate the melting point, the system's internal energy was plotted as a function of temperature, Figure 2.10.4. This Figure shows that, around the fusion temperature, a step in the energy values occurs (as observed experimentally). Based on the onset temperature of this process, the fusion temperatures of SMZ forms I and II were estimated as T_{fus}(cr I) = 519.4 K and T_{fus}(cr II) = 488.2 K, respectively. These results are in fair agreement with the experimental fusion temperature, T_{fus}(cr I) = 509.35 K, and that T_{fus}(cr I) > T_{fus}(cr II).

It should finally be noted that in the internal energy variation of form II, no solid-solid phase transitions were detected until fusion. This indicates that the II→I phase transition is unlikely to occur under these simulation conditions or, as discussed above, the current model can't capture this process.

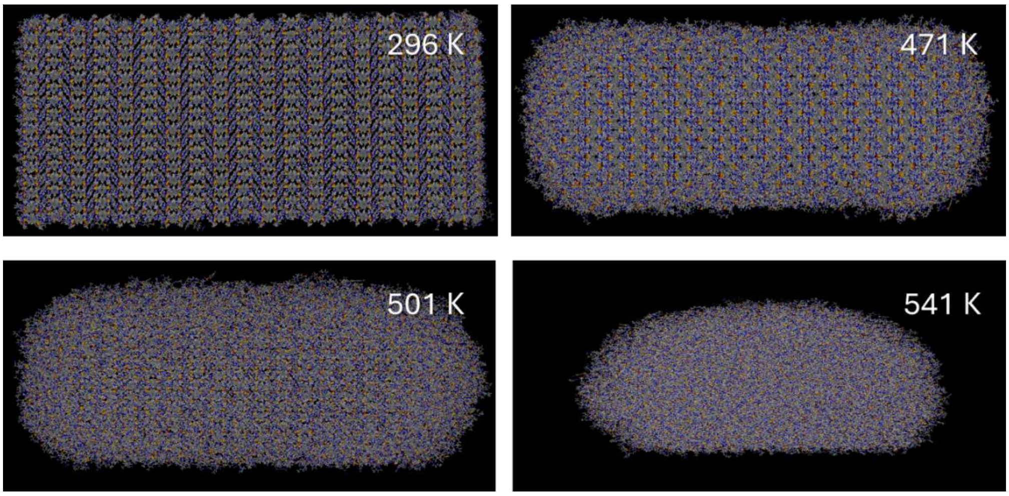

Figure 2.10.3. Snapshots of the crystallite of form II at different temperatures.

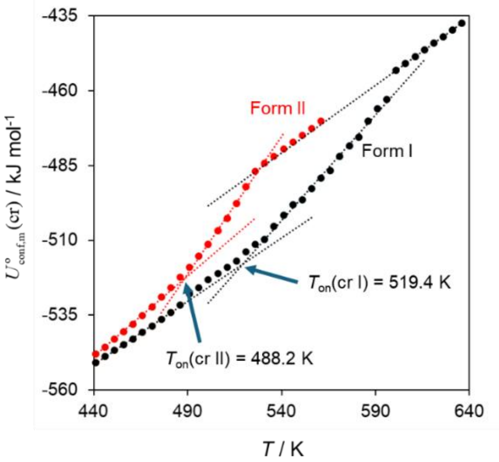

Figure 2.10.4. Variation of the standard molar configurational internal energy as a function of temperature for Form I (black dots) and II (red dots) in the vicinity of the melting temperature region.

e) Conformational Flexibility. To gain a molecular insight into the experimentally observed II→I phase transition, the angular distributions of selected SMZ dihedral angles were investigated as a function of temperature for form I and II based on the MD simulation trajectories (Figure 2.10.5). The results show that, with increasing temperature, no significant changes are observed in the dihedrals distribution (apart from the predictable enhanced thermal motion) except for those involving the aniline ring (note that the methyl group also undergoes hindered rotation at all investigated temperatures, although this is not shown in the Figure). In the latter case, the following conclusions can be drawn:

(*i*) The NH_2 group is essentially frozen at low temperatures, but its mobility increases with temperature (Figure 2.10.5e-f). This effect is more pronounced in form II than in form I, indicating a larger mobility of this group in phase II. This will, therefore, facilitate the breaking of the hydrogen bonds in which the NH_2 group is involved, facilitating the II→I transition.

(*ii*) While in form I, the dihedral rotation around the bond linking the aniline ring to the sulfone group is essentially frozen in a fixed position, a significant shift is observed in the dihedral positions in form II. Specifically, in form II, the maximum of probability changes from $\varphi_{C\text{–}C\text{–}S\text{–}C}$ = 85°, 282° at low temperatures to $\varphi_{C\text{–}C\text{–}S\text{–}C}$ = 98°, 266° at high temperatures (Figure 2.10.5h), while in form I, the maximum values remain centered at $\varphi_{C\text{–}C\text{–}S\text{–}C}$ = 95°, 266° (Figure 2.10.5g). As a result, with the increase in temperature, the $\varphi_{C\text{–}C\text{–}S\text{–}C}$ angles of form II approach the values noticed in form I.

The latter results suggest that, although the II → I transition was not directly observed in the simulations, the initial stages of this process can have been captured. It can therefore be speculated that the phase transition begins with an increase in the mobility of the aniline ring in form II, which, triggered by temperature, starts to adopt the orientation found in form I. Furthermore, a less efficient environment in form II to sustain the hydrogen bonds involving the NH_2 group can facilitate the molecular rearrangements necessary for the phase transition to occur.

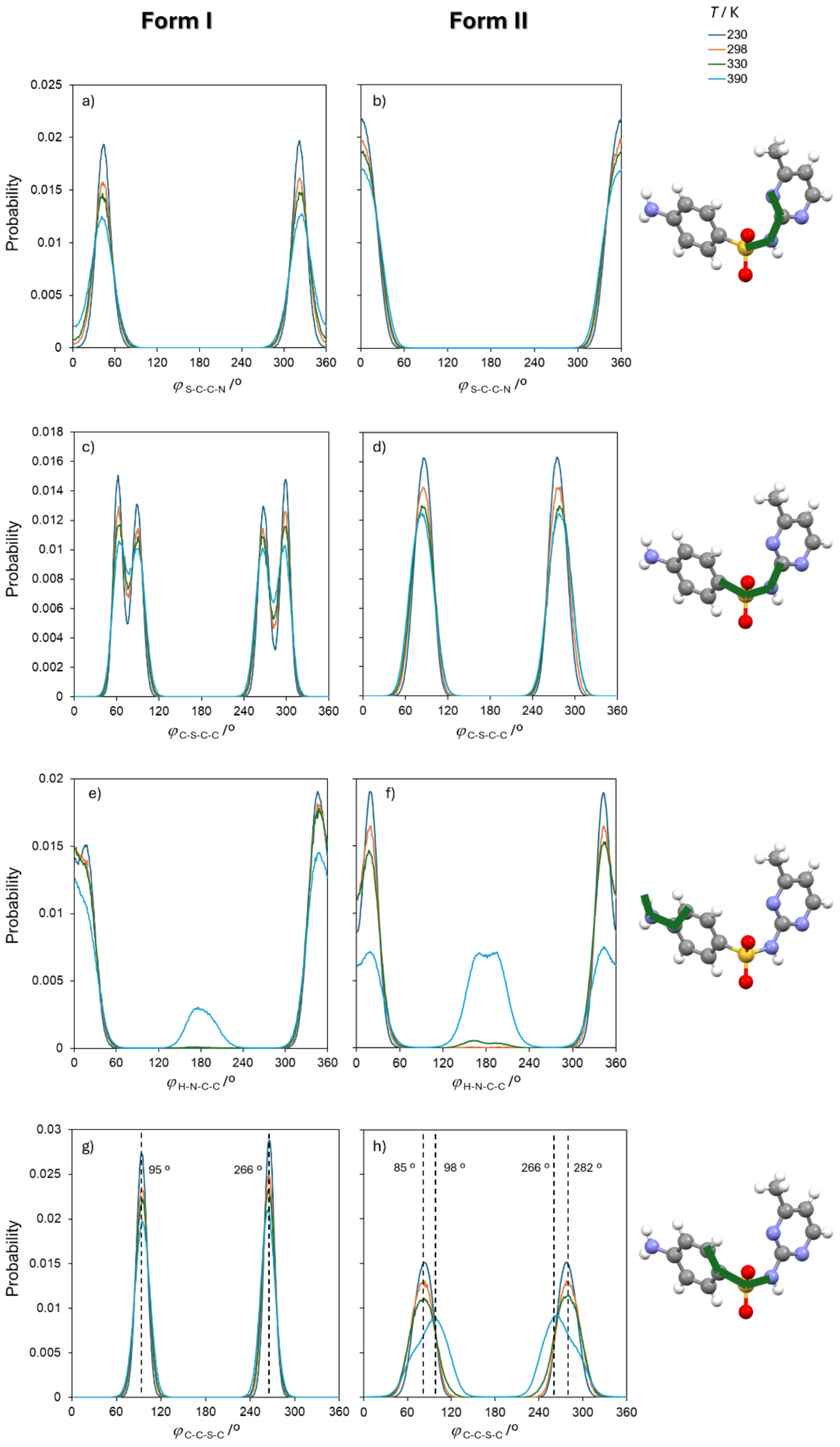

Figure 2.10.5. Distribution of the angle values, φ, for selected dihedrals of SMZ in form I (left) and II (right) as a function of temperature. The molecules on the right show the dihedral angle (highlighted in green) studied in the adjacent plots.

References

[1] R. W. Hockney and J. W. Eastwood, *Computer Simulation Using Particles*, A. Hilger, Bristol England ; Philadelphia, 1988.

[2] A. P. Thompson, H. M. Aktulga, R. Berger, D. S. Bolintineanu, W. M. Brown, P. S. Crozier, P. J. I. Veld, A. Kohlmeyer, S. G. Moore, T. D. Nguyen, R. Shan, M. J. Stevens, J. Tranchida, C. Trott and S. J. Plimpton, *Comput. Phys. Commun.*, 2022, **271**, 10817.

[3] C. E. S. Bernardes, *J. Chem. Inf. Model.*, 2022, **62**, 1471-1478.

[4] C. S. D. Lopes, M. E. Minas da Piedade and C. E. S. Bernardes, *Phys. Chem. Chem. Phys.*, 2025, DOI: 10.1039/D5CP01216C.

[5] F. Neese, *Wires Comput. Mol. Sci.*, 2012, **2**, 73-78.

[6] J. P. Perdew, K. Burke and M. Ernzerhof, *Phys. Rev. Lett.*, 1996, **77**, 3865-3868.

[7] J. P. Perdew, K. Burke and M. Ernzerhof, *Phys. Rev. Lett.*, 1997, **78**, 1396-1396.

[8] S. Grimme, S. Ehrlich and L. Goerigk, *J. Comput. Chem.*, 2011, **32**, 1456-1465.

[9] D. E. Woon and T. H. Dunning, *J. Chem. Phys.*, 1993, **98**, 1358-1371.

[10] C. M. Breneman and K. B. Wiberg, *J. Comput. Chem.*, 1990, **11**, 361-373.

[11] E. Caldeweyher, S. Ehlert, A. Hansen, H. Neugebauer, S. Spicher, C. Bannwarth and S. Grimme, *J. Chem. Phys.*, 2019, **150**.

[12] A. Najibi and L. Goerigk, *J. Chem. Theory Comput.*, 2018, **14**, 5725-5738.

[13] A. Najibi and L. Goerigk, *J. Comput. Chem.*, 2020, **41**, 2562-2572.

[14] C. R. Groom, I. J. Bruno, M. P. Lightfoot and S. C. Ward, *Acta Crystallogr.*, 2016, **B72**, 171-179.

[15] E. Tiesinga, P. J. Mohr, D. B. Newell and B. N. Taylor, *Rev. Mod. Phys.*, 2021, **93**, 033105

[16] C. E. S. Bernardes and A. Joseph, *J. Phys. Chem. A*, 2015, **119**, 3023-3034.

[17] C. F. Macrae, I. Sovago, S. J. Cottrell, P. T. A. Galek, P. McCabe, E. Pidcock, M. Platings, G. P. Shields, J. S. Stevens, M. Towler and P. A. Wood, *J. Appl. Crystallogr.*, 2020, **53**, 226-235.

[18] M. Abraham, A. Alekseenko, V. Basov, C. Bergh, E. Briand, A. Brown, M. Doijade, G. Fiorin, S. Fleischmann, S. Gorelov, G. Gouaillardet, A. Gray, M. E. Irrgang, F. Jalalypour, J. Jordan, C. Kutzner, J. A. Lemkul, M. Lundborg, P. Merz, V. Miletic, D. Morozov, J. Nabet, S. Pall, A. Pasquadibisceglie, M. Pellegrino, H. Santuz, R. Schulz, T. Shugaeva, A. Shvetsov, A. Villa, S. Wingbermuehle, B. Hess and E. Lindahl, *Zenodo*, 2020, DOI: 10.5281/zenodo.3460414, https://doi.org/10.5281/zenodo.10721181.

[19] M. J. Abraham, T. Murtola, R. Schulz, S. Páll, J. C. Smith, B. Hess and E. Lindahl,

SoftwareX, 2015, **1-2**, 19-25.

2.11. XtalPi: pseudo-supercritical path method (PSCP)

Contributed by Yizu Zhang, Zhuocen Yang, Qun Zeng, and Guangxu Sun

Traditional experimental determination of heat capacity across wide temperature ranges is time-consuming and expensive, particularly for new drug compounds and their polymorphs [1]. On one hand, the heat capacity could be derived through phonon calculations using methods like density functional theory harmonic approximation (DFT-HA), performed using the finite displacement method. The phonon density of states is constructed from the calculated frequencies, and the harmonic heat capacity is obtained through statistical thermodynamic integration of the vibrational partition function. DFT-based quasi-harmonic approximations (DFT-QHA) compute volume-dependent phonon frequencies at each volume point, enabling determination of the Grüneisen parameters and thermal expansion properties required for quasi-harmonic heat capacity evaluation. On the other hand, heat capacity can be evaluated via theoretical Gibbs free energy data derived from molecular dynamics (MD) methods, such as the pseudo-supercritical path method (PSCP) [2]. DFT-HA can predict heat capacity at constant pressure isobaric $Cv(T)$. However, for the more commonly used and experimentally accessible heat capacity at constant pressure isobaric $Cp(T)$, it is necessary to account for thermal expansion effects. DFT-QHA incorporates quasi-harmonic volume effects by accounting for volume-dependent phonon frequencies and thermal expansion. For anharmonic contributions beyond the quasi-harmonic framework, MD based methods such as PSCP are required to capture the full temperature-dependent vibrational properties. The relationship between Gibbs free energy $G(T)$ and $Cp(T)$ is established through fundamental thermodynamic principles:

$$Cp(T) = -T\left(\frac{\partial^2 G(T)}{\partial T^2}\right)_P \qquad \text{Eq (1)}$$

For solid organic compounds, the temperature dependence of heat capacity is commonly described by the empirical formula [3]:

$$Cp(T) = a + bT + c/T^2 \qquad \text{Eq (2)}$$

where:

- a (J/mol·K): the baseline constant term representing low-temperature vibrational contributions

- b (J/mol·K^2): linear temperature coefficient reflecting vibrational mode excitation
- c (J·K/mol): inverse-square temperature term accounting for low-temperature quantum effects

The functional form of Equation (2) is a widely adopted empirical representation for the temperature dependence of heat capacity in minerals and crystalline solids, commonly known as the Maier-Kelley equation established in 1932 [3]. This formulation incorporates the fundamental physics of lattice vibrations where the constant term a represents the classical high-temperature limit approaching the Dulong-Petit value, the linear term accounts for anharmonic effects and thermal expansion contributions, and the c/T^2 term captures the low-temperature quantum mechanical behavior of vibrational modes. Through integration of the thermodynamic relationship expressed in Equation (1), the corresponding Gibbs free energy expression [3] could be derived:

$$G(T) = ATlnT + BT^2 + CT + D/T + E \quad \text{Eq (3)}$$

where the parameters in Equation (3) relate to heat capacity coefficients through:

$$a = -A, b = -2B, c = -2D \quad \text{Eq (4)}$$

Based on literature review [4-8], typical parameter ranges for organic solid compounds are:

Parameter	Range	Physical Basis
a (J/mol·K)	50~150	Atomic contributions (~3-5 J/mol·K per atom)
b (J/mol·K^2)	0.1~0.8	Vibrational mode temperature dependence
c (J·K/mol)	1×10^4~1×10^6	Low-temperature quantum effects and molecular complexity

The fitting for Equation (3) is based on the PSCP Gibbs free energy data covering temperatures up to 450 K, bounded global optimization was conducted using Microsoft Excel Solver, employing the Generalized Reduced Gradient (GRG) Nonlinear optimization method to perform least-squares minimization. The global optimization was executed with a population size of 1,000 and a random seed value of 100. The upper and lower bounds of parameters A, B and D are derived from the rational ranges of a, b and c through the inverse relationship using

Equation (4). The difference between the fitting results against the PSCP Gibbs free energy data is within 1%. The further derived a, *b* and *c* parameters could thus be used to predict the heat capacity of the corresponding forms. Moreover, heat capacity values depend solely on the curvature of the free energy profile and not on its values, which are subject to inaccuracies in the force field's absolute potential energy. The PSCP calculations adopt a tailor-made force field based on putative low-energy crystals from a lightweight crystal structure prediction. However, the heat capacities predicted for the two forms are systematically overestimated by around 100 J/mol/K, a parallel calibration against the experimental data at 300 K is used to ensure quantitative accuracy. A comparison of experimentally measured and MD simulated unit cell volumes are illustrated in the following figure for Form I and II. Systematic overestimations of the absolute unit cell volume values and slopes are observed.

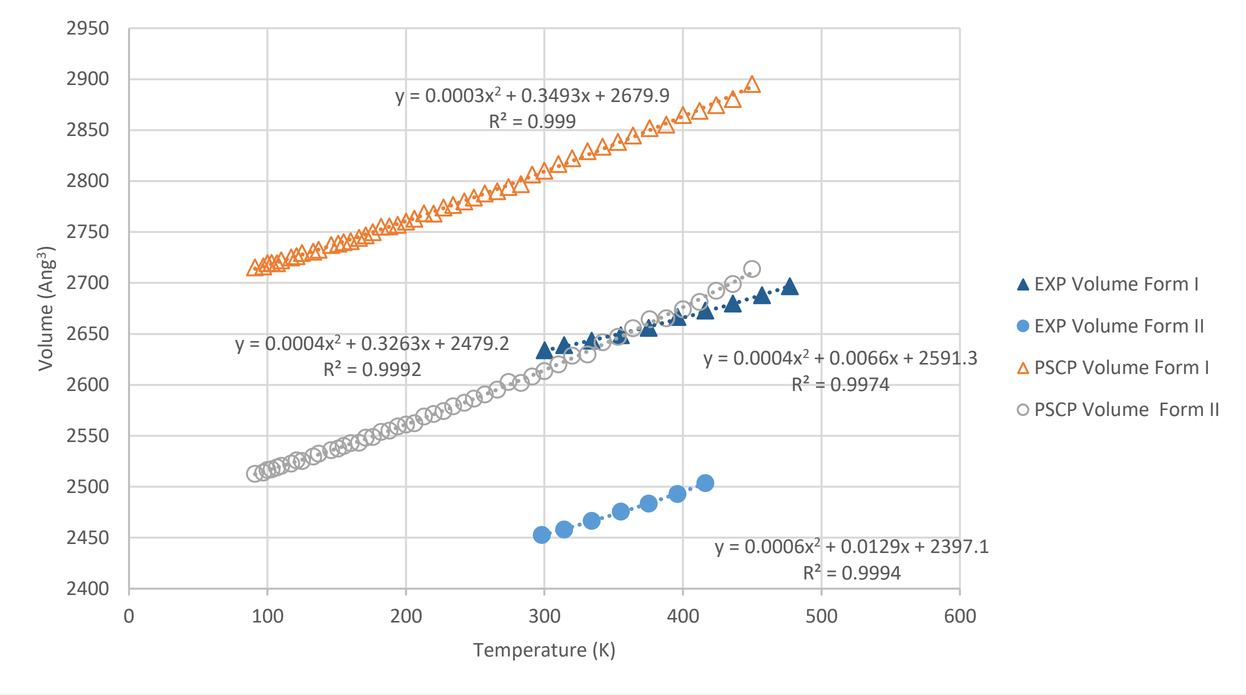

References

[1] Höhne, G., Hemminger, W., & Flammersheim, H. J. (2003). Differential Scanning Calorimetry. Springer.

[2] Yang, M., Dybeck, E., Sun, G., Peng, C., Samas, B., Burger, V. M., ... & Wood, G. P. (2020). Prediction of the relative free energies of drug polymorphs above zero kelvin. Crystal Growth & Design, 20(8), 5211-5224.

[3] Maier, C. G., & Kelley, K. K. (1932). An equation for the representation of high-temperature heat content data1. Journal of the American chemical society, 54(8), 3243-3246.

[4] NIST Chemistry WebBook. (2023). Retrieved from https://webbook.nist.gov/

[5] Chickos, J. S., & Acree, W. E. (2002). Enthalpies of vaporization of organic and organometallic compounds, 1880–2002. Journal of Physical and Chemical Reference Data, 31(2), 537-698.

[6] Domalski, E. S., & Hearing, E. D. (1996). Heat capacities and entropies of organic compounds in the condensed phase. Volume III. Journal of Physical and Chemical Reference Data, 25(1), 1-525.

[7] Barin, I. (1995). Thermochemical Data of Pure Substances (3rd ed.). VCH.

[8] Verevkin, S. P., Emel'yanenko, V. N., & Kozlova, S. A. (2008). Thermodynamic properties of mixtures containing ionic liquids. Journal of Physical and Chemical Reference Data, 37(3), 1345-1364.

2.12. MME@UCL – PGM + MBAR

Contributed by Edgar Olehnovics, Matteo Salvalaglio.

To compute the crossing temperature between Forms I and II of sulfamerazine, we have adopted a computational strategy that combines targeted Bennett acceptance ratio (BAR) calculations enabled by a probabilistic generative model (PGM)[1-3], and classical multistate Bennet acceptance ratio (MBAR) calculations. The targeted BAR approach was adopted to compute Helmholtz free energies of Form I and II supercells at a specific reference temperature, with respect to a common reference state described by a flat analytical distribution. These Helmholtz free energies were then converted to the corresponding estimates of Gibbs free energies by accounting for the constant pressure effects. MBAR was instead employed to directly compute Gibbs free energy differences in each polymorph as a function of temperature.

2.12.1. Helmholtz to Gibbs conversion

Potential energy function of polymorph i in units of energy, written as U_i, can be defined using the global potential energy surface $U(\boldsymbol{r},\boldsymbol{h})$, where $\boldsymbol{r}$ is a vector describing positions of all N atoms in the supercell, and $\boldsymbol{h} = h_{ij}$; $i,j = 1,2,3$ is the lower-triangular matrix of supercell lattice vectors:

$$U_i(\boldsymbol{r},\boldsymbol{h}) = U(\boldsymbol{r},\boldsymbol{h}) - \ln 1_i(\boldsymbol{r}) \quad ; \quad 1_i(\boldsymbol{r}) = \begin{cases} 1 \text{ if } \boldsymbol{r} \in \Omega_i \\ 0 \text{ if } \boldsymbol{r} \notin \Omega_i \end{cases} \qquad \text{(Eq. S1)}$$

In Eq.S1, $1_i(\boldsymbol{r})$ is an indicator function for supercell configurations $\boldsymbol{r}$ belonging to the

configurational space (Ω) of polymorph i. For a configuration microstate $\boldsymbol{x}$ (defined shortly) of an isothermal–isobaric ensemble (NPT), a normalised microstate probability distributions is a Boltzmann distribution $p_i(\boldsymbol{x}\,;T) = p_i(\boldsymbol{x}; N, P, T)|_{N,P}$ where we can fix both the number of atoms N and the chosen isotropic pressure P, leaving only temperature T is a free parameter:

$$p_i(\boldsymbol{x}\,;T) = \exp\big(g_i(T)\big)\,\mathcal{L}_i(\boldsymbol{x};T) \quad ; \quad \mathcal{L}_i(\boldsymbol{x};T) = \exp\big(-O_i(\boldsymbol{x};T)\big) \qquad (\text{Eq. S2})$$

In Eq.S2, $g_i(T)$ is unknown absolute configurational Gibbs free energy (FE) of the ensemble in adimensional unit k_BT, and $\mathcal{L}_i$ denotes the un-normalised likelihood of the Boltzmann distribution. Furthermore, $O_i(\boldsymbol{x};T)$ in Eq.S2 is the instantaneous enthalpy of microstate $\boldsymbol{x}$.

Given that $V = \det(\boldsymbol{h})$ is the instantaneous volume of the supercell and $\beta = k_BT$, there are two types of NPT ensembles that can be considered. Choosing $\boldsymbol{x} = [\boldsymbol{r}, V]$ is suitable for isotropic fluctuations of the box $\boldsymbol{h}$. For anisotropic fluctuations $\boldsymbol{x} = [\boldsymbol{r}, \boldsymbol{h}]$. The definition of instantaneous enthalpy depends on the choice of $\boldsymbol{x}$, and affects all downstream computations:[4]

$$O_i([\boldsymbol{r}, V];T) = \beta(U_i(\boldsymbol{r}, \boldsymbol{h}) + PV) \qquad (\text{Eq. S3})$$

$$O_i([\boldsymbol{r}, \boldsymbol{h}];T) = \beta(U_i(\boldsymbol{r}, \boldsymbol{h}) + PV) + \ln(h_{22}h_{33}^2) \qquad (\text{Eq. S4})$$

Writing f_i to represent the adimensional configurational Helmholtz free energy of a Canonical (NVT) ensemble of the same system in a fixed simulation box, and integrating $p_i(\boldsymbol{x}\,;T)$ over $\boldsymbol{r}$, gives two respective log (marginal) probabilities of either the volume (one-dimensional $\ln p_i(V;T)$), or the entire box (six-dimensional $\ln p_i(\boldsymbol{h};T)$), respectively:

$$\ln \int_{\Omega(V)} p_i([\boldsymbol{r}, V];T)\, d\boldsymbol{r} = \ln p_i(V;T) = g_i^{[V]}(T) - \beta PV - f_i(V,T) \qquad (\text{Eq. S5})$$

$$\ln \int_{\Omega(\boldsymbol{h})} p_i([\boldsymbol{r}, \boldsymbol{h}];T)\, d\boldsymbol{r} = \ln p_i(\boldsymbol{h};T) = g_i^{[h]}(T) - \beta P \det(\boldsymbol{h}) - f_i(\boldsymbol{h},T) - \ln(h_{22}h_{33}^2) \; (\text{Eq. S6})$$

Eq.S5 and Eq.S6 can be rearranged to give expressions that hold for any box, and allow Helmholtz FE to be converted into Gibbs FE:[5]

$$g_i^{[V]}(T) = f_i(V,T) + \beta PV + \ln p_i(V;T) \qquad (\text{Eq. S7})$$

$$g_i^{[h]}(T) = f_i(\boldsymbol{h},T) + \beta P \det(\boldsymbol{h}) + \ln p_i(\boldsymbol{h};T) + \ln(h_{22}h_{33}^2) \qquad (\text{Eq. S8})$$

In the current work, we made a consistent choice of using Eq.S7 for the Helmholtz to Gibbs conversion, and Eq.S3 for computing enthalpies inside MBAR. However, all NPT simulations that were preformed correspond to ensembles with microstates of the form $\boldsymbol{x} = [\boldsymbol{r}, \boldsymbol{h}]$. This is because a fully-flexible barostat was used in all NPT simulations.[4]

In relation to Eq.S7, we used normalised 1D histograms, fitted on the relevant NPT datasets, to model the volume distributions $\tilde{p}_i(V) \approx p_i(V)$, like in Ref.[5] Furthermore, like in Refs [5] and [6] we choose to evaluate Eq.S7 on V that corresponds to the average, most representative,

supercell box shape ($\boldsymbol{h}_0 = \langle \boldsymbol{h} \rangle_{NPT}$). Thus, the actual Helmholtz to Gibbs conversion that was used, is a combination of Eq.S7 and Eq.S8, that we limited to a chosen reference temperature T_{ref}:

$$\tilde{g}_i(T_{\mathrm{ref}}) \coloneqq f_i(\boldsymbol{h}_0, T_{\mathrm{ref}}) + \beta_{\mathrm{ref}} P \det(\boldsymbol{h}_0) + \ln \tilde{p}_i(V_0) \quad ; \quad V_0 = \det(\boldsymbol{h}_0) \qquad (\mathrm{Eq.}S9)$$

Our preliminary observations in sulfamerazine Forms I, II, III, and IV indicated that estimates of Gibbs FE differences $\Delta g_{ij} = g_i - g_j$ were largely unaffected by the choice Eq.S7 vs. Eq.S8 (for the f to g conversion), or the choice of Eq.S3 vs. Eq.S4 (for the enthalpies inside MBAR), provided that $f_i(\boldsymbol{h}_0, T_{\mathrm{ref}})$ is computed using a well-converged reference box in each system. That being said, in further work it would be useful to test our current method of choice (Eq.S9) against direct and rigorous estimates of $g_i^{[h]}(T_{\mathrm{ref}})$ with an appropriate PGM.[7]

2.12.2. Gibbs FE differences as a function of temperature with MBAR

Sampling $p_i(\boldsymbol{x}\,; T)$ in Eq.S2 at K different temperatures $T_1, \dots, T_K$ allows to define a normalised mixture distribution $p_i^M(\boldsymbol{x}) = K^{-1} \sum_{k=1}^{K} p_i(\boldsymbol{x}\,, T_k)$. This mixture distribution allows to write the following ensemble average, where the weight $w_i^M = \exp(-C)\, p_i^M$ are proportional to p_i^M up to a global constant labelled as C:[8]

$$g_i(T) = -\ln \int d\boldsymbol{x}\, \mathcal{L}_i(\boldsymbol{x}, T) = -\ln \int d\boldsymbol{x} \frac{p_i^M(\boldsymbol{x})}{p_i^M(\boldsymbol{x})} \mathcal{L}_i(\boldsymbol{x}, T) \cong -\ln \langle \frac{\mathcal{L}_i(T)}{w_i^M} \rangle_{p_i^M} + C \quad (\mathrm{Eq.S10})$$

The ensemble average in Eq.S10 is equivalent to exponential averaging,[9] where the weight w_i^M can be moved into the exponent of the numerator. The empirical average gives a curve $\Delta g_i(T) = g_i(T) + C$, where the unknown constant offset C can be set to any value. We can thus choose $C = -g_i(T_{\mathrm{ref}})$, resulting in $\Delta g_i(T_{\mathrm{ref}}) = 0$, for any choice of T_{ref}. To obtain the correct set of self-consistent weights w_i^M, it is first necessary to accurately converge the Gibbs FE differences $\Delta g_i^{kk'} = g_i(T_k) - g_i(T_{k'})$ between the discrete ensembles sampled at the K different temperatures (of the same Form i). This was done using the *pymbar* function compute_free_energy_differences.[10,8]

In this work, we have performed simulations at seven discrete temperatures (i.e. $K = 7$), obtaining NPT datasets for the mixture distribution sampled at $T =$ 200, 250, 300, 350, 400, 450, and 500 K, at 1 atmospheric pressure (i.e., $P = 1\mathrm{atm}$). It was found that sampling timescales ranging from 1 to 9 ns at each temperature yielded sufficiently consistent and accurate results, as shown in Figure S4. Equipped with the well-converged weights w_i^M, Eq.S10 was evaluated at arbitrary temperatures using compute_perturbed_free_energies functionality of *pymbar*, which also provides analytical error estimates.[11] This produced a curve $\Delta g_i(T)$, that was shifted such that $\Delta g_i(T_{\mathrm{ref}}) = 0$. In the current work $T_{\mathrm{ref}} = 300\mathrm{K}$ was chosen for

convenience. The process was repeated for different Forms $i = \mathrm{I, II, III, IV}$, giving four different curves, all intersecting at T_{ref}. Equipped with $\tilde{g}_i(T_{\mathrm{ref}})$ computed from the previous section, allowed to recover the estimates of interest ($g_i(T)\ \forall\ i$):

$$g_i(T) \approx [g_i(T) - g_i(T_{\mathrm{ref}})] + \tilde{g}_i(T_{\mathrm{ref}}) \qquad (\mathrm{Eq.S11})$$

The accuracy of $g_i(T)$ in Eq.S11 depends on the error on the estimate of f_i, added to the errors along the curve $\Delta g_i(T)$, where both error estimates are analytical. Estimates of f_i were computed robustly, without assumptions using targeted BAR calculations based on the PGM method, detailed in Ref.[2,3].

2.12.3. Molecular Dynamics Simulations Setup

MD simulations were performed using OpenMM with the OPLS-AA force field, without constraints. The forcefield parameters are available on github/E471r/FEcrys/O/MM/molecules/smz/misc/OPLS_smz.itp.

Lennard-Jones interactions had a switching function set between 0.475 nm and 0.5 nm, with dispersion correction being active beyond these distances. Coulombic interactions were solved using the PME algorithm, with a real-space cutoff set to 0.5 nm and the EwaldErrorTolerance parameter set to 0.0001. The dynamics were integrated at a constant temperature with a timestep of 2 fs, using a Langevin integrator (LangevinMiddleIntegrator), with a friction coefficient set to 1 ps^{-1}. Finite pressure simulations used the Monte Carlo flexible barostat (MonteCarloFlexibleBarostat), perturbing all six degrees of freedom of the simulation box, every 25 timesteps.[4]

The main results reported in the paper were based on sulfamerazine supercells containing 24 molecules. Other supercell sizes, used for assessing the consistency of Helmholtz free energy differences as a function of system size, are reported in Figure S5B. Figure S7 validates the idea of choosing any T_{ref} for which standard errors on the Helmholtz free energy estimates can be minimised. In further work, this observation is equally useful for minimising error on any explicit Gibbs FE estimates between polymorphs.

An ideal supercell was first equilibrated at 300 K and 1 atm for 5 ns, to obtain a supercell with a simulation box that best matches the average simulation box. A 60 ns NVT simulation, at 300 K (T_{ref}), was then sampled in this simulation box to obtain the training and validation data for the PGM to enable the targeted BAR estimates of the relevant Helmholtz free energy. To obtain the temperature-dependent Gibbs free energy differences between the same polymorph samples at different temperatures using MBAR, a series of 10 ns-long simulations was run at temperatures of $200, 250, 300, 350, 400, 450, 500$ K, and 1 atm.

2.12.4. Supplementary Results

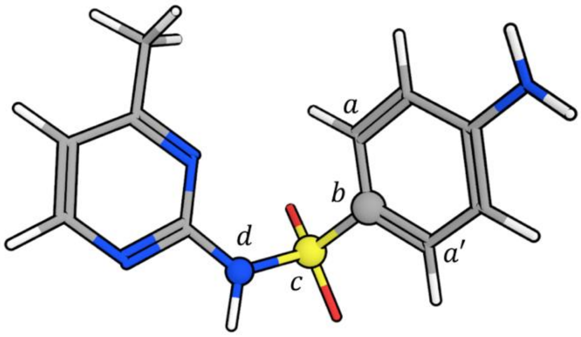

Figure 2.12.1. *Sulfamerazine molecular structure representation convention.* The molecule contains 30 atoms. For the representation layer of the PGM, the Cartesian block was set to the atoms labelled b, c, d, with the position of atom c describing the translation of the molecule[2,3]. The rotation of the aniline ring was described by torsional angles $[a, b, c, d]$ and $[a', b, c, d]$

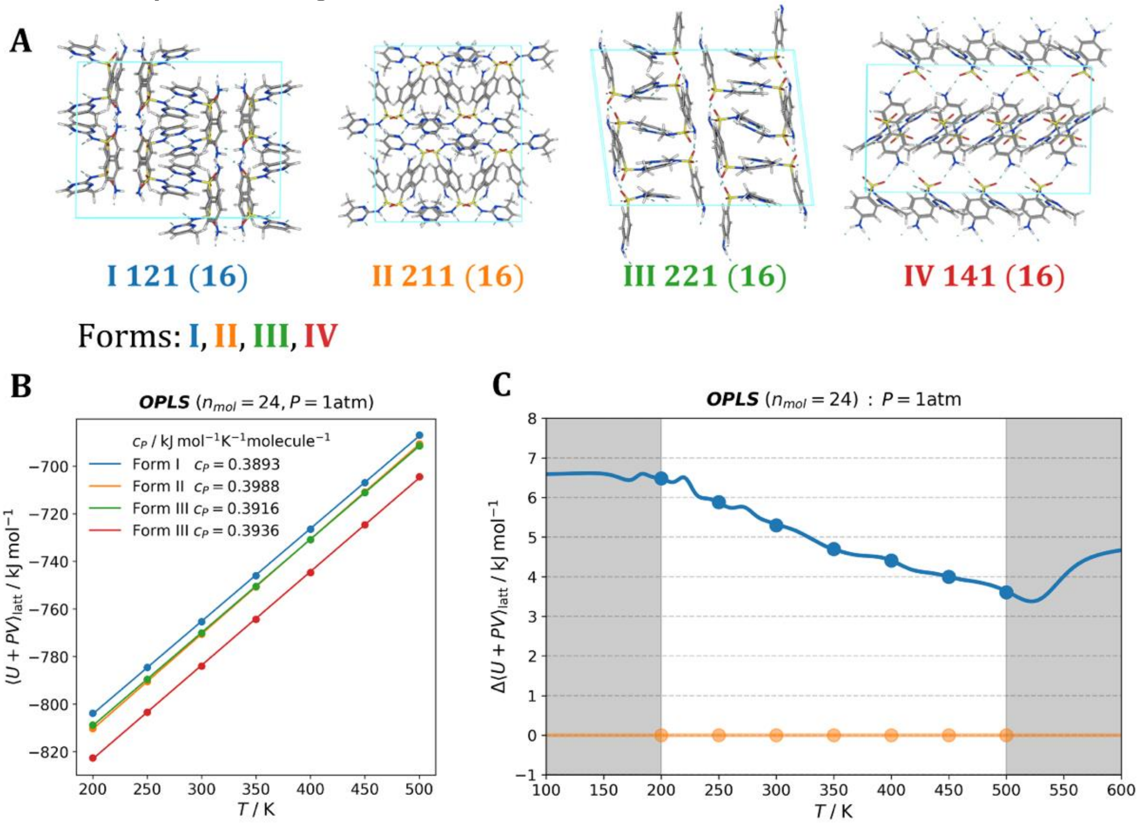

Figure 2.12.2. *Average enthalpies and heat capacity estimates.* **A.** Supercells of the four Forms of sulfamerazine. **B.** Average enthalpies as a function of temperature are plotted as solid circles. Heat capacities, listed on the top left, are estimated as the slopes of the linear regressions of the average enthalpies. **C.** Enthalpy difference between forms I and II as a function of temperature. The points refer to a direct average of the sampled enthalpies from individual NPT datasets. The solid lines correspond to the expected average enthalpies at intermediate, non-sampled temperatures, obtained with MBAR function `compute_expectations_inner`.

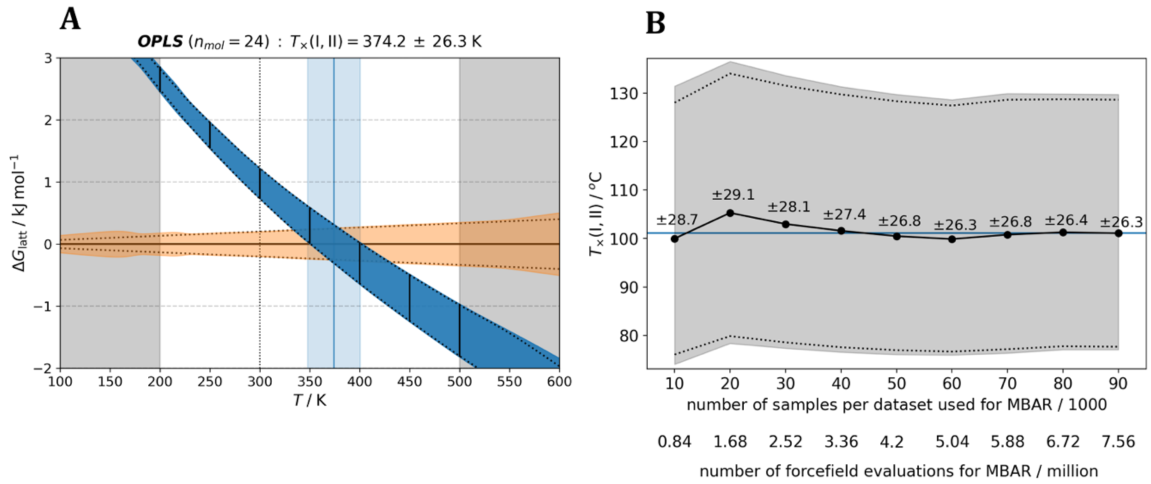

Figure 2.12.3. *Crossing temperature between sulfamerazine Forms I and II.* **A.** Gibbs FE differences between the two Forms I (blue) and II (orange), as a function of temperature. The reference temperature, $T_{\mathrm{ref}} = 300\mathrm{K}$, is indicated with a vertical dotted line. The total error bars are represented in the plot using shaded regions. Standard errors from PGM alone are plotted using dotted lines, highlighting that the MBAR interpolation across temperatures contributed negligibly to the overall uncertainty of the estimate. **B.** Estimates of the crossing temperature between the two Forms as a function of the number of samples per temperature used to carry out the MBAR calculations. The error bars (filled in region vs. dotted lines) have the same meaning as in A. Since all the estimates are clearly within the overall error of each other, it can be concluded that as few as 840,000 force field evaluations are sufficient to estimate the crossing temperature consistently.

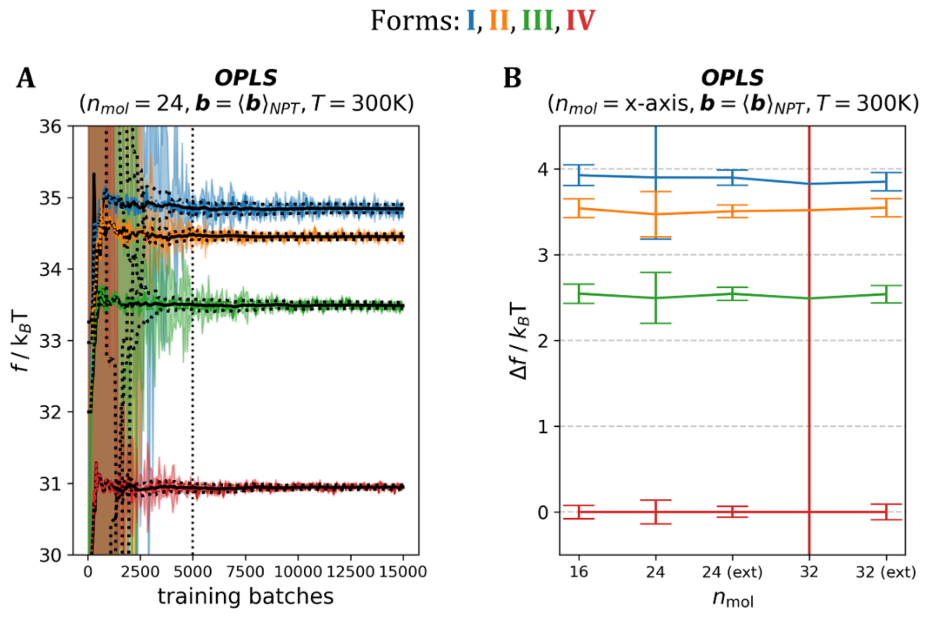

Figure 2.12.4. *Helmholtz FE estimates in four different Forms of sulfamerazine.* **A.** Estimates of the *absolute* Helmholtz FEs at temperature T_{ref} ($f(N, \langle \boldsymbol{h} \rangle_{NPT}, T)$ in Eq.S6), as a function of PGM training progress. The FE estimates are plotted in black (dotted: standard error). All four estimates can be considered converged after 5000 training batches; however, the analytical error bars (black dotted lines) continued to decrease with further training. **B.** Consistency of FE differences with system and dataset size. The supercell size is reported in terms of the number of molecules on the x-axis. All metastable states were sampled by MD for 30 ns. The label *(ext)* refers to the estimates obtained for the same supercells, but using an extended datasets extracted from 60 ns simulations, corresponding to 15,000

training batches. PGMs were trained on 80% of the available MD data, while FEs were computed using BAR on the remaining 20% of the data.

Table 2.12.1. **Conformational flexibility sampled by MD.** The table reports the conformational flexibility observed during 10-ns-long NPT simulations of supercells containing Forms I and II, each with 24 molecules. During all simulations, none of the molecules rotated by significantly large angles, indicating that, within a 10 ns timescale, the crystals were not starting to melt, even at 500 K. The columns refer to three types of observed conformational transitions. The slowest intramolecular conformational change was the 180-degree rotation of the symmetric aniline ring (discussed in Figure 2.12.5). The PGM was trained only on data sampled at $T_{\text{ref}} = 300\,\text{K}$, where all metastable states associated with CH_3 and NH_2 rotations were sampled ergodically.

**Form I** @ 1 atm Temperature [K]	Packing sliding/translation of layers	Conformations CH_3 rotation	NH_2 rotation	Aniline ring rotation
200	N	Y	N	N
250	N	Y	N	N
300	N	Y	Y	N
350	N	Y	Y	N
400	Y	Y	Y	N
450	YY	Y	Y	N
500	YYY	Y	Y	N
**Form II** @ 1atm Temperature [K]	Packing sliding/translation of layers	Conformations CH_3 rotation	NH_2 rotation	Aniline ring rotation
200	N	Y	Y	N
250	N	Y	Y	N
300	N	Y	Y	N
350	N	Y	Y	N
400	N	Y	Y	N
450	N	Y	Y	Y
500	N	Y	Y	YY

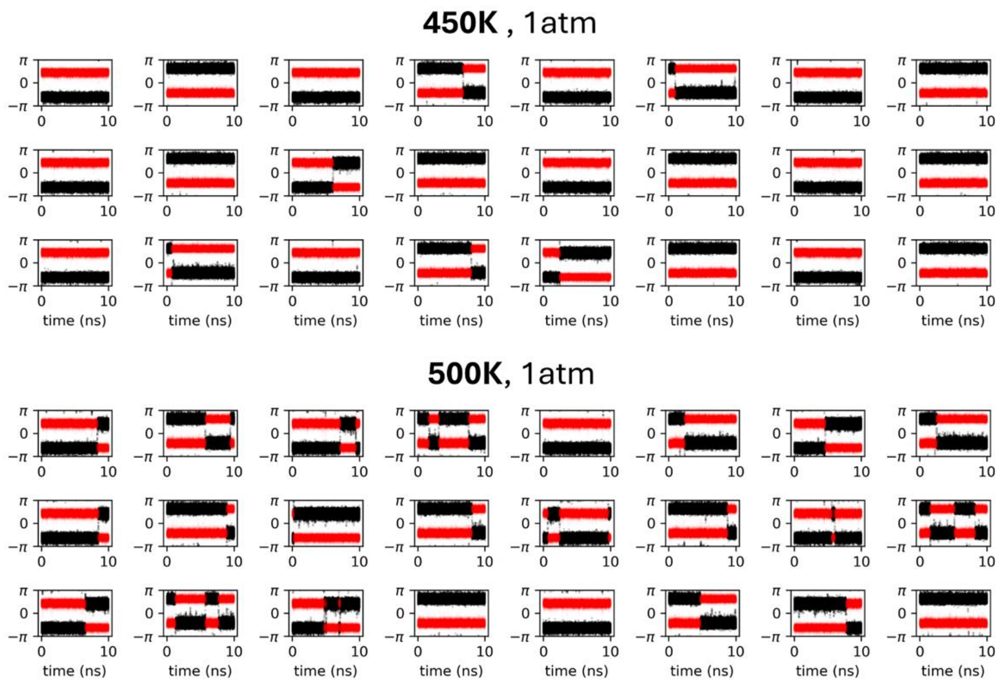

Figure 2.12.5. *Rotation of the symmetric aniline ring in Form II at higher temperature*. Each plot reports instantaneous configurations of the aniline ring of one of the molecules within a Form II supercell, containing a total of 24 molecules. The colors black and red represent the trajectory of two chemically equivalent torsional angles $[a, b, c, d]$ and $[a', b, c, d]$ respectively (labelled in Figure 2.12.1). The plots illustrate the rare event rotation of the aniline ring during NPT simulations, with 180-degree rotations

being more frequent at higher temperatures. Both conformations are energetically equivalent, due to the chemical equivalence of this ring around the S-C bond. At T between 200 and 400 K, no rotations of the ring were observed during 10 ns-long simulations.

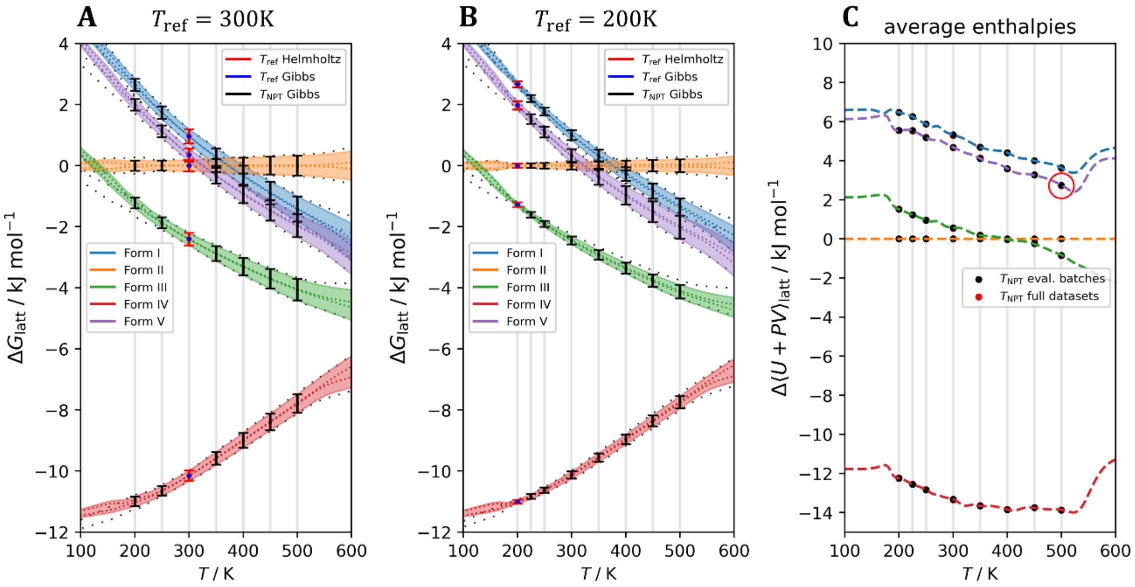

Figure 2.12.6. *Crossing temperatures between sulfamerazine Forms I-V.* **A.** Gibbs FE differences as a function of temperature, with T_{ref} =300K (reference temperature used for PGM calculations). Each coloured curve is based on 3.78×10^6 forcefield evaluations for MBAR, with grey dotted lines comparing to a low-data regime (0.42×10^6 forcefield evaluations for MBAR). The coloured dotted lines report the standard error from MBAR alone. **B.** Gibbs FE differences as a function of temperature, with T_{ref} =200K (reference temperature used for PGM calculations). Each coloured curve is based on 5.04×10^6 forcefield evaluations for MBAR, with grey dotted lines comparing to a low-data regime (0.56×10^6 forcefield evaluations for MBAR). The coloured dotted lines report the standard error from MBAR alone. **C.** Differences in average enthalpy between the NPT datasets as a function of temperature, interpolated with MBAR at higher-data regime. PGM calculations of Helmholtz FE differences: At 300K (**A**), 1.5×10^6 forcefield evaluations were involved during the entire training period, in each Form, training on data with random permutations of hydrogen atoms of the NH2 and CH3 groups (i.e., both groups *symmetry randomized*). The latter step was largely necessary in Form IV, due to slow NH2 rotation at 300K. At 200K (**B**), 1×10^6 forcefield evaluations were involved during the entire training period, in each Form, training on data with *symmetry randomised* NH2 group, and *symmetry reduced* CH3 group. The figure (**A** vs. **B**) shows self-consistency of choosing any T_{ref}, with lower temperature enabling lower PGM error, in turn reducing the total error. The figure also validates the symmetry adjustment protocol, that is necessary for preprocessing NVT data containing symmetric rare events, prior to training the current version of the PGM (not permutationally invariant) on fully ergodic data. 60ns of NVT data (training and validation) was used for each Helmholtz FE estimate (red).

References

[1] Olehnovics, E.; Liu, Y. M.; Mehio, N.; Sheikh, A. Y.; Shirts, M. R.; Salvalaglio, M. Assessing the Accuracy and Efficiency of Free Energy Differences Obtained from Reweighted Flow-Based Probabilistic Generative Models. *J. Chem. Theory Comput.* **2024**, *20* (14), 5913–5922. https://doi.org/10.1021/acs.jctc.4c00520.

[2] Olehnovics, E.; Liu, Y. M.; Mehio, N.; Sheikh, A. Y.; Shirts, M. R.; Salvalaglio, M. Accurate Lattice Free Energies of Packing Polymorphs from Probabilistic Generative Models. *J. Chem. Theory Comput.* **2025**, *21* (5), 2244–2255. https://doi.org/10.1021/acs.jctc.4c01612.

[3] Olehnovics, E.; Liu, Y. M.; Mehio, N.; Sheikh, A. Y.; Shirts, M.; Salvalaglio, M. Lattice Free Energies of Molecular Crystals Using Normalizing Flow. *ChemRxiv* **2025**. https://doi.org/10.26434/chemrxiv-2025-92x2f.

[4] Vandenhaute, S.; Rogge, S. M. J.; Van Speybroeck, V. Large-Scale Molecular Dynamics Simulations Reveal New Insights Into the Phase Transition Mechanisms in MIL-53(Al). *Front. Chem.* **2021**, *Volume 9-2021*. https://doi.org/10.3389/fchem.2021.718920.

[5] Cheng, B.; Ceriotti, M. Computing the Absolute Gibbs Free Energy in Atomistic Simulations: Applications to Defects in Solids. *Phys. Rev. B* **2018**, *97* (5), 054102. https://doi.org/10.1103/PhysRevB.97.054102.

[6] Yang, M. J.; Dybeck, E.; Sun, G. X.; Peng, C. W.; Samas, B.; Burger, V. M.; Zeng, Q.; Jin, Y. D.; Bellucci, M. A.; Liu, Y.; Zhang, P. Y.; Ma, J.; Jiang, Y. A.; Hancock, B. C.; Wen, S. H.; Wood, G. P. F. Prediction of the Relative Free Energies of Drug Polymorphs above Zero Kelvin. *Crystal Growth & Design*, 2020, *20*, 5211–5224. https://doi.org/10.1021/acs.cgd.0c00422.

[7] Wirnsberger, P.; Ibarz, B.; Papamakarios, G. Estimating Gibbs Free Energies via Isobaric-Isothermal Flows. *Mach. Learn. Sci. Technol.* **2023**, *4* (3), 035039. https://doi.org/10.1088/2632-2153/acefa8.

[8] Shirts, M. R. Reweighting from the Mixture Distribution as a Better Way to Describe the Multistate Bennett Acceptance Ratio, 2017. https://arxiv.org/abs/1704.00891.

[9] Zwanzig, R. W. High-Temperature Equation of State by a Perturbation Method. I. Nonpolar Gases. *J. Chem. Phys.* **1954**, *22* (8), 1420–1426. https://doi.org/10.1063/1.1740409.

[10] Shirts, M. R.; Chodera, J. D. Statistically Optimal Analysis of Samples from Multiple Equilibrium States. *J. Chem. Phys.* **2008**, *129* (12), 124105. https://doi.org/10.1063/1.2978177.

[11] Chodera, J. D.; Swope, W. C.; Noé, F.; Prinz, J.-H.; Shirts, M. R.; Pande, V. S. Dynamical Reweighting: Improved Estimates of Dynamical Properties from Simulations at Multiple Temperatures. *J. Chem. Phys.* **2011**, *134* (24), 244107. https://doi.org/10.1063/1.3592152.

2.13. Zero-point energies

Table 2.13.1. Zero point energies (in kJ mol^{-1}) for forms I and II of SMZ

Group	Form I	Form II	Form II - Form I	
PriceUCL	591.21	594.51	3.30	Harmonic calcs with poor Cv so inaccurate
RussoGSK	589.31	592.34	3.03	Harmonic calcs with poor Cv so inaccurate
XtalPi	590.52	594.09	3.57	Harmonic calcs with poor Cv so inaccurate
Arhangelskis	509.12	592.84	2.72	Harmonic phonons
TCG-UNITO	591.84	594.76	2.92	ZPE of fully optimized structure (electronic minimum)
Boese light	613.85	616.08	2.23	Min of EOS fits at 0 K
Boese light	614.00	616.37	2.37	ZPE of fully optimized structure (electronic minimum)
Boese tight	613.57	615.52	1.95	Min of EOS fits at 0 K, tight electronic energies and light phonons

2.14. Lattice energy differences of forms I and V

Only a few groups performed calculations on form V, which was discovered almost a year after the initial lattice energy calculations, mainly because the differences in the structure are so small that the energy differences would probably be within the numerical error in the calculations. The calculations that were done (Table 2.14.1) support this assumption.

As the two structures mainly differ in one of the two types of interfaces between the hydrogen bonded layers (Figure 2 m/s), and the phonons describing moving the layers relative to each other are likely to be low frequency and very anharmonic, as seen in MD simulations (SI 2.12). The temperature-dependent free energy differences are relatively more important (Figure 2.9.1 and Figure 2.12.6).

Table 2.14.1 Lattice energy differences between forms I and V.

Group	$U_{latt}(V)$-$U_{latt}(I)$ / kJ mol^{-1}	Notes
Price UCL	0.03	Form I more stable by plane wave (PBE+TS) CASTEP
Price UCL	–0.44	Form V more stable by atomic orbital(PBE+XDM) FHI-AIMS
XtalPi	0.05	Form I more stable
Boese light	0.3	Form I more stable
Boese tight	0.0	Both forms have the same stability
Whitfield	–0.21	Form V more stable by fixed 150 K cell, PBE-D3
Whitfield	–0.44	Form V more stable by fixed 150 K cell, SCAN-rVV10
AMS	0.076	Form I is more stable than form V (this includes all single points and single molecule corrections)
CB@Lisbon	-0.4 ± 2.0	MD forcefield
